# Pericytes contribute to pulmonary vascular remodeling via HIF2α signaling

**DOI:** 10.1038/s44319-023-00054-w

**Published:** 2024-01-19

**Authors:** Hyunbum Kim, Yu Liu, Jiwon Kim, Yunhye Kim, Timothy Klouda, Sudeshna Fisch, Seung Han Baek, Tiffany Liu, Suzanne Dahlberg, Cheng-Jun Hu, Wen Tian, Xinguo Jiang, Kosmas Kosmas, Helen A Christou, Benjamin D Korman, Sara O Vargas, Joseph C Wu, Kurt R Stenmark, Vinicio de Jesus Perez, Mark R Nicolls, Benjamin A Raby, Ke Yuan

**Affiliations:** 1 Division of Pulmonary Medicine, Boston Children’s Hospital, Harvard Medical School, Boston, MA USA; 2 Stanford Cardiovascular Institute; Department of Medicine, Stanford University, Stanford, CA 94305 USA; 3 Department of Medicine, Brigham and Women Hospital, Boston, MA USA; 4 Cardiovascular Pulmonary Research Laboratories, Division of Pulmonary Sciences and Critical Care Medicine, Division of Pediatrics-Critical Care, Departments of Medicine and Pediatrics, University of Colorado, Anschutz Medical Campus, Aurora, CO USA; 5 Division of Pulmonary, Allergy and Critical Care Medicine, Dept of Medicine, Stanford University, Stanford, CA USA; 6 Department of Pediatric Newborn Medicine, Brigham and Women’s Hospital, Harvard Medical School, Boston, MA USA; 7 Division of Allergy/Immunology and Rheumatology, Department of Medicine, University of Rochester Medical Center, Rochester, NY 14623 USA; 8 Division of Pathology, Boston Children’s Hospital, Harvard Medical School, Boston, MA USA

**Keywords:** Pericyte, Vascular Remodeling, HIF2α, Pulmonary Hypertension, Cardiovascular System, Molecular Biology of Disease, Vascular Biology & Angiogenesis

## Abstract

Vascular remodeling is the process of structural alteration and cell rearrangement of blood vessels in response to injury and is the cause of many of the world’s most afflicted cardiovascular conditions, including pulmonary arterial hypertension (PAH). Many studies have focused on the effects of vascular endothelial cells and smooth muscle cells (SMCs) during vascular remodeling, but pericytes, an indispensable cell population residing largely in capillaries, are ignored in this maladaptive process. Here, we report that hypoxia-inducible factor 2α (HIF2α) expression is increased in the lung tissues of PAH patients, and HIF2α overexpressed pericytes result in greater contractility and an impaired endothelial-pericyte interaction. Using single-cell RNAseq and hypoxia-induced pulmonary hypertension (PH) models, we show that HIF2α is a major molecular regulator for the transformation of pericytes into SMC-like cells. Pericyte-selective HIF2α overexpression in mice exacerbates PH and right ventricular hypertrophy. Temporal cellular lineage tracing shows that HIF2α overexpressing reporter NG2+ cells (pericyte-selective) relocate from capillaries to arterioles and co-express SMA. This novel insight into the crucial role of NG2+ pericytes in pulmonary vascular remodeling via HIF2α signaling suggests a potential drug target for PH.

## Synopsis

HIF2α signaling plays an important role in the transition of pericytes into smooth muscle cell-like cells in pulmonary hypertension. Hence, targeting HIF2α in pericytes may be a potential therapeutic approach for pulmonary hypertension.

- Overexpressing HIF2α selectively in pericytes results in vigorous muscularization of distal microvessels and loss of endothelial integrity.
- Deletion of HIF2α in pericytes alleviates muscularization and remodeling of precapillary arterioles.
- Lineage tracing of the reporter color labeled cells show that HIF2α overexpressing NG2 cells gain a SMC-like phenotype during disease progression.
- Inhibition of HIF2α downstream signaling pathways using AMD3100 protects mice from hypoxia induced pulmonary hypertension.

## Abstract (web summary)

HIF2α signaling plays an important role in the transition of pericytes into smooth muscle cell-like cells in pulmonary hypertension. Hence, targeting HIF2α in pericytes may be a potential therapeutic approach for pulmonary hypertension.

## Graphical abstract

**Figure:**
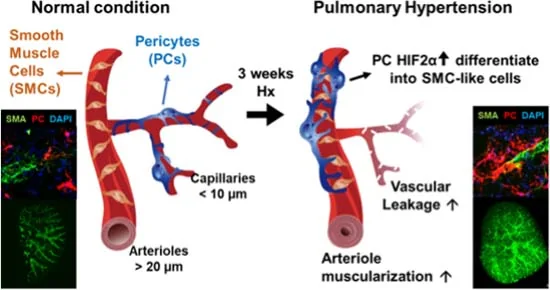

**Figure:**
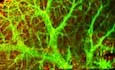

## Introduction

Vascular remodeling is an active process of the structural alteration of blood vessels that can adversely affect arterial function and structure. Vascular remodeling may play a central role in the pathogenesis of a wide spectrum of cardiovascular disorders, including atherosclerosis, systemic arterial hypertension, coronary artery disease, peripheral vascular disease, and pulmonary arterial hypertension (PAH). Pulmonary vascular remodeling involves the loss of small peripheral arterioles and the structural changes in intima, media and adventitia, often with the interplay of inflammatory cells and deposition of extracellular matrix scaffolding (Abe et al, 2010; Humbert et al, 2019; Stacher et al, 2012). Increased vasoconstriction resulting from remodeling processes leads to PAH, a life-threatening disease associated with a progressive elevation in pulmonary arterial pressure and right ventricular failure (Simonneau et al, 2019). Dynamic intercellular communications among endothelial, smooth muscle, fibroblasts and immune cells have been studied in intima thickening that occludes the lumen of vessels with a diameter larger than 200 µm. However, whether capillary lesions lead to vascular remodeling remains unclear.

Pericytes, one of the key components of capillaries, is closely associated with the thin layer of endothelial cells (ECs) and are essential for endothelial function, integrity, and homeostasis (Bergers and Song, 2005; Stratman et al, 2009; Yuan et al, 2019). They are also multipotent perivascular mural cells embedded in the basement membrane and may differentiate into vascular muscle cells under stress or injury (Diaz-Flores et al, 2009; Yamagishi and Imaizumi, 2005). The loss or relocation of pericytes is one of the key pathological features in pulmonary hypertension (PH), indicating their critical role in vasomotor tone and the structural support of microvessels (Ricard et al, 2014; Yuan et al, 2015). Moreover, our group observed that pericytes isolated from human PAH lungs had similar genetic profiles with smooth muscle cells (SMCs) and one of their commonly activated pathways was related to one of the HIF2α-regulated transcriptional factors that were associated with pulmonary arteriolar muscularization (Yuan et al, 2020).

Chronic exposure to hypoxia (Hx) in mice (3 weeks at FiO_2_ 10%) causes PH with a release of mitogen growth factors and facilitates a degree of muscularization and remodeling in pulmonary arteries. Abundant evidence has shown that dysregulation of hypoxia-inducible factors (HIF) activities is essential for developing chronic Hx-induced PH (Chan et al, 2021, Cowburn et al, 2016; Dai et al, 2016; Hu et al, 2019; Kim et al, 2013; Tang et al, 2018). HIFs are a family of transcription factors that function as master regulators of oxygen homeostasis involving an O_2_-sensitive α-subunit (mainly HIF1α and HIF2α) (Dengler et al, 2014; Haase, 2009; Liao et al, 2007). Although HIF1α and HIF2α show similarities in their DNA binding and dimerization domains, they exhibit different vascular morphogenetic processes under hypoxic stress(Brusselmans et al, 2003; Howard et al, 2012; Schultz et al, 2006; Shimoda and Semenza, 2011; Yuan et al, 2013). Global HIF1α deletion or antisense oligonucleotides in mice did not prevent hypoxia-induced PH (Hu et al, 2019). However, HIF2α accumulates/dimerizes with HIF1β in the nucleus and plays a significant role in vascularization and pulmonary development under hypoxia (Compernolle et al, 2002; Scortegagna et al, 2003). HIF2α accumulation in the pulmonary ECs is sufficient to cause abnormal muscularization of peripheral pulmonary arteries and right ventricular hypertrophy (RVH) (Kapitsinou et al, 2016). Severe vascular defects in mice are ascribed to the activation of HIF2α, whereas endothelial-specific knockout (KO) of the HIF2α gene ameliorates the development and progression of PH (Cowburn et al, 2016; Dai et al, 2016; Hu et al, 2019; Tang et al, 2018). The animals that have upregulation of HIF2α in SMCs undergo abnormal pulmonary vascular remodeling and the early onset of PH (Chan et al, 2021; Howard et al, 2012). Although the contribution of pulmonary ECs and SMCs in large arteriolar remodeling has been highlighted, very little is known regarding the role of HIF2α in pericytes during capillary remodeling.

In this study, we examined pericyte contribution to vascular remodeling via HIF2α activation in pulmonary circulation in human and mice. Pericytes expressing upregulated HIF2α were identified in idiopathic PAH patients by single-cell RNA sequencing (scRNA-seq) and clinical PAH tissue samples. HIF2α overexpression in human lung pericytes had greater contractility and impaired endothelial-pericyte tube formation. We also evaluated the role of HIF2α in mouse PH vascular remodeling using scRNA-seq and cell lineage tracing approaches. Generating three novel transgenic mouse models, we demonstrated that (1) the overexpression of HIF2α selectively in pericytes resulted in vigorous muscularization of distal microvessels and loss of endothelial integrity; (2) the deletion of HIF2α largely in pericytes alleviated muscularization and remodeling of precapillary arterioles; (3) lineage tracing of the reporter color labeled HIF2α overexpressing NG2 cells gained a SMC-like phenotype during the disease progression; (4) the inhibition of HIF2α downstream signaling pathways using AMD3100 protected mice from Hx-induced PH. Taken together, our study demonstrated a better understanding of the molecular mechanism of HIF2α in pericyte-associated vascular remodeling.

## Results

### Pericytes have upregulated expression of HIF2α in human PAH clinical samples

**Figure 1:**
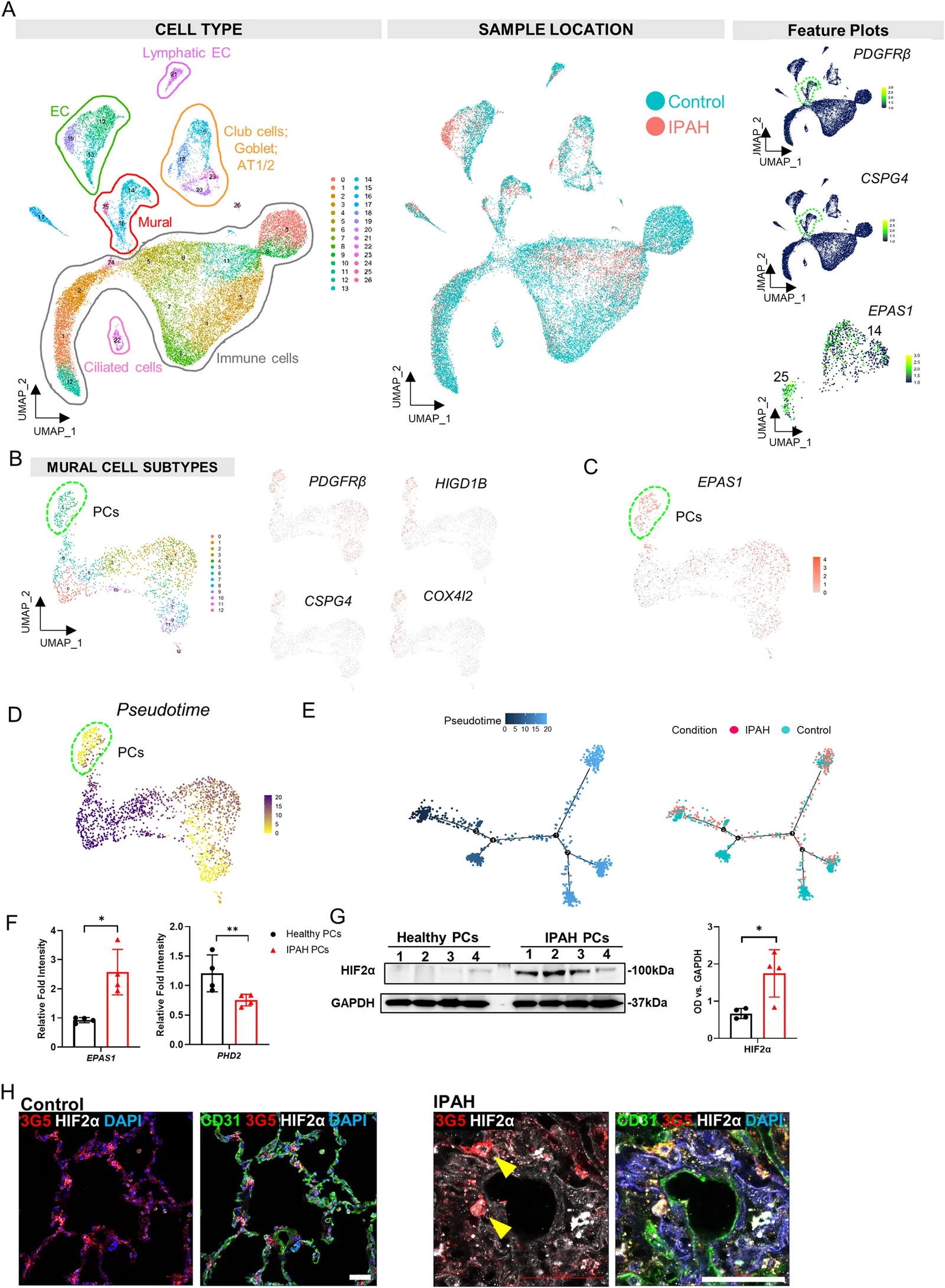
HIF2α is upregulated in human IPAH lung pericytes and PAH lung samples. (**A**) 27 distinct cell clusters were generated using a Uniform Manifold Approximation and Projection (UMAP) plot from both human control and IPAH lungs by single-cell RNA-sequencing analysis. Each color represents a different cell cluster and sample. Expression profiles of *CSPG4* and *PDGFRβ* in all generated clusters and their expression were identified in annotated mural cell clusters #14/16/25. Expression profile of *EPAS1* was showed in zoom-in clusters. (**B**) Mural cell subtypes were re-clustered from cluster 14/16/25. Sub-cluster 5 was enriched with expression profiles of *PDGFRβ*, *CSPG4, HIGD1B* and *COX4I2* and designated as pericyte cluster. (**C**) *EPAS1* was highly expression in sub-cluster 5 by the green dash line. (**D**, **E**) Pseudotime plot shows sub-cluster 5 has immature lineage and additional pseudotime plots of cluster 14/16/25 show control and IPAH mural cell trajectories. (**F**, **G**) (**F**) mRNA levels of *EPAS1*, *PHD2* by qPCR, and (**G**) the protein levels of HIF2α by western blot in control (*n* = 4) and IPAH patient lung pericytes (*n* = 4) were measured. All experiments were repeated at least three biological replicates. Means ± SEM was derived from at least three biological replicates. *depicts a statistically significant difference: *P < 0.05, and **P < 0.01 (paired *t* test with parametric test). (**H**) Immunofluorescence staining on control (*n* = 5) and IPAH patient lung tissues (*n* = 5) using HIF2α (white), 3G5 (red), CD31 (green), and nuclei were stained by DAPI (blue). Arrows indicate the colocalization of 3G5 with HIF2α. Scale bar = 50 µm. Data information: means ± SEM was derived from at least six biological replicates. At least four different fields from each condition were counted. *Depicts a statistically significant difference: *P < 0.05 and **P < 0.01 (one-way ANOVA with Turkey’s multiple comparisons test). Source data are available online for this figure.

**Figure EV1:**
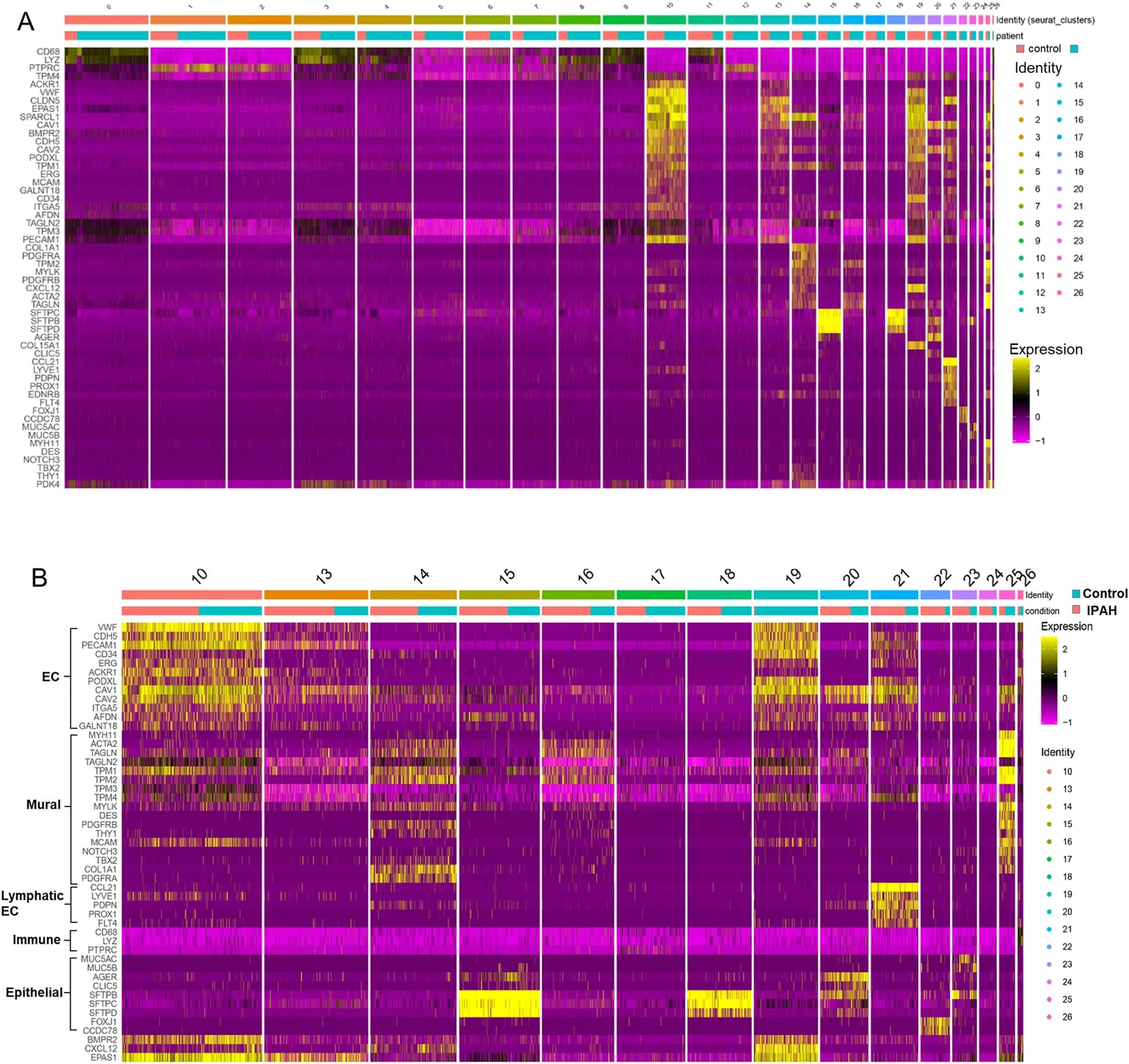
27 clusters are identified using human IPAH lung single-cell RNAseq. (**A**) 27 distinct cell clusters were generated by Uniform Manifold Approximation and Projection (UMAP) plot from both human control and IPAH lungs by single-cell RNA-sequencing analysis. (**B**) The heatmap of expressed genes in clusters 10 and 13–26 was determined by the top gene expressions.

Investigating the transcriptomic alternations of human lung vascular cells has provided us with a much deeper understanding of the drivers of pathogenesis. We utilized and re-analyzed previously published data sets from three idiopathic PAH (IPAH) and six control lungs using droplet-based single-cell RNA sequencing (Saygin et al, 2020; Data ref: Saygin et al, 2020). 27 cell clusters from over 30,000 individual cells were identified. Each cluster contained cells from both control and IPAH lungs and was grouped based on the presence of published markers (Fig. 1A; the heatmap with all clusters is in Fig. EV1A,B). For example, the endothelial cell clusters (#10, 13, 19) were identified by significant and distinct expression of *vWF, CDH5, PECAM1, CD34, ERG, ACKR1, PODXL, CAV2, ITGA5, AFDN*; mural cell clusters (#14, 16, 25) were determined using *MYH11, ACTA2, TAGLN, TAGLN2, TPM1/2/3/4*; lymphatic EC cluster (#21) was identified using *LYVE1, PDPN, PROX1* and *FLT4*; immune cell clusters (#0-9, 11, 12, 24) were identified using *CD68, LYZ, PTPRC*. Clusters # 15, 17, 18, 20, 22, 23 had epithelial lineage cells, such as club cells (*CCSP*), goblet cells (*MUC5A/B/C*), type 1/2 pneumocytes (*AGER, CLIC5, PDPN, FTPB/C/D*), and ciliated cells (*FOXJ1, CCDC78*).

Among the three mural cell clusters (#14, 16, 25), we sought to select any cells expressing either *CSPG4* or *PDGFRβ* (Fig. 1A). A total of 379 single positive cells (CSPG4+ or PDGFRβ + ) were identified in all clusters. Clusters #14, 16, 25 were further sub-clustered into 13 clusters based on their signature genes (Fig. 1C). Sub-cluster 5 was featured by *CSPG4/PDGFRβ/CSPG4/COX4I2 thus* was designated as a pericyte cluster where *EPAS1* was highly expressed in 76% of cells (Fig. 1B,C). Filtering over 33,000 genes (fold change >1.5, absolute gene expression >0.3), 569 genes were yielded as significantly highly expressed in IPAH pericytes compared to the control. Among them, the top 20 most differentially expressed genes (DEG) included *THY1*, a signature gene of mature SMCs (Appendix Table S1). We then aligned mural cells in Pseudotime for sub-cluster 5 and pericyte-enriched sub-cluster 5 (in yellow) showed an immature lineage status (Fig. 1D). We further modeled disease progression trajectories for cluster 14/16/25 (Fig. 1E). In addition, most IPAH mural cells (orange dots) localized to their corresponding cell states and clustered into later time points (the lightest blue clusters contained more orange dots) when compared to the distributed mural cells at early time points in control samples. Together, these data suggested that IPAH mural cells were more actively undergoing a differentiation process than controls, thus cell fate and phenotype were prone to be SMC-like.

**Figure EV2:**
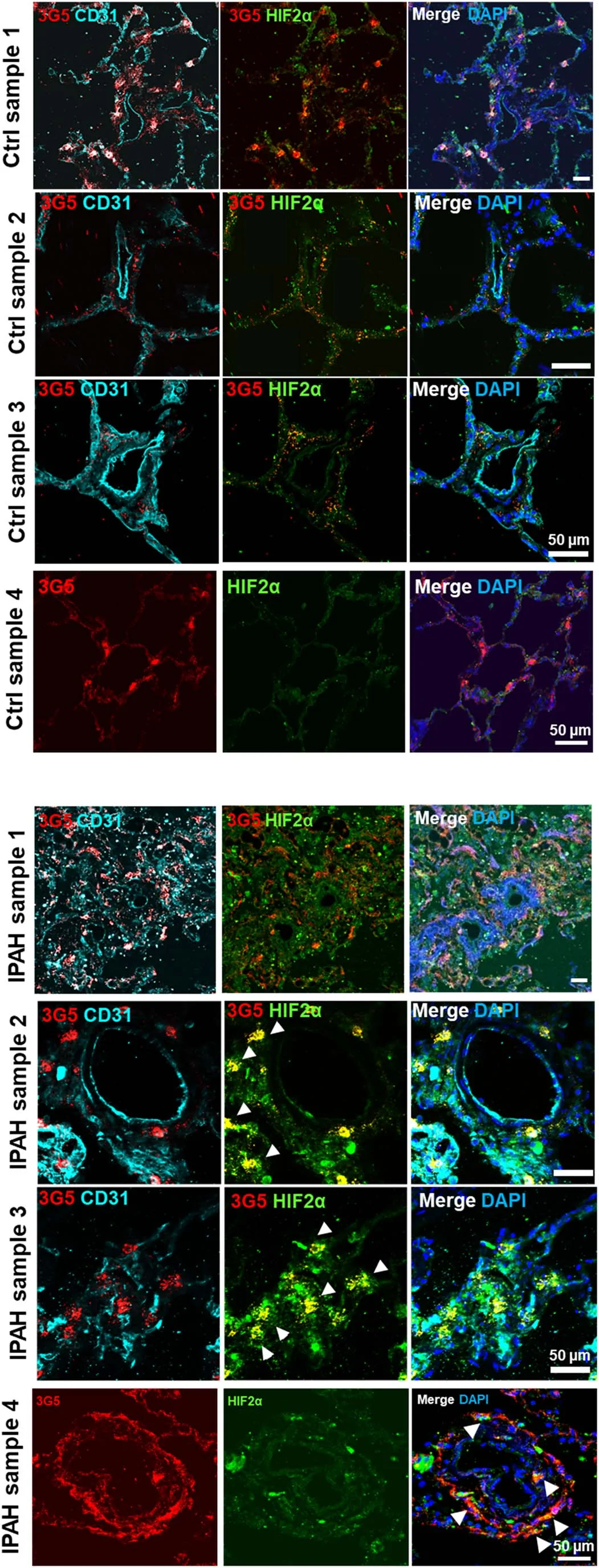
Four more control and Four more IPAH patient lung tissues were immunofluorescence stained using HIF2α (Green), 3G5 (Red), CD31 (Cyan), and nuclei were stained by DAPI (blue). 3G5 colocalized by HIF2α was indicated by arrows. The higher magnification images are on the bottom two rows. Scale bar =  50 µm.

Our prior study identified CXCL12 (aka SDF1) as a candidate gene that could orchestrate mural cell behavior in the lung. SDF1 is a transcriptional target of HIF1α (hypoxia-inducible factor 1α), a member of the HIF signaling that is stabilized on exposure to hypoxia (Ceradini et al, 2004). HIF2α (gene name *EPAS1*) has been studied widely in the CXCL12 pathway in ECs or SMCs, but its function in pericytes is under investigation. To first confirm the results of scRNA-seq, we conducted qPCR for *EPAS1* and its related genes including *VHL* and *PHDs*, and protein expression of HIF2α on lung pericytes isolated from four healthy donors and four IPAH patients who underwent a lung transplant (Appendix Table S2). Interestingly, IPAH pericytes exhibited higher levels of *EPAS1*/HIF2α and this upregulation was mainly altered by *PHD2* not other genes (Fig. 1F–G; Appendix Fig. S1). Lastly, IPAH patient lung tissues demonstrated an overall increased expression of HIF2α in lesions by IF staining (Figs. 1H and EV2). When counterstained with pericyte marker 3G5 ganglioside, 60% 3G5 positive cells also co-expressed HIF2α in IPAH samples, whereas 5% in controls (Fig. 1H, arrows). Based on these data, we speculated that HIF2α activation may mediate PC lineage change.

### Human pulmonary pericytes overexpressing HIF2α transform into SMC-like cells

**Figure 2:**
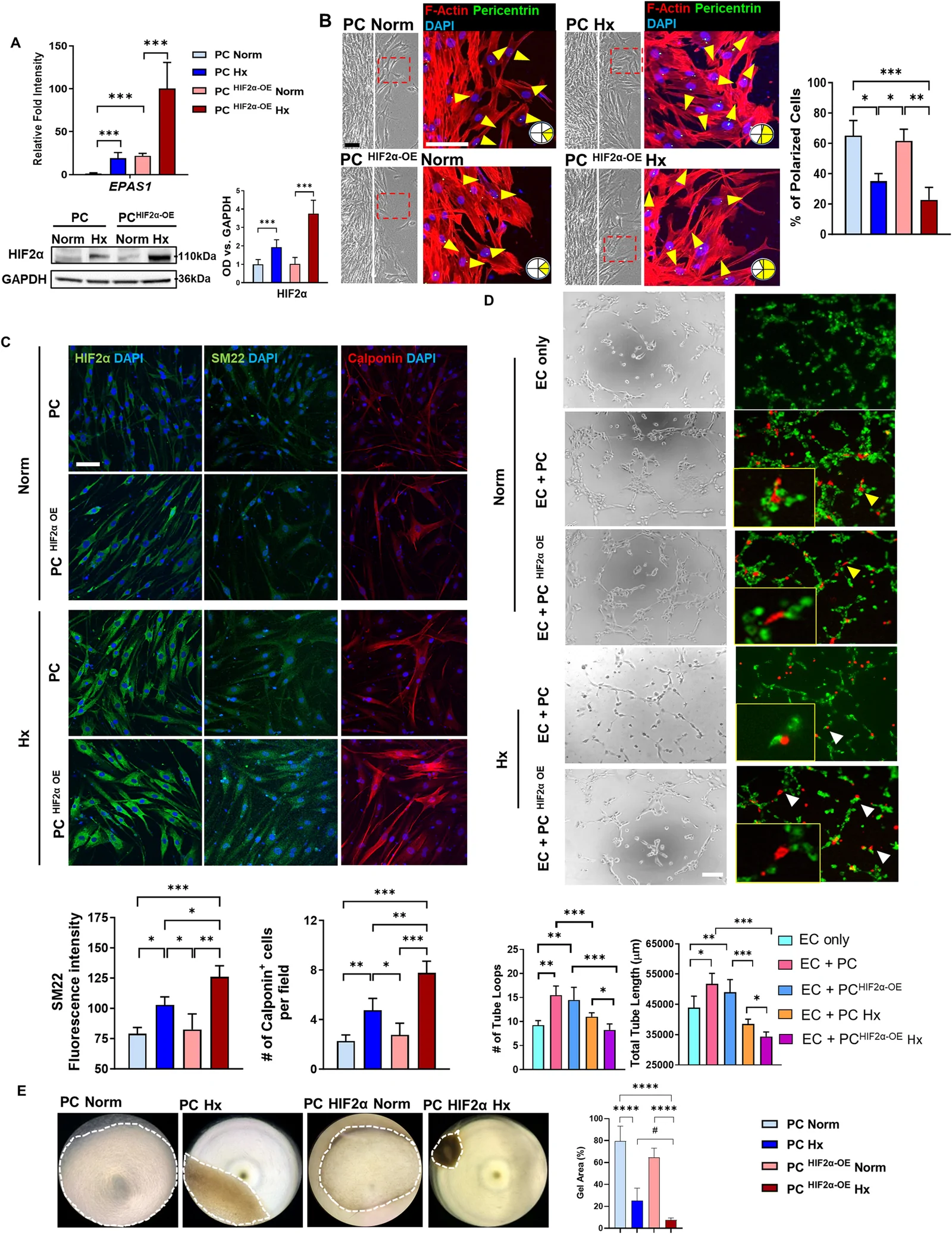
Human lung pericytes overexpressing HIF2α transform into SMC-like cells. (**A**) The mRNA expression levels of *EPAS1* and the protein expression levels of HIF2α in human pericytes with/without HIF2α overexpression (OE) under hypoxia or normoxia were measured using qPCR and western blot (WB). (**B**) Representative images show the polarity of PC (top) and PC HIF2α OE (bottom) by the wound-healing assay. The white lines indicate 0 hr. Red dash boxes were enlarged and shown on the right. Cell polarity was assessed by Pericentrin (the yellow section in the circle at the bottom right of each panel indicates the area of polarized cell direction). The arrowheads indicate directions of cell movement by the alignment of MTOC and the cell nucleus. The number of polarized cells was quantified in the graph on the right. The color legends were the same as A. Scale bar = 50 µm. (**C**) Immunofluorescence (IF) images of normoxia (top) and hypoxia (bottom) of PC and PC HIF2α OE stained by HIF2α (left, green), SM22 (middle, green), and Calponin (right, red). Nuclei were stained with DAPI (blue). The fluorescence intensity of SM22 and the number of calponin-expressing cells over the total number of DAPI-positive cells were quantified and compared between groups. Scale bar = 50 µm. (**D**) Pericytes with/without HIF2α overexpression were cocultured with ECs on Matrigel. Yellow arrows indicate PC overage on EC tubes. White arrows indicate reduced cell–cell interaction. Scale bar = 100 µm. The number of total loops and total tube length were quantified below. (**E**) The representative images show the sizes of the collagen gels of PC or PC HIF2α OE with or without Hx. The dashed lines represent the size of the collagen gel after 48 h. The gel coverage area was quantified by ImageJ. Data information: Means ± SEM was derived from at least six biological replicates. At least six different fields from each condition were counted. *, # Depicts a statistically significant difference: *P < 0.05, **P < 0.01, ***P < 0.001, and ****P < 0.0001 (one-way ANOVA with Turkey’s multiple comparisons test). ^#^*P* < 0.05 (unpaired *t* test). Source data are available online for this figure.

To evaluate the effects of HIF2α upregulation in lung pericytes, we transfected control pericytes with a plasmid containing the *EPAS1* gene (PC^HIF2α-OE^) followed by a 24 h stabilization using a hypoxic incubator (FiO_2_: 1%). First, we divided pericytes into four groups: control pericytes cultured in normoxia or hypoxia, and HIF2α transfected pericytes cultured in normoxic PC^HIF2α-OE^ or hypoxic PC^HIF2α-OE^ to compare the expression of HIF2α at the molecular level. The mRNA and protein expression level of HIF2α in hypoxic groups was the highest among the others (Fig. 2A; Appendix Fig S2).

We next measured the cellular polarity using a wound-healing assay and immunostaining of F-actin and Pericentrin, a protein located within the microtubule-organizing center (MTOC; Fig. 2B). MTOC is localized in front of the nucleus, and its alignment with the cell’s long axis indicates the direction of cellular movement (arrowheads, see M&M for quantification). The yellow color in the pie plots indicated the overall directions of movement. After 10 h, MTOCs of normoxic PC and PC^HIF2α OE^ were located close to the wound edge, pointing toward the gap and narrowing the gap. In contrast, hypoxic PC and PC^HIF2α OE^ demonstrated wider and random directions, suggesting abnormal polarity. Hypoxic PC^HIF2α-OE^ also showed increased expressions of HIF2α and SMC markers Calponin and SM22 by IF after 7 days Hx (Fig. 2C). Furthermore, we tested PC^HIF2α-OE^ interaction with endothelial cells and measured angiogenesis on Matrigel. Tube formation and total tube length were increased when PCs or PC^HIF2α^ (PKH26 red-stained) cocultured with EC (PKH67 green-stained) under normoxia. However, hypoxic co-culture of ECs and PCs had reduced tube formation, and hypoxic EC with PC^HIF2α-OE^ further resulted in reduced pericyte coverage and the total tube length/loops (Fig. 2D, white arrow). Lastly, we measured the contractility, which indicated the mechanical tension of mature mesenchymal cells, using collagen gel assay (Yuan et al, 2020). Hypoxic PC^HIF2α-OE^ demonstrated significantly increased contractility by sixfold decreased gel size than other conditions, suggesting the gain of a SMC-like phenotype (Fig. 2E). However, the potential lineage change was not associated with cell morphology or proliferation (Appendix Figs. S3 and 4).

### Murine pericytes show increased expression levels of HIF2α after hypoxia-induced PH lungs

**Figure 3:**
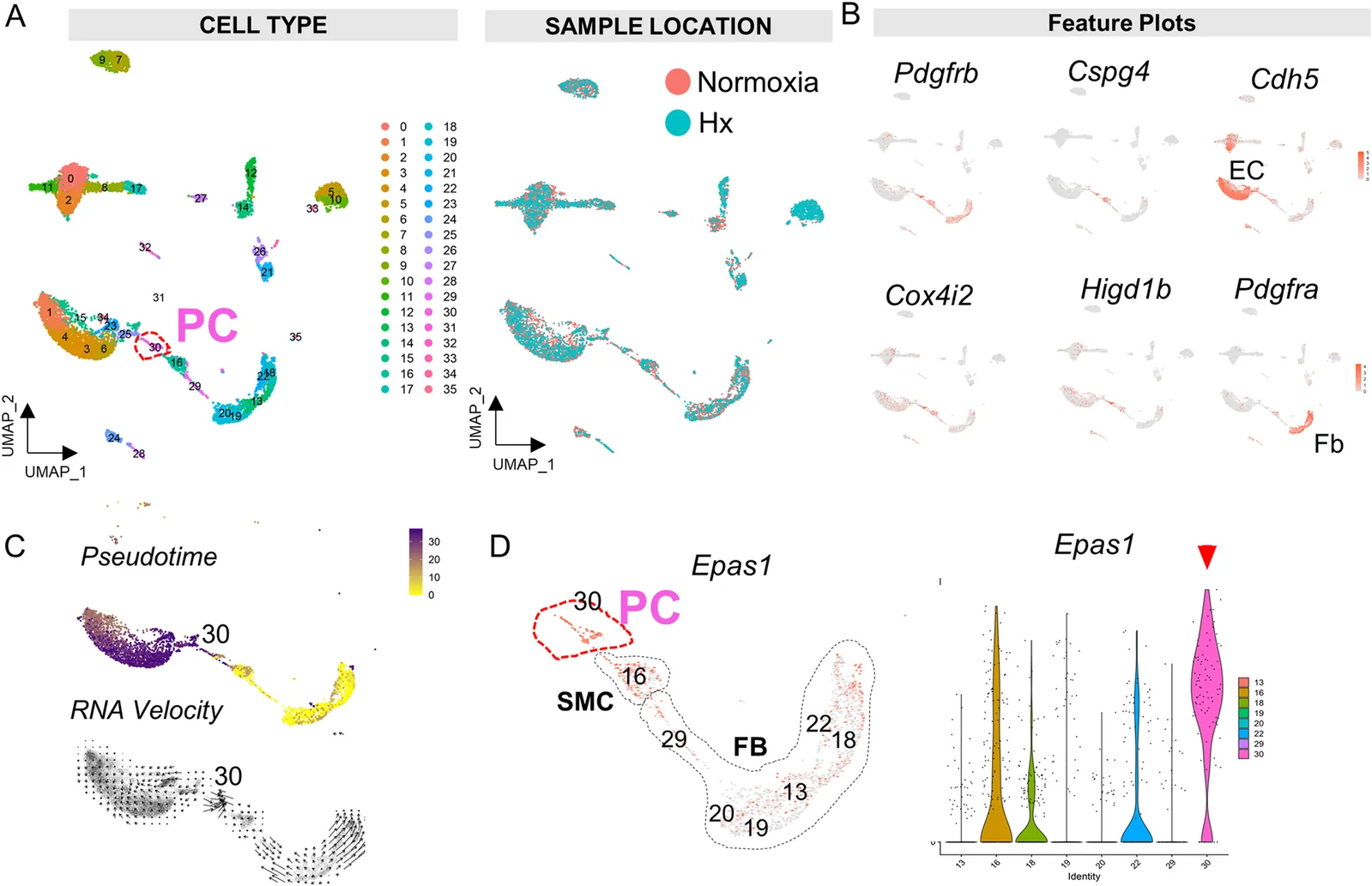
Murine pericytes show increased expression levels of *Epas1* after hypoxia-induced PH lungs. (**A**) 36 distinct cell clusters were generated using UMAP plots from normoxia vs hypoxia lungs by scRNA-seq. (**B**) The UMAP plot of whole lung cells underwent hypoxia vs normoxia, and cluster 30 was identified as pericyte cluster. The feature gene plots to distinguish different mural cell population including pericytes/SMCs/fibroblasts. The Feature plots identified clusters of PCs (*Pdgfrb, Cspg4, Cox4i2 &Higd1b*), ECs(*Cdh5*) & fibroblasts(*Pdgfra*). (**B**) Clusters of ECs and mural cells as mesenchymal clusters from (**A**) were enlarged. (**C**) Pseudotime analysis shows PCs (cluster 30) have a mixture of early (yellow) and late lineages (purple). RNA Velocity shows PCs are prone to have active lineage change (long arrows). (**D**) The gene expression level of *Epas1* is shown in different mural clusters. Violin plot shows the gene expression level of *Epas1* in the defined clusters and is the highest in cluster 30 (red arrow).

To further test our hypothesis on pericyte transformation, we performed 10× single-cell transcriptomic analysis on whole lung cell populations from three WT animals exposed to 3-week Hx. Single-cell suspension was achieved by enzymatically dissociation and depletion of CD45 + CD326+ cells after Miltenyi AUTOMACS sorting. To better resolve differences between heterogeneity of cells using unsupervised graph clustering partitioned cells into groups, we combined a published data set of hypoxic and normxic lung cells with ours (Thomas et al, 2022; Data ref: Lilly et al, 2022) and visualized all of them using uniform manifold approximation and projection (UMAP; Fig. 3A). Cells were sequenced at a high sequencing depth used for all subsequent analyses, and a total of 13,343 genes with a significant *P* value per cell were obtained. We also eliminated cells that expressed gene counts >4000 as doublets or <200 as cell debris before analysis. Using expression data for more than 2500 genes per cell, we identified different clusters of cell-type-specific markers. The initial set of markers was widely expressed in one cell type, not in any other clusters, as their signature genes and 36 clusters were identified (Fig. 3B). Markers used to define pericytes as cluster 30 were based on *Pdgfrb,Cspg4, Cox4i2, Higd1b*; SMCs as cluaters 16 and 29 were based on *Acta2, Myh11, Tagln, Mylk, Mcam*,; ECs as clusters 1, 3, 4, 6, 15, 23, 25, and 34 were based on *Cdh5, Pecam1, vWf*, *Flt1*; the fibroblast markers as cluater 13, 18, 19, 20, 22 were based on *S100a4, Col3a1*, and *Pdgfra* (Baek et al, 2022; Lendahl et al, 2022; Vanlandewijck et al, 2018). Based on the above method, distinct mesenchymal clusters were identified, and each cluster containing a percentage of cells from both normoxia and 3-week hypoxia. We extracted all mesenchymal cells and performed lineage trajectory analysis. Intriguingly, Pseudotime showed PC (cluster #30) had a mixture of early and late lineage stages and RNA velocity showed high PC differentiation rate by long arrow sizes. Both analyses consistently suggested that PCs were undergoing an active differentiation process (Fig. 3C). Notably, *Epas1* was significant in 97% of cells of cluster 30 and further demonstrated by Volin plot (Fig. 3D, red arrow). Noticeably, the *Hif1a* expression level was not significantly altered in any clusters. Above all, the single-cell RNAseq revealed that the upregulated expression of *Epas1* may play a vital role in pericyte lineage change.

### HIF2α overexpression in pericyte-dominant transgenic mice develop severe vascular remodeling and muscularization

**Figure 4:**
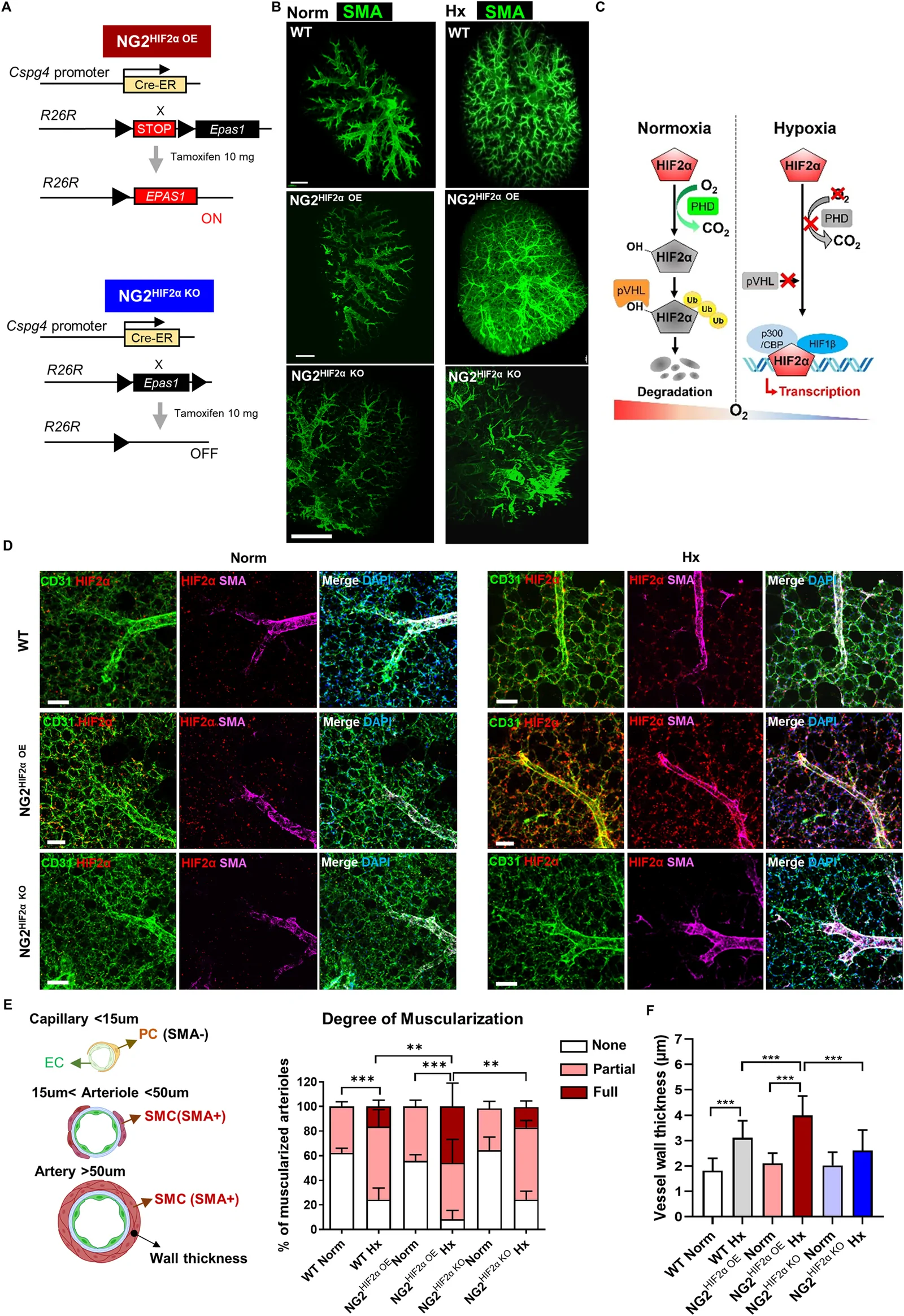
HIF2α overexpression in PC-dominant transgenic mice develop severe vascular remodeling and muscularization. (**A**) Diagrams show the construction design of the transgenic NG2^HIF2α OE^ and NG2^HIF2α KO^ mice. (**B**) Representative light-sheet microscopic images from iDISCO-cleared lung lobes, showing 3D views of SMA-labeled lobes of WT, NG2^HIF2α OE^, and NG2^HIF2α KO^ mice exposed to normoxia vs hypoxia. Scale bar = 1 mm. SMA indicates the mural cell coverage elongated the distal vessels after Hx. All experiments were repeated at least four biological replicates. (**C**) Schematic overview of the HIF2α signaling pathway in normoxia vs hypoxia. (**D**) Precision-cut lung slices of WT, NG2^HIF2α OE^, and NG2^HIF2α KO^ mice with normoxia vs hypoxia were stained for CD31 (green), HIF2α (red), and SMA (magenta). Nuclei were stained with DAPI (blue). Scale bar = 50 µm. All experiments were repeated at least six biological replicates. (**E**) Schematic images represent a visual definition of none, partially, and full muscularization in the cross-section of vessels. The graph represents the quantification of the percentage of muscularized vessels over the total number of vessels in each group. Each condition contained 120 vessels (diameter = 50 µm) from ten mice. Means ± SEM was derived from at least three biological replicates. (**F**) The graph shows the measurements of the vessel wall thickness of each group using H&E staining (see Suppl Fig. 9). Each group contained 120 vessels (30 µm < diameter <100 µm) from six mice. Each vessel was measured in four different thickness areas (by measuring the outer wall boundary minus the inner wall boundary) to calculate the average thickness. Data information: Means ± SEM was derived from at least three biological replicates. *Depicts a statistically significant difference: **P < 0.01 and ***P < 0.001 (one-way ANOVA with Turkey’s multiple comparisons test). Source data are available online for this figure.

To assess the biological contribution of HIF2α in PC to Hx-induced vascular remodeling, we generated conditional transgenic murine lines by crossing NG2-Cre-ER :: Rosa26-Stop^*flox/flox*^-*Epas1* (i.e., NG2^HIF2α OE^) or with Rosa26-*Epas1*^*flox/flox*^ (i.e., NG2^HIF2α KO^) (Fig. 4A). A 10 mg dose of tamoxifen via intraperitoneal injection was sufficient to selectively overexpress or decrease HIF2α in NG2 promoter-driven cells, selectively on PCs. After tamoxifen administration, mice were rested for at least 14 days before being placed in a Hx chamber (FiO_2_ 10%) for up to 3 weeks. The expression levels of HIF2α were validated on whole lung tissue lysates, and the hypoxic NG2^HIF2α OE^ sample showed a twofold increased mRNA level of *Epas1* and a fivefold increased protein level of HIF2α using qPCR and western blot (WB) (Appendix Fig. S5A,B). The deletion efficiency of the *Epas1* gene in NG2^HIF2α KO^ was also determined by qPCR and WB. The mRNA and protein levels of *EPAS1* in hypoxic NG2^HIF2α KO^ were not different than those in the normoxia or WT littermate groups (Appendix Fig. S5C).

Increased SMA staining was found in distal microvessels (<30 µm in diameter) of hypoxic NG2^HIF2α OE^ compared to controls, indicating increased smooth muscle coverage and muscularization (but not vessel pruning or angiogenesis) using whole lobe deep tissue clearing iDISCO and imaging by light-sheet microscopy **(**Fig. 4B; Movies EV1 and 2). In contrast, no change in SMA expression and coverage in NG2^HIF2α KO^ whole lungs was found compared to controls, indicating that reduction of HIF2α ameliorated remodeling and muscularization (Fig. 4B). Although NG2^HIF2α OE^ normoxic lungs expressed higher levels of HIF2α mRNA and protein than the wild-type littermate (WT) group, their vasculature did not show a distinctive remodeling, which was likely associated with HIF2α ubiquitous degradation pathway described in Fig. 4C. Briefly, HIF2α subunits undergo rapid degradation under normal oxygen levels in room air (O_2_ 21%) (Semenza, 2012) whereas, in low oxygen levels (O_2_ 1–10%), stabilization of HIF2α subunits can be achieved by the inhibition of PHD-dependent proline hydroxylation (Fig. 4C). Therefore, the goal to overexpress HIF2α in NG2 cells was achieved via exposure to Hx.

Looking in-depth at individual arteries using confocal microscopy, muscularization of small arterioles with a diameter of 15–50 µm was found in hypoxic NG2^HIF2α OE^ (Fig. 4D). NG2-positive cells had a significantly higher level of HIF2α in hypoxia whereas the HIF2α was reduced due to degradation in normoxia (Fig. 4D, the middle row). Immunostaining revealed that HIF2α cells (red) were SMA- (magenta) and localized within the alveolar-capillary space in normoxia. In contrast, those cells were SMA + after 3-week Hx and covered the muscularized arterioles (~ 20 µm). Staining of mature SMC marker SMMHC indicated that the distal arterioles in normoxia were not muscularized, which were SMMHC- (red) SMA- (green), yet in Hx, distal arterioles highly expressed SMMHC and SMA signals representing vigorous muscularization and vascular remodeling (Appendix Fig. S6, the middle row, the color became yellow if colocalized with green, arrowheads). The reduced level of HIF2α was further validated in NG2^HIF2α KO^ under normoxia or 3-week Hx (Fig. 4D, the bottom row). Unlike NG2^HIF2α OE^, distal arterioles (15 <diameter <50 µm) of NG2^HIF2α KO^ were SMA-, similar to WT normoxic lungs. In addition, a majority of SMMHC + SMA+ cells NG2^HIF2α KO^ showed no difference to WT-Hx (Appendix Fig. S6, the bottom row).

**Figure 5:**
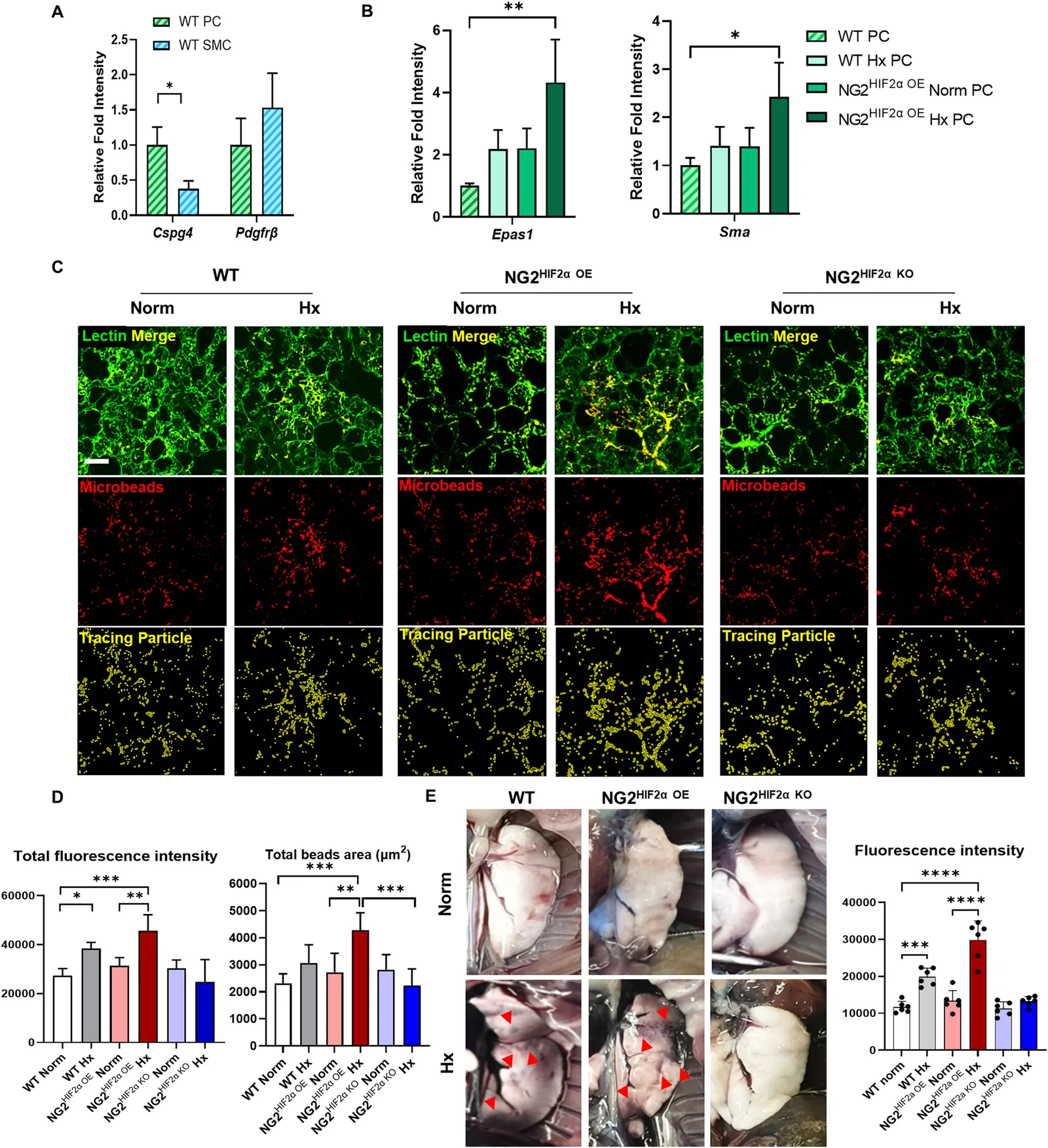
HIF2α overexpression in PC-dominant transgenic mice develop vascular leakage. (**A**) mRNA levels of *Cspg4* and *Pdgfrβ* were measured in CD140b+ (PC-dominant) and CD146+ (SMC-dominant) cells isolated from WT mice. (**B**) mRNA expression levels of *Epas1* and *Sma* were measured in CD140b+ (PC-dominant) cells isolated from WT and NG2^HIF2αOE^ mice under normoxia or hypoxia. (**C**, **D**) Vascular integrity was evaluated in each group using lectin/microsphere bead injection. The degree of vascular leakage was evaluated by the excessive red fluorescence-labeled microsphere beads that remained in the endothelium after flushing. The red fluorescent intensity was converted into fluorescent particles and the total area was quantified using Aivia. Scale bar = 50 µm. (**E**) Representative images of vascular permeability from WT, NG2^HIF2α OE^, and NG2^HIF2α KO^ mice using Evans blue under normoxia or hypoxia. The blue dye leaked through the interstitial space and remained as blue patches after flushing (red arrows). Data information: Means ± SEM was derived from at least six biological replicates. *Depicts a statistically significant difference: *P < 0.05, **P < 0.01, and ***P < 0.001. Paired *t* test with a parametric test for (**A**) and one-way ANOVA with Turkey’s multiple comparisons test for the rest. Source data are available online for this figure.

The definition of mural cell coverage used in this study was summarized in Fig. 4E. Under normal conditions, pericytes resided in capillaries (diameter <15 µm) were SMA-. Arterioles were defined as diameters ranging 15–50 µm and partially covered with SMA+ SMCs. Arteries were defined as diameters larger than 50 µm where the SMA + SMC coverage was full (100%). The degree of muscularization was calculated by the ratio of the area contacting the circumference of SMA+ SMCs with the EC layer over the circumference of the EC layer. The wall thickness was the subtraction from the diameter of the outer to the inner layer (Fig. 4F). Arterioles were usually the transiting zone from non-muscular to muscular, therefore, we evaluated the degree of muscularization using arterioles in each condition. 45% of arterioles in hypoxic NG2^HIF2α OE^ had partial SMA coverage and 45% of them were fully covered, whereas normoxic NG2^HIF2α OE^ had 37% partial and only 1% full coverage. More muscular coverage was associated with thicker walls. Hypoxic NG2^HIF2α OE^ had the highest wall thickness compared to the other conditions when considering vessel diameter >30 µm using H&E staining (Appendix Fig. S7). To validate immunostaining results, we sorted PCs based on one of its surface markers Cd140b (aka Pdgfrb), and SMCs based on Cd146 (aka Mcam), using combined methods of Miltenyi AutoMACS and Dynabeads. Cd140b cells showed more *Cspg4* expression than Cd146 cells but no difference in the expression level of *Pdgfrb*, suggesting the Cd140b-positive cells were predominantly pericytes (Fig. 5A). Cd140b-sorted cells from hypoxic NG2^HIF2α OE^ showed the highest expression levels of *Epas1* and smooth muscle marker *Sma* whereas Cd146 sorted cells did not have any change in expression levels of *Epas1* or *Sma* (Fig. 5B; Appendix Fig. S8), suggesting NG2 cells overexpressing HIF2α transform to be more SMC-like cells. However, the excessive muscularization was not associated with fibrosis using Trichome staining in all conditions (Appendix Fig. S9).

### HIF2α overexpression in PC-selective transgenic mice develop vascular leakage

Abnormal vascular remodeling can be associated with the loss of endothelial barrier function, we thus evaluated vascular integrity and permeability by FITC-lectin/Rhodamine-microspheres (50 nm in diameter) via i.v. injection. After carefully flushing with PBS to eliminate circulating lectin and microspheres, the pulmonary vasculature and its integrity were displayed by FITC-lectin and microspheres, respectively. If barrier dysfunction existed, microspheres tended to remain in the loose junctions of the endothelium. Compared to its normoxic control, a discontinuous FITC-lectin staining and a substantially increased amount of red fluorescence microspheres remained in the vasculature of hypoxic NG2^HIF2α OE^ lungs, suggesting capillary integrity was disrupted. However, hypoxic NG2^HIF2α KO^ showed no difference of leakiness compared with its controls when the fluorescence intensity was converted to tracing particles, indicating that HIF2α reduction protected from endothelial barrier dysfunction (Fig. 5C,D; Appendix Fig. S10A). These results were further validated using Evans Blue assay. The normoxic and hypoxic NG2^HIF2α KO^ did not have any dye residue, whereas the hypoxic NG2^HIF2α OE^ demonstrated more areas of blue dye leakage and significantly higher fluorescence intensity than other conditions, suggesting the disrupted permeability and homeostasis of the endothelium (Fig. 5E, red arrows).

### HIF2α overexpression in pericyte-selective transgenic mice exacerbates PH and RVH

**Figure 6:**
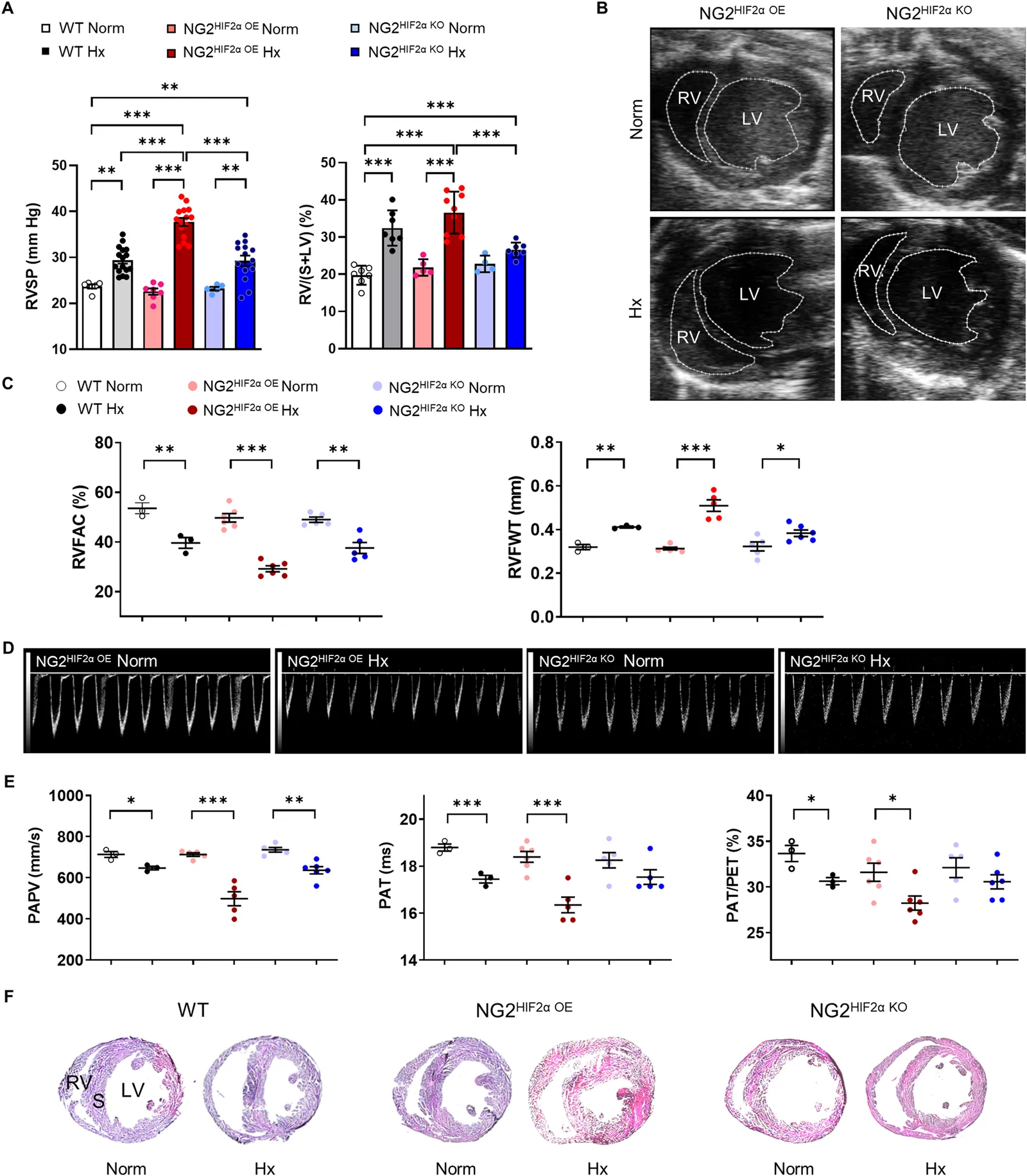
HIF2α overexpression in pericyte-dominant transgenic mice exacerbates PH and RVH. (**A**) RVSP and Fulton Index were measured on NG2^HIF2α OE^ and NG2^HIF2α KO^ mice with or without Hx. (**B**) Parasternal short-axis views at the mid-papillary level of the left ventricle in the diastole phase demonstrated an enlarged RV chamber in NG2^HIF2α OE^ after 3-week hypoxia, but no RV size difference in NG2^HIF2α KO^ mice. (**C**) RVFAC and RVFWT were measured by echocardiogram in NG2^HIF2α OE^ and NG2^HIF2α KO^ mice with or without Hx. (**D**, **E**) Pulmonary outflow PW Doppler tracings were used for quantification in PW Doppler tracings, PA peak velocity, PAT, and PAT/PET ratios, in NG2^HIF2α OE^ and NG2^HIF2α KO^ mice with or without Hx. (**F**) Representative images of sectioned hearts show the RV wall thickness from each group. Data information: means ± SEM was derived from at least four biological replicates. *Depicts a statistically significant difference: *P < 0.05, **P < 0.01, and ***P < 0.001 (one-way ANOVA with Turkey’s multiple comparisons test). Source data are available online for this figure.

**Figure EV3:**
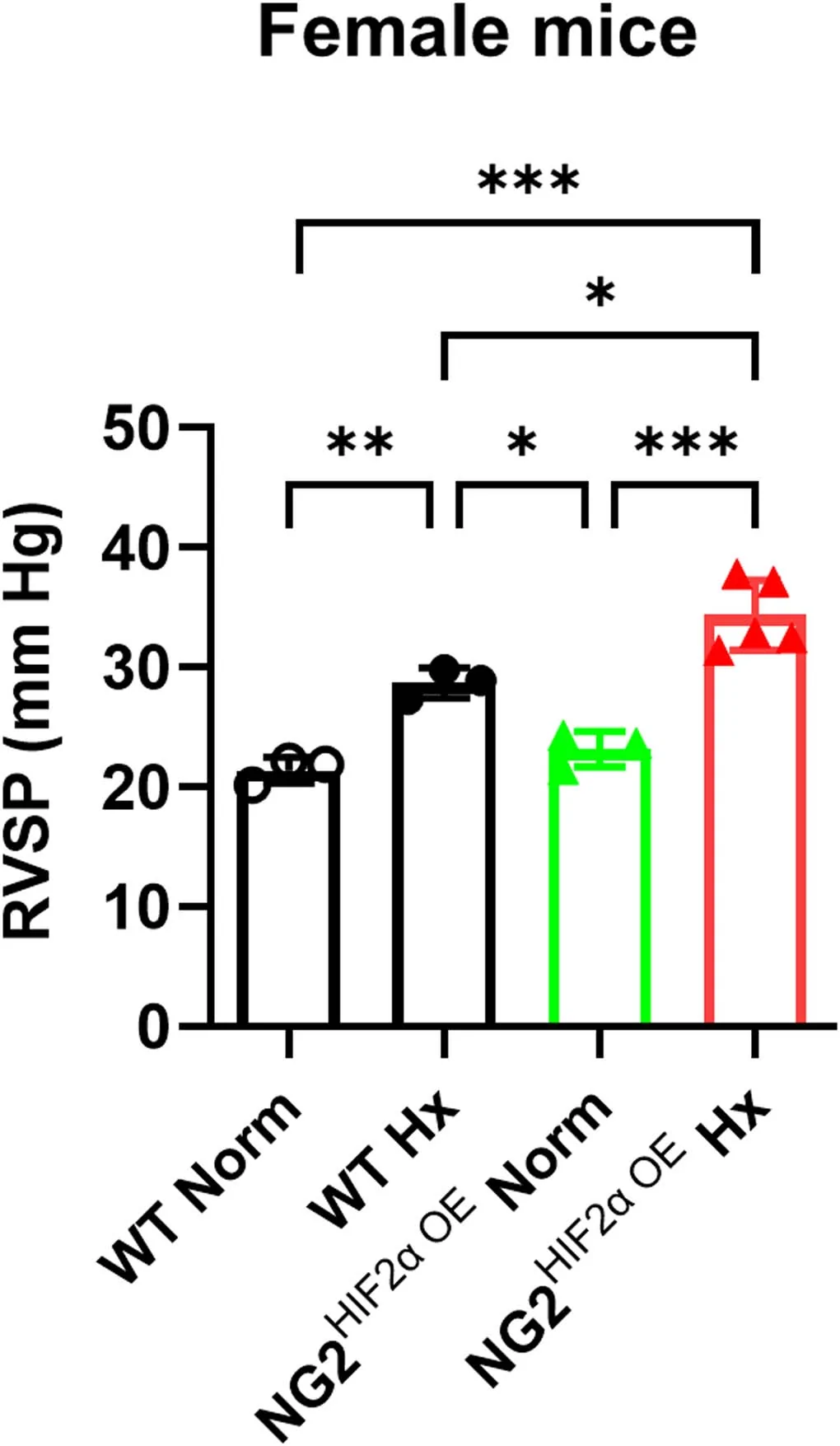
RVSP measurements were performed on WT and NG2^HIF2α OE^ female mice with or without 3-wk Hx. *Depicts a statistically significant difference: *P < 0.05, **P < 0.01, and ***P < 0.001 (one-way ANOVA with Turkey’s multiple comparisons test).

To determine whether these transgenic mice developed Hx-induced PH, we measured the right ventricle systolic pressure (RVSP) by catheterization, which indicates the pulmonary artery (PA) systolic pressure, as well as Fulton index, a weight ratio of RV to left ventricle plus septum (RV/LV + S; Fig. 6A). After 3 week Hx, NG2^HIF2α OE^ mice developed significantly elevated RVSP (37.07 mmHg compared to its normoxic control of 22.32 mmHg). Along with elevated RVSP, NG2^HIF2α OE^ mice developed significantly more RVH as indicated by Fulton index (average of 36.5%) and RV dysfunction by echocardiography assessments (Fig. 6A). Moreover, female hypoxic NG2^HIF2α OE^ mice also exhibited RVSP up to an average of 34.37 mmHg, which was significantly higher than other groups, indicating no effect of gender differences (Fig. EV3). In contrast, hypoxic NG2^HIF2α KO^ still had a significantly high RVSP but did not develop RVH, although five out of fourteen had RVSP < 27 mmHg. The sustained PH was due to hypoxic pulmonary vasoconstriction, which was HIF-independent (Fig. 6A). Given that the hypoxic exposure produces changes in RV structure and function in murine models(Poels et al, 2014; Pugliese et al, 2015; Zhu et al, 2019), we performed ultrasound imaging of the RV area and the free wall on both OE and KO mice at the end of 3-week Hx and obtained heart sections for postmortem histology.

First, RV fractional area change (RVFAC) was assessed by comparing the RV area at the systolic and diastolic phases measured by the parasternal short-axis view for the reduction in RV contractility. A significant reduction was observed in each group after hypoxia (WT hypoxia 39.6% vs. normoxia 53.5%; NG2^HIF2α OE^ hypoxia 29.1% vs. normoxia 49.7%; NG2^HIF2α KO^ hypoxia 37.5% vs. normoxia 48.9%). All echo data were analyzed using a single analyzer and over three consecutive cardiac cycles. In addition, RV-free wall thickness (RVFWT) was assessed for further evidence of RVH (Fig. 6B,C; Movies EV3–6). RV wall thickness was also elevated in all hypoxic groups, but highly significantly increased in the OE group (WT hypoxia 0.41 mm vs. normoxia 0.32 mm; NG2^HIF2α OE^ hypoxia 0.50 mm vs. normoxia 0.31 mm; NG2^HIF2α KO^ hypoxia 0.38 mm vs. normoxia 0.32 mm). While structural and functional changes were noted in each model compared to normoxic controls, these changes were more significant in OE mice, while KO mice had normal heart function which was similar values to WT controls. Moreover, under Hx, all groups exhibited a decrease in PA peak velocity (PAPV) and pulmonary acceleration time (PAT) and ejection time (PET) ratio, suggesting a perturbation of right ventricular diastolic dysfunction (Fig. 6D,E). Both PAPV (mm/s) and PAT/PET ratio were decreased in Hx (PAPV: NG2^HIF2α OE^ hypoxia 498.15 ± 76.27 vs. normoxia 713.01 ± 18.80 mm/s, NG2^HIF2α KO^ hypoxia 636.72 ± 43.39 vs. normoxia 736.31 ± 25.13 mm/s; PAT/PET: NG2^HIF2α OE^ hypoxia 28.23 ± 1.89 vs. normoxia 31.60 ± 2.41, NG2^HIF2α KO^ hypoxia 30.56 ± 1.90 vs. normoxia 32.11 ± 2.43). Interestingly, NG2^HIF2α OE^ hypoxic mice showed a more significant decrease in overall heart function measurements. The H&E staining of the sectioned heart showed that RV enlargement and wall thickening were more significant in hypoxic NG2^HIF2α OE^ mice compared to other controls. In contrast, the RV of hypoxic NG2^HIF2α KO^ showed no difference to its control (Fig. 6F). Therefore, the hypoxic NG2^HIF2α KO^ mice had normal RV function and were protected from hypoxia-induced PH and RVH. Lastly, analysis in LV function and mass did not show any differences between WT and NG2^HIF2α OE^ mice under normoxia or hypoxia (Appendix Fig. S11). Taken together, the hemodynamics, echocardiography, and histology were all consistent with a pathological role of HIF2α in pulmonary vascular remodeling. Our results provided insights into a potential key in the transcriptional regulatory function of HIF2α in NG2 cells during the development of Hx-induced PH and its associated vascular remodeling.

### HIF2α overexpression in tdT cells (PC-selective) become SMC-like during vascular remodeling

**Figure 7:**
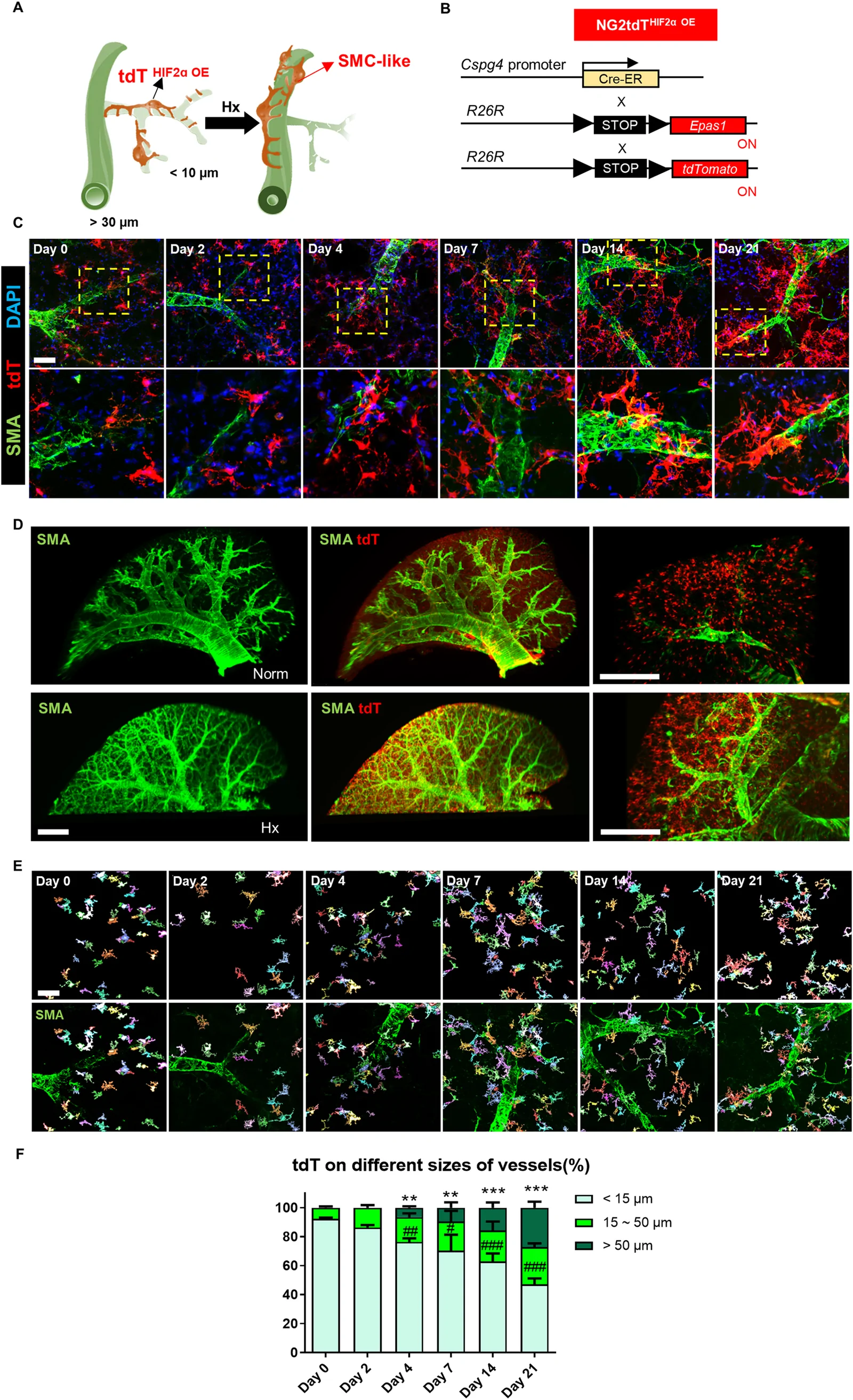
HIF2α overexpression in tdT (PC-dominant) cells become SMC-like during vascular remodeling. (**A**) The schematic figure represents tdT^HIF2α OE^ cells differentiating into SMC-like cells and their translocation/recruitment towards the larger arterioles. (**B**) The diagram shows the construction design of NG2tdT^HIF2α OE^ transgenic mice. (**C**) Precision-cut lung slices of NG2tdT^HIF2α OE^ mice were stained with SMA (green) and DAPI (blue), representing the temporal translocation and SMA expression levels of tdT^HIF2α OE^ cells after exposure to hypoxia on days 0, 2, 4, 7, 12, and 21. Scale bar = 50 µm. (**D**) Representative light-sheet microscopic images from iDISCO-cleared lung lobes showed 3D views of SMA-labeled lobes from NG2tdT^HIF2α OE^ mice exposed to hypoxia. tdT was enhanced using RFP antibodies. The 3D structure of both lobes can be found in the supplemental Movies EV7 and 8. Scale bar = 1 mm. (**E**) Computational conversion of tdT^HIF2α OE^ cells into 3D mesh structure was quantified (top row) using Aivia and images were combined with SMA-stained arterioles (bottom row). Scale bar = 50 µm. (**F**) The total number of meshes on the SMA-stained blood vessel at each time point (from **C**) was measured and the number of tdT-positive cells on the arterioles versus the total number of DAPI-positive cells on different sizes of vessels was quantified. Each group had at least 11 animals. Means ± SEM was derived from at least six biological replicates. * or ^#^ Depicts a statistically significant difference for vessels >50 μm size: **P < 0.01 and ***P < 0.001 (paired *t* test with parametric test compared to day 0). # depicts a statistically significant difference for vessels <15 μm size: ^#^*P* < 0.05, ^##^*P* < 0.01, and ^###^*P* < 0.001 (paired *t* test with parametric test compared to day 0). Source data are available online for this figure.

Based on excessive muscularization combined with high PH, we speculated that HIF2α overexpression in NG2 (pericyte-selective) may switch to SMC phenotype (Fig. 7A). To further examine how HIF2α overexpressing NG2 cell migrated over time, we bred the NG2^HIF2α OE^ with ROSA26-tdTomato (Ai14), which permitted tamoxifen-inducible expression of HIF2α and tdT (red fluorescence) in NG2 promoter-driven cells for tracing HIF2α OE cells in vivo (i.e., NG2tdT^HIF2α OE^; Fig. 7B).

On day 0, tdT cells were mainly present in the parenchymal and exhibited the healthy/original morphology of thin elongated punctate processes (Fig. 7C). As Hx progressed on days 2–4, an increased number of tdT cells migrated from the alveolar-capillary space to nearby regions of medium-sized (>50 µm) vessels. Up to day 7, tdT cells moved closer to the adjacent arterioles, and their processes were multiplied and shortened, reaching out to neighboring tdT cells. After 14 days of Hx, tdT processes were abundant, thickened and flattened while some of the tdT cells covered arterioles and co-expressed SMA. Noticeably, more tdT cells aggregated near arterioles and formed unexpected “surrounding” patterns. After 21 days of Hx, a significant number of tdT cells covered the stem of the medium-sized vessels, along with accumulation at the tip of distal microvessels and SMA + . These lineage tracing and staining results strongly suggested tdT cell differentiation into SMC-like cells, thus confirming that NG2 cell differentiation contributed to vessel remodeling via a HIF2α activation.

Similar to confocal images, light-sheet microscopic images of NG2tdT^HIF2α OE^ mice exposed to 3 week of Hx displayed tdT cells wrapped around arterioles (>50 µm in diameter), and distal microvessels (<20 µm) engaging in muscularization with a considerable increase in the SMA expression (Fig. 7D; Movies EV7 and 8). As expected, highly muscularized microvessels covered with tdT+SMA+ cells of arterioles were more prominent in NG2tdT^HIF2α OE^ mice exposed to 6-week Hx (Appendix Fig. S12A). In addition, we tested whether reoxygenation would reverse the vascular remodeling by exposing NG2tdT^HIF2α OE^ mice to 6-week Hx and then re-exposing to 3-week normoxia (Appendix Fig. S12B). Interestingly, a significant decrease in SMA expression and minimum tdT coverage at distal microvessels was found. We speculate that the restoration of healthy vasculature was due to the HIF2α deactivation in a normoxic condition.

**Figure EV4:**
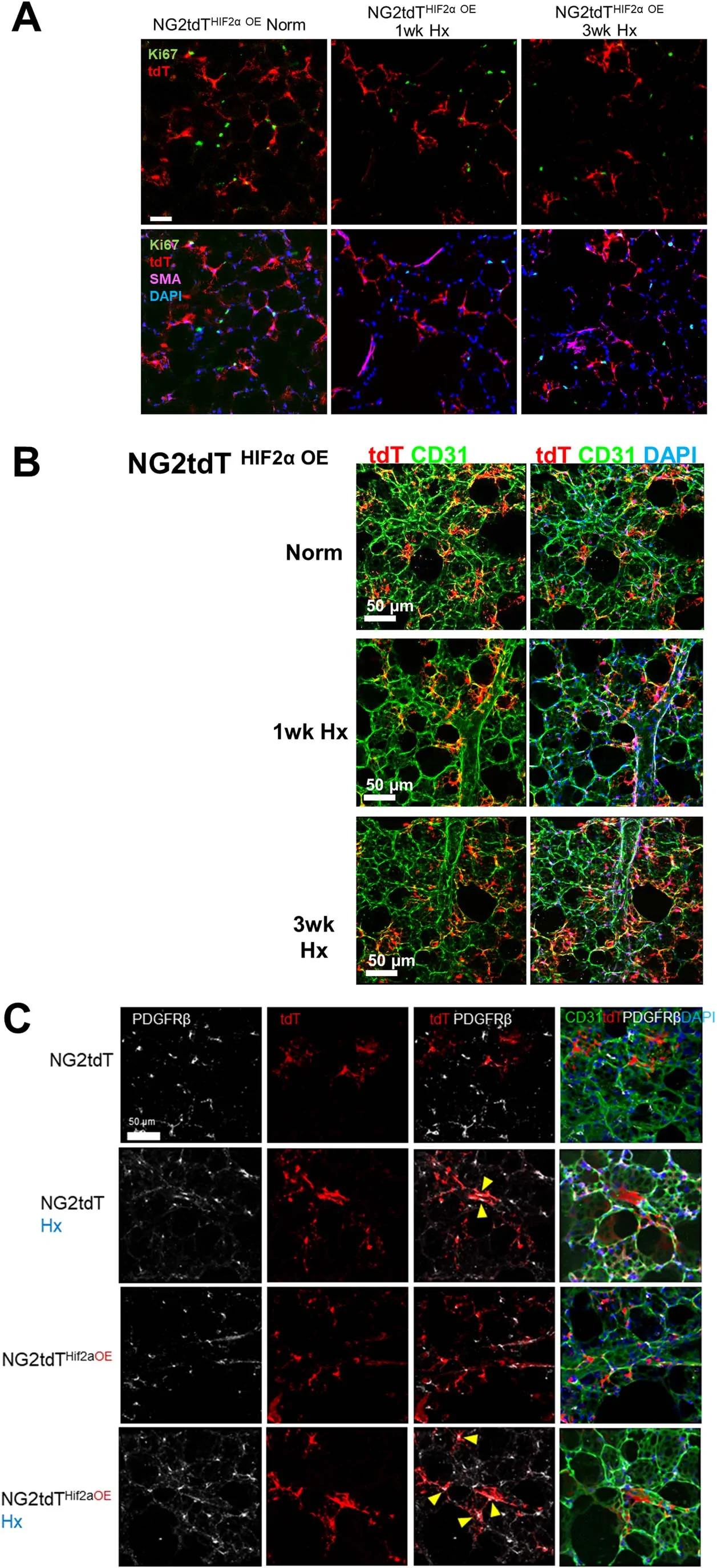
Characterization of tdT cells in NG2tdT^HIF2α OE^ lungs. (**A**) tdT cells did not colocalize with Ki67 (cell proliferation marker) under normoxia and hypoxia over time (1 wk and 3-wk Hx), by comparing the precision-cut lung slices of NG2tdT^HIF2α OE^ mice. Slices were stained for Ki67 (green), tdTamato (red), SMA (magenta) and DAPI (blue). (**B**) tdT cells closely contacted with CD31(Green) endothelial cells under normoxia and hypoxia over time (1 wk and 3-wk Hx). (**C**) tdT cells from both NG2tdT and NG2tdT^HIF2α OE^ co-expressed PDGFRβ (White) under normoxia and 3-wk Hx. Yellow arrowheads indicate tdT wrapped around arterioles and co-expressed PDGFRβ. Scale bar = 50 µm.

Next, we sought to document and quantify the number and distribution of NG2tdT ^HIF2α OE^ cells on arterioles with different diameters at each time point by using 3D mesh construction via computational deep learning software Aivia (Fig. 7E,F; Appendix Fig. S10B). Before Hx exposure, more than 92% of tdT cells were located on the capillaries (<15 µm in diameter). As the duration of Hx exposure increased, the number of tdT cells covering the middle and large-size vessels also increased, whereas the number of tdT cells in small microvessels decreased. Consistent with the confocal images, after 3 week Hx, more than 52% of tdT cells were located on the middle and large-size vessels (25% at 15–50 µm and 27% at >50 µm), indicating the HIF2α activated tdT cells were necessary for vascular remodeling. Furthermore, we used the cell proliferation marker Ki67 to explore whether tdT cells located in the larger blood vessels originated from their daughter cells (Fig. EV4). There was a minimal (<5%) colocalization of tdT and Ki67 signals, suggesting that tdT cells were not proliferative, consistent with the results of cell proliferation assay in human pericytes in vitro (Fig. EV4A). Instead of proliferation, continuous recruitment events under hypoxia may have occurred as tdT remained PDGFRβ + , directly contacted with CD31+ ECs and relocated to arterioles (Fig. EV4B,C). Lastly, to test if recruitment of mural cell coverage affected the distal alveolar structure, staining of podoplanin (PDPN) and surfactant protein C (SP-C) were chosen as markers for alveolar epithelial type I (ATI) and type II cells (AT2), respectively (Appendix Figs. S13 and 14). No distinctive structural change or cell number difference in AT1 and AT2 was shown in hypoxic NG2^HIF2α OE^ and NG2^HIF2α KO^. Thus, HIF2α activation only impacted on NG2 cells.

### HIF2α overexpression in pericyte-dominant transgenic mice attenuate from PH by HIF2α related inhibitor

**Figure 8:**
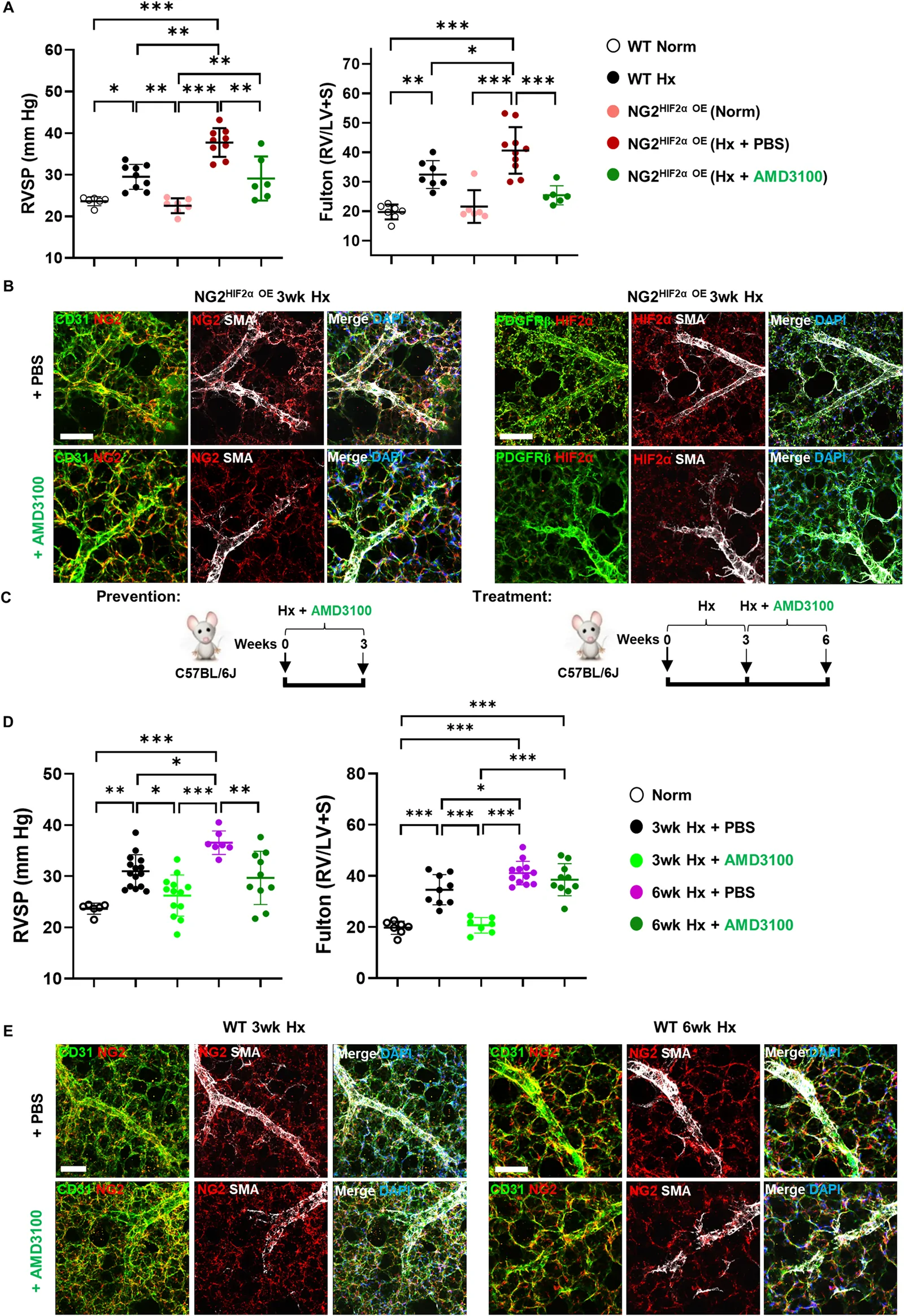
HIF2α overexpression in pericyte-dominant transgenic mice attenuate the effects of PH with a HIF2α-related inhibitor. (**A**) RVSP and Fulton Index were measured in NG2^HIF2α OE^ mice with AMD3100 and their corresponding controls. (**B**) Precision-cut lung slices of NG2^HIF2α OE^ mice in 3-week hypoxia with/without AMD3100 treatment were stained for CD31 (green), NG2 (red), and SMA (white) on the left and PDGFRβ (green), HIF2α (red), and SMA (white) on the right. Nuclei were stained with DAPI (blue). Corresponding control slices can be found in Fig. EV5. (**C**) The diagrams show the prevention and treatment models using AMD3100 and hypoxia on WT mice. (**D**) RVSP and Fulton Index were measured to evaluate the effectiveness of AMD3100 as a prevention or treatment against hypoxia-induced PH. (**E**) Precision-cut lung slices of WT mice in 3-week (left) and 6-week (right) hypoxia treated with AMD3100 as prevention or treatment strategies were stained for CD31 (green), NG2 (red), and SMA (white) with DAPI (blue) stained for nuclei. Data information: All scale bars = 50 µm. Means ± SEM was derived from at least six biological replicates. *Depicts a statistically significant difference: *P < 0.05, **P < 0.01, and ***P < 0.001 (one-way ANOVA with Turkey’s multiple comparisons test). Source data are available online for this figure.

**Figure EV5:**
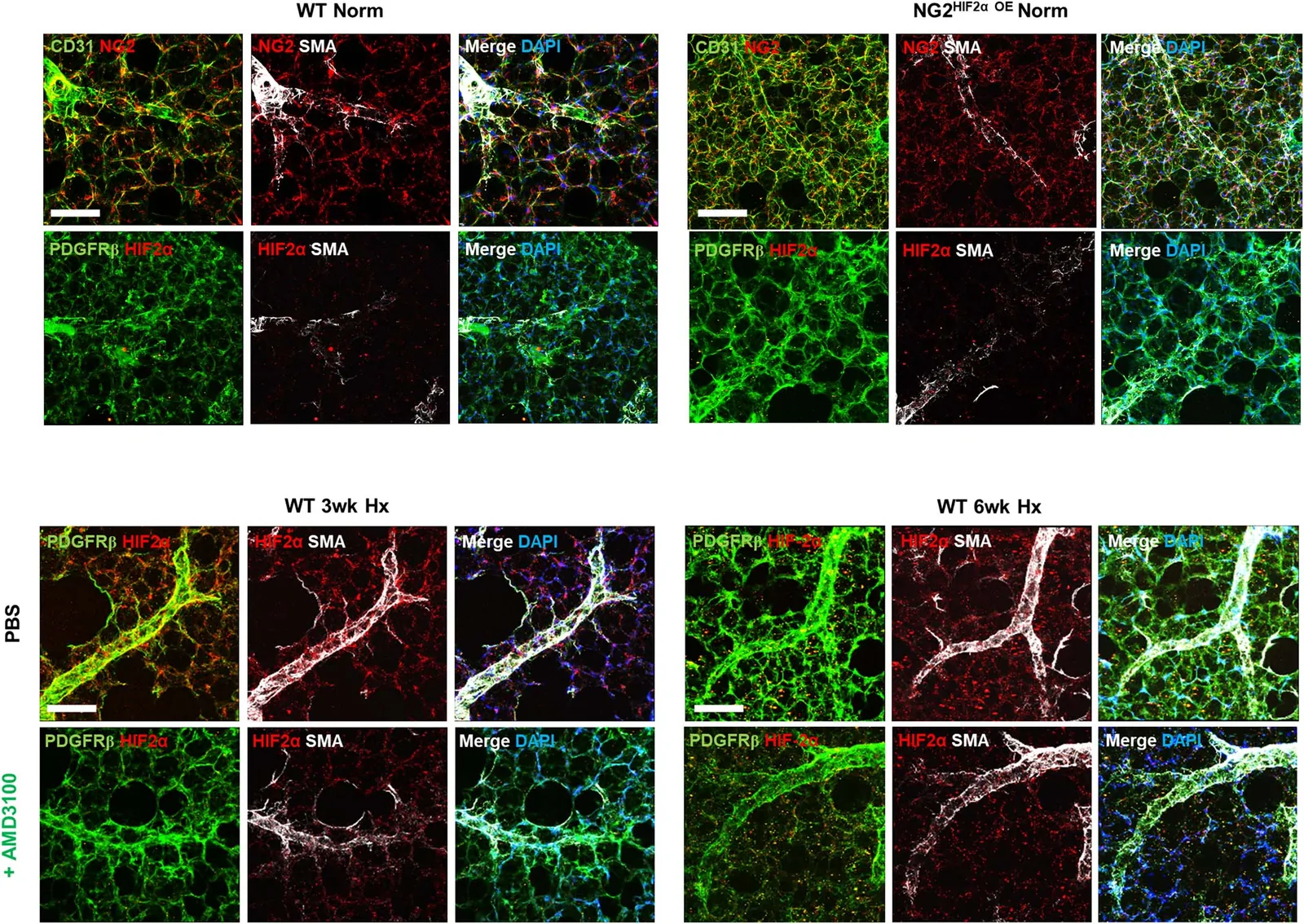
Treatment of AMD3100 successfully prevents and alleviates vessel muscularization. The precision-cut lung slices of WT mice in 3-wk (left) and 6-wk (right) hypoxia were stained for PDGFRβ (green), HIF2α (red), and SMA (white) with DAPI (blue) stained for nuclei. Scale bar = 50 µm.

In our previous work, hypoxic NG2tdT (NG2-Cre-ER x Ai14 tdTomato) mice treated with AMD3100 exhibited fewer or a complete absence of tdT cells associated with muscularized arterioles. In addition, mice that received AMD3100 had significantly lower RVSP and RVH compared to those who received PBS alone (Yuan et al, 2020). Since HIF2α exhibited transcriptional activity for chemokine SDF1, which was essential for directing cell migration through its receptor CXC chemokine receptor 4 (CXCR4), we sought to determine whether CXCR4 antagonist AMD3100 could exert a therapeutic effect on the NG2^HIF2α OE^ mice. We administered AMD3100 to NG2^HIF2α OE^ throughout 3-week Hx by using implantable osmotic pumps. Intriguingly, NG2^HIF2α OE^ mice with AMD3100 had lower RVSP and RV hypertrophy than those who received PBS alone (Fig. 8A). In addition, these animals exhibited less SMA expression in distal microvessels, fewer cells on the SMA positively stained arterioles, and more evenly distributed pericytes within the alveolar-capillary space indicated by PDGFRβ-positive and NG2-positive stained cells compared to controls (Figs. 8B and EV5). However, mice without AMD3100 treatment retained high RVSP and RVH, and the lung vasculature contained significant muscularization in pulmonary arterioles (PAs) where pericytes accumulated.

We further investigated the therapeutic role of AMD3100 as a prevention or a treatment plan on C57BL/6J background animals (Fig. 8C). AMD3100 was administered along when the mice underwent 3-week Hx. In the treatment plan, animals were exposed to 3-week Hx and then administered AMD3100 along with three more weeks of Hx. Consistent with our previous findings, the prevention plan protected animals from developing PH and reduced vascular remodeling and muscularization (Fig. 8D). However, 6-week Hx controls that received the treatment plan showed a reduced RVSP measurement but not a significant improvement in RVH, suggested a higher dosing AMD3100 may attenuate RVH. The precision lung cut slice staining showed reduced smooth muscle coverage on distal vessels (Figs. 8E and EV5). These data indicated that inhibition of HIF2α-mediated downstream pathway significantly reduced the initiation and development of Hx-induced PH and vascular remodeling.

## Discussion

Using a combination of bioinformatics analysis and genetic approaches, we found that HIF2α was a key mediator in driving pericytes to participate in distal vascular remodeling and muscularization. Cell cluster identification and lineage trajectory analysis from two distinct single-cell RNA datasets of human IPAH patient samples and mouse whole lungs indicated pericyte heterogeneity and their transformation into SMC-like cells. We demonstrated the upregulation of HIF2α in pericytes from clinical PAH lung samples. The overexpression of HIF2α in hypoxic human healthy pericytes resulted in increased expression levels of SMC markers and was also associated with abnormal polarity, reduced tube formation, endothelial barrier dysfunction, and increased contractility.

In hypoxic NG2^HIF2α OE^ animals, NG2 cells (pericyte-selective) overexpressing HIF2α transformed into SMC-like cells. These “abnormal” cells played an important role in the distal microvessel remodeling by migrating from the alveolar-capillary space to medium-sized vessels which resulted in reduced vascular integrity and stability. Moreover, hemodynamics analysis on NG2^HIF2α OE^ animals indicated more severe PH characteristics than controls, which included high RVSP, RVH, and significantly lower RV fractional area change, PA peak velocity, and PAT/PET ratio. These results were further evident by lineage tracing and 3D computational constructed mesh studies. In contrast, NG2^HIF2α KO^ animals exhibited substantially attenuated muscularization of distal microvessels, less mural cell migration, and intact vascular integrity and stability. Treatment of HIF2α related inhibitor AMD3100 successfully alleviated PH symptoms and features, which was reflected by improved RV function and more stabilized vasculature in hypoxic NG2^HIF2α OE^ animals.

The HIF family of transcriptional factors comprises three members HIF1α, 2α, and 3α (Lee et al, 2019; Semenza, 2012). Despite a substantial number of ongoing studies, the role of HIF1α in the development of Hx-induced PH remains controversial, most likely due to variations in the mode of Hx, experimental setting, and animal genotyping (global or cell-specific and embryonic vs inducible systems). Interestingly, our RNAseq datasets did not show any differential expressions of *HIF1a*, which led us to study HIF2α more closely. *EPAS1* was ubiquitously expressed in pulmonary EC clusters but showed no difference between IPAH vs. control ECs. In contrast, *EPAS1* expression was significantly upregulated in IPAH *PDGFRβ* + *CSPG4*+ cells, whose gene signatures were designated as pericytes. *EPAS1* was identified as one of the top mural cell signature markers in an independent and comprehensive fibroblast and mural cell identification and discrimination analysis (Muhl et al, 2020). Combining our previous work on pericyte SDF1 biology and its strong association with transcriptional activator HIF2α, our current work continued to explore pericyte transformation and its contribution to the development of pulmonary vascular remodeling in-depth.

Most published studies using transgenic mice regulate cell-type-specific HIFs from developing embryos and exposure to chronic Hx for PH development (Cowburn et al, 2016; Dai et al, 2016; Kapitsinou et al, 2016; Labrousse-Arias et al, 2016; Tang et al, 2018). To exclude possible side effects from gene deletion initiated in the embryonic stage, we utilized Cre-loxP transgenic strains to control HIF2α inducement and deletion more precisely in adult mice. Yet, one of the limitations of our work was that NG2 was not an exclusive marker for pericytes as our prior study found that NG2tdT had drawbacks with 10% tdT were other NG2 mural cells (SMCs or fibroblasts) (Yuan et al, 2020). To overcome this off-target effect, we carefully tamoxifen dosing based on the animal body weight and quantified that 90% tdT were pericytes (this method also labeled at least 40% pericytes at a capillary level when using a PDGFRb antibody). We further distinguished pericytes (SMA-) from SMCs (SMA + ) using their unique morphology. In addition, pericytes had oval cell bodies with long processes on capillaries, whereas SMCs had a spindle patchy pattern on arterioles bigger than 50 µm. Due to the non-specific label, the 10% other mural cells might contribute to the high pressure seen in NG2^HIF2α OE^ animals. Despite this potential synergistic effect, the PH protection seen in NG2^HIF2α KO^ animals provided strong complementary evidence that the HIF2α depletion selectively in NG2 cells was sufficient to protect from PH when compared to hypoxic NG2^HIF2α OE^.

Vascular remodeling is a dynamic, complex process that involves interplay between different cell types. The work by Sheikh and colleagues identify SMC precursors located in the junction of the pulmonary arteriole muscular–nonmuscularized border that migrate distally, thus contributing to muscularization (Sheikh et al, 2015). The same group also identifies myeloid cell lineages that represent SMCs and generate PDGFβ which induces muscularization (Sheikh et al, 2018). Others demonstrate that bone marrow-derived progenitor cells involved in vessel wall thickening co-express SMC markers, which contribute to the pulmonary vascular remodeling in hypoxia-induced PH (Davie et al, 2004; Hayashida et al, 2005). Our findings suggested a model in which capillary NG2+ cells (pericyte-selective) relocated and accumulated within precapillary arterioles under HIF2α activation, thereby contributing to vasoconstriction and muscularization. All the cell morphological and lineage changes may be synergistic events in the context of our work and others’ work. More defined and delineated intracellular mechanisms are needed to provide insights into the development of future therapeutics.

In addition, HIF2α had been shown as an indispensable regulator of proinflammatory cytokine/chemokine expression, including IL6, TNFα, and innate immunity (Imtiyaz et al, 2010; Ryu et al, 2014). An interesting approach for future study will explore whether NG2^HIF2α OE^ animals have any immune cell infiltration and cytokine secretion in capillaries. The inflammation may induce endothelial barrier dysfunction which results in capillary leakage and exacerbated large vessel remodeling. More work must be carried out to study this complex remodeling process in the pathogenesis of PH, particularly how to study early-on remodeling events in PAH patients. HIF2α has been associated with the stemness, differentiation, and regeneration of satellite cells (Xie et al, 2018). In addition, HIF2α-specific targeted expression of known pluripotent factors, such as OCT4, SOX2, and NANOG, can affect stem cell function and promote tumor growth (Covello et al, 2006; Das et al, 2019). Thus, further studies are needed to explore the critical role of HIF2α in cellular fate transition and homeostasis and whether inhibition of HIF2α can reverse mesenchymal differentiation.

There is growing interests in targeting HIF2α to treat human diseases outside of PAH. Recent studies have led to treating renal cell carcinoma by specifically inhibiting transcriptional activity of HIF2α (not HIF1α) with a small molecule named PT2385 and PT2977 (Wallace et al, 2016; Wehn et al, 2018; Xu et al, 2019). Regulation of HIF activity by small molecules in cancer occurs in different mechanisms. These HIF2α antagonists inhibit HIF2α transcriptional activity by inhibiting the protein–protein interaction between HIF2α and HIF1β, resulting in intense anti-tumor activity in vivo and in vitro (Wigerup et al, 2016). Given the potential pathological role of HIF2α in PH, the pharmacologic inhibition specifically targeting HIF2α not other HIFs could lay an important foundation for future treatment designs. More relevant to our work, PT2977 (a.k.a Belzutifan) mitigates PH and decreases RVH in three von Hippel Lindau (VHL) transgenic animal models, through SDF1/CXCR4 signaling axis (Ghosh et al, 2021). Moreover, a small HIF2α inhibitor (PT2567) significantly attenuates PH in the rat PH model by inhibiting monocyte recruitment and vascular cell proliferation (Hu et al, 2019). It would be intriguing to seek whether or not these inhibitory effects can be achieved in our transgenic animal models or in other vascular cell types including bone marrow-derived cells and fibrocytes, which are recruited to the vessel wall and differentiate into an SMC-like cell (Yeager et al, 2012). AMD3100 (a.k.a Plerixafor), an SDF1 receptor CXCR4 antagonist, is a preventive strategy to block the SDF1-activated pathway and is vital for cell migration during cardiovascular development (Dillenburg-Pilla et al, 2015). AMD3100 also prevents pulmonary arterial muscularization in several PH animal models (Bordenave et al, 2020; Farkas et al, 2014; Young et al, 2009). A similar ameliorating effect on PH is also noted using a different CXCR4 inhibitor called Silibinin (Zhang et al, 2019). In our study, treatment of AMD3100 attenuated PH and RV hypertrophy in hypoxic NG2^HIF2α OE^ animals and was effective as a prevention strategy in wild-type animals exposed to 3-week Hx. However, it was less effective as a therapeutic treatment on 6-week Hx-induced RVH. Therefore, a more comprehensive study should be carefully performed to characterize its efficacy until the point where RVH becomes a severe and irreversible condition.

To the best of our knowledge, we showed for the first time the critical role of HIF2α in pericytes as a critical mediator for vascular remodeling. Overexpression of HIF2α contributes to severe PH with progressive vessel remodeling and RVH, whereas knockout of HIF2α relieves PH. By lineage tracing of pericyte-selective HIF2α overexpressing cells under Hx, we demonstrate their relocation and involvement in vessel muscularization. Moreover, inhibition of the HIF2α downstream signaling pathway alleviates the development of PH. Identification of pericyte HIF2α signaling in the vascular remodeling process will be instrumental in developing novel approaches for future therapeutic strategies to treat PAH and other vascular remodeling-related diseases.

## Methods

### Clinical samples

Samples were obtained through PHBI networks (http://phbi.org/index.do) and clinical info is included in Appendix Table S2. This study was approved by the Institutional Review Board of Boston Children’s Hospital with approval IRB-P00039854.

### Animals

All experimental animal protocols used in this study were approved with protocol number 20-08-4069 by the Animal Care Committee and institutional guidelines of Boston Children’s Hospital and adhered to the published guidelines of the National Institutes of Health on the use of laboratory animals. LSL*-EPAS1* and EPAS1^flox/flox^ mice were a gift from Mark Nicolls Lab (Stanford University). B6.Cg-Tg(Cspg4-Cre/Esr1*)BAkik/J (NG2-Cre-ER) mice (https://www.jax.org/strain/008538), tdTomato Ai14 (https://www.jax.org/strain/007908) were purchased from Jax. Ear clip-based genotyping of the progeny was used to identify littermate controls, LSL*-EPAS1*, and EPAS1^flox/flox^ mice. Male mice were treated with 2 mg of tamoxifen (20 mg/ml dissolved in corn oil) for 5 consecutive days to induce the *EPAS1* overexpression or knockout. After tamoxifen treatments, mice were allowed to rest for 7–14 days before future experimentation. The average body weight of the mice used in our studies was 27–32 g and age from 2 to 4 months old.

#### AMD3100 treatment

AMD3100 was purchased from Tocris (Cat#3299, Tocris Bioscience, Ellisville) and reconstituted in 1× PBS for a working solution of 50 mg/mL. ALZET^®^ Osmotic Pumps (#1004) were filled with 5 mg of AMD3100 and inserted subcutaneously through an incision behind the ear and over the shoulder blade of the front leg. The pump was implanted subcutaneously on the back, started hypoxia day 1, and remained till day 21.

### scRNA-seq data pre-processing

Chromium Single Cell Software Suite v1.1 (10X Genomics) was used to pre-process the single-cell RNAseq data such as for sample de-multiplexing, barcode processing, and single-cell gene counting. Illumina BCL output folder was used for sample de-multiplexing based on the 8-bp sample index read and for generating FASTQ files for the single-end reads as well as the sample index by Illumina bcl2fastq. Next, reads in FASTQ files were mapped to mouse reference genome (UCSC mm10) with STAR. Reads that confidently mapped to the transcriptome were delivered in a BAM file. Chromium cellular barcodes were used to generate gene-barcode matrices. Filtered gene-barcode matrices containing only cellular barcodes in MEX format were used for downstream analysis.

### scRNA-seq data analysis

Genes expressed in <3 cells and cells with <200 or >6000 detected genes were excluded. Cells with the percentage of mitochondrial genes >5% were additionally filtered. The raw read counts generated by 10X CellRanger were further normalized by a global-scaling normalization method ¡°LogNormalize¡± that normalizes the gene expression measurements for each cell by the total expression. The log-transformed normalized single-cell expression values were used for visualizations and differential expression tests. The scaled z-scored values are used for principal component analysis (PCA). Statistically significant principal components determined by a resampling test were kept for Uniform Manifold Approximation and Projection (UMAP) analysis.

Differentially expressed genes (DEGs) among clusters were detected by comparing cells in each cluster against all other cells with the likelihood-ratio test. Gene A was defined as cluster X biomarker if it can be detected in >=25% cells, adjusted *P* value < 5% and fold change >=0.25 between cells of cluster X and all other cells. All these analyses were performed in the Seurat package v2.0 (Stuart et al, 2019). Reference: Comprehensive Integration of Single-Cell Data.

### Hypoxia studies

For hypoxia studies, male mice were placed in a hypoxia chamber and exposed to 10% inspired O_2_ with access to food and water ad libitum for up to 3 weeks. The chamber was regulated with a continuous mixture of room air and nitrogen gas to establish the hypoxic environment, while the chamber environment was monitored using an oxygen analyzer (Servomex, Sugar Land, TX). CO_2_ was removed via soda lime granules, and chamber temperature was maintained at ambient room temperature (22–24 °C). The chamber was inspected daily for O_2_ concentration, CO_2_ concentration, and animal welfare. Animals were euthanized using controlled isoflurane flow and cervical dislocation. Following sacrifice, animals were exsanguinated by perfusion through the right ventricle with 15–20 ml of 1× PBS at 37 °C. Lungs and hearts were harvested and processed for experiments discussed below.

### Mouse lung tissue clearing and precision-cut lung slices

Mice were sacrificed following institutional requirements. The pulmonary vasculature was flushed with PBS by inserting a needle connected to a reservoir into the right ventricle and allowing the fluid to drain through an incision in the dorsal aorta. The lung lobes were inflated with 2% low-melt agarose in PBS injected through the trachea. Agarose-filled lungs were solidified in ice-cold PBS, and the lobes were separated, fixed in 4% PFA at 4 °C overnight, followed by a PBS wash the next day.

#### For lung tissue clearing

Briefly, fixed lungs were gradually dehydrated into 100% methanol (day 0). Then, samples were incubated overnight in 33% methanol, 66% dichloromethane at RT (day 1), and treated with a solution of 5% H_2_O_2_/methanol overnight a 4 °C (day 2). On day 3, after progressive rehydration from 100% MeOH to PBS containing 0.2% Triton X-100 (PTx.2), samples were permeabilized overnight at 37 °C in PTx.2 with 2.3% glycine and 20% DMSO. Next, samples were blocked for 24 h at 37 °C in PTx.2 10% DMSO, 5% normal goat serum solution. On day 4, tissues were incubated in primary antibodies diluted in PTwH (PTx.2 with 0.001% heparin) 5% goat serum, 10% DMSO for 48 h at 37 °C. Then, samples were washed 6–7 times in PTwH until the next day. On day 7, samples are incubated for 48 h in secondary antibodies diluted in PTwH 5% goat serum at 37 °C. On day 9, after washing three times for 1 h in PTwH, samples were progressively dehydrated into 100% methanol overnight. On day 10, samples were incubated for 3 h in 33% methanol, 66% dichloromethane at room temperature. Following two 30-min washes in dichloromethane, specimens were cleared in dibenzyl ether (DBE).

#### For precision-cut lung slices

Each lobe (from mice or rats) was vibratome-sectioned (Leica VT1000 S) at a thickness of ~300 µm. Vibratome sections were blocked with 5% normal goat serum in 0.5% Triton X-100/PBS (PBS-T) for 1 h at room temperature and then incubated with primary antibodies overnight at 4 °C. The next day, the sections were washed three times in PBS-T (1 h per wash) followed by incubation with secondary antibodies overnight at 4 °C. The next day, the samples were washed three times in PBS-T and placed on slides in mounting media (Vector Labs). All images were captured using a Zeiss confocal 880 Airyscan 2 and processed by Aivia software.

### Immunofluorescence staining

#### Cell culture

For human pericyte in vitro experiments, staining was performed using the following steps: once the investigation had concluded, cells were washed with 1× PBS three times, followed by fixation by 4% paraformaldehyde for 10–15 min. Following fixation, cells were then permeabilized with 1× PBS containing 0.1% Triton X-100 and blocked with normal goat serum for 1 h, followed by primary antibody incubation overnight at 4 °C. The next day, cells were rewashed with 1× PBS and incubated with 488, 594, or 647 fluorophores containing secondary antibodies for 1 h at room temperature (Life Technologies Corporation). Slides were mounted with Prolong Gold Antifade Solution containing DAPI (Life Technologies Corporation).

#### Human and mouse lung tissue

For lung sections from human or mouse tissues, these sections were sourced from OCT-embedded tissues, which were thawed from −80 °C to room temperature, followed by washing in 1× PBS containing 0.1% Triton X-100 and blocked with normal goat serum for 1 h, followed by primary antibody incubation overnight at 4 °C. The next day, cells were rewashed with 1× PBS and incubated with 488, 594, or 647 fluorophores containing secondary antibodies for 1 h at room temperature. Slides were mounted with Prolong Gold Antifade Solution containing DAPI (Life Technologies Corporation). Mouse lung paraffin embedding and sectioning, followed by Trichrome and H&E staining, were performed by the Harvard Histopathology Core.

The following antibodies were used for human and mouse lung tissues, as well as in vitro fixed cell culture:

Rabbit-anti-mouse NG2 (1:100, AB5320, Millipore)

Rabbit-anti-mouse HIF2α (1:100, NB100-122, Novus Biologicals)

Mouse-anti-human HIF2α (1:100, NB100-132, Novus Biologicals)

Rabbit-anti-mouse SMMHC (1:100, J64817, Alfa Aesar)

Mouse-anti-mouse/human SMA-647 (1:100, sc-32251, Santa Cruz)

Rat-anti-mouse-PDGFRβ (1:100, MA5-15143, Thermo Scientific)

Hamster-anti-mouse-Podoplanin (1:100, 14-5381-81, eBioscience)

Rabbit-anti-mouse SP-C (1:100, AB3786, Millipore)

Rat-anti-mouse CD31(1:100; BD-Pharmingen, 553370 or 550274)

Sheep anti-human CD31 (1:100, AF806, R and D)

Rabbit-anti-human Calponin (1:100, Ab700, Abcam)

Goat-anti-human SM22(1:100, Ab10135, Abcam).

### Real-time quantitative PCR

Tissue and cell line mRNA expression was determined by quantitative Taqman real-time PCR. Total cellular RNA was extracted using the RNeasy Mini Kit (Qiagen, Redwood City, CA) and converted to cDNA using The High Capacity cDNA Reverse Transcription Kit with RNase Inhibitor (Thermo Fisher Scientific, MA, USA) following the manufacturer’s protocol. GAPDH was used for normalization. All real-time quantitative PCR studies were run in triplicates. ΔCT determined the difference in mRNA expression against GAPDH. Primers were purchased from Integrated DNA Technologies (IDT), IA, USA.

### Western blot

Tissues and cells were washed three times with ice-cold 1× PBS, and lysates were lysed in protein extraction solution (RIPA, G-Biosciences, MO, USA) with protease and phosphatase inhibitors (Sigma-Aldrich, Saint Louis, MO, USA) and vortexed for 10 s before centrifugation. Supernatants were transferred to fresh microcentrifuge tubes and stored at −20 °C. The protein concentration was determined by the Pierce BCA assay (Thermo Scientific Product #23227, Rockford, Illinois). Equal amounts of protein were loaded onto each lane of a 4–12% Bis-Tris gel (Life Technologies) and subjected to SDS-PAGE electrophoresis under reducing conditions. Proteins were transferred to polyvinylidene fluoride (PVDF) blotting membrane (GE Healthcare, Chicago, IL, USA) at 35 V for 2 h, blocked with 3% bovine serum albumin (BSA) in 1× Tris-buffered saline containing 0.1% Tween-20 (TBS-T) (pH 7.5), and incubated with primary antibodies overnight at 4 °C with gentle agitation. Membranes were incubated with horseradish peroxidase-conjugated secondary antibodies (1:2000, Bio-Rad, Hercules, CA, USA) for 1 h at room temperature. Finally, visualization was performed using the ECL Prime Western Blotting Detection Reagent (GE Healthcare, Chicago, IL, USA). Signal normalization was performed against GAPDH. Antibodies used were the following: HIF2α (1:1000, Catalog # NB100-122, Novus Biologicals, CO, USA), and GAPDH for loading control (1:5000, Catalog # 2118 L, Cell Signaling Technology, MA, USA).

### Vascular integrity and permeability assay

For Evans blue staining and vessel staining with lectin-FITC/Rhodamine-microbead, the mouse was anesthetized with isoflurane, and its body was tilted 30° head down for retro-orbital injection using 1-ml syringe capped with 27 g needle. Evans blue was injected at 3 µl/g body weights and waited for more than 5 min to circulate the solution. After the snout, paws, and tail showed blue, the mouse was euthanized and proceeded to tissue harvest. 100 µl of 0.1 mg/ml lectin-FITC from *Lycopersicon esculentum* (L32478; Thermo) was injected. After 5 min of incubation, an additional 100 µl of rhodamine-microbead (MCG-R50-EACH; Thermo) was injected into another eye and waited for 5 min. Then, the mouse was euthanized and proceeded to tissue harvest.

### Hemodynamics measurement and heart tissue histology

Right ventricular systolic pressure (RVSP) was measured under isoflurane anesthesia (1.5% in 2LPM O_2_) by inserting a 1.4 F catheter (Millar Instruments) via the right jugular E2 vein, as described previously. Hemodynamic tracings were analyzed using PowerLab software. After sacrifice, hearts were obtained and evaluated for the Fulton Index. The division between the right and left ventricles was identified, and the right ventricle was removed. The masses of the right ventricle and left ventricle plus septum were measured. Calculations were completed as follows: Fulton Index = (Right Ventricle Mass)/(Left Ventricle Mass + Septum Mass).

For optimal cutting temperature compound (OCT compound) embedding, the hearts were removed, washed in PBS, and fixed in 4% paraformaldehyde solution for 24 h. After 24 h, the fixed hearts were washed in PBS for 24 h and embedded in 30% sucrose for 24 h. Then, the fixed hearts cut in the short-axis direction at the one-third level from the apex to the base and in the same location between different groups and then processed for OCT embedding. The OCT-embedded sectioned samples were thawed from −80 °C to room temperature, followed by washing in 1× PBS and stained with hematoxylin and eosin. Images were captured with a light microscope (Olympus BX-41 upright microscope, Olympus) at 4× objective.

### Echocardiography

RV and LV function were assessed by functional rodent echocardiography according to the procedures described previously. Briefly, mice were anesthetized in an induction chamber filled with 3.5% isoflurane mixed with 100% oxygen and then placed in a supine position on a heated stage containing ECG leads. The heated stage also held a facemask with 1.5% isoflurane in oxygen delivered at 1.0 L/min and an anal temperature probe by which temperature was maintained at 37 °C. Then mice were subjected to transthoracic echo using VisualSonics Vevo 3100 (VisualSonics Inc.Toronto, ON, Canada) and transducer (MS-550D, 22–55 MHz). A chemical hair remover was used to remove the chest fur. RVFAC was measured via parasternal short-axis view, and RVFWT was measured via parasternal long-axis RV outflow tract-level M-mode. Pulse-wave Doppler echo was used to record the pulmonary blood outflow at the aortic valve level in the short-axis view to measure PAPV, PAT, and PET. LV function was achieved by measuring LV internal dimensions (LVID), and posterior wall thicknesses (PW) at diastole and systole from M-mode images at the level of the papillary muscles. All measurements were taken in compliance with the American Society of Echocardiography guidelines.

### Cell culture

#### Pericytes

Pericytes were obtained from human donor lung tissue samples, and lineage was confirmed by flow cytometry and western immunoblotting as previously described (Sorensen et al, 2009; Yuan et al, 2020). Pericytes were cultured in pericyte medium used with pericyte growth supplement (ScienCell, Cat #1252), 2 ml FBS, and 5 mL penicillin/streptomycin solutions. pcDNA3.1-HIF2a and its control plasmids were kindly provided by Dr. Chengjun Hu (University of Colorado).

#### Pulmonary artery endothelial cell (PAEC)

PAECs were obtained from PromoCell (PromoCell GmbH, Heidelberg, Germany) or ScienCell. All PAECs were cultured in endothelial cell media with 5% FBS, endothelial cell growth supplement (ScienCell, Cat #1001), and penicillin/streptomycin solutions.

### Wound-healing assay

Cell-culture plates with inserts (catalog number: 81176) were purchased from ibidi GmbH (Munich, Germany). Around 1.5 × 10^4^ cells (Pericytes and PC^HIF2α OE^) were seeded into either side of the inserts. Cells were synchronized using starvation media for 12 h before removal of the inserts. Serial images of the gap between Pericytes and PC^HIF2α OE^ were taken for 10 h. Cell migration rate and orientation were calculated by comparing cells at 0 and 10 h. Pericentrin (1:1000; ab4448; Abcam) and rhodamine-phalloidin (1:400; Invitrogen) were used to stain the microtubule-organizing center (MTOC) and actin filaments, respectively. For the quantification of polarized cells, polarized cells were shown as percentage against the total cells in the image field. Total cells were counted by the DAPI-stained cells, and polarized cells were counted by the orientation of pericentrin to its nucleus.

### Proliferation assay

Cells were seeded in triplicate on 96-well plates (2.0 × 10^3^ cells per well) (Nunc, Rochester, NY) and cultured in complete medium overnight at 37 °C and 5% CO_2_. The next day, cells were starved in the blank medium for 24 h. Day 0 was the baseline for comparison measured right before changing back to complete medium. Cell numbers were calculated on days 1, 2, and 3 using the CellTiter 96 Aqueous One Solution Cell Proliferation Assay (Promega, Sunnyvale, CA) following the manufacturer’s instructions.

### Matrigel co-culture tube formation assay

Matrigel (Thermo Fisher, #12760013) was placed on ice and thawed overnight in a 4 °C refrigerator then dispersed on an Ibidi μ-Slide Angiogenesis (Ibidi product #81506, Martinsned, Germany) with 10 μL per well and allowed to polymerize for 30 min at 37 °C. PAECs and pericytes were stained with cell membrane dye PKH67 green (MINI67; Sigma-Aldrich, USA) and PKH red (MINI26; Sigma-Aldrich, USA), respectively, following the manufacturer’s protocol. Approximately 5.0 × 10^3^ PAECs were mixed with ~1.0 × 10^3^ pericytes in 100 μL of pericyte medium and loaded per well. After 6 h, images were obtained via light microscope (Olympus BX-41 upright microscope, Olympus). Total tube loops and length were quantified using the angiogenesis analyzer plug-in of ImageJ software. Numbers were based off three replicates per experiment and each experiment was repeated at least three times.

### Contractility assay

Before starting the assay, cells were prepared, serum-starved, and incubated at 37 °C overnight. The following day, 3 mL of ice-cold collagen solution was prepared by mixing 1.8 mL of 1× DMEM with 0.3 ml FBS and 0.75 ml of Bovine Collagen-1 in a 50 ml falcon tube. The solution was kept on ice to prevent solidification when adjusting the pH to 7.4 using 0.1 N NaOH. A cell suspension was prepared (10^5^ cells/ml collagen solution) and plated in triplicate in a 96-well plate with 100 µl of collagen per well. To prepare cell suspension, media was aspirated, and plates were washed twice with 8 mL of 1× PBS followed by incubation with 1 mL of trypsin for 5 min at 37 °C. After 5 min, 5 mL of pericyte media was added to stop the reaction, and 10 µl of cells were transferred from the culture plate to a hemocytometer slide to estimate cell count before suspension in collagen solution. After plating the collagen-cell suspension, the plate was incubated at 37 °C for 30 min. Once solidified, 100 µl of pericyte media was added on top of the gels. Images were captured with a light microscope (Olympus BX-41 upright microscope, Olympus) at 4× objective after 72 h. The surface area was quantified using ImageJ.

### Pericyte and smooth muscle cell isolation

Lung tissues from WT and NG2^HIF2α OE^ mice were prepared to isolate PC and SMC. The day before the isolation, 25 μL of sheep anti-rat IgG Dynabeads (11035; Invitrogen, Carlsbad, CA) were incubated with 10 μg of CD31, CD45, CD146 IgG, overnight. The next day, fresh lung tissue was washed with 1× PBS twice to remove medium. Then, the tissue was minced and digested in a solution by using Miltenyi GentleMACS dissociator (Miltenyi Biotec, Germany). After the dissociation, 20 mL of cold PBS containing 0.1% FBS was added immediately. The suspension was passed through a BD Falcon 70-μm cell strainer (BD Biosciences, San Jose, CA) to remove debris or undigested fibrous tissue. The cell pellet was resuspended in 1 mL 1× PBS containing 40 μL of CD45 IgG-coated magnetic beads and gently rotated at 4 °C for 20 min. CD45 beads were then depleted by the magnet, and 40 μl of CD146 IgG-coated magnetic beads was mixed with the left-over cell solution and gently rotated at 4 °C for 30 min. CD146-beads were collected and wash with 1× PBS three times and used for experiments as isolated SMC. Left-over cell solution was mixed with 40 μL of CD31 IgG-coated magnetic beads and gently rotated at 4 °C for 30 min. CD31-beads were depleted from the solution, and lastly the left-over cell solution was incubated at 4 °C for 30 min with 6.6 μL of CD140b-APC IgG. After 30 min, cells were washed with 1× PBS three times, and resuspended in 100 μL of anti-APC Microbeads 4 °C for 30 min. Finally, by MACS, PCs were isolated by the position selection with CD140b-APC microbeads. The purity of Cd140b+ cells was further tested by FACS, using Cd31 and Pdgfrb antibodies. The FACS showed 1.5% CD31 + 85.7% Pdgfrb +.

### Fluorescence quantification using Aivia

First, the pixel classifier in the tab of the Analysis Panel was used to train the Aivia software to track only the target objects. Then, the background pixel and targeted pixel (wanted channels) were set up using the drawing tools under 3D tools. Using the “Teach” tab under the Pixel classifier, the software applied the same commands to all the regions of interest.

#### Microsphere quantification

Selected the fluorescent image and imported it into the software. Using ‘Cell analysis’ tab under the Recipe Console panel, the presetting function of vesicle detection was applied. Morphological, intensity, position, and count measurements were achieved by clicking the Export Spreadsheet icon.

#### 3D mesh conversion

Using the “3D cell analysis – Mesh” tab in the Recipe Console panel, the parameters of the Detection and Partition group were set up to detect the object of interest. After the proper selections of region of interests, morphological and intensity measurements for each converted 3D object such as surface area, volume, length etc. were generated.

### Experimental study design and statistical analysis

The number of samples studied per experiment is indicated in Figure Legends. All data are expressed as mean ± standard deviation (SD). All experiments were repeated at least three times and repeated as biological replicates. If not specify, the data collected were under randomized procedures, blinded and all included. Statistical significance was determined using paired *t* test or ordinary one-way ANOVA with Turkey’s multiple comparison tests unless stated otherwise with *P < 0.05, **P < 0.01, ***P < 0.005.

## Supplementary information

Appendix

Movie EV1

Movie EV2

Movie EV3

Movie EV4

Movie EV5

Movie EV6

Movie EV7

Movie EV8

Source Data Fig. 1

Source Data Fig. 2

Source Data Fig. 4

Source Data Fig. 5

Source Data Fig. 6

Source Data Fig. 7

Source Data Fig. 8

Peer Review File

Expanded View Figures

## Data Availability

Raw and analyzed murine scRNA-seq data generated in this study will be available through the Gene Expression Omnibus under the GEO accession number GSE249461. The single-cell RNAseq pipeline (including cellranger-7.0.0, Seurat v3.6, and Monocle v2.0) was used to process all fastq files into the read counts and visualization files. Cell ranger was used to calculate raw read counts for each gene and each cell. Seurat was used for the UMAP clustering and identify differentially expressed genes. Monocle was used for calculating the pseudotime for cells.

## References

[CR1] Abe K, Toba M, Alzoubi A, Ito M, Fagan KA, Cool CD, Voelkel NF, McMurtry IF, Oka M (2010) Formation of plexiform lesions in experimental severe pulmonary arterial hypertension. Circulation 121:2747–54 20547927 10.1161/CIRCULATIONAHA.109.927681

[CR2] Baek SH, Maiorino E, Kim H, Glass K, Raby BA, Yuan K (2022) Single cell transcriptomic analysis reveals organ specific pericyte markers and identities. Front Cardiovasc Med 9:876591 35722109 10.3389/fcvm.2022.876591 PMC9199463

[CR3] Bergers G, Song S (2005) The role of pericytes in blood-vessel formation and maintenance. Neuro Oncol 7:452–64 16212810 10.1215/S1152851705000232 PMC1871727

[CR4] Bordenave J, Thuillet R, Tu L, Phan C, Cumont A, Marsol C, Huertas A, Savale L, Hibert M, Galzi JL, Bonnet D, Humbert M, Frossard N, Guignabert C (2020) Neutralization of CXCL12 attenuates established pulmonary hypertension in rats. Cardiovasc Res 116:686–697 31173066 10.1093/cvr/cvz153

[CR5] Brusselmans K, Compernolle V, Tjwa M, Wiesener MS, Maxwell PH, Collen D, Carmeliet P (2003) Heterozygous deficiency of hypoxia-inducible factor-2alpha protects mice against pulmonary hypertension and right ventricular dysfunction during prolonged hypoxia. J Clin Investig 111:1519–27 12750401 10.1172/JCI15496 PMC155039

[CR6] Ceradini DJ, Kulkarni AR, Callaghan MJ, Tepper OM, Bastidas N, Kleinman ME, Capla JM, Galiano RD, Levine JP, Gurtner GC (2004) Progenitor cell trafficking is regulated by hypoxic gradients through HIF-1 induction of SDF-1. Nat Med 10:858–64 15235597 10.1038/nm1075

[CR7] Chan XY, Volkova E, Eoh J, Black R, Fang L, Gorashi R, Song J, Wang J, Elliott MB, Barreto-Ortiz SF, Chen J, Lin BL, Santhanam L, Cheng L, Lee FS, Prchal JT, Gerecht S (2021) HIF2A gain-of-function mutation modulates the stiffness of smooth muscle cells and compromises vascular mechanics. iScience 24:102246 33796838 10.1016/j.isci.2021.102246 PMC7995528

[CR8] Compernolle V, Brusselmans K, Acker T, Hoet P, Tjwa M, Beck H, Plaisance S, Dor Y, Keshet E, Lupu F, Nemery B, Dewerchin M, Van Veldhoven P, Plate K, Moons L, Collen D, Carmeliet P (2002) Loss of HIF-2alpha and inhibition of VEGF impair fetal lung maturation, whereas treatment with VEGF prevents fatal respiratory distress in premature mice. Nat Med 8:702–10 12053176 10.1038/nm721

[CR9] Covello KL, Kehler J, Yu H, Gordan JD, Arsham AM, Hu CJ, Labosky PA, Simon MC, Keith B (2006) HIF-2alpha regulates Oct-4: effects of hypoxia on stem cell function, embryonic development, and tumor growth. Genes Dev 20:557–70 16510872 10.1101/gad.1399906 PMC1410808

[CR10] Cowburn AS, Crosby A, Macias D, Branco C, Colaco RD, Southwood M, Toshner M, Crotty Alexander LE, Morrell NW, Chilvers ER, Johnson RS (2016) HIF2alpha-arginase axis is essential for the development of pulmonary hypertension. Proc Natl Acad Sci USA 113:8801–6 27432976 10.1073/pnas.1602978113 PMC4978263

[CR11] Dai Z, Li M, Wharton J, Zhu MM, Zhao YY (2016) Prolyl-4 hydroxylase 2 (PHD2) deficiency in endothelial cells and hematopoietic cells induces obliterative vascular remodeling and severe pulmonary arterial hypertension in mice and humans through hypoxia-inducible factor-2alpha. Circulation 133:2447–58 27143681 10.1161/CIRCULATIONAHA.116.021494 PMC4907810

[CR12] Das B, Pal B, Bhuyan R, Li H, Sarma A, Gayan S, Talukdar J, Sandhya S, Bhuyan S, Gogoi G, Gouw AM, Baishya D, Gotlib JR, Kataki AC, Felsher DW (2019) MYC regulates the HIF2alpha stemness pathway via Nanog and Sox2 to maintain self-renewal in cancer stem cells versus non-stem cancer cells. Cancer Res 79:4015–4025 31266772 10.1158/0008-5472.CAN-18-2847 PMC6701948

[CR13] Davie NJ, Crossno Jr JT, Frid MG, Hofmeister SE, Reeves JT, Hyde DM, Carpenter TC, Brunetti JA, McNiece IK, Stenmark KR (2004) Hypoxia-induced pulmonary artery adventitial remodeling and neovascularization: contribution of progenitor cells. Am J Physiol Lung Cell Mol Physiol 286:L668–78 12754186 10.1152/ajplung.00108.2003

[CR14] Dengler VL, Galbraith M, Espinosa JM (2014) Transcriptional regulation by hypoxia inducible factors. Crit Rev Biochem Mol Biol 49:1–15 24099156 10.3109/10409238.2013.838205 PMC4342852

[CR15] Diaz-Flores L, Gutierrez R, Madrid JF, Varela H, Valladares F, Acosta E, Martin-Vasallo P, Diaz-Flores Jr L (2009) Pericytes. Morphofunction, interactions and pathology in a quiescent and activated mesenchymal cell niche. Histol Histopathol 24:909–69 19475537 10.14670/HH-24.909

[CR16] Dillenburg-Pilla P, Patel V, Mikelis CM, Zarate-Blades CR, Doci CL, Amornphimoltham P, Wang Z, Martin D, Leelahavanichkul K, Dorsam RT, Masedunskas A, Weigert R, Molinolo AA, Gutkind JS (2015) SDF-1/CXCL12 induces directional cell migration and spontaneous metastasis via a CXCR4/Galphai/mTORC1 axis. FASEB J 29:1056–68 25466898 10.1096/fj.14-260083 PMC4422355

[CR17] Farkas D, Kraskauskas D, Drake JI, Alhussaini AA, Kraskauskiene V, Bogaard HJ, Cool CD, Voelkel NF, Farkas L (2014) CXCR4 inhibition ameliorates severe obliterative pulmonary hypertension and accumulation of C-kit(+) cells in rats. PLoS ONE 9:e89810 24587052 10.1371/journal.pone.0089810 PMC3933653

[CR18] Ghosh MC, Zhang DL, Ollivierre WH, Noguchi A, Springer DA, Linehan WM, Rouault TA (2021) Therapeutic inhibition of HIF-2alpha reverses polycythemia and pulmonary hypertension in murine models of human diseases. Blood 137:2509–2519 33512384 10.1182/blood.2020009138 PMC8109019

[CR19] Haase VH (2009) Oxygen regulates epithelial-to-mesenchymal transition: insights into molecular mechanisms and relevance to disease. Kidney Int 76:492–9 19536078 10.1038/ki.2009.222 PMC3623274

[CR20] Hayashida K, Fujita J, Miyake Y, Kawada H, Ando K, Ogawa S, Fukuda K (2005) Bone marrow-derived cells contribute to pulmonary vascular remodeling in hypoxia-induced pulmonary hypertension. Chest 127:1793–8 15888860 10.1378/chest.127.5.1793

[CR21] Howard LS, Crosby A, Vaughan P, Sobolewski A, Southwood M, Foster ML, Chilvers ER, Morrell NW (2012) Distinct responses to hypoxia in subpopulations of distal pulmonary artery cells contribute to pulmonary vascular remodeling in emphysema. Pulm Circ 2:241–9 22837865 10.4103/2045-8932.97616 PMC3401878

[CR22] Hu CJ, Poth JM, Zhang H, Flockton A, Laux A, Kumar S, McKeon B, Mouradian G, Li M, Riddle S, Pugliese SC, Brown RD, Wallace EM, Graham BB, Frid MG, Stenmark KR (2019) Suppression of HIF2 signalling attenuates the initiation of hypoxia-induced pulmonary hypertension. Eur Respir J 54:1900378 31515405 10.1183/13993003.00378-2019 PMC6911916

[CR23] Humbert M, Guignabert C, Bonnet S, Dorfmuller P, Klinger JR, Nicolls MR, Olschewski AJ, Pullamsetti SS, Schermuly RT, Stenmark KR, Rabinovitch M (2019) Pathology and pathobiology of pulmonary hypertension: state of the art and research perspectives. Eur Respir J 53:1801887 30545970 10.1183/13993003.01887-2018 PMC6351340

[CR24] Imtiyaz HZ, Williams EP, Hickey MM, Patel SA, Durham AC, Yuan LJ, Hammond R, Gimotty PA, Keith B, Simon MC (2010) Hypoxia-inducible factor 2alpha regulates macrophage function in mouse models of acute and tumor inflammation. J Clin Investig 120:2699–714 20644254 10.1172/JCI39506 PMC2912179

[CR25] Kapitsinou PP, Rajendran G, Astleford L, Michael M, Schonfeld MP, Fields T, Shay S, French JL, West J, Haase VH (2016) The endothelial prolyl-4-hydroxylase domain 2/hypoxia-inducible factor 2 axis regulates pulmonary artery pressure in mice. Mol Cell Biol 36:1584–94 26976644 10.1128/MCB.01055-15 PMC4859687

[CR26] Kim YM, Barnes EA, Alvira CM, Ying L, Reddy S, Cornfield DN (2013) Hypoxia-inducible factor-1alpha in pulmonary artery smooth muscle cells lowers vascular tone by decreasing myosin light chain phosphorylation. Circ Res 112:1230–3 23513056 10.1161/CIRCRESAHA.112.300646 PMC4005857

[CR27] Labrousse-Arias D, Castillo-Gonzalez R, Rogers NM, Torres-Capelli M, Barreira B, Aragones J, Cogolludo A, Isenberg JS, Calzada MJ (2016) HIF-2alpha-mediated induction of pulmonary thrombospondin-1 contributes to hypoxia-driven vascular remodelling and vasoconstriction. Cardiovasc Res 109:115–30 26503986 10.1093/cvr/cvv243 PMC4692290

[CR28] Lee JW, Ko J, Ju C, Eltzschig HK (2019) Hypoxia signaling in human diseases and therapeutic targets. Exp Mol Med 51:1–13 31221962 10.1038/s12276-019-0235-1 PMC6586801

[CR29] Lendahl U, Muhl L, Betsholtz C (2022) Identification, discrimination and heterogeneity of fibroblasts. Nat Commun 13:3409 35701396 10.1038/s41467-022-30633-9 PMC9192344

[CR30] Liao D, Corle C, Seagroves TN, Johnson RS (2007) Hypoxia-inducible factor-1alpha is a key regulator of metastasis in a transgenic model of cancer initiation and progression. Cancer Res 67:563–72 17234764 10.1158/0008-5472.CAN-06-2701

[CR31] Lilly B, Khan A, Manivannan S (2022) Gene expression Omnibus GSE209738 (https://www.ncbi.nlm.nih.gov/geo/query/acc.cgi?acc=GSE209738)

[CR32] Muhl L, Genove G, Leptidis S, Liu J, He L, Mocci G, Sun Y, Gustafsson S, Buyandelger B, Chivukula IV, Segerstolpe A, Raschperger E, Hansson EM, Bjorkegren JLM, Peng XR, Vanlandewijck M, Lendahl U, Betsholtz C (2020) Publisher Correction: single-cell analysis uncovers fibroblast heterogeneity and criteria for fibroblast and mural cell identification and discrimination. Nat Commun 11:4493 32883975 10.1038/s41467-020-18511-8 PMC7471259

[CR33] Poels EM, Bitsch N, Slenter JM, Kooi ME, de Theije CC, de Windt LJ, van Empel VP, da Costa Martins PA (2014) Supplementing exposure to hypoxia with a copper depleted diet does not exacerbate right ventricular remodeling in mice. PLoS ONE 9:e92983 24736644 10.1371/journal.pone.0092983 PMC3988035

[CR34] Pugliese SC, Poth JM, Fini MA, Olschewski A, El Kasmi KC, Stenmark KR (2015) The role of inflammation in hypoxic pulmonary hypertension: from cellular mechanisms to clinical phenotypes. Am J Physiol Lung Cell Mol Physiol 308:L229–52 25416383 10.1152/ajplung.00238.2014 PMC4338929

[CR35] Ricard N, Tu L, Le Hiress M, Huertas A, Phan C, Thuillet R, Sattler C, Fadel E, Seferian A, Montani D, Dorfmuller P, Humbert M, Guignabert C (2014) Increased pericyte coverage mediated by endothelial-derived fibroblast growth factor-2 and interleukin-6 is a source of smooth muscle-like cells in pulmonary hypertension. Circulation 129:1586–97 24481949 10.1161/CIRCULATIONAHA.113.007469

[CR36] Ryu JH, Chae CS, Kwak JS, Oh H, Shin Y, Huh YH, Lee CG, Park YW, Chun CH, Kim YM, Im SH, Chun JS (2014) Hypoxia-inducible factor-2alpha is an essential catabolic regulator of inflammatory rheumatoid arthritis. PLoS Biol 12:e1001881 24914685 10.1371/journal.pbio.1001881 PMC4051611

[CR37] Saygin D, Tabib T, Bittar HE, Valenzi E, Sembrat J, Chan SY, Rojas M, Lafyatis R (2020) Gene expression Omnibus GSE169471 (https://www.ncbi.nlm.nih.gov/geo/query/acc.cgi?acc=GSE169471) 10.1177/2045894020908782 PMC7052475 32166015

[CR38] Saygin D, Tabib T, Bittar HET, Valenzi E, Sembrat J, Chan SY, Rojas M, Lafyatis R (2020) Transcriptional profiling of lung cell populations in idiopathic pulmonary arterial hypertension. Pulm Circ 10(1):1–15 10.1177/2045894020908782 PMC7052475 32166015

[CR39] Schultz K, Fanburg BL, Beasley D (2006) Hypoxia and hypoxia-inducible factor-1alpha promote growth factor-induced proliferation of human vascular smooth muscle cells. Am J Physiol Heart Circ Physiol 290:H2528–34 16399861 10.1152/ajpheart.01077.2005

[CR40] Scortegagna M, Ding K, Oktay Y, Gaur A, Thurmond F, Yan LJ, Marck BT, Matsumoto AM, Shelton JM, Richardson JA, Bennett MJ, Garcia JA (2003) Multiple organ pathology, metabolic abnormalities and impaired homeostasis of reactive oxygen species in Epas1-/- mice. Nat Genet 35:331–40 14608355 10.1038/ng1266

[CR41] Semenza GL (2012) Hypoxia-inducible factors in physiology and medicine. Cell 148:399–408 22304911 10.1016/j.cell.2012.01.021 PMC3437543

[CR42] Sheikh AQ, Misra A, Rosas IO, Adams RH, Greif DM (2015) Smooth muscle cell progenitors are primed to muscularize in pulmonary hypertension. Sci Transl Med 7:308ra159 26446956 10.1126/scitranslmed.aaa9712 PMC4629985

[CR43] Sheikh AQ, Saddouk FZ, Ntokou A, Mazurek R, Greif DM (2018) Cell autonomous and non-cell autonomous regulation of SMC progenitors in pulmonary hypertension. Cell Rep 23:1152–1165 29694892 10.1016/j.celrep.2018.03.043 PMC5959296

[CR44] Shimoda LA, Semenza GL (2011) HIF and the lung: role of hypoxia-inducible factors in pulmonary development and disease. Am J Respir Crit Care Med 183:152–6 21242594 10.1164/rccm.201009-1393PP PMC3159088

[CR45] Simonneau G, Montani D, Celermajer DS, Denton CP, Gatzoulis MA, Krowka M, Williams PG, Souza R (2019) Haemodynamic definitions and updated clinical classification of pulmonary hypertension. Eur Respir J 53:1801913 30545968 10.1183/13993003.01913-2018 PMC6351336

[CR46] Sorensen I, Adams RH, Gossler A (2009) DLL1-mediated Notch activation regulates endothelial identity in mouse fetal arteries. Blood 113:5680–8 19144989 10.1182/blood-2008-08-174508

[CR47] Stacher E, Graham BB, Hunt JM, Gandjeva A, Groshong SD, McLaughlin VV, Jessup M, Grizzle WE, Aldred MA, Cool CD, Tuder RM (2012) Modern age pathology of pulmonary arterial hypertension. Am J Respir Crit Care Med 186:261–72 22679007 10.1164/rccm.201201-0164OC PMC3886716

[CR48] Stratman AN, Malotte KM, Mahan RD, Davis MJ, Davis GE (2009) Pericyte recruitment during vasculogenic tube assembly stimulates endothelial basement membrane matrix formation. Blood 114:5091–101 19822899 10.1182/blood-2009-05-222364 PMC2788982

[CR49] Stuart T, Bulter A, Hoffman P, Hafemeister C, Papalexi E, Mauck WM, Hao Y, Stoeckius M, Smibert P, Satija R (2019) Comprehensive Integration of Single-Cell Data. Cell 177:1–15 10.1016/j.cell.2019.05.031 PMC6687398 31178118

[CR50] Tang H, Babicheva A, McDermott KM, Gu Y, Ayon RJ, Song S, Wang Z, Gupta A, Zhou T, Sun X, Dash S, Wang Z, Balistrieri A, Zheng Q, Cordery AG, Desai AA, Rischard F, Khalpey Z, Wang J, Black SM et al (2018) Endothelial HIF-2alpha contributes to severe pulmonary hypertension due to endothelial-to-mesenchymal transition. Am J Physiol Lung Cell Mol Physiol 314:L256–L275 29074488 10.1152/ajplung.00096.2017 PMC5866501

[CR51] Thomas S, Manivannan S, Garg V, Lilly B (2022) Single-cell RNA sequencing reveals novel genes regulated by hypoxia in the lung vasculature. J Vasc Res 59:163–175 35294950 10.1159/000522340 PMC9117417

[CR52] Vanlandewijck M, He L, Mae MA, Andrae J, Ando K, Del Gaudio F, Nahar K, Lebouvier T, Lavina B, Gouveia L, Sun Y, Raschperger E, Rasanen M, Zarb Y, Mochizuki N, Keller A, Lendahl U, Betsholtz C (2018) A molecular atlas of cell types and zonation in the brain vasculature. Nature 554:475–480 29443965 10.1038/nature25739

[CR53] Wallace EM, Rizzi JP, Han G, Wehn PM, Cao Z, Du X, Cheng T, Czerwinski RM, Dixon DD, Goggin BS, Grina JA, Halfmann MM, Maddie MA, Olive SR, Schlachter ST, Tan H, Wang B, Wang K, Xie S, Xu R et al (2016) A small-molecule antagonist of HIF2alpha is efficacious in preclinical models of renal cell carcinoma. Cancer Res 76:5491–500 27635045 10.1158/0008-5472.CAN-16-0473

[CR54] Wehn PM, Rizzi JP, Dixon DD, Grina JA, Schlachter ST, Wang B, Xu R, Yang H, Du X, Han G, Wang K, Cao Z, Cheng T, Czerwinski RM, Goggin BS, Huang H, Halfmann MM, Maddie MA, Morton EL, Olive SR et al (2018) Design and activity of specific hypoxia-inducible factor-2alpha (HIF-2alpha) inhibitors for the treatment of clear cell renal cell carcinoma: discovery of clinical candidate (S)-3-((2,2-difluoro-1-hydroxy-7-(methylsulfonyl)-2,3-dihydro-1 H-inden-4-yl)oxy)-5-fluorobenzonitrile (PT2385). J Med Chem 61:9691–9721 30289716 10.1021/acs.jmedchem.8b01196

[CR55] Wigerup C, Pahlman S, Bexell D (2016) Therapeutic targeting of hypoxia and hypoxia-inducible factors in cancer. Pharmacol Ther 164:152–69 27139518 10.1016/j.pharmthera.2016.04.009

[CR56] Xie L, Yin A, Nichenko AS, Beedle AM, Call JA, Yin H (2018) Transient HIF2A inhibition promotes satellite cell proliferation and muscle regeneration. J Clin Investig 128:2339–2355 29533927 10.1172/JCI96208 PMC5983316

[CR57] Xu R, Wang K, Rizzi JP, Huang H, Grina JA, Schlachter ST, Wang B, Wehn PM, Yang H, Dixon DD, Czerwinski RM, Du X, Ged EL, Han G, Tan H, Wong T, Xie S, Josey JA, Wallace EM (2019) 3-[(1S,2S,3R)-2,3-Difluoro-1-hydroxy-7-methylsulfonylindan-4-yl]oxy-5-fluorobenzo nitrile (PT2977), a hypoxia-inducible factor 2alpha (HIF-2alpha) inhibitor for the treatment of clear cell renal cell carcinoma. J Med Chem 62:6876–6893 10.1021/acs.jmedchem.9b00719 31282155

[CR58] Yamagishi S, Imaizumi T (2005) Pericyte biology and diseases. Int J Tissue React 27:125–35 16372479

[CR59] Yeager ME, Nguyen CM, Belchenko DD, Colvin KL, Takatsuki S, Ivy DD, Stenmark KR (2012) Circulating fibrocytes are increased in children and young adults with pulmonary hypertension. Eur Respir J 39:104–11 21700605 10.1183/09031936.00072311 PMC3319160

[CR60] Young KC, Torres E, Hatzistergos KE, Hehre D, Suguihara C, Hare JM (2009) Inhibition of the SDF-1/CXCR4 axis attenuates neonatal hypoxia-induced pulmonary hypertension. Circ Res 104:1293–301 19423843 10.1161/CIRCRESAHA.109.197533 PMC2757744

[CR61] Yuan G, Peng YJ, Reddy VD, Makarenko VV, Nanduri J, Khan SA, Garcia JA, Kumar GK, Semenza GL, Prabhakar NR (2013) Mutual antagonism between hypoxia-inducible factors 1alpha and 2alpha regulates oxygen sensing and cardio-respiratory homeostasis. Proc Natl Acad Sci USA 110:E1788–96 23610397 10.1073/pnas.1305961110 PMC3651442

[CR62] Yuan K, Orcholski ME, Panaroni C, Shuffle EM, Huang NF, Jiang X, Tian W, Vladar EK, Wang L, Nicolls MR, Wu JY, de Jesus Perez VA (2015) Activation of the Wnt/planar cell polarity pathway is required for pericyte recruitment during pulmonary angiogenesis. Am J Pathol 185:69–84 25447046 10.1016/j.ajpath.2014.09.013 PMC4278244

[CR63] Yuan K, Shamskhou EA, Orcholski ME, Nathan A, Reddy S, Honda H, Mani V, Zeng Y, Ozen MO, Wang L, Demirci U, Tian W, Nicolls MR, de Jesus Perez VA (2019) Loss of endothelium-derived Wnt5a is associated with reduced pericyte recruitment and small vessel loss in pulmonary arterial hypertension. Circulation 139:1710–1724 30586764 10.1161/CIRCULATIONAHA.118.037642 PMC6443444

[CR64] Yuan K, Liu Y, Zhang Y, Nathan A, Tian W, Yu J, Sweatt AJ, Shamshou EA, Condon D, Chakraborty A, Agarwal S, Auer N, Zhang S, Wu JC, Zamanian RT, Nicolls MR, de Jesus Perez VA (2020) Mural cell SDF1 signaling is associated with the pathogenesis of pulmonary arterial hypertension. Am J Respir Cell Mol Biol 62:747–759 32084325 10.1165/rcmb.2019-0401OC PMC7258825

[CR65] Zhang T, Kawaguchi N, Yoshihara K, Hayama E, Furutani Y, Kawaguchi K, Tanaka T, Nakanishi T (2019) Silibinin efficacy in a rat model of pulmonary arterial hypertension using monocrotaline and chronic hypoxia. Respir Res 20:79 31023308 10.1186/s12931-019-1041-y PMC6485095

[CR66] Zhu Z, Godana D, Li A, Rodriguez B, Gu C, Tang H, Minshall RD, Huang W, Chen J (2019) Echocardiographic assessment of right ventricular function in experimental pulmonary hypertension. Pulm Circ 9:2045894019841987 30942120 10.1177/2045894019841987 PMC6566495

