# Supplementary material for: Pericytes contribute to pulmonary vascular remodeling via HIF2α signaling
Source: EMBO Rep. 2024 Jan 19;25(2):13. doi: 10.1038/s44319-023-00054-w (PMC10897382; doi:10.1038/s44319-023-00054-w)
Supplement: Supplementary file 1 — Appendix [file 44319_2023_54_MOESM1_ESM.pdf]

# Table of Content


Appendix Figure S1 ------------------------------------------ Page 2

Appendix Figure S2 ------------------------------------------ Page 2

Appendix Figure S3 ------------------------------------------ Page 2

Appendix Figure S4 ------------------------------------------ Page 3

Appendix Figure S5 ------------------------------------------ Page 3

Appendix Figure S6 ------------------------------------------ Page 4

Appendix Figure S7 ------------------------------------------ Page 5

Appendix Figure S8 ------------------------------------------ Page 5

Appendix Figure S9 ------------------------------------------ Page 6

Appendix Figure S10 ------------------------------------------ Page 7

Appendix Figure S11 ------------------------------------------ Page 8

Appendix Figure S12 ------------------------------------------ Page 8

Appendix Figure S13 ------------------------------------------ Page 9

Appendix Figure S14 ------------------------------------------ Page 9

Appendix Table S1 ------------------------------------------ Page 10

Appendix Table S2 ------------------------------------------ Page 11

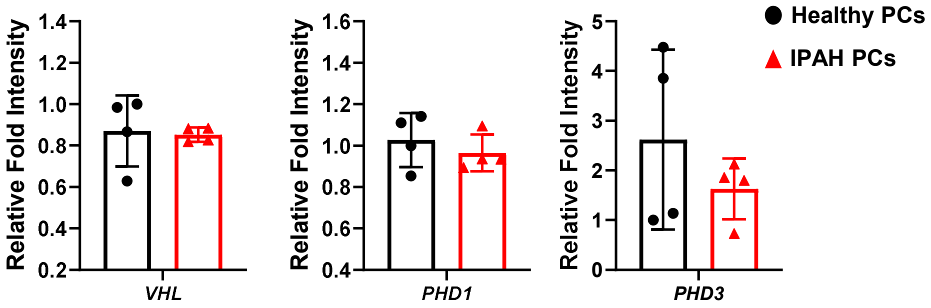


**Appendix Figure S1.** mRNA expression levels of *VHL*, *PHD1* and *PHD3*, which were HIF2α stabilization-related molecules, showed no difference in IPAH patient pericytes (n=4) compared to healthy lung pericytes (n=4). Paired t-test with a parametric test was used.

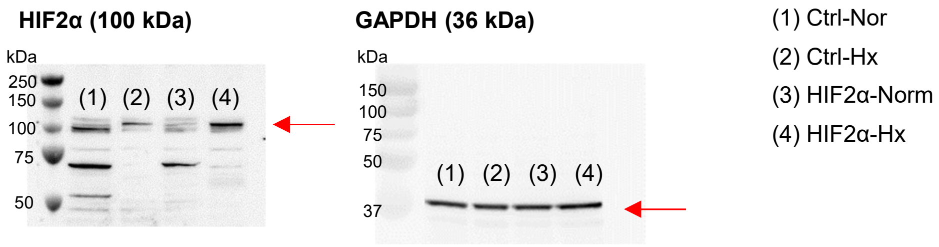


**Appendix Figure S2.** The protein expression levels of HIF2α were measured after human healthy pericytes were transfected with the *EPAS1* plasmid with or without hypoxia exposure by Western blot.

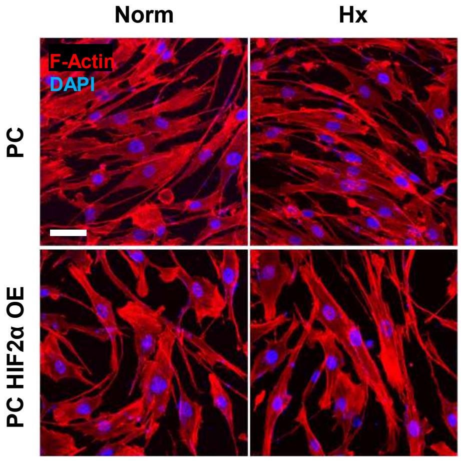


**Appendix Figure S3.** Normoxia (left) and hypoxia (right) of PC (top row) and PC $^{HIF2α\ OE}$ (bottom row) showed no significant difference in cell morphology and F-actin expression. Represented by F-actin (red) IF staining and nuclei were stained with DAPI (blue). Scale bar = 50 µm.

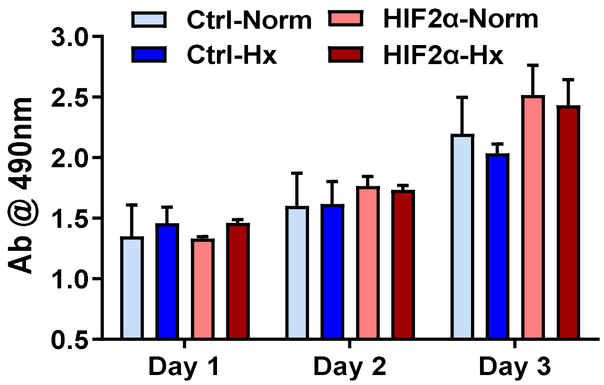


**Appendix Figure S4.** The proliferation rates of PC and PC$^{HIF2\alpha OE}$ over 3 days in normoxia or hypoxia (n=4) were measured by MTS assay. The statistical analysis was compared to the control norm on each day.

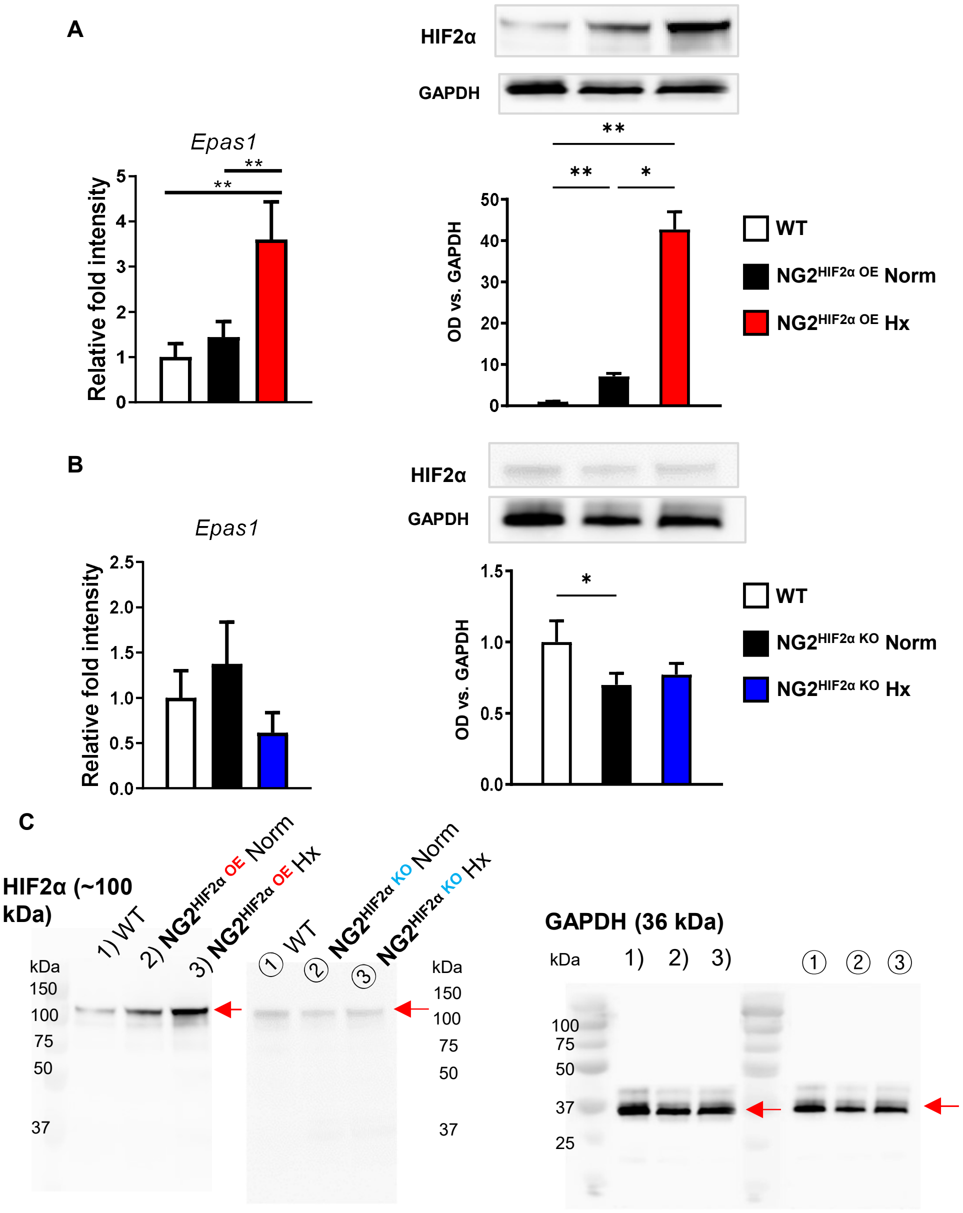


**Appendix Figure S5.** The mRNA expression levels of *Epas1* and the protein expression levels of HIF2α were measured in NG2$^{HIF2\alpha OE}$, NG2$^{HIF2\alpha KO}$ and WT animals using quantitative PCR and WB. (A) HIF2α was measured in NG2$^{HIF2\alpha OE}$ mice under normoxia and hypoxia. Left: mRNA levels of *Epas1* by qPCR; right: protein levels of HIF2α by WB. The ratio of band intensity using GAPDH as normalization was quantified. (B) The mRNA expression levels of *Epas1* and the protein expression level of HIF2α in NG2$^{HIF2\alpha KO}$ mice by normoxia and hypoxia were measured. (C) The protein expression levels of HIF2α and housekeeping GAPDH from NG2$^{HIF2\alpha OE}$ and NG2$^{HIF2\alpha KO}$ mouse whole lungs with/without hypoxia were measured by Western blot. Means ± SEM were derived from at least three biological replicates. *Depicts a statistically significant difference: **P<0.05* and ***P<0.01* (one-way ANOVA with Turkey's multiple comparisons test).

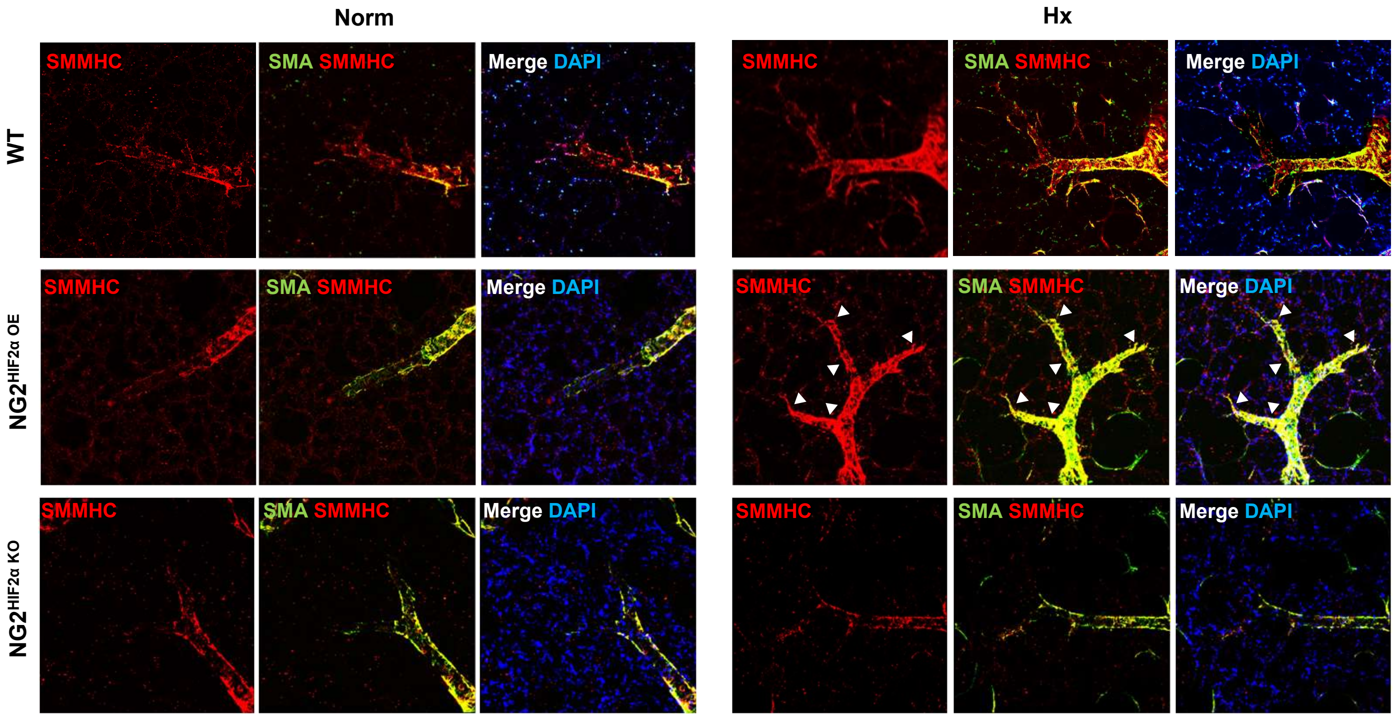


**Appendix Figure S6.** Precision cut lung slices of WT, NG2$^{HIF2α\ OE}$ and NG2$^{HIF2α\ KO}$ mice with normoxia vs hypoxia were stained for SMA (green) and SMMHC (red) to indicate the co-expression of SMA and SMMHC. Nuclei were stained with DAPI (blue). Scale bar = 50 µm.

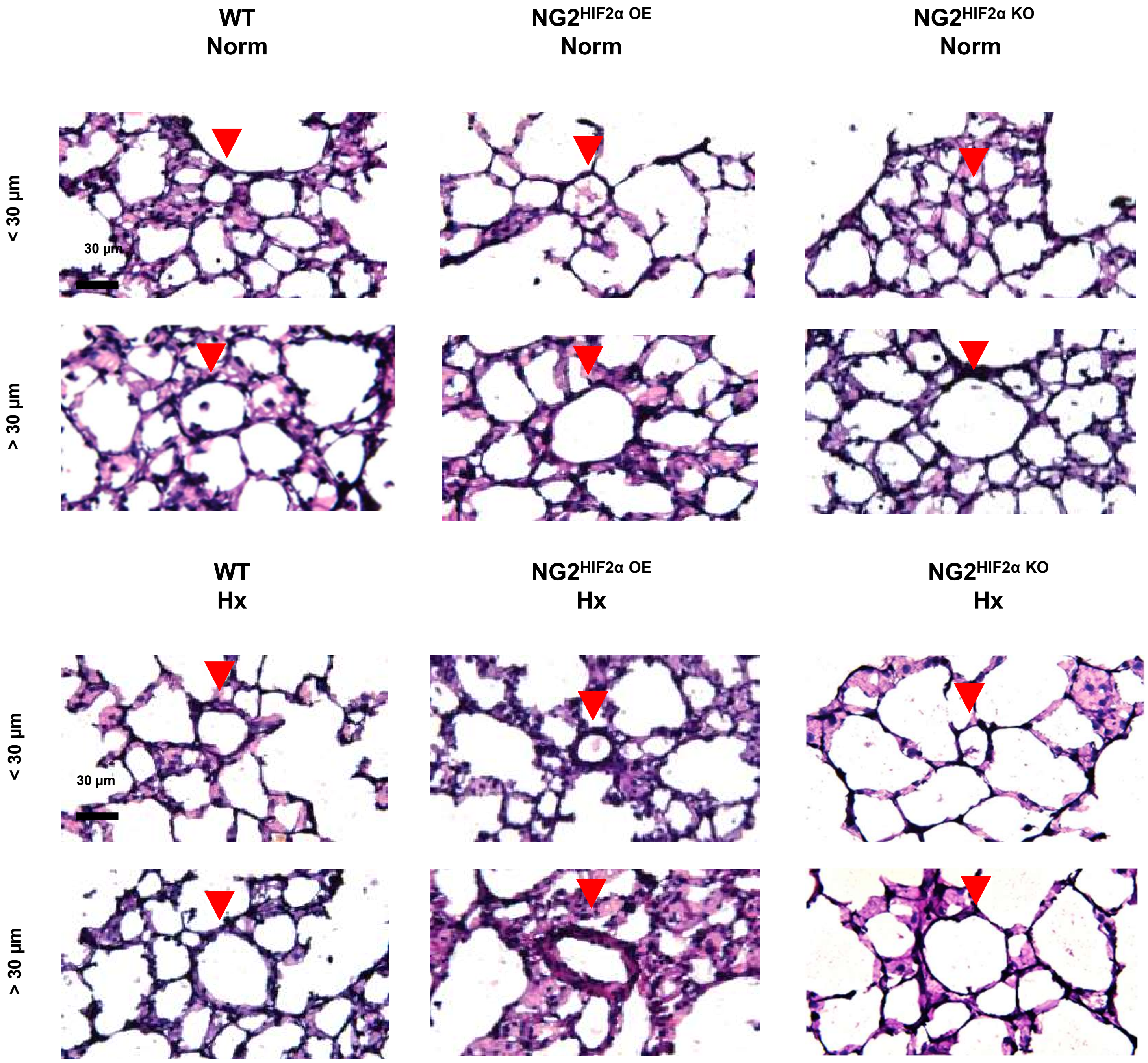


**Appendix Figure S7.** Vessel muscularization was quantified in WT, NG2$^{HIF2α\ OE}$ and NG2$^{HIF2α\ KO}$ with or without Hx. H&E staining represents the different sizes of vessels (smaller or larger than 30 µm in diameter) for each group. After 3wk Hx, NG2$^{HIF2α\ OE}$ vessels exhibited the largest wall thickness. Scale bar = 30 µm.

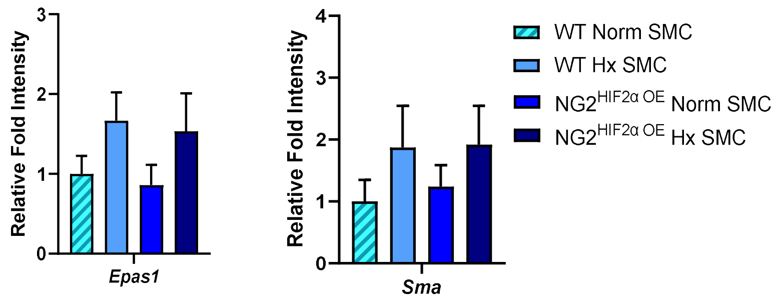


**Appendix Figure S8.** The mRNA expression levels of *Epas1* and *Sma* in isolated CD146+(SMC dominant) cells from WT and NG2$^{HIF2α\ OE}$ mice under normoxia or hypoxia (n=4) were measured.

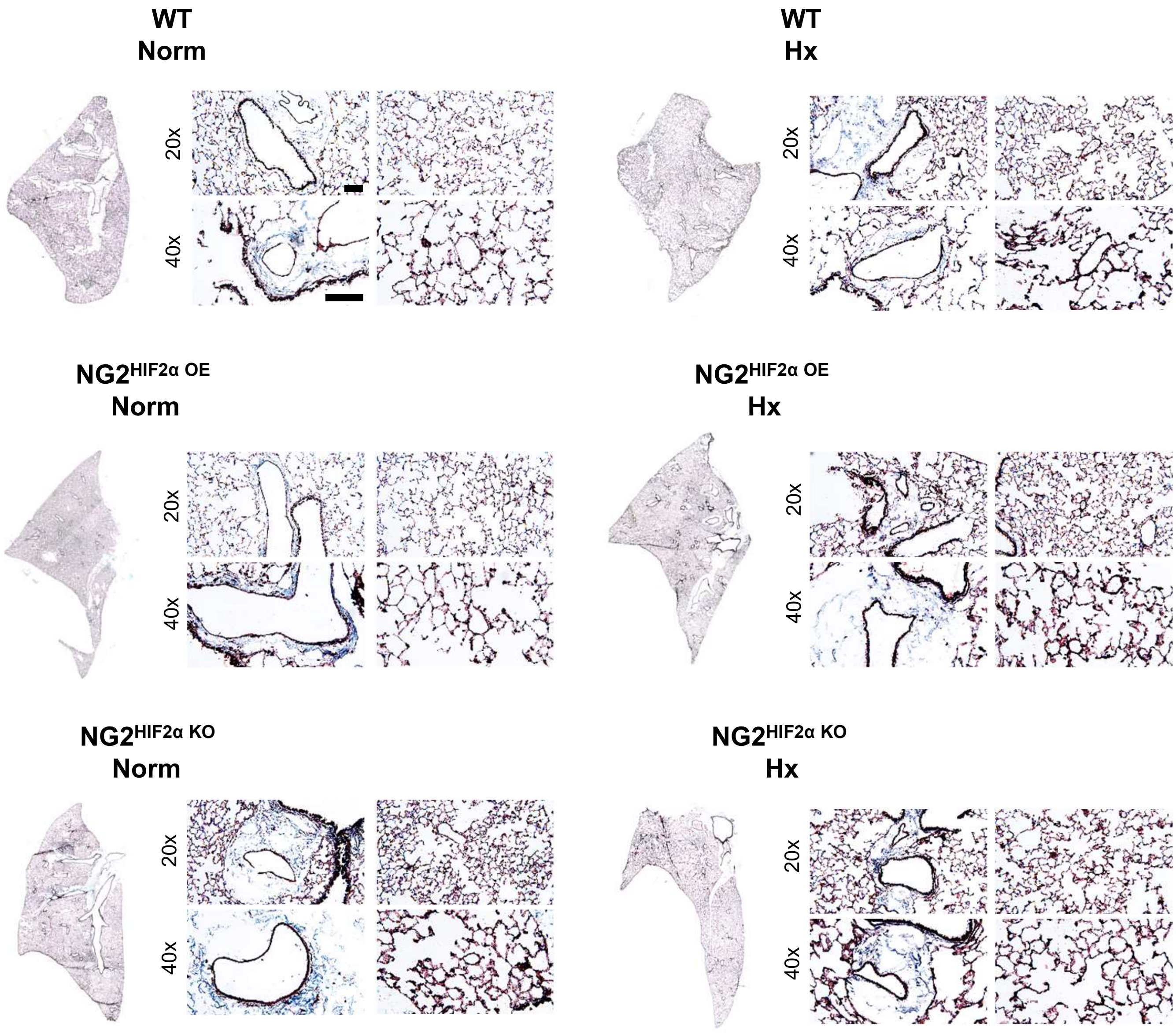


**Appendix Figure S9.** The collagen deposition of each condition was stained using Masson's Trichrome. No significant difference in collagen-blue intensity was found among WT, NG2$^{HIF2α\ OE}$, and NG2$^{HIF2α\ KO}$ under normoxia or 3wk hypoxia. Scale bar = 100 μm.

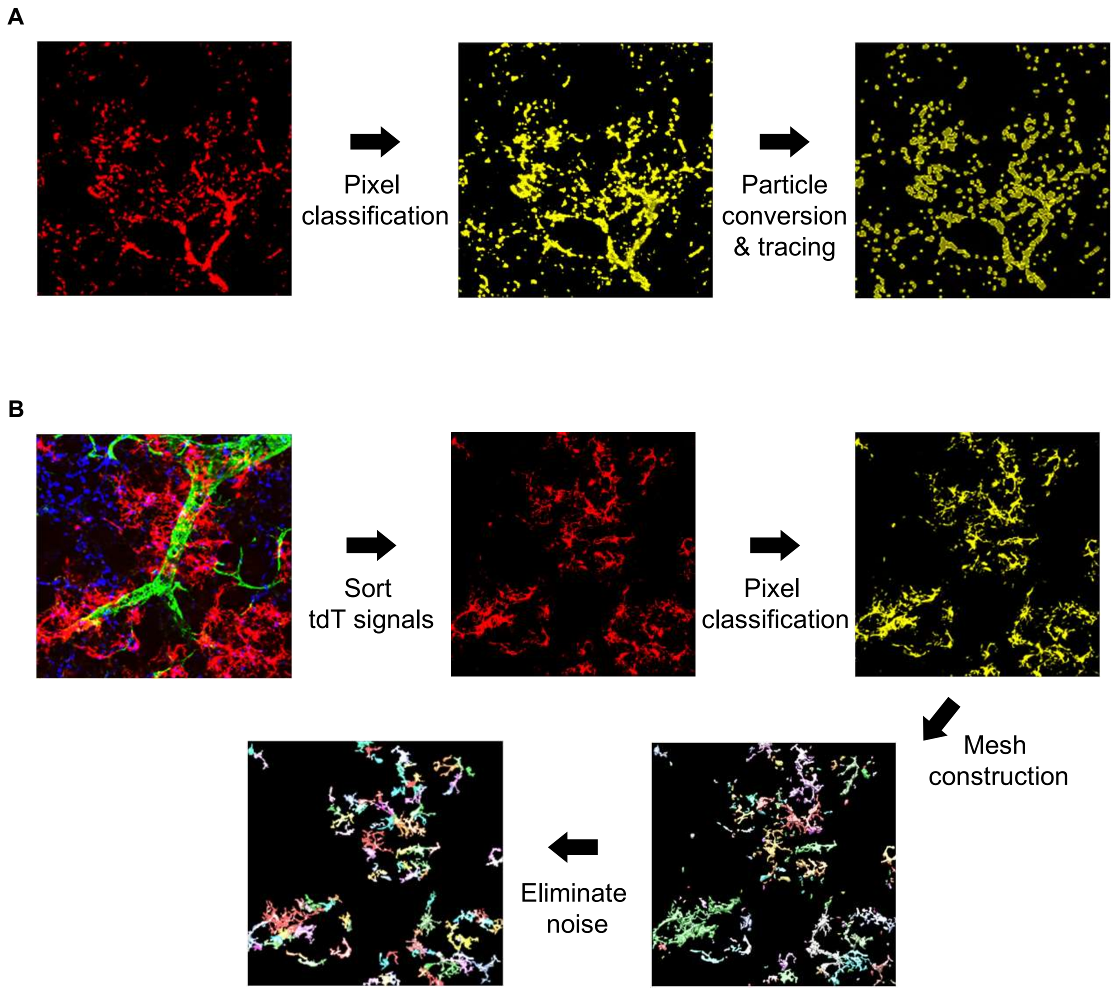


**Appendix Figure S10.** The detailed stepwise procedures of quantification methods are found in Fig 5 & 7. (A) The intensity of fluorescent microsphere beads was converted to pixel classification and then further converted to particle tracing using computational machine learning Aivia 3D function tools. (B) The tdT color intensity was translated to pixel classification and mesh construction using Aivia quantification tools. See details in the Material and Method section.

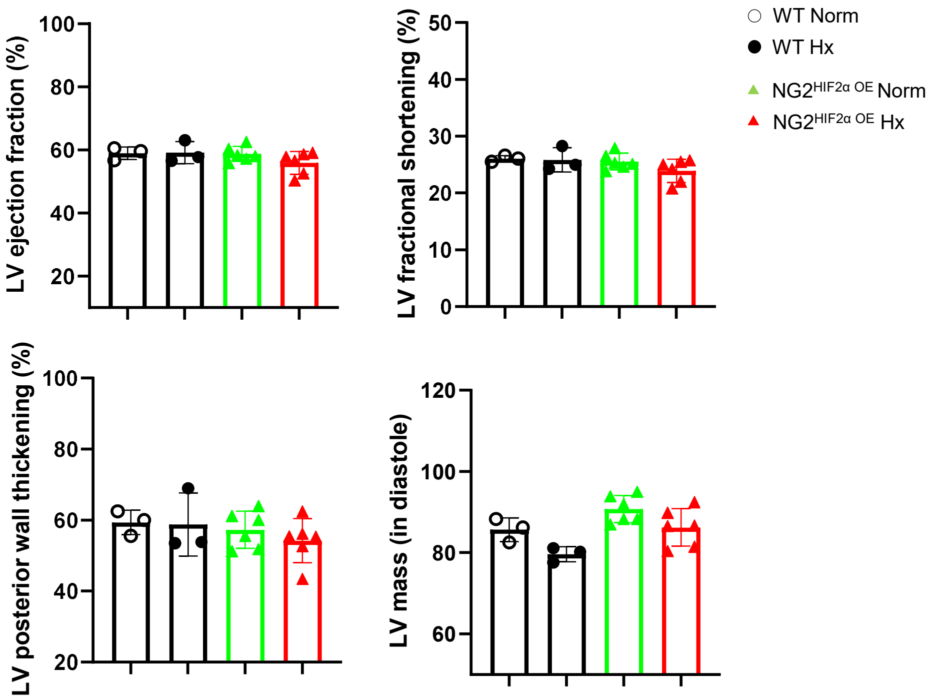


**Appendix Figure S11.** LV function and mass by echocardiography were measured in WT and NG2$^{HIF2\alpha\ OE}$ mice with or without 3wk Hx. There was no significant difference in LV ejection fraction, fractional shortening, posterior wall thickness, and mass.

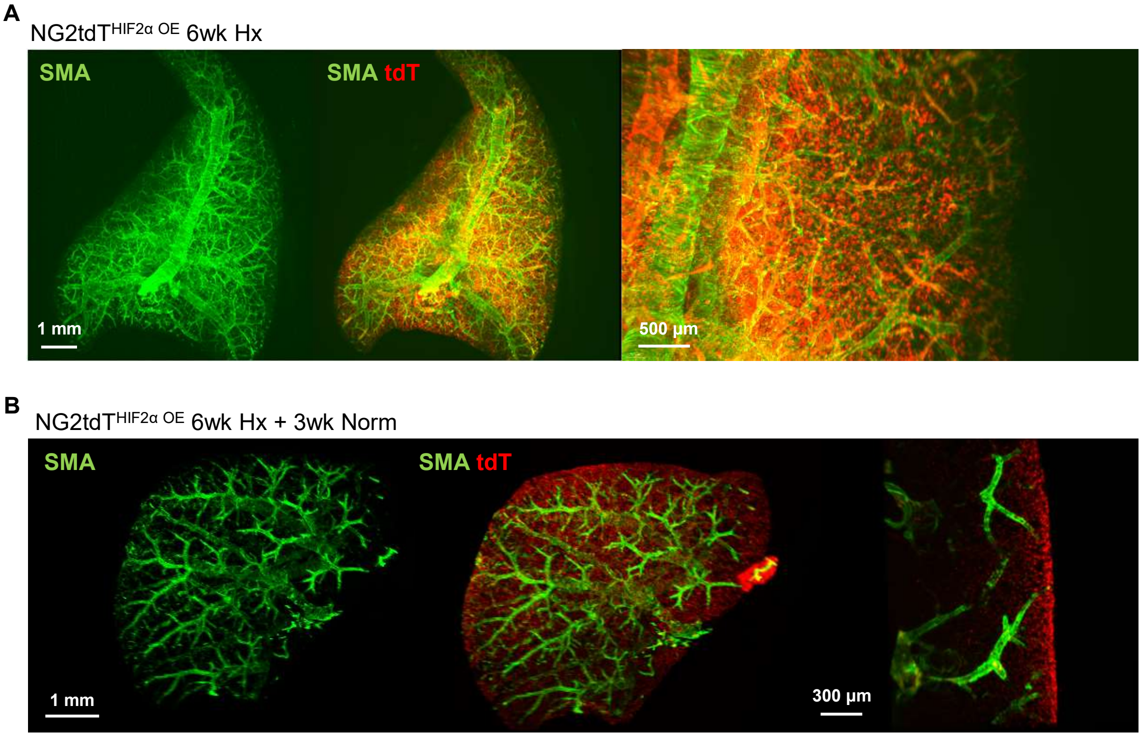


**Appendix Figure S12.** Representative light-sheet microscopic images from iDISCO-cleared whole lung lobes show 3D views of SMA-labeled vasculature from NG2tdT$^{HIF2\alpha\ OE}$ mice. (A) Whole lung lobes of mice exposed to 6wk Hx had increased muscularization of distal vessels. (B) Whole lung lobes of mice exposed to 6wk Hx and then 3wk normoxia, as a recovery model, had decreased vessel muscularization.

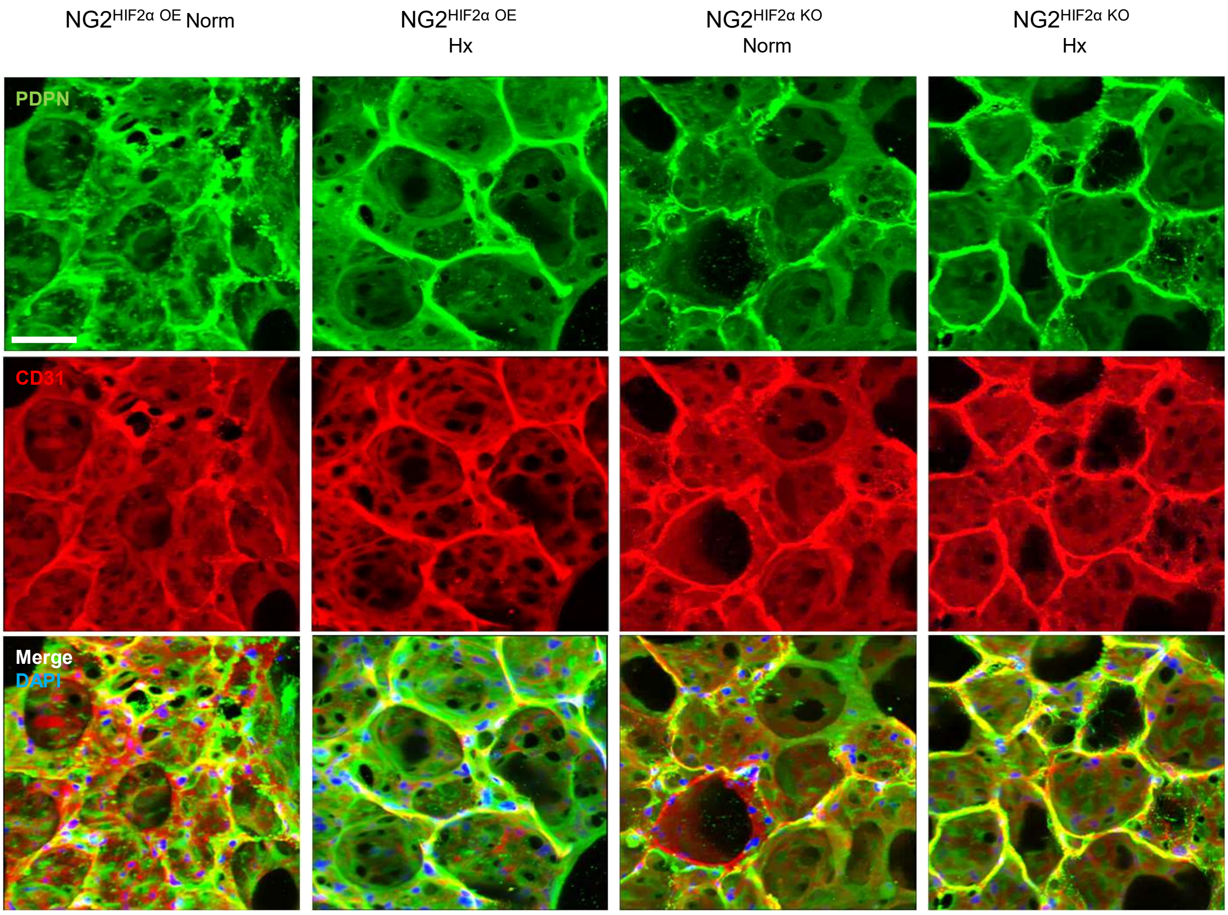


**Appendix Figure S13.** Alveolar type I epithelial cells (ATI) of both NG2$^{HIF2\alpha\ OE}$ and NG2$^{HIF2\alpha\ KO}$ murine line under normoxia or 3wk Hx had no structural change. The precision cut lung slices were stained for Podoplanin (PDPN, green) for ATI, CD31 (red) for endothelium, and DAPI (blue). Scale bar = 50 μm.

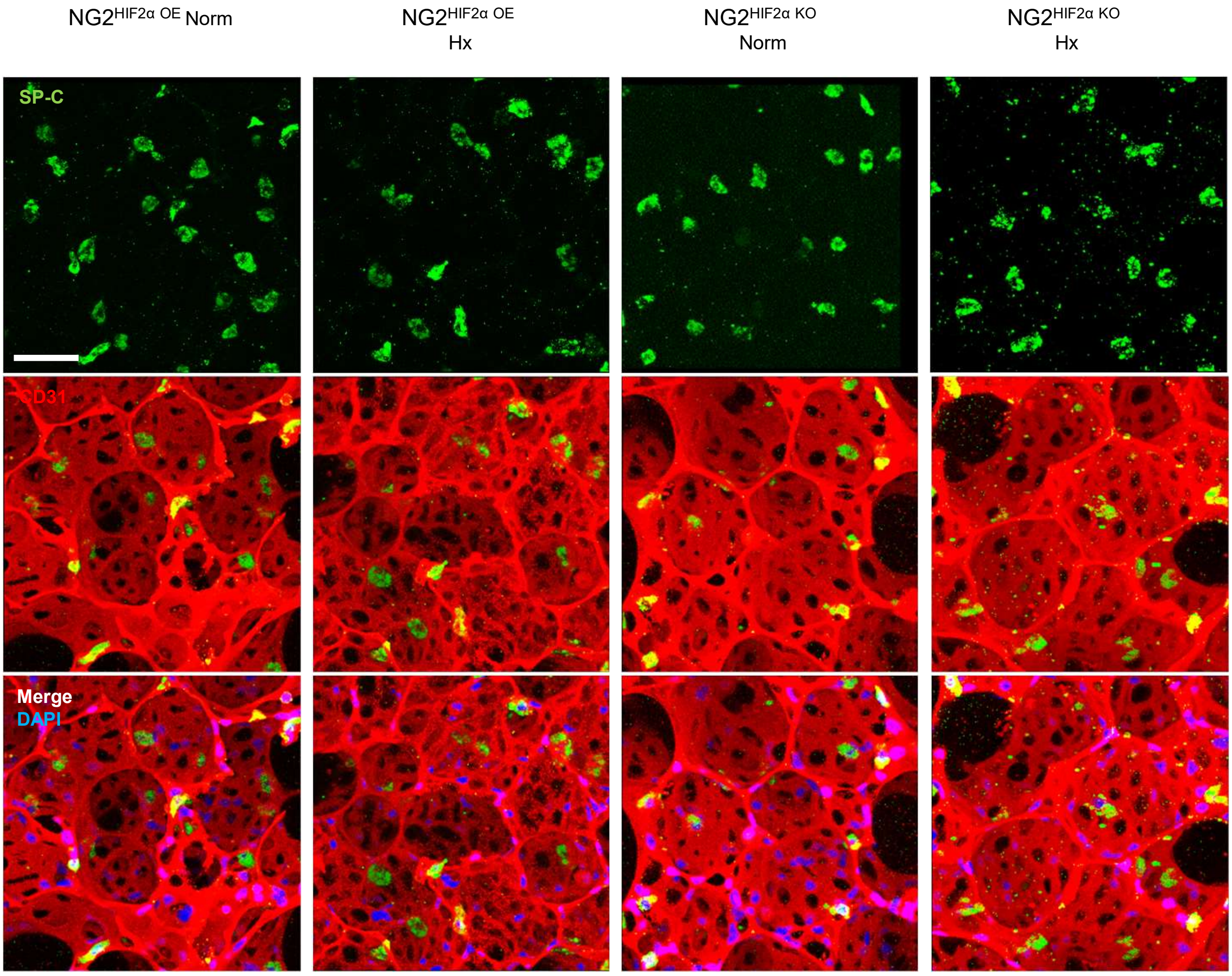


**Appendix Figure S14.** Alveolar type II epithelial cells (AT2) of both NG2$^{HIF2\alpha\ OE}$ and NG2$^{HIF2\alpha\ KO}$ murine line under normoxia or 3wk Hx had no structure change. The precision cut lung slices were stained for surfactant protein-C (SP-C, green) for AT2, CD31 (red) for endothelium, and DAPI (blue). Scale bar = 50 μm.

| # | Gene name | Protein name | Fold Change | p_val |
|---|---|---|---|---|
| 1 | **THY1** | **Thy-1 membrane glycoprotein** | 3.33 | 0.000234 |
| 2 | IGFBP2 | Insulin-like growth factor-binding protein 2 | 2.96 | 0.000309 |
| 3 | LGALS3BP | Galectin-3-binding protein | 2.34 | 4.61E-05 |
| 4 | COL1A2 | Collagen alpha-2(I) chain | 2.28 | 0.009641 |
| 5 | **FAM162B** | **Family with sequence similarity 162 member B** | 2.27 | 0.014575 |
| 6 | **PTN** | **Pleiotrophin** | 2.25 | 3.59E-05 |
| 7 | GADD45A | Growth arrest and DNA damage-inducible protein GADD45 alpha | 2.23 | 0.000294 |
| 8 | ENG | Endoglin | 2.22 | 0.000235 |
| 9 | ITM2C | Integral membrane protein 2C | 2.15 | 8.3E-07 |
| 10 | COL18A1 | Collagen alpha-1(XVIII) chain | 2.14 | 2.35E-05 |
| 11 | TGFBI | Transforming growth factor-beta-induced protein ig-h3 | 2.12 | 0.002178 |
| 12 | CD9 | CD9 antigen | 2.09 | 0.005832 |
| 13 | ID3 | DNA-binding protein inhibitor ID-3 | 2.09 | 9.41E-05 |
| 14 | IGFBP5 | Insulin-like growth factor-binding protein 5 | 2.07 | 0.040323 |
| 15 | ARHGDIB | Rho GDP-dissociation inhibitor 2 | 2.03 | 0.007274 |
| 16 | WFDC1 | WAP four-disulfide core domain protein 1 | 2.01 | 2.84E-05 |
| 17 | **RGS5** | **Regulator of G-protein signaling 5** | 2.00 | 0.04991 |
| 18 | CRIP2 | Cysteine rich protein 2 | 1.98 | 5.99E-06 |
| 19 | HCFC1R1 | Host cell factor C1 regulator 1 | 1.95 | 7.72E-05 |
| 20 | TPPP3 | Tubulin polymerization-promoting protein family member 3 | 1.94 | 0.000263 |

**Appendix Table S1.** DEG of Sub-cluster 5 from clusters human IPAH *CSPG4+PDGFRβ+* pericytes vs Control *CSPG4+PDGFRβ+* pericytes identified from mural cell clusters 14, 16 and 25. Mature smooth muscle cell marker *THY-1* was significantly upregulated.

| | Patient # | Age, yr | Sex | Etiology | 6MWD (m) | Therapies | Hemodynamics | |
|---|---|---|---|---|---|---|---|---|
| | | | | | | | mPAP,mmHg | PVR,WU |
| IPAH | 1 | 40 | M | IPAH | 420 | sildenafil, ambrisentan, treprostinil | 64 | 16.77 |
| | 2 | 51 | M | IPAH | 378 | sildenafil, epoprostenol | 30 | 6.09 |
| | 3 | 14 | F | IPAH | 440.1 | sildenafil, tadalafil, ambrisentan, treprostinil, epoprostenol, bosentan | 86 | n/a |
| | 4 | 62 | F | IPAH | 259.4 | epoprostenol, ambrisentan, bosentan | 47 | 6.17 |
| | 5 | 7 | F | IPAH | 422.5 | Sildenafil, ambrisentan, bosentan, iloprost, treprostinil | 88 | 17.62 |

| | Patient # | Age, yr | Sex | Cause of Death |
|---|---|---|---|---|
| Donor control | 1 | 52 | F | Hypoxic brain death secondary to PEA arrest |
| | 2 | 11 | M | Anoxia - Declared Brain Dead - Natural Causes (Hanging) |
| | 3 | 25 | M | Head Trauma/Intracranial Hemorrhage/Motor Vehicle Collision |
| | 4 | 26 | M | Gunshot wound to the head |
| | 5 | 36 | F | Subarachnoid hemorrhage |

**Appendix Table S2.** Clinical characteristics of patient samples were used in the study. F, female; M, male; 6MWD, 6-minute walking distance; IPAH, idiopathic pulmonary arterial hypertension; mPAP, mean pulmonary arterial pressure; PVR, pulmonary vascular resistance; WU, Wood units; PEA, pulseless electrical activity.
