# Supplementary figures and images for: Pericytes contribute to pulmonary vascular remodeling via HIF2α signaling (part 1 of 2)
Source: EMBO Rep. 2024 Jan 19;25(2):13. doi: 10.1038/s44319-023-00054-w (PMC10897382; doi:10.1038/s44319-023-00054-w)

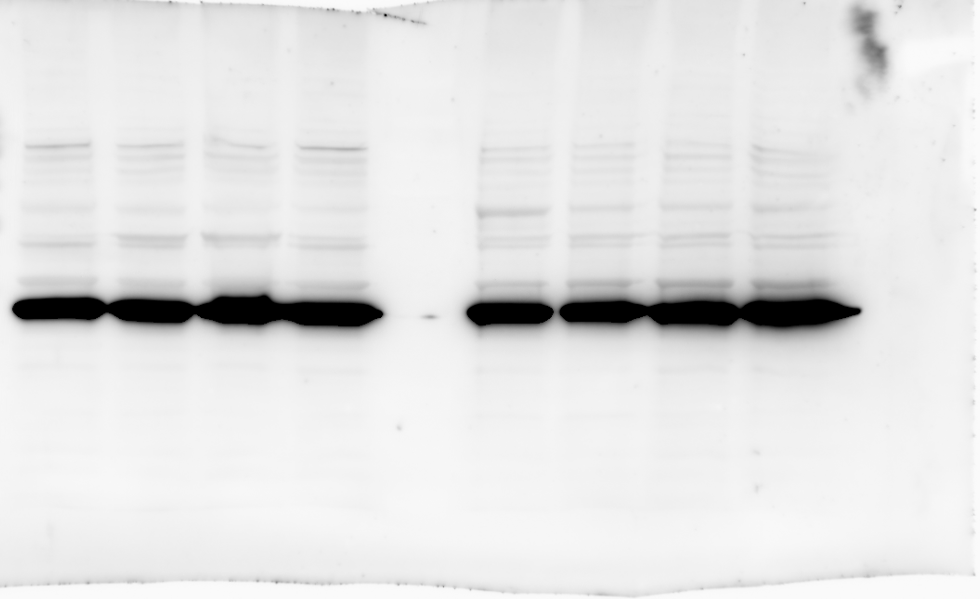

Supplement: Supplementary file 10 — Source Data Fig. 1 [file 44319_2023_54_MOESM10_ESM.zip › Figure 1/1G/Fig 1G-WB-control.tif]

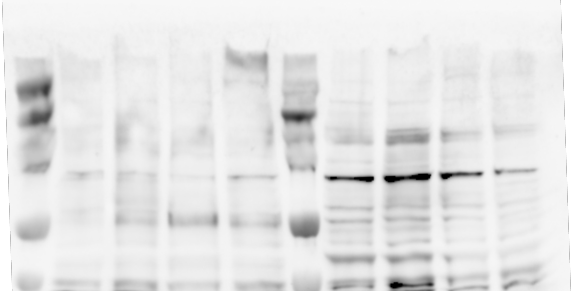

Supplement: Supplementary file 10 — Source Data Fig. 1 [file 44319_2023_54_MOESM10_ESM.zip › Figure 1/1G/Fig 1G-WB.tif]

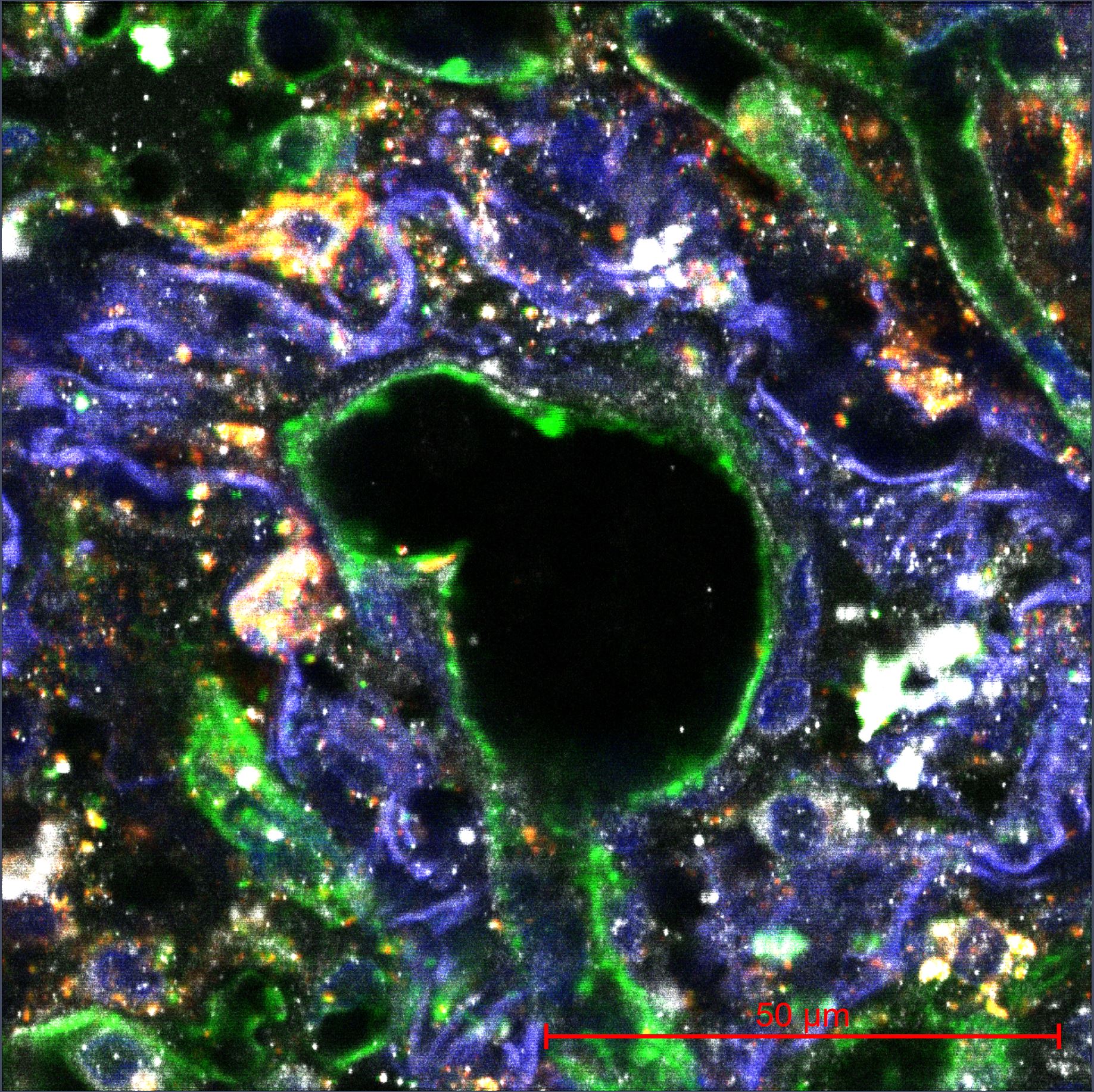

Supplement: Supplementary file 10 — Source Data Fig. 1 [file 44319_2023_54_MOESM10_ESM.zip › Figure 1/1H/Fig 1H IPAH all.JPG]

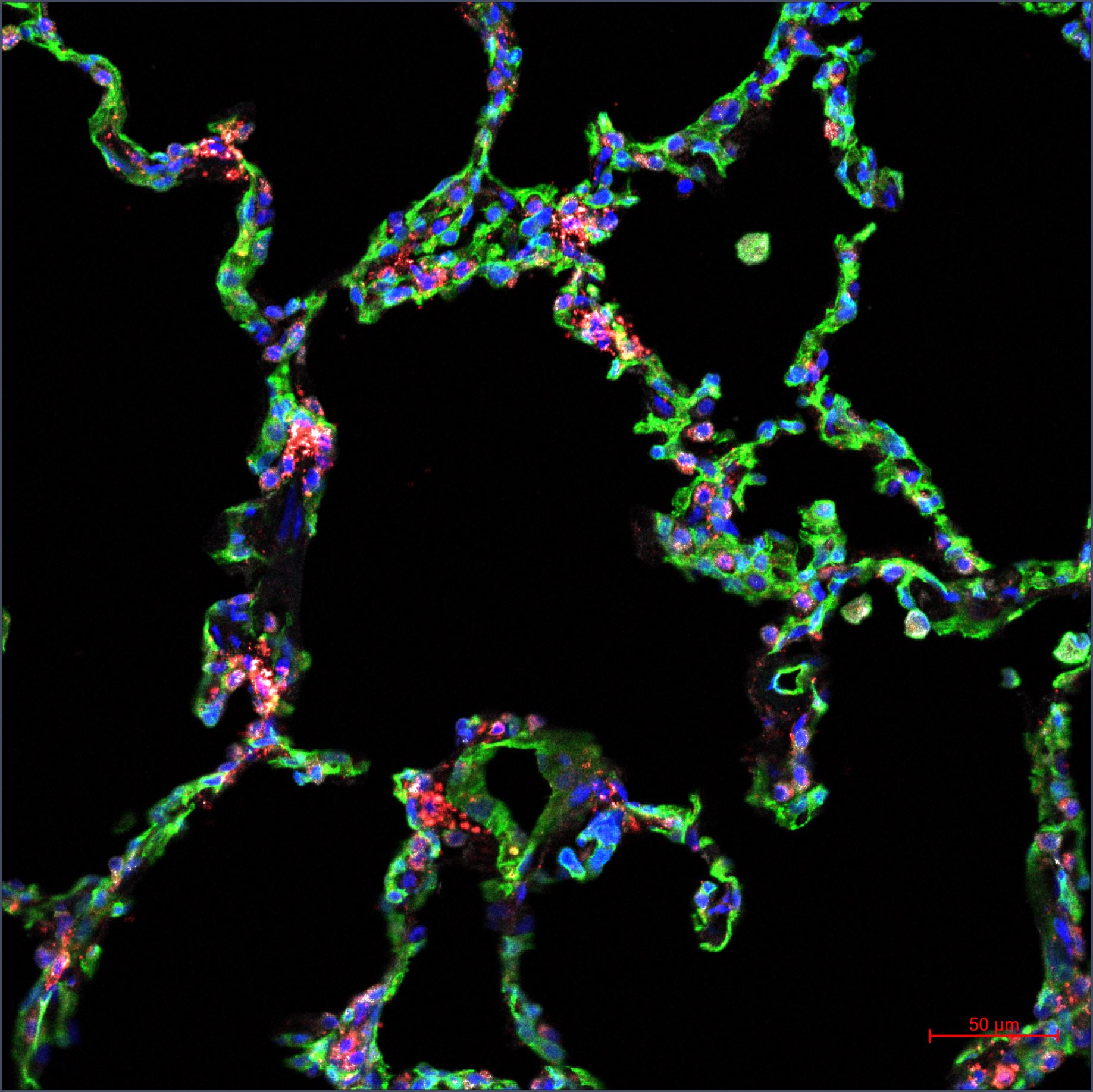

Supplement: Supplementary file 10 — Source Data Fig. 1 [file 44319_2023_54_MOESM10_ESM.zip › Figure 1/1H/Fig 1H-control-all.JPG]

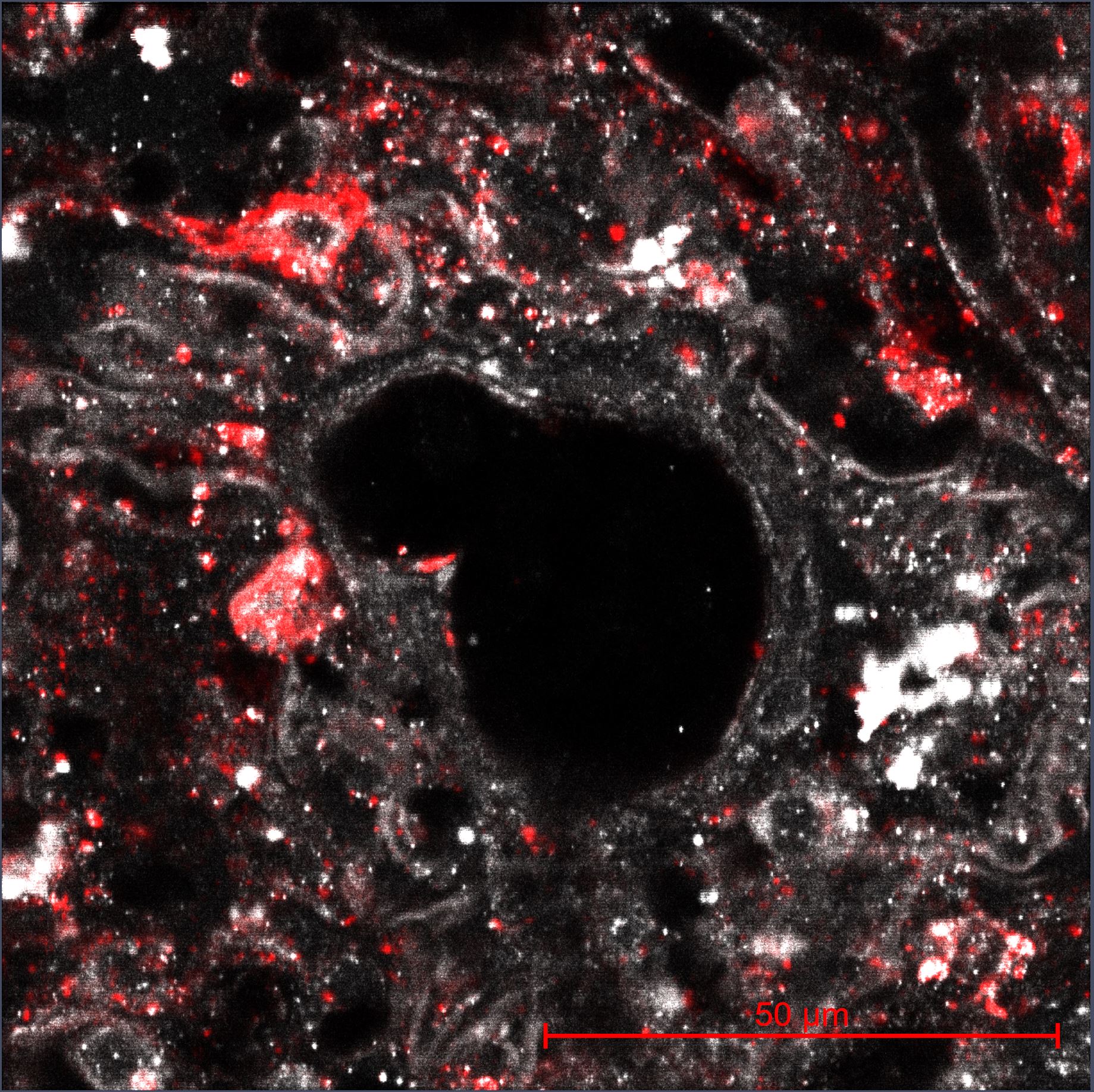

Supplement: Supplementary file 10 — Source Data Fig. 1 [file 44319_2023_54_MOESM10_ESM.zip › Figure 1/1H/Fig 1H-IPAH-3g5 hif2a.JPG]

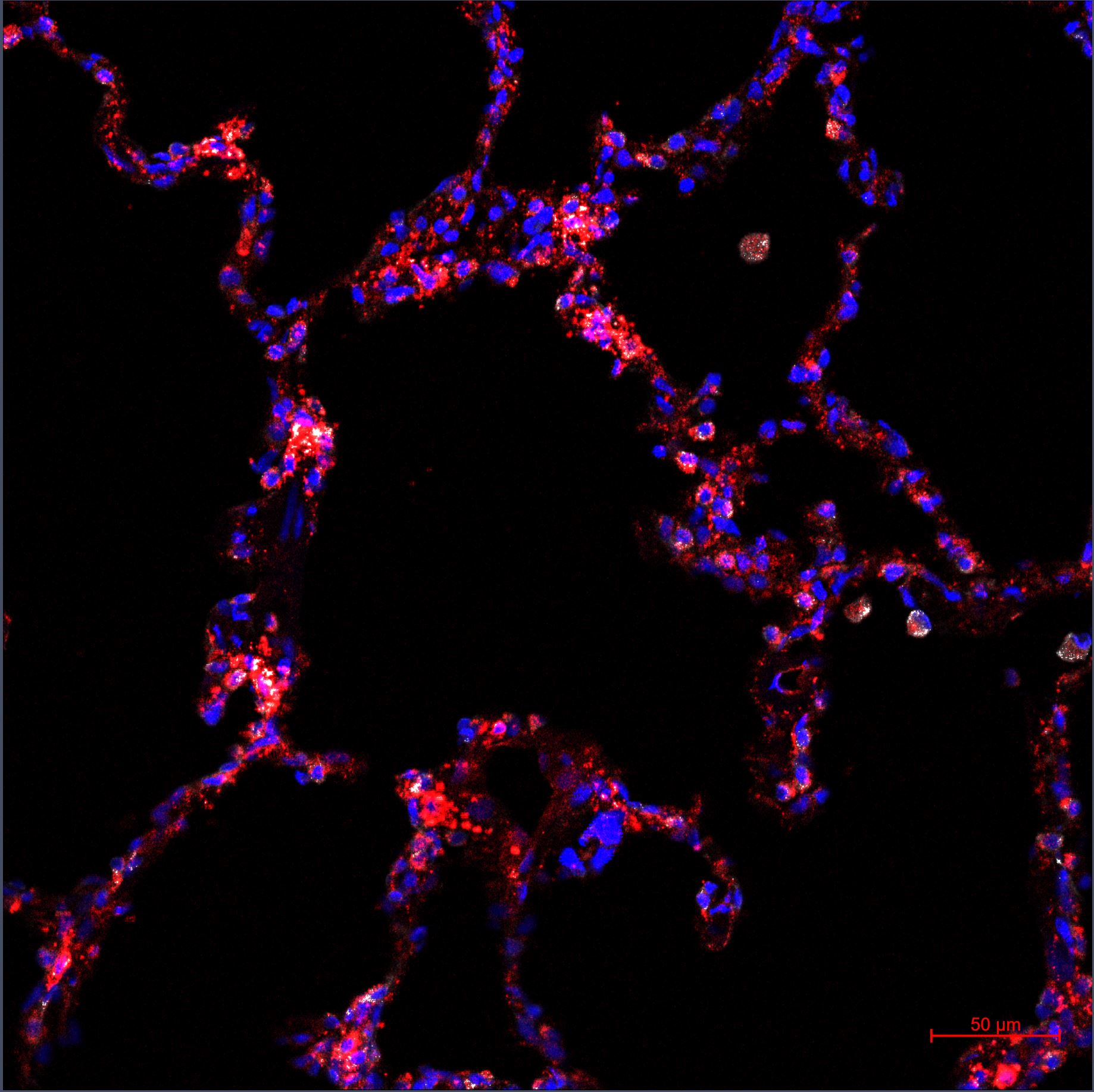

Supplement: Supplementary file 10 — Source Data Fig. 1 [file 44319_2023_54_MOESM10_ESM.zip › Figure 1/1H/Fig1H-control-3g5 hif2a.JPG]

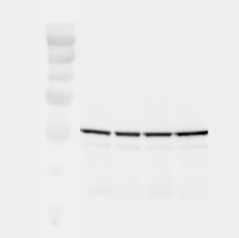

Supplement: Supplementary file 11 — Source Data Fig. 2 [file 44319_2023_54_MOESM11_ESM.zip › Figure 2/2A/GAPDH.tif]

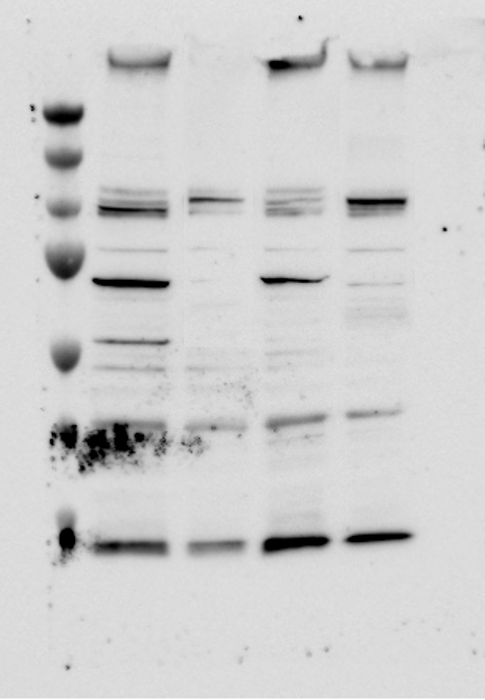

Supplement: Supplementary file 11 — Source Data Fig. 2 [file 44319_2023_54_MOESM11_ESM.zip › Figure 2/2A/HIF2a.tif]

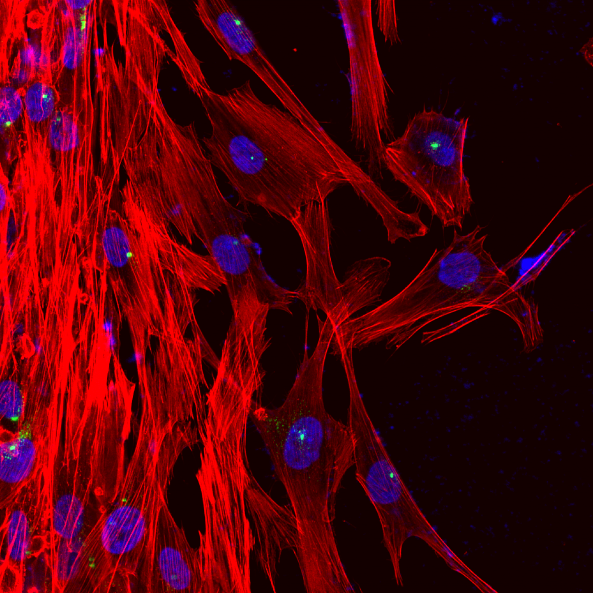

Supplement: Supplementary file 11 — Source Data Fig. 2 [file 44319_2023_54_MOESM11_ESM.zip › Figure 2/2B/1_PC_Norm.bmp]

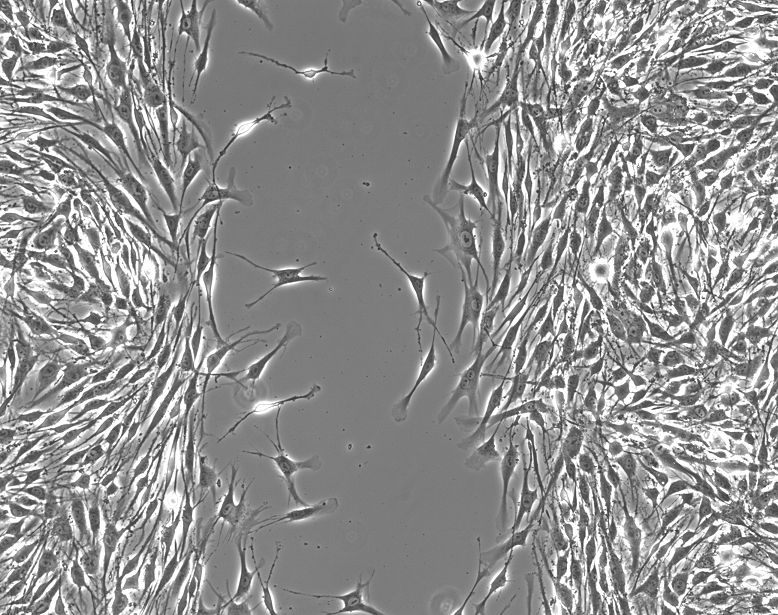

Supplement: Supplementary file 11 — Source Data Fig. 2 [file 44319_2023_54_MOESM11_ESM.zip › Figure 2/2B/1_PC_Norm.tif]

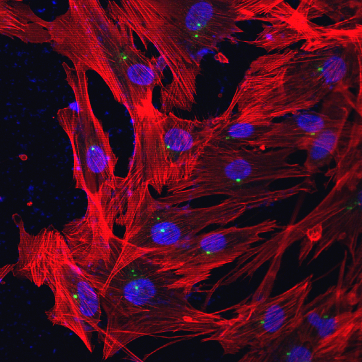

Supplement: Supplementary file 11 — Source Data Fig. 2 [file 44319_2023_54_MOESM11_ESM.zip › Figure 2/2B/2_PC_Hx.bmp]

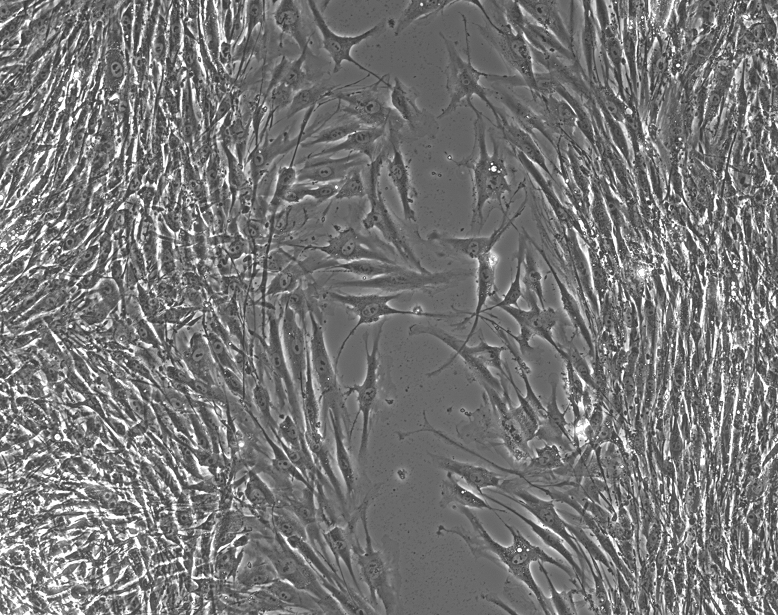

Supplement: Supplementary file 11 — Source Data Fig. 2 [file 44319_2023_54_MOESM11_ESM.zip › Figure 2/2B/2_PC_Hx.tif]

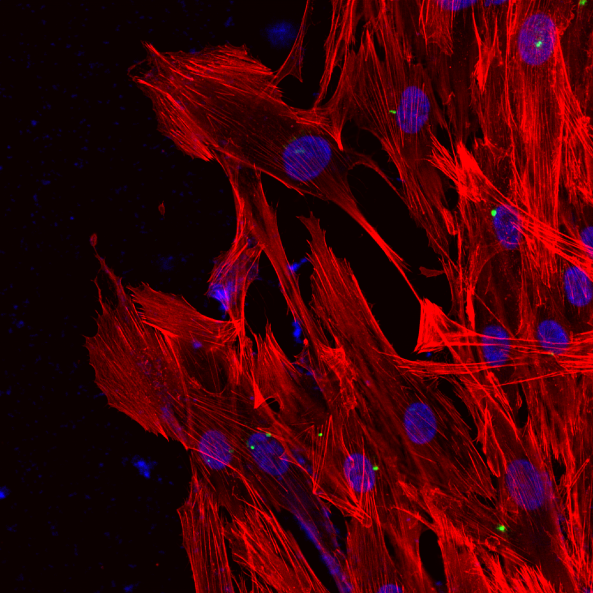

Supplement: Supplementary file 11 — Source Data Fig. 2 [file 44319_2023_54_MOESM11_ESM.zip › Figure 2/2B/3_PC HIF2a OE_Norm.bmp]

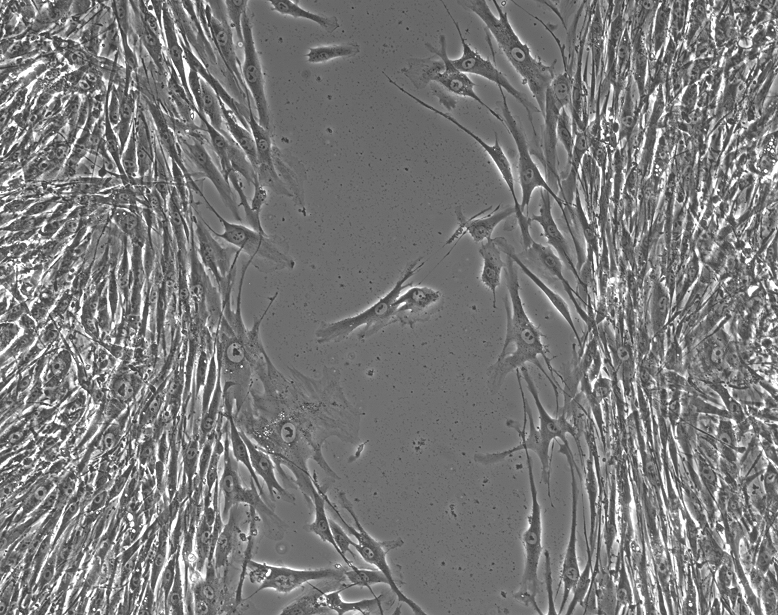

Supplement: Supplementary file 11 — Source Data Fig. 2 [file 44319_2023_54_MOESM11_ESM.zip › Figure 2/2B/3_PC HIF2a OE_Norm.tif]

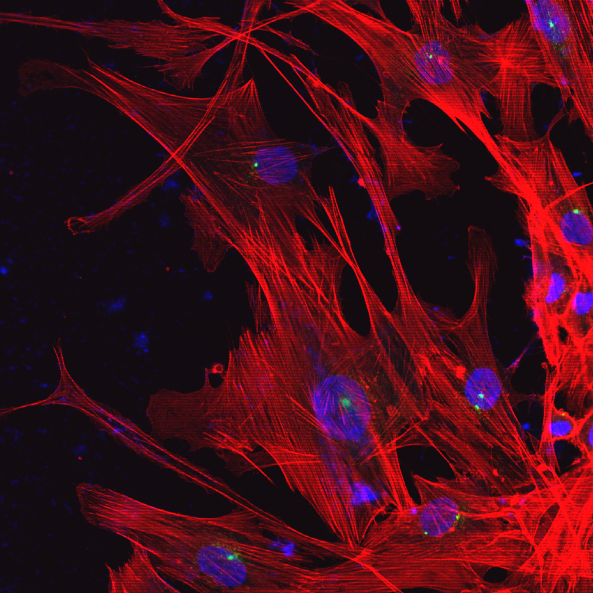

Supplement: Supplementary file 11 — Source Data Fig. 2 [file 44319_2023_54_MOESM11_ESM.zip › Figure 2/2B/4_PC HIF2a OE_Hx_.bmp]

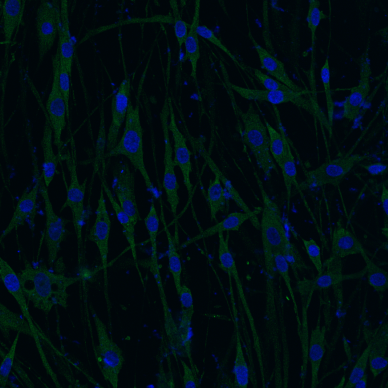

Supplement: Supplementary file 11 — Source Data Fig. 2 [file 44319_2023_54_MOESM11_ESM.zip › Figure 2/2C/1_PC_Norm_HIF2a-Green_DAPI-Blue.bmp]

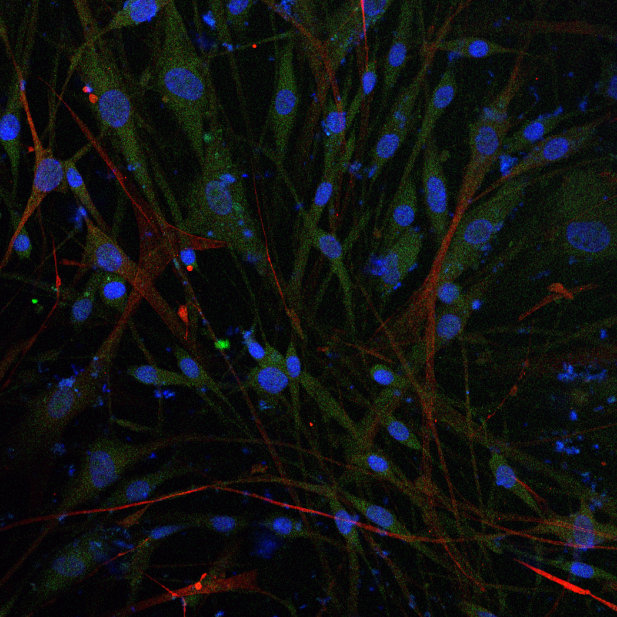

Supplement: Supplementary file 11 — Source Data Fig. 2 [file 44319_2023_54_MOESM11_ESM.zip › Figure 2/2C/1_PC_Norm_SM22-Green_Calponin-Red_DAPI-Blue.bmp]

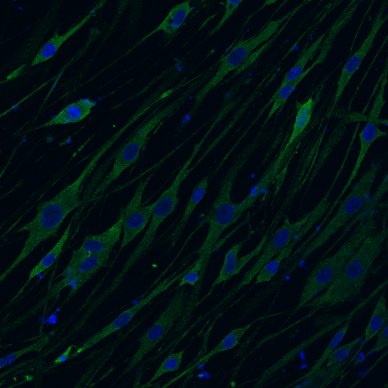

Supplement: Supplementary file 11 — Source Data Fig. 2 [file 44319_2023_54_MOESM11_ESM.zip › Figure 2/2C/2_PC HIF2a_Norm_HIF2a-Green_DAPI-Blue.bmp]

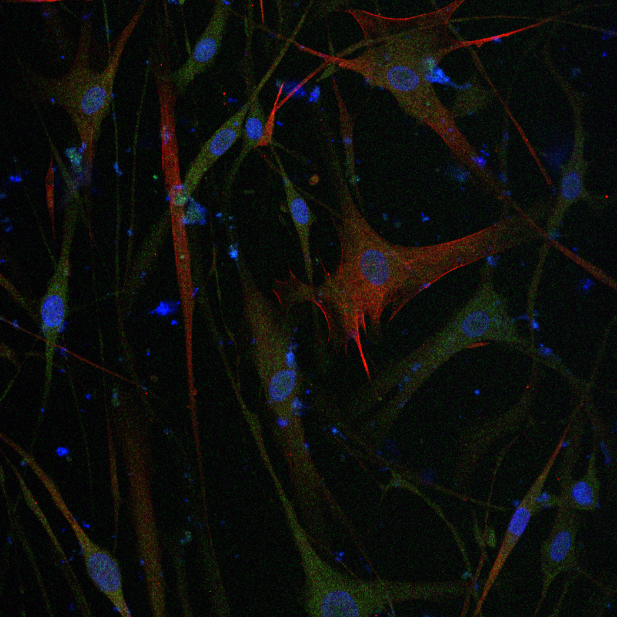

Supplement: Supplementary file 11 — Source Data Fig. 2 [file 44319_2023_54_MOESM11_ESM.zip › Figure 2/2C/2_PC HIF2a_Norm_SM22-Green_Calponin-Red_DAPI-Blue.bmp]

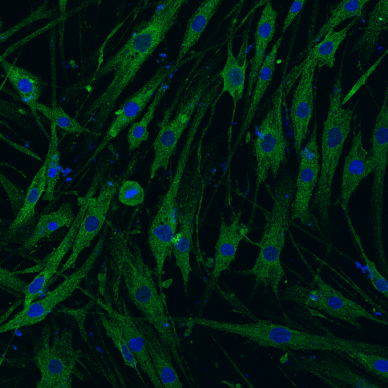

Supplement: Supplementary file 11 — Source Data Fig. 2 [file 44319_2023_54_MOESM11_ESM.zip › Figure 2/2C/3_PC_Hx_HIF2a-Green_DAPI-Blue.bmp]

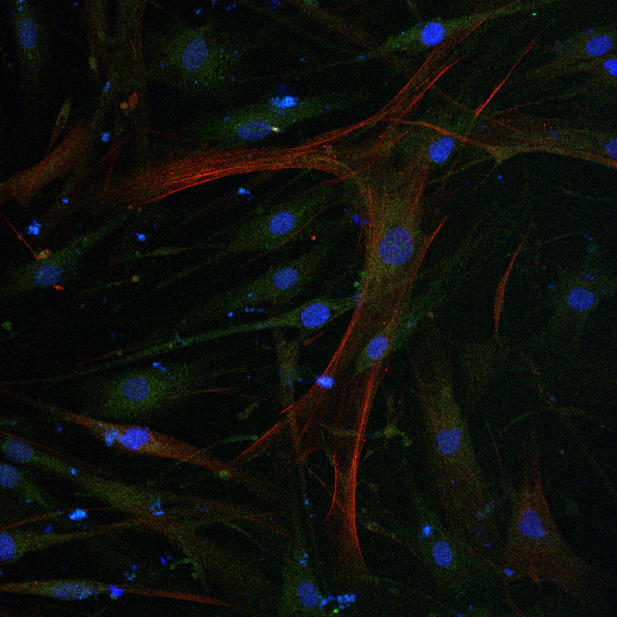

Supplement: Supplementary file 11 — Source Data Fig. 2 [file 44319_2023_54_MOESM11_ESM.zip › Figure 2/2C/3_PC_Hx_SM22-Green_Calponin-Red_DAPI-Blue.bmp]

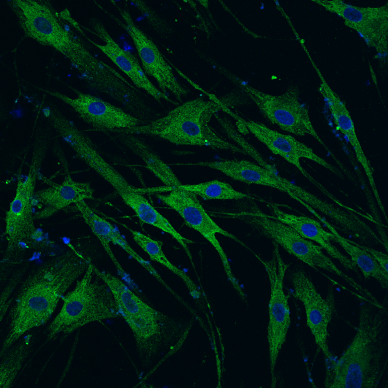

Supplement: Supplementary file 11 — Source Data Fig. 2 [file 44319_2023_54_MOESM11_ESM.zip › Figure 2/2C/4_PC HIF2a_Hx_HIF2a-Green_DAPI-Blue.bmp]

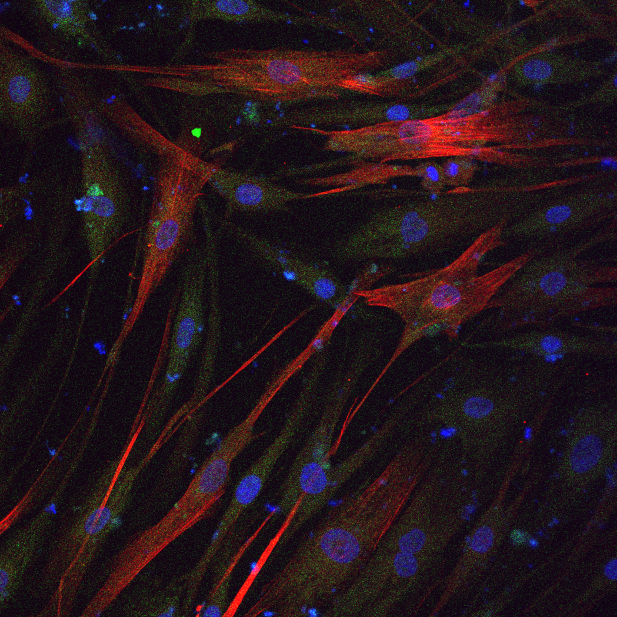

Supplement: Supplementary file 11 — Source Data Fig. 2 [file 44319_2023_54_MOESM11_ESM.zip › Figure 2/2C/4_PC HIF2a_Hx_SM22-Green_Calponin-Red_DAPI-Blue.bmp]

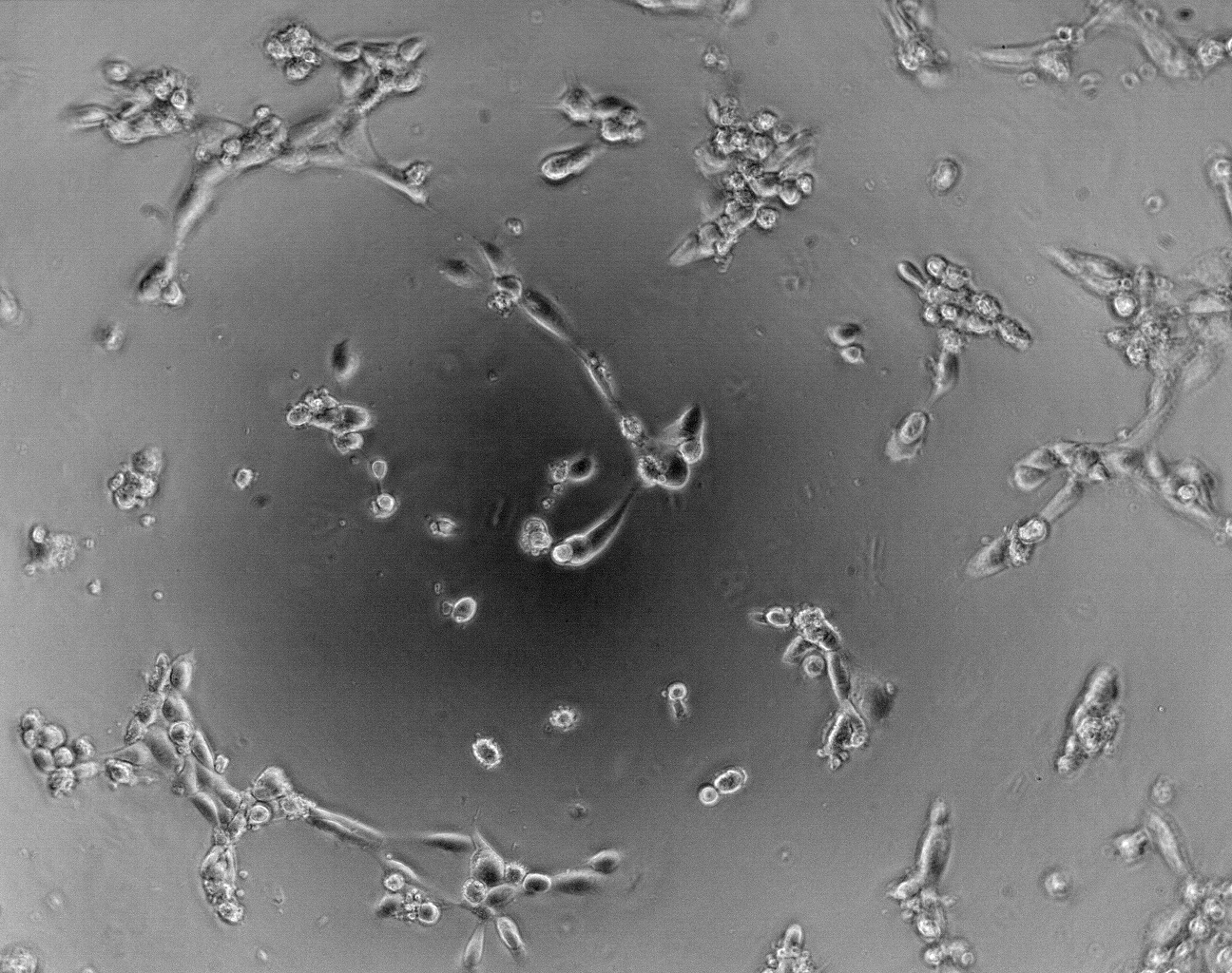

Supplement: Supplementary file 11 — Source Data Fig. 2 [file 44319_2023_54_MOESM11_ESM.zip › Figure 2/2D/1_EC only_BF.tif]

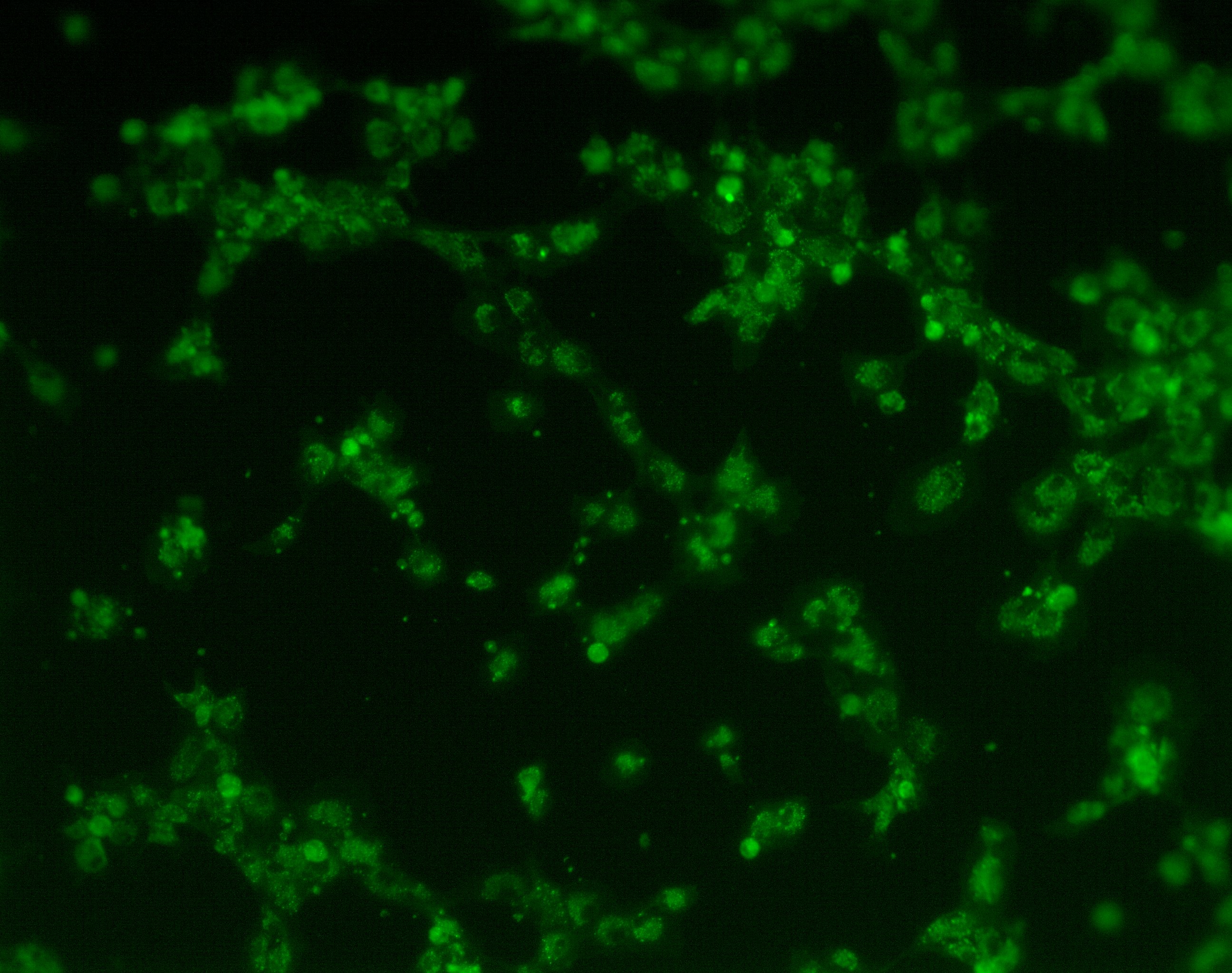

Supplement: Supplementary file 11 — Source Data Fig. 2 [file 44319_2023_54_MOESM11_ESM.zip › Figure 2/2D/1_EC only_RG.jpg]

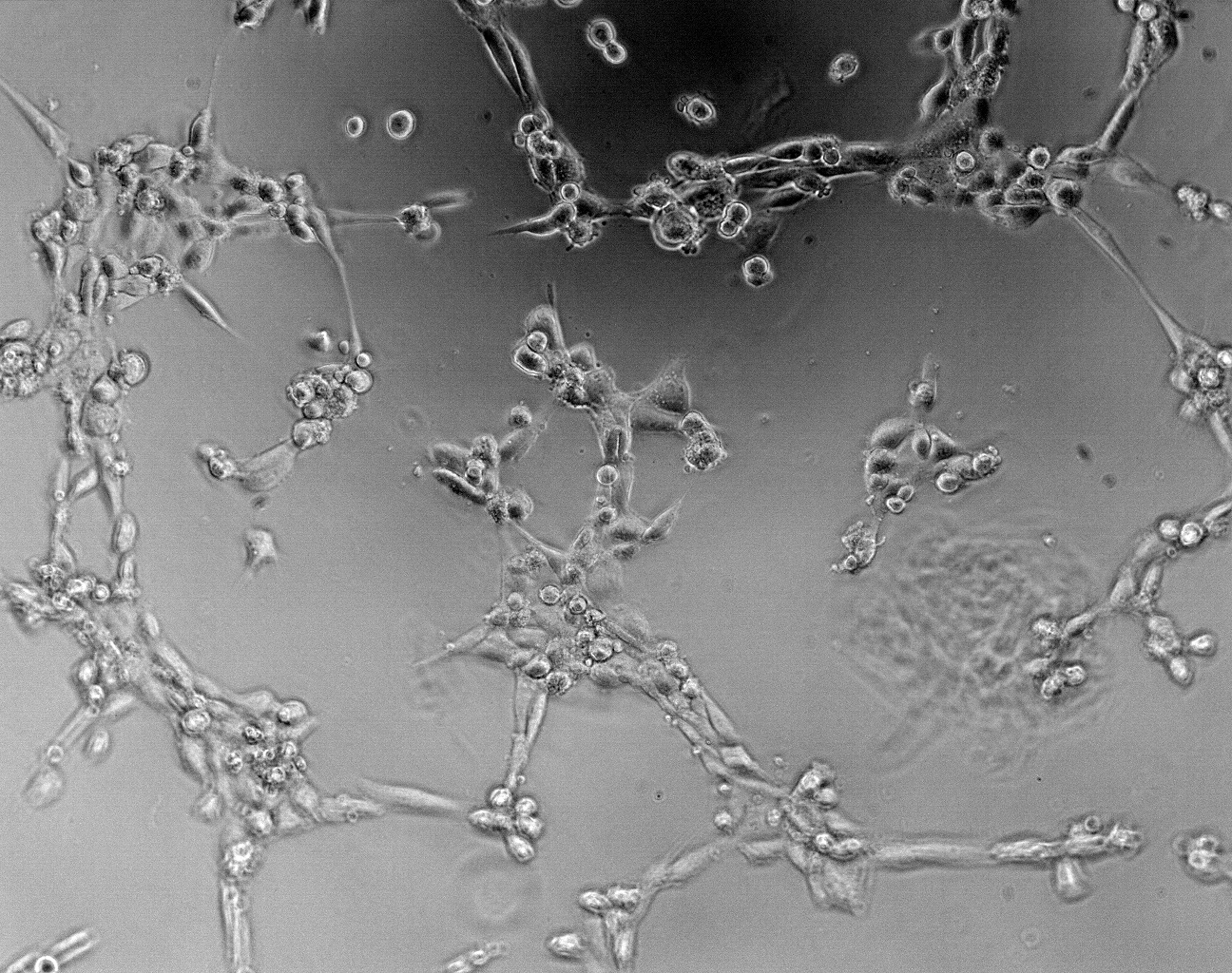

Supplement: Supplementary file 11 — Source Data Fig. 2 [file 44319_2023_54_MOESM11_ESM.zip › Figure 2/2D/2_EC_PC_BF.tif]

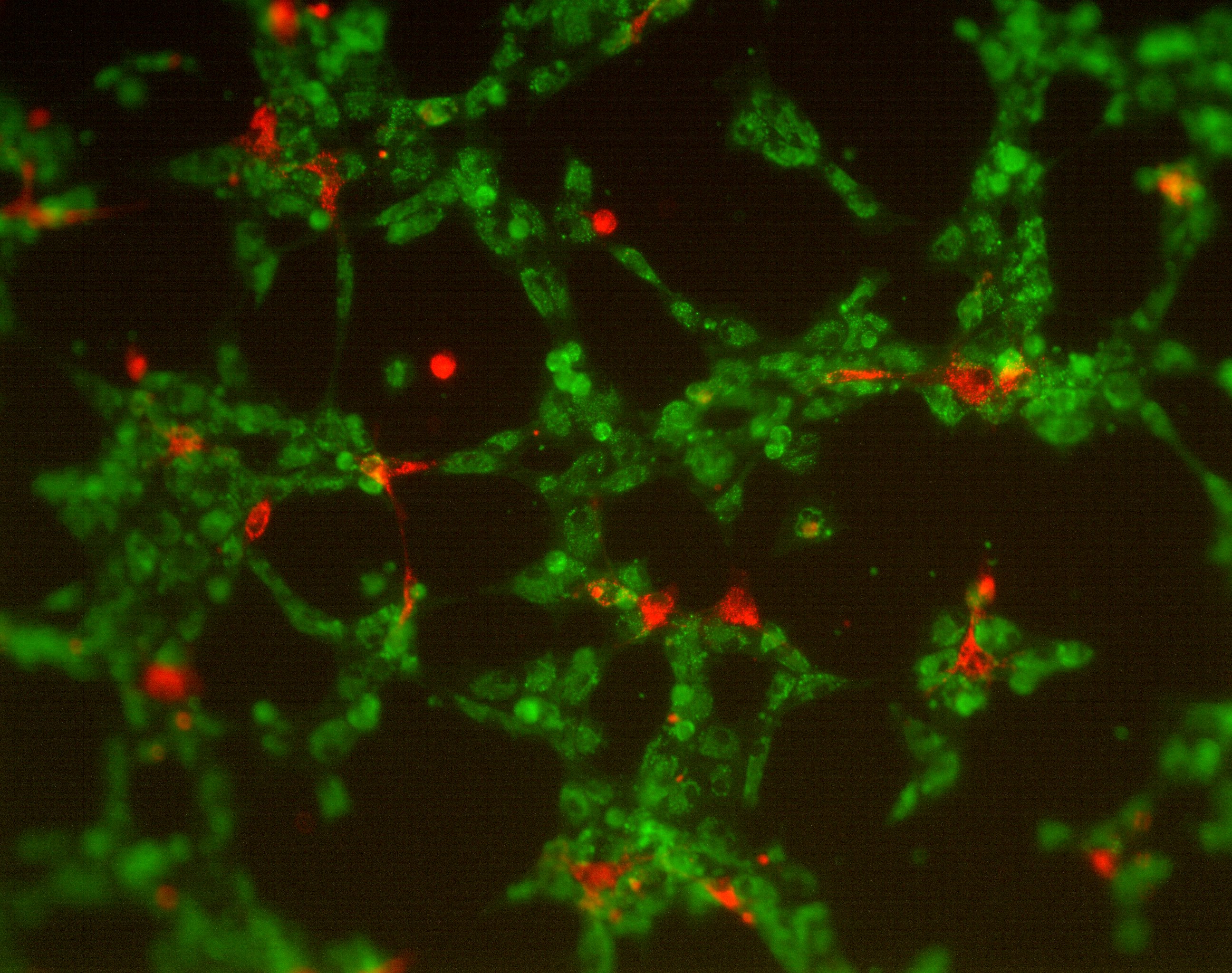

Supplement: Supplementary file 11 — Source Data Fig. 2 [file 44319_2023_54_MOESM11_ESM.zip › Figure 2/2D/2_EC_PC_RG.jpg]

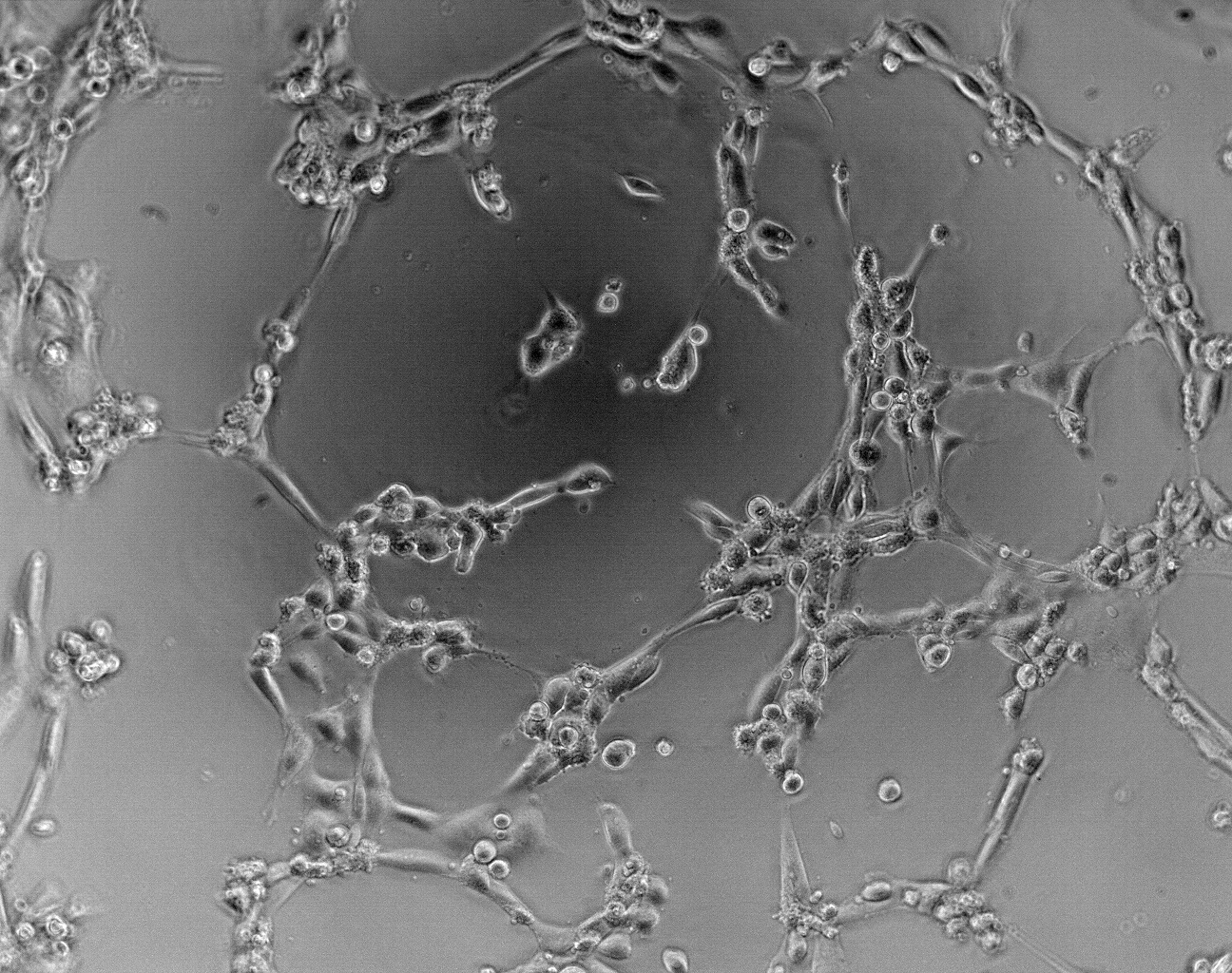

Supplement: Supplementary file 11 — Source Data Fig. 2 [file 44319_2023_54_MOESM11_ESM.zip › Figure 2/2D/3_EC_PC HIF2a_BF.tif]

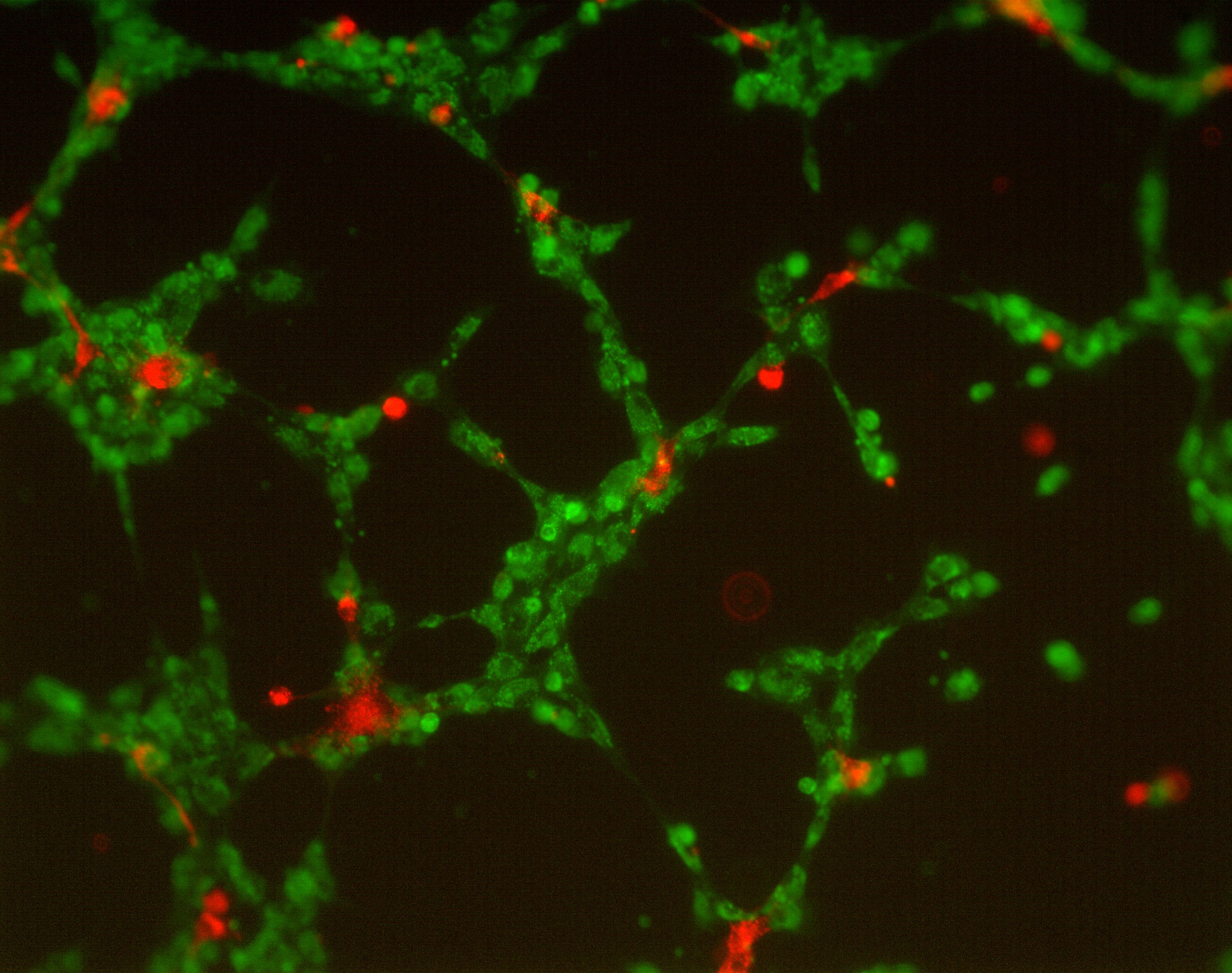

Supplement: Supplementary file 11 — Source Data Fig. 2 [file 44319_2023_54_MOESM11_ESM.zip › Figure 2/2D/3_EC_PC HIF2a_RG.jpg]

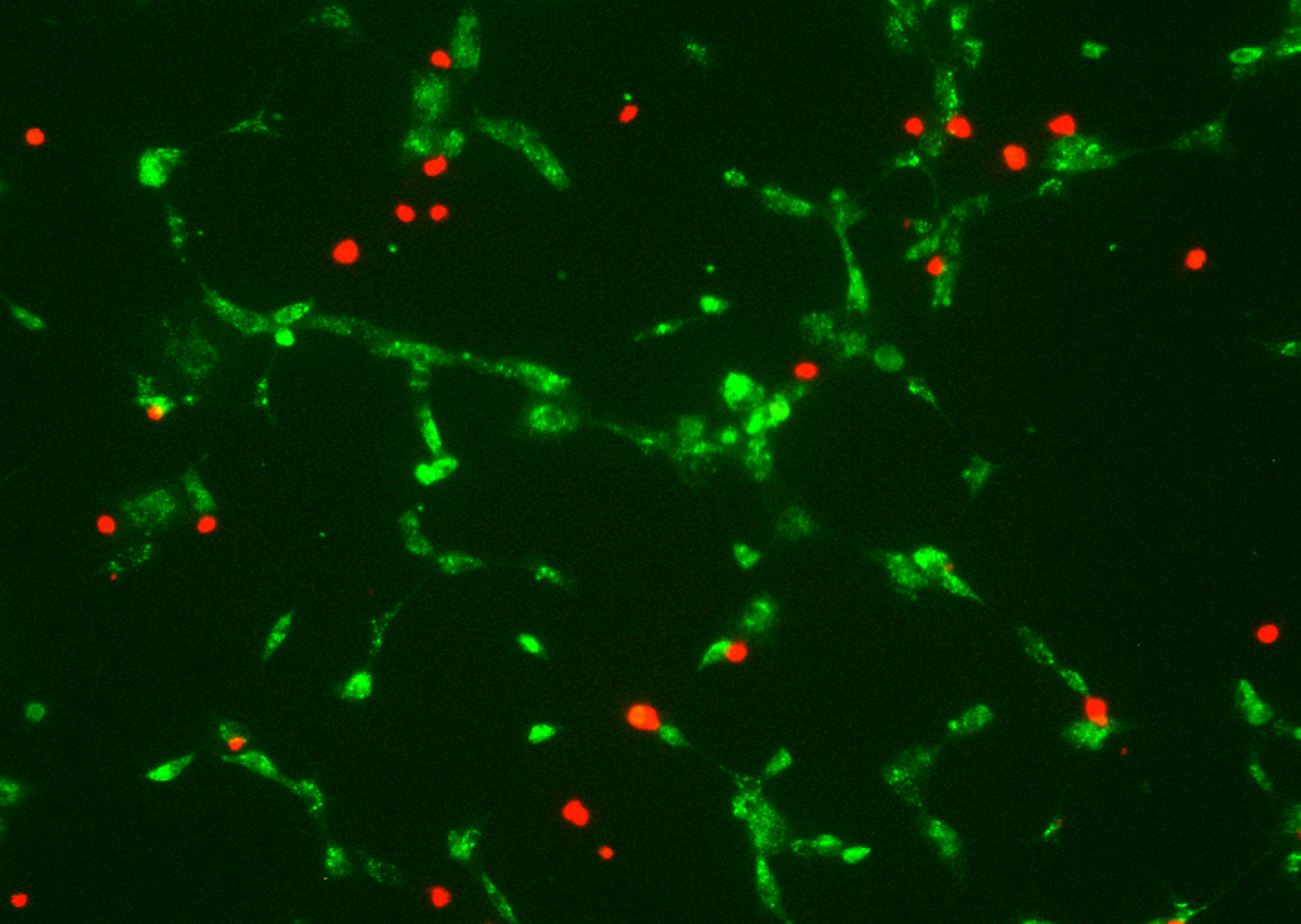

Supplement: Supplementary file 11 — Source Data Fig. 2 [file 44319_2023_54_MOESM11_ESM.zip › Figure 2/2D/4-PC+EC+Hx _RG.tif]

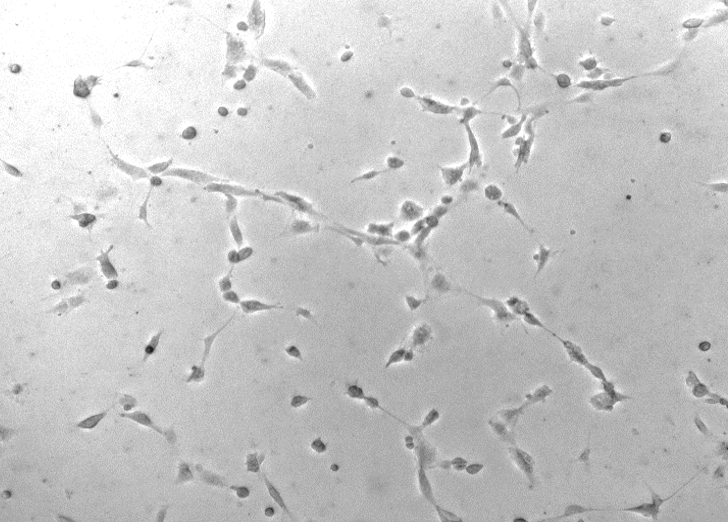

Supplement: Supplementary file 11 — Source Data Fig. 2 [file 44319_2023_54_MOESM11_ESM.zip › Figure 2/2D/4-PC+EC+Hx_BF.tif]

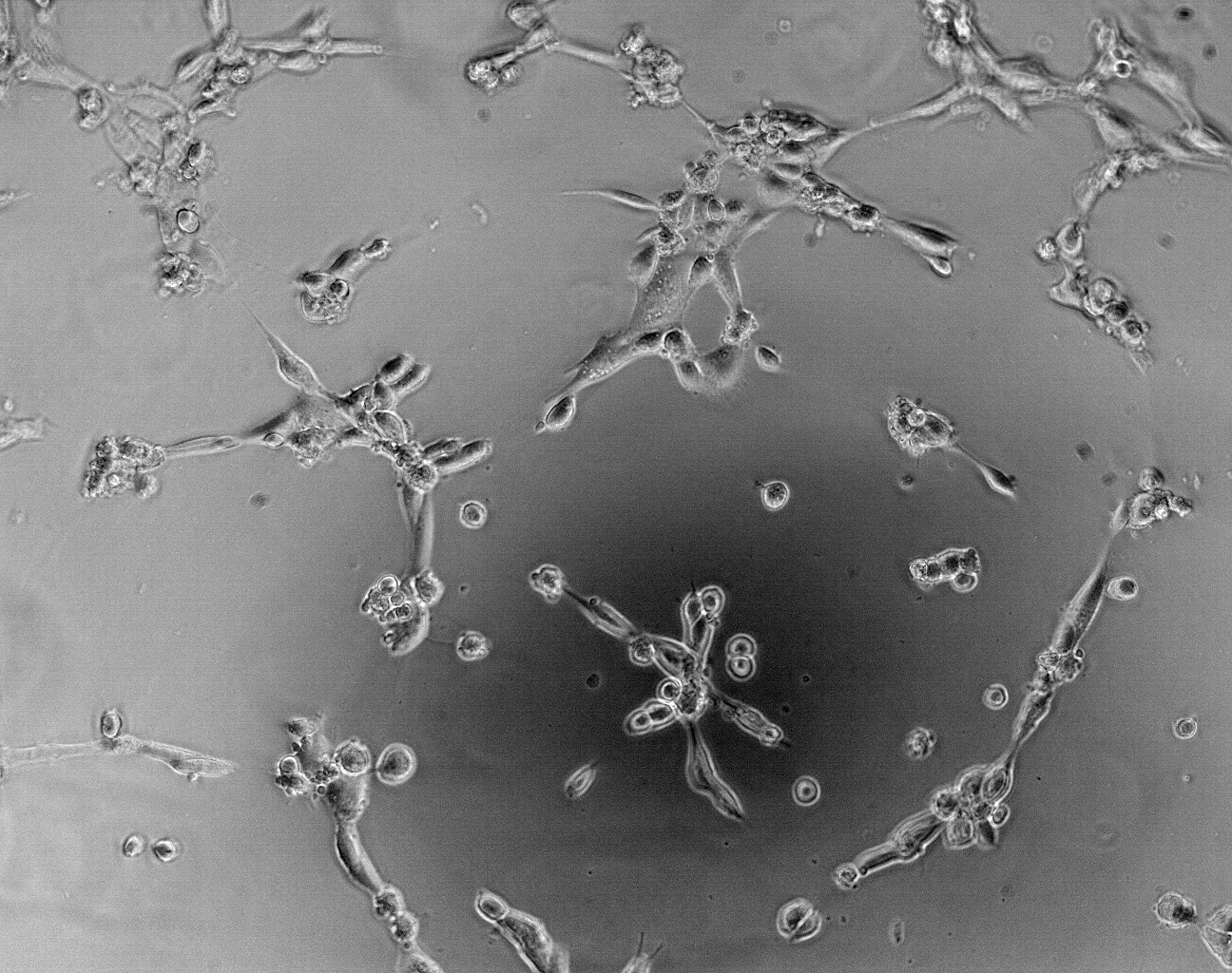

Supplement: Supplementary file 11 — Source Data Fig. 2 [file 44319_2023_54_MOESM11_ESM.zip › Figure 2/2D/5_EC_PC HIF2a_Hx_BF.tif]

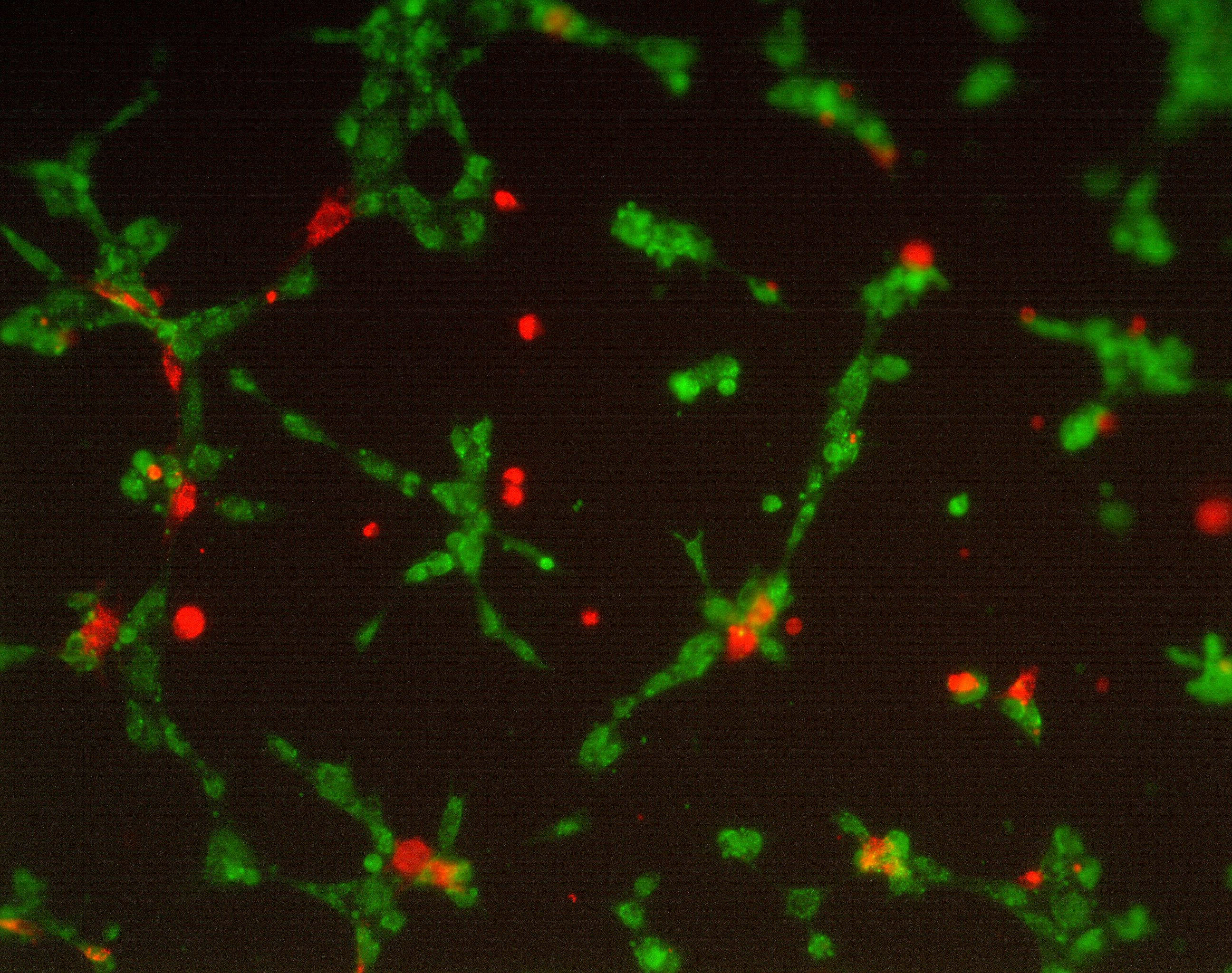

Supplement: Supplementary file 11 — Source Data Fig. 2 [file 44319_2023_54_MOESM11_ESM.zip › Figure 2/2D/5_EC_PC HIF2a_Hx_RG.jpg]

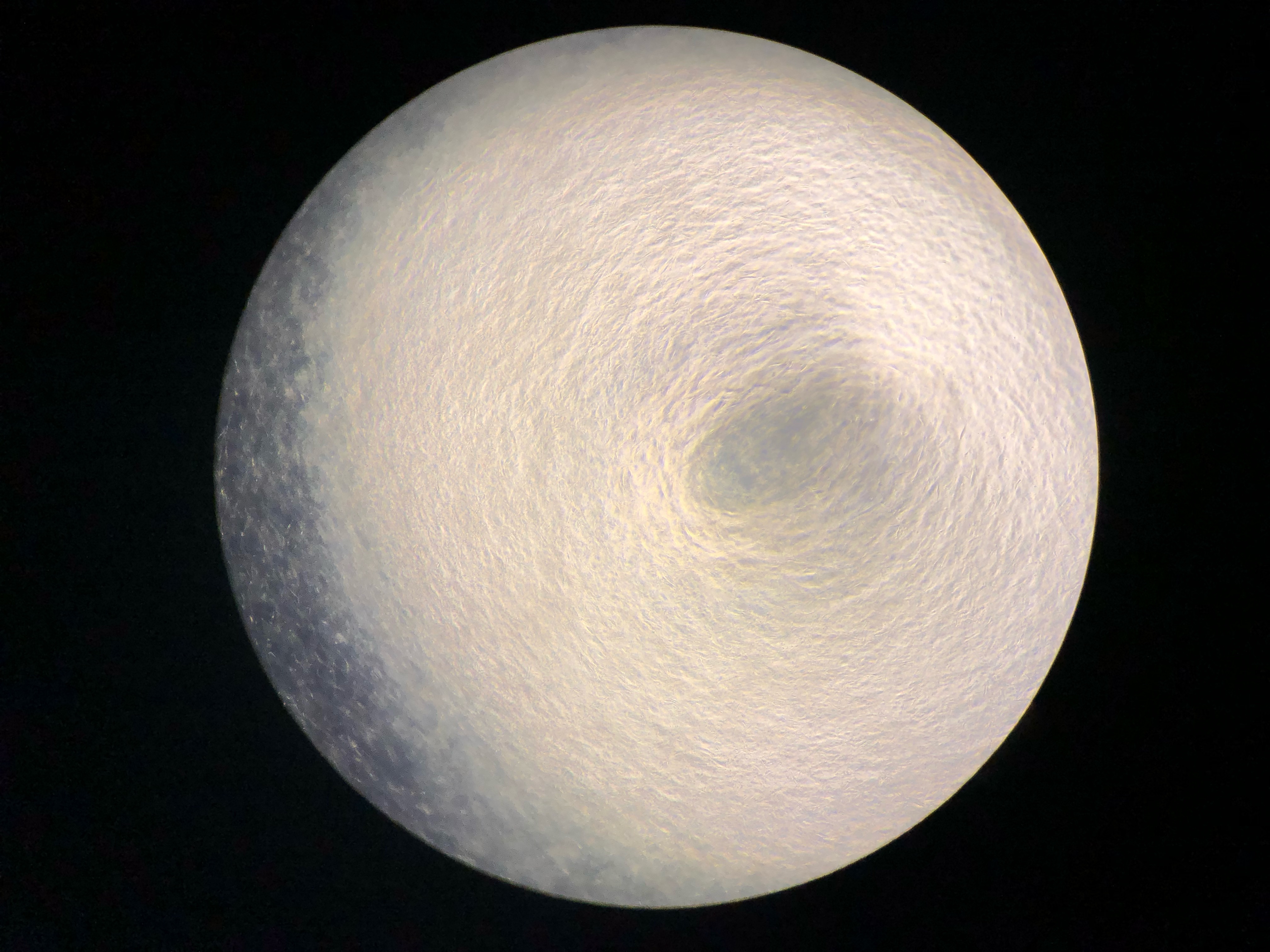

Supplement: Supplementary file 11 — Source Data Fig. 2 [file 44319_2023_54_MOESM11_ESM.zip › Figure 2/2E/1_PC_Norm.jpeg]

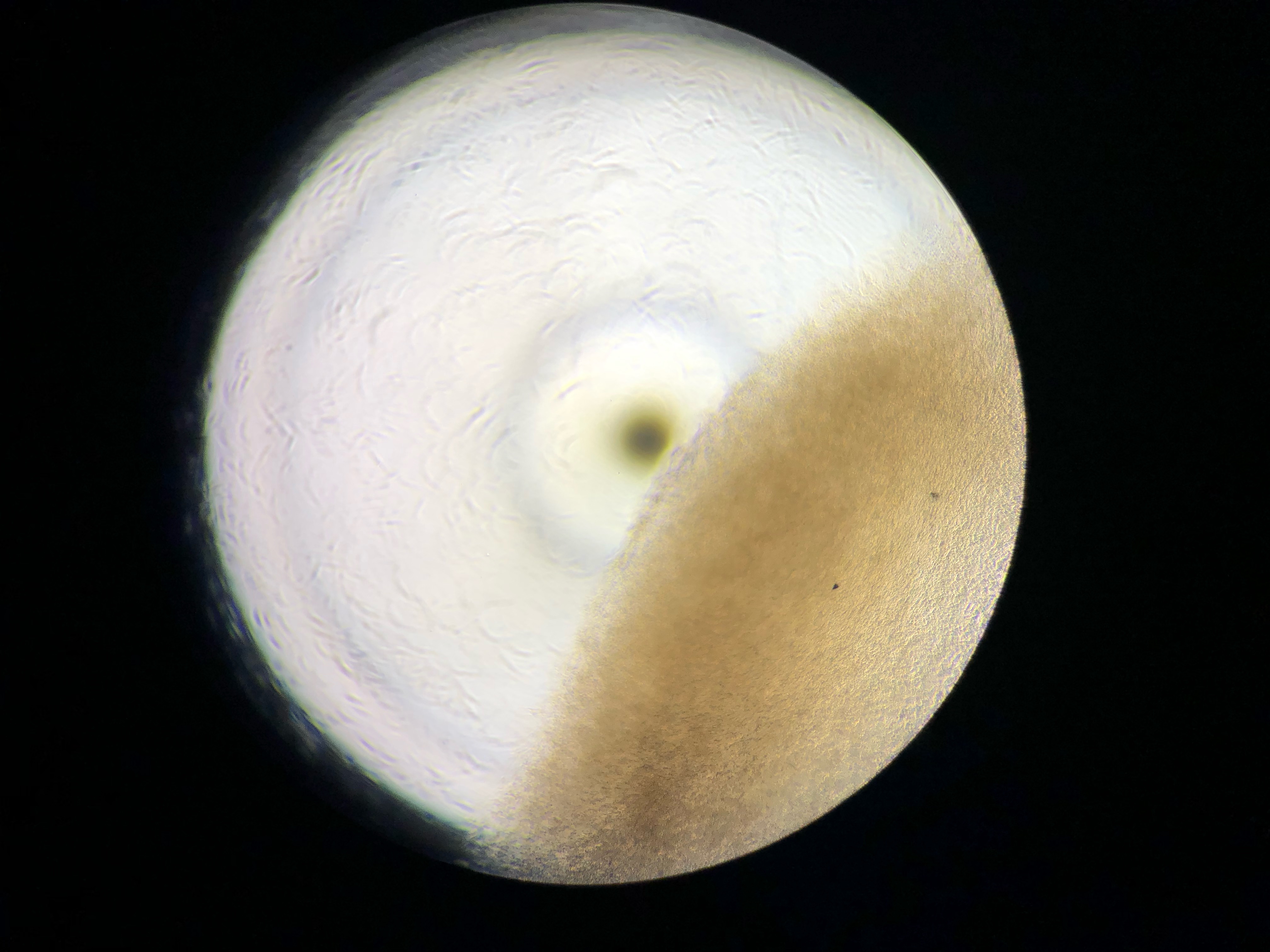

Supplement: Supplementary file 11 — Source Data Fig. 2 [file 44319_2023_54_MOESM11_ESM.zip › Figure 2/2E/2_PC_Hx.jpeg]

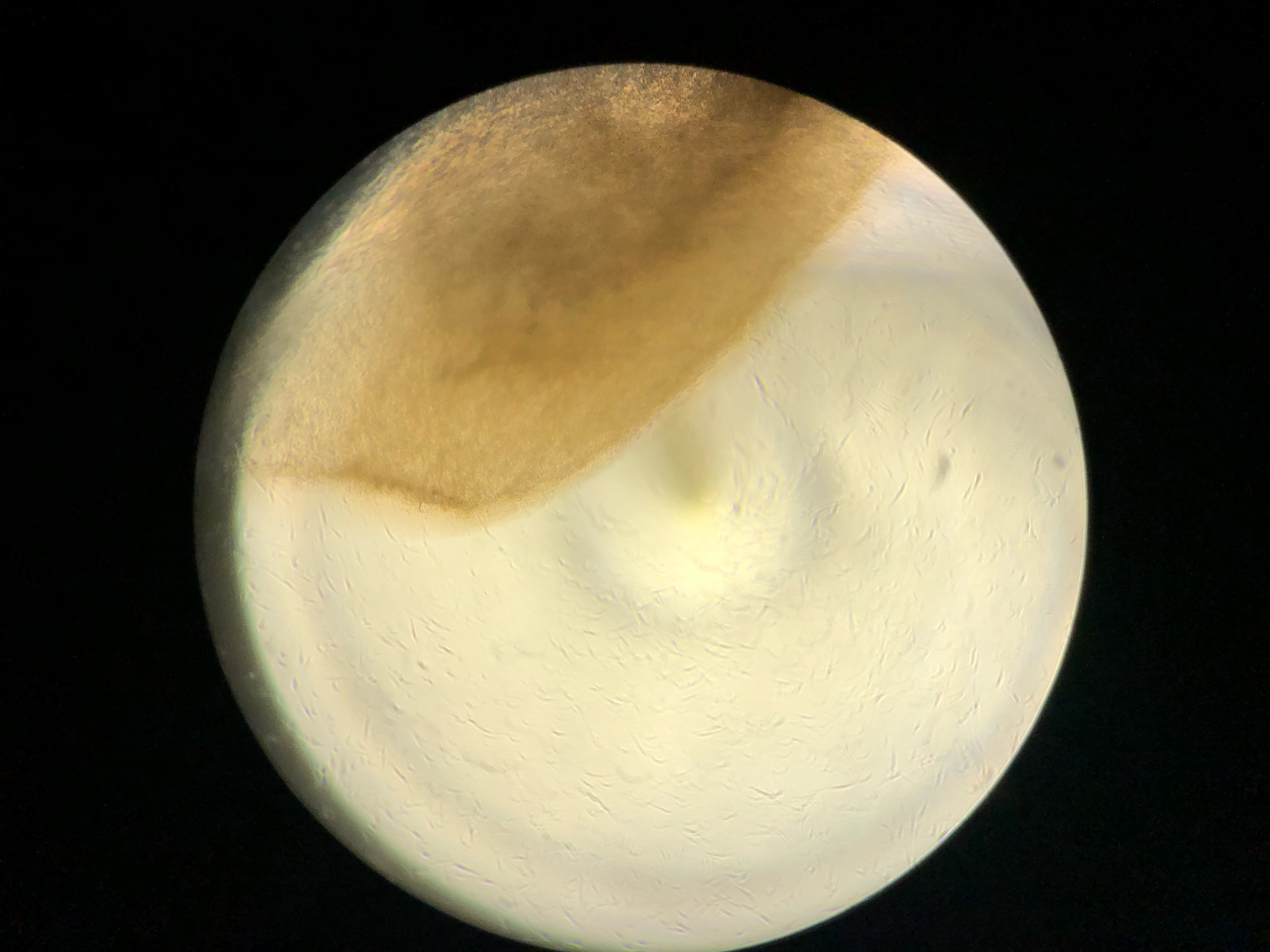

Supplement: Supplementary file 11 — Source Data Fig. 2 [file 44319_2023_54_MOESM11_ESM.zip › Figure 2/2E/3_PC HIF2a_Norm.jpeg]

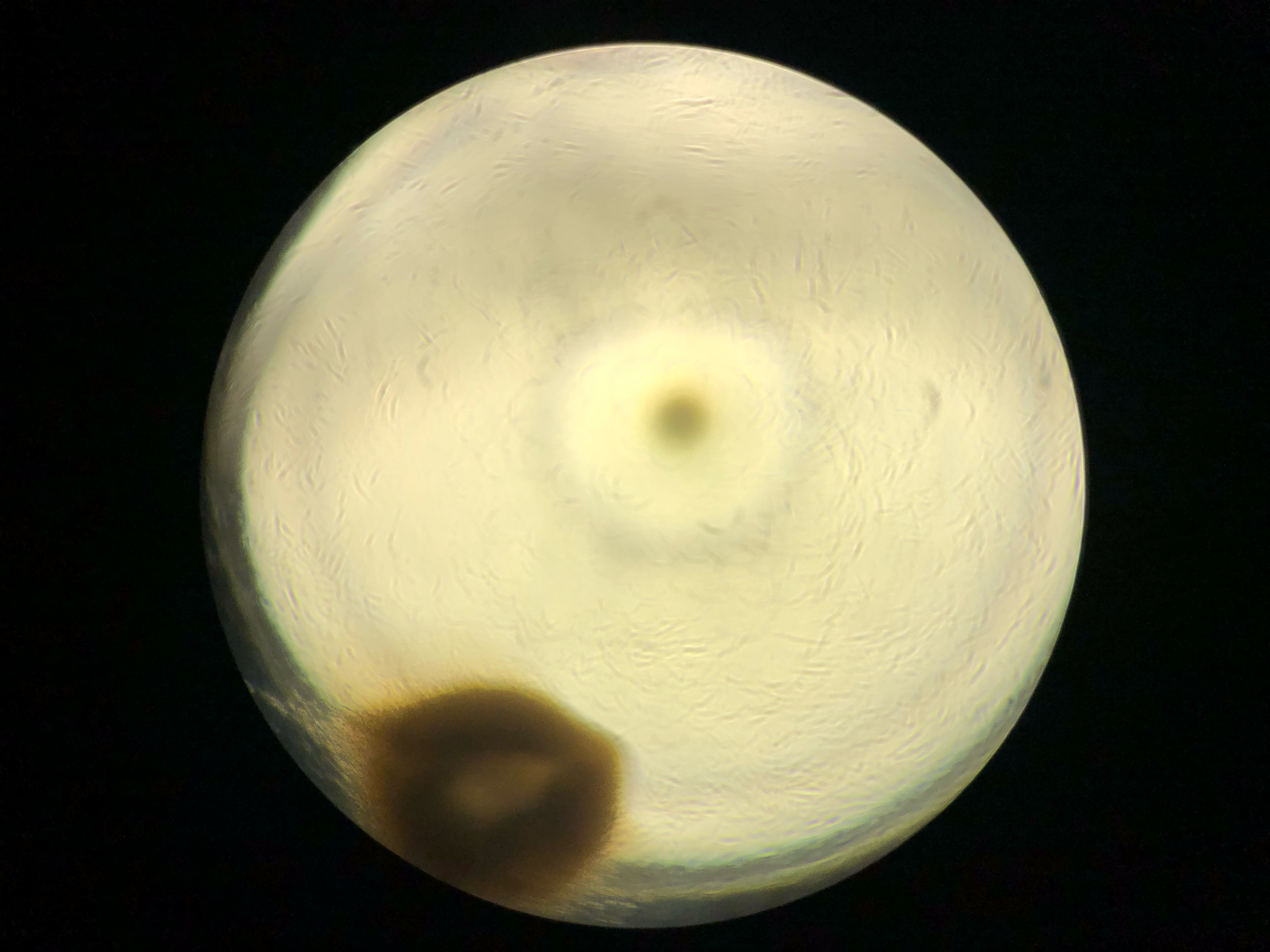

Supplement: Supplementary file 11 — Source Data Fig. 2 [file 44319_2023_54_MOESM11_ESM.zip › Figure 2/2E/4_PC HIF2a_Hx.jpeg]

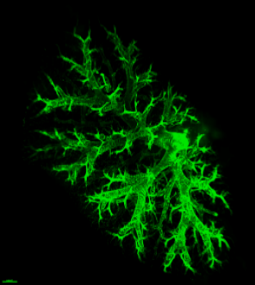

Supplement: Supplementary file 12 — Source Data Fig. 4 [file 44319_2023_54_MOESM12_ESM.zip › 4B/1_WT-Norm.tif]

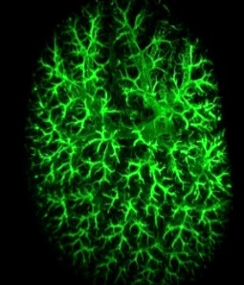

Supplement: Supplementary file 12 — Source Data Fig. 4 [file 44319_2023_54_MOESM12_ESM.zip › 4B/2_WT-Hx.tif]

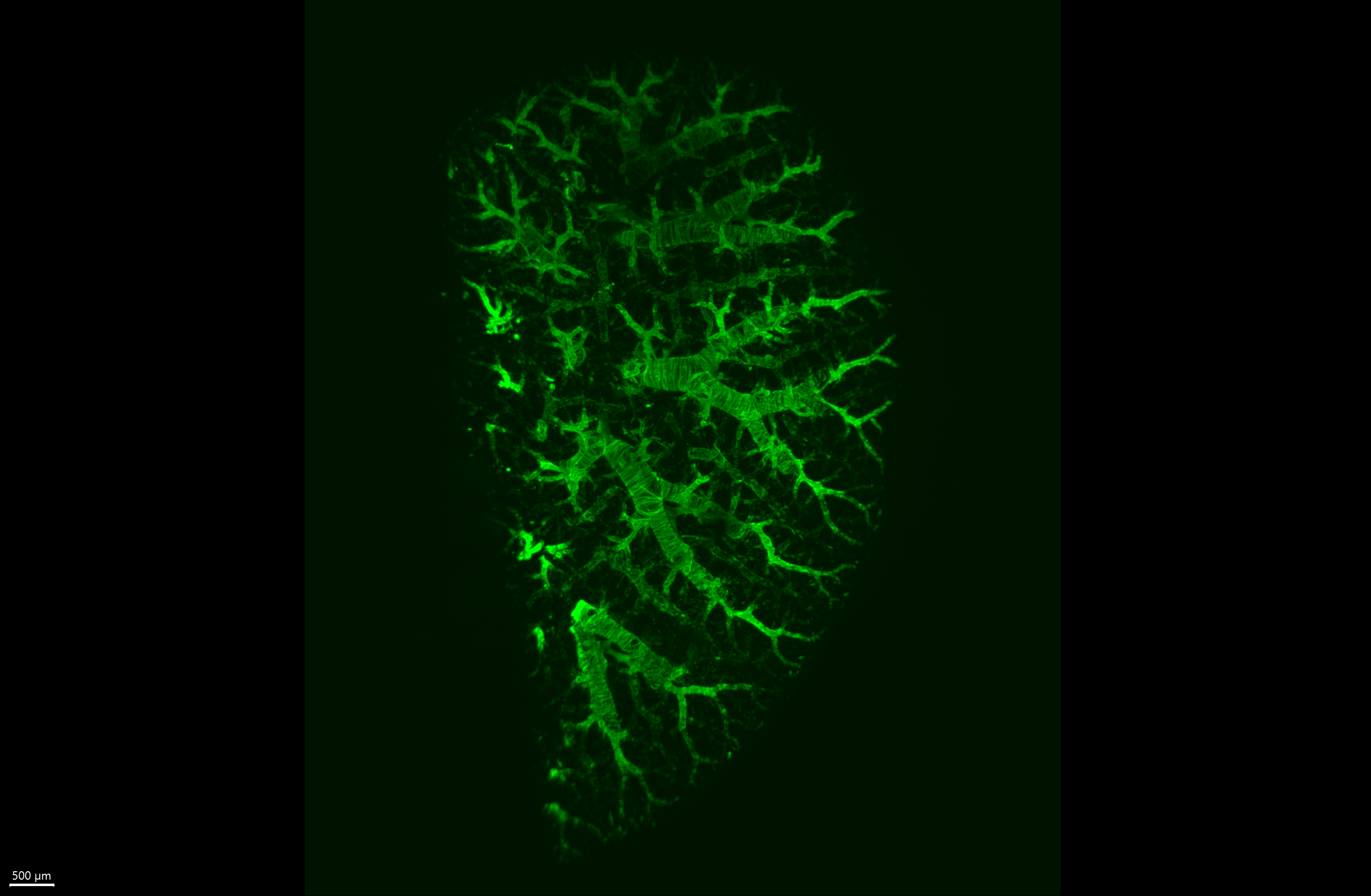

Supplement: Supplementary file 12 — Source Data Fig. 4 [file 44319_2023_54_MOESM12_ESM.zip › 4B/3_NG2 HIF2a OE norm.tif]

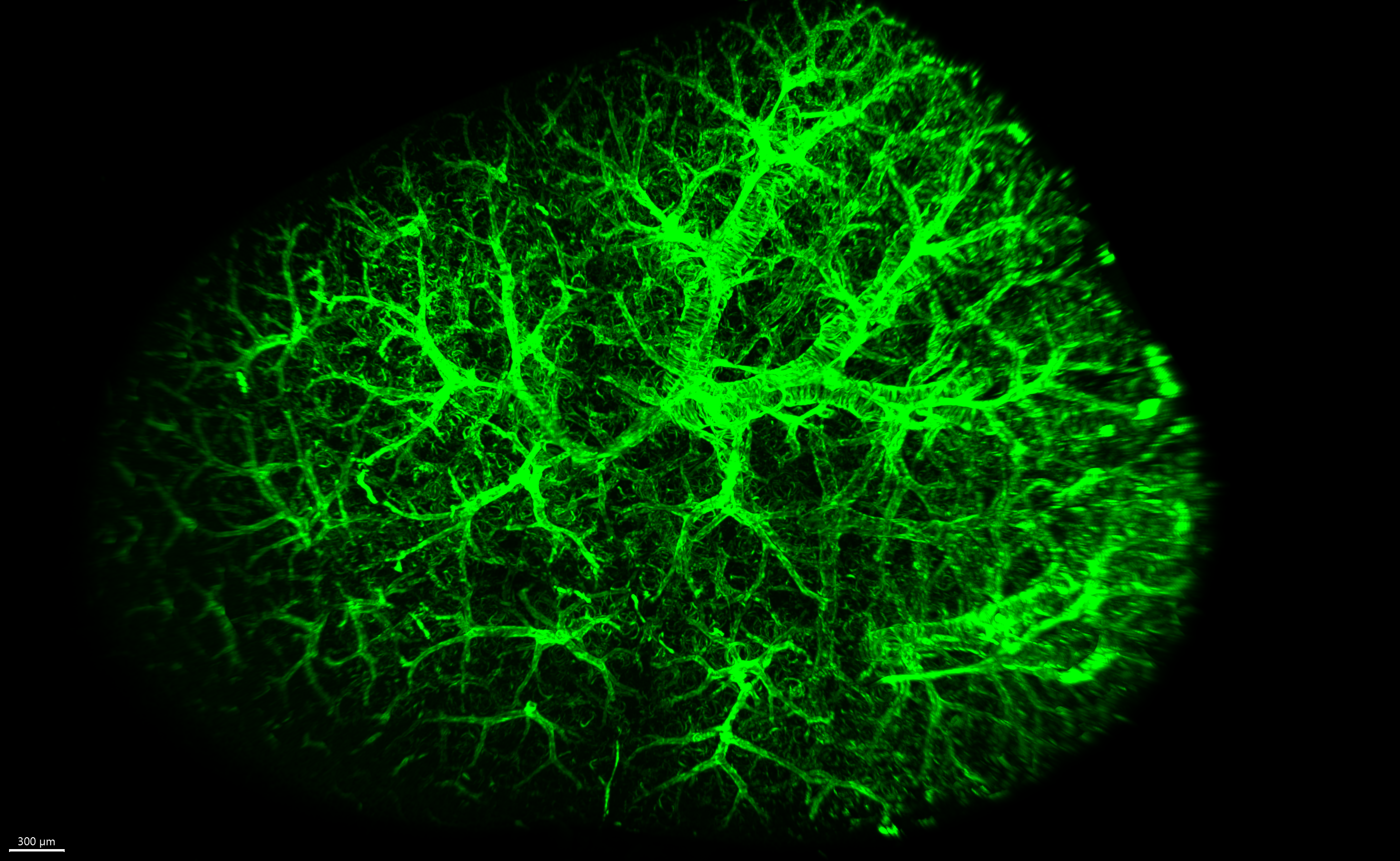

Supplement: Supplementary file 12 — Source Data Fig. 4 [file 44319_2023_54_MOESM12_ESM.zip › 4B/4_NG2 HIF2a OE Hx.tif]

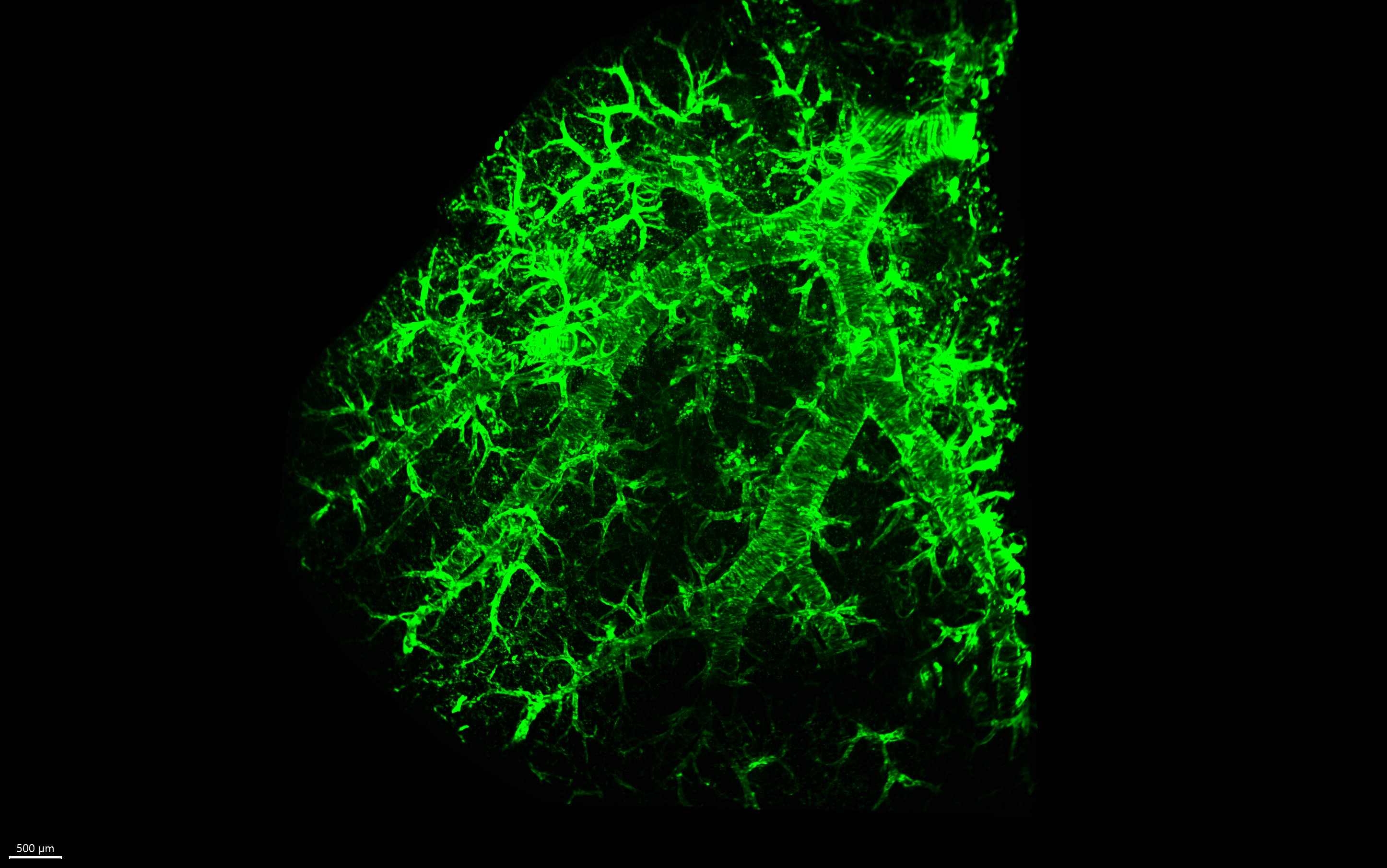

Supplement: Supplementary file 12 — Source Data Fig. 4 [file 44319_2023_54_MOESM12_ESM.zip › 4B/5_NG2 HIF2a KO norm.tif]

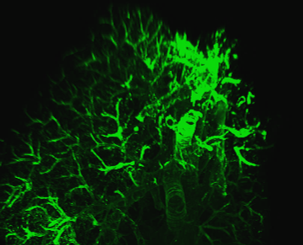

Supplement: Supplementary file 12 — Source Data Fig. 4 [file 44319_2023_54_MOESM12_ESM.zip › 4B/6_NG2 HIF2a KO Hx.tif]

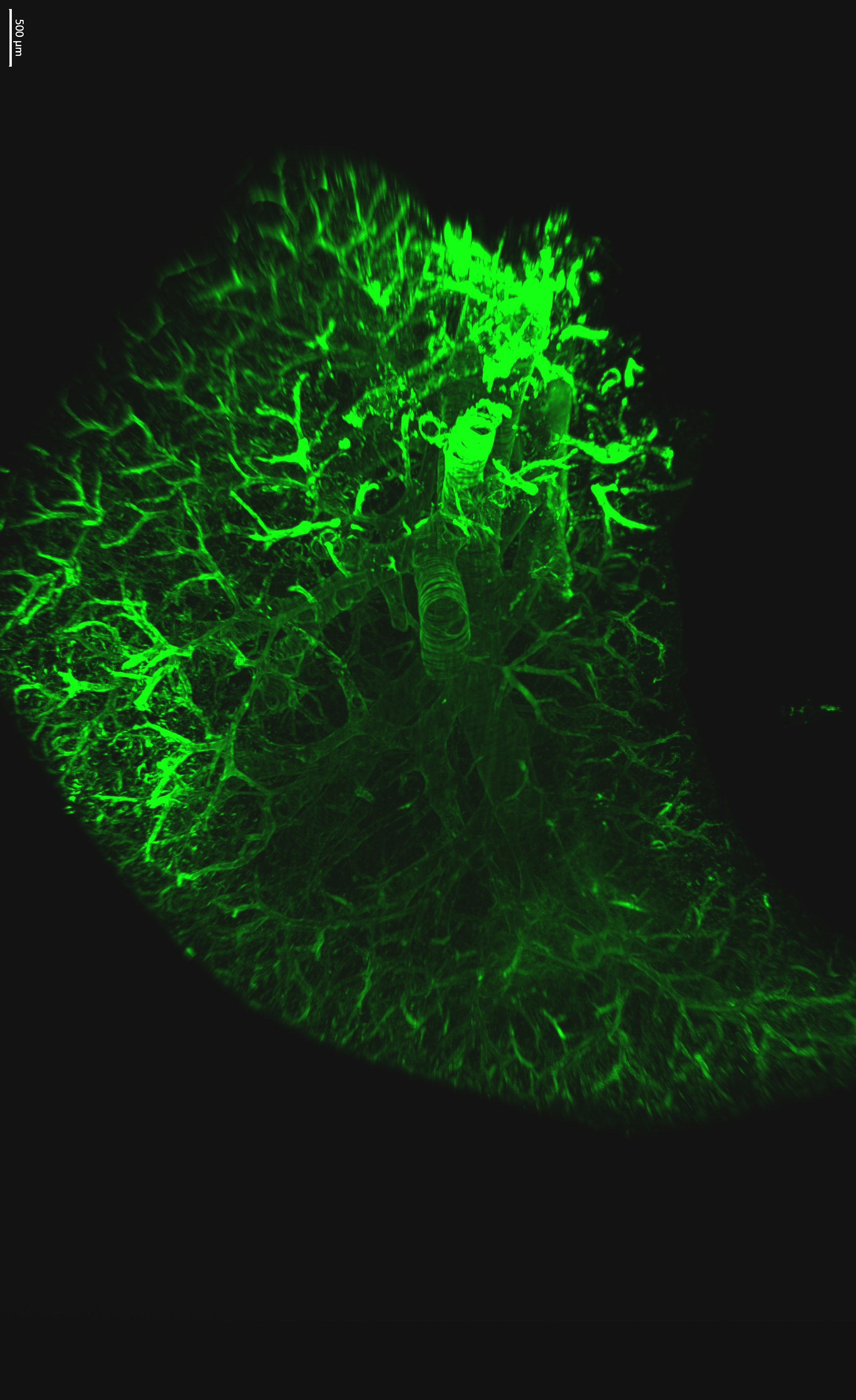

Supplement: Supplementary file 12 — Source Data Fig. 4 [file 44319_2023_54_MOESM12_ESM.zip › 4B/Fig4B-NG2hif2a-KO-3wkhx.tif]

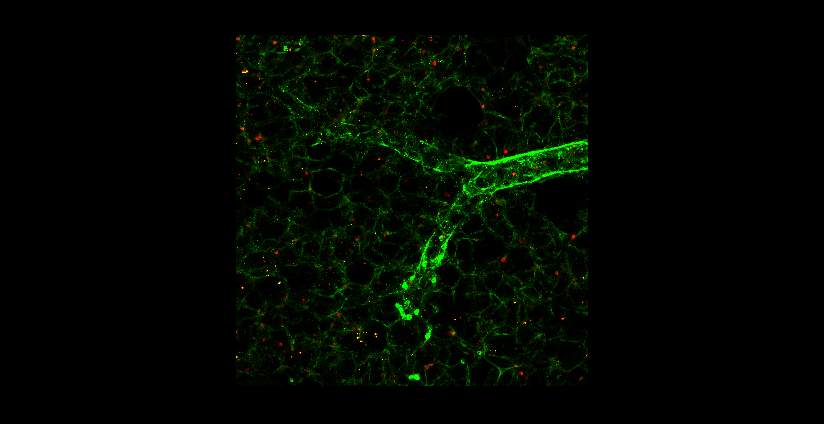

Supplement: Supplementary file 12 — Source Data Fig. 4 [file 44319_2023_54_MOESM12_ESM.zip › 4D/1_NG2_WT_Normoxia_HIF2a-Red_CD31-Green.bmp]

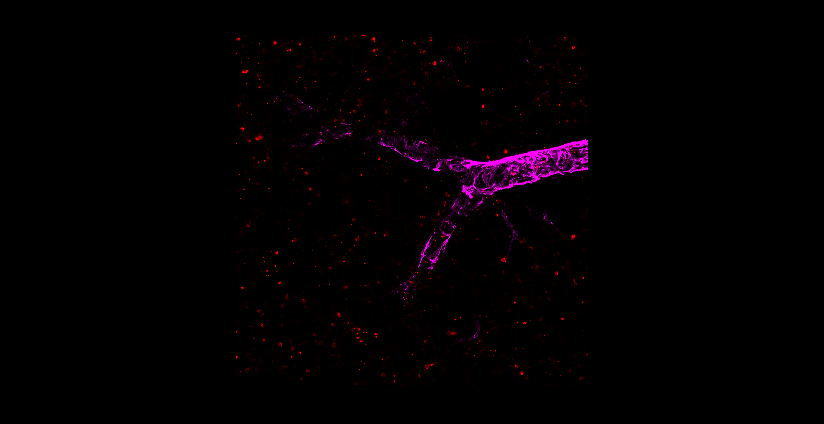

Supplement: Supplementary file 12 — Source Data Fig. 4 [file 44319_2023_54_MOESM12_ESM.zip › 4D/1_NG2_WT_Normoxia_HIF2a-Red_SMA-Magenta.bmp]

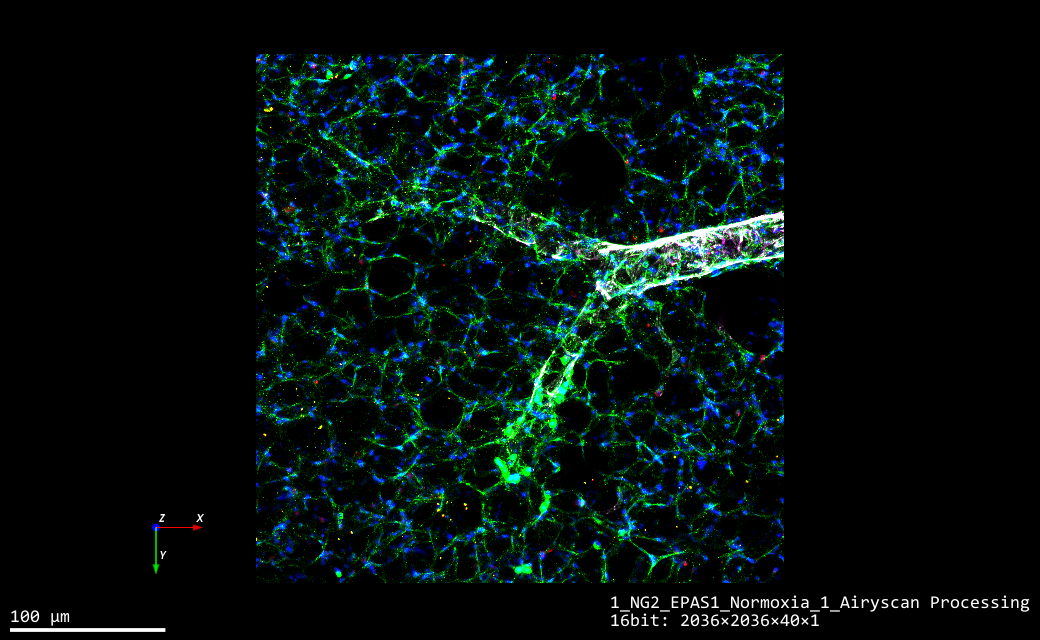

Supplement: Supplementary file 12 — Source Data Fig. 4 [file 44319_2023_54_MOESM12_ESM.zip › 4D/1_NG2_WT_Normoxia_Merge.bmp]

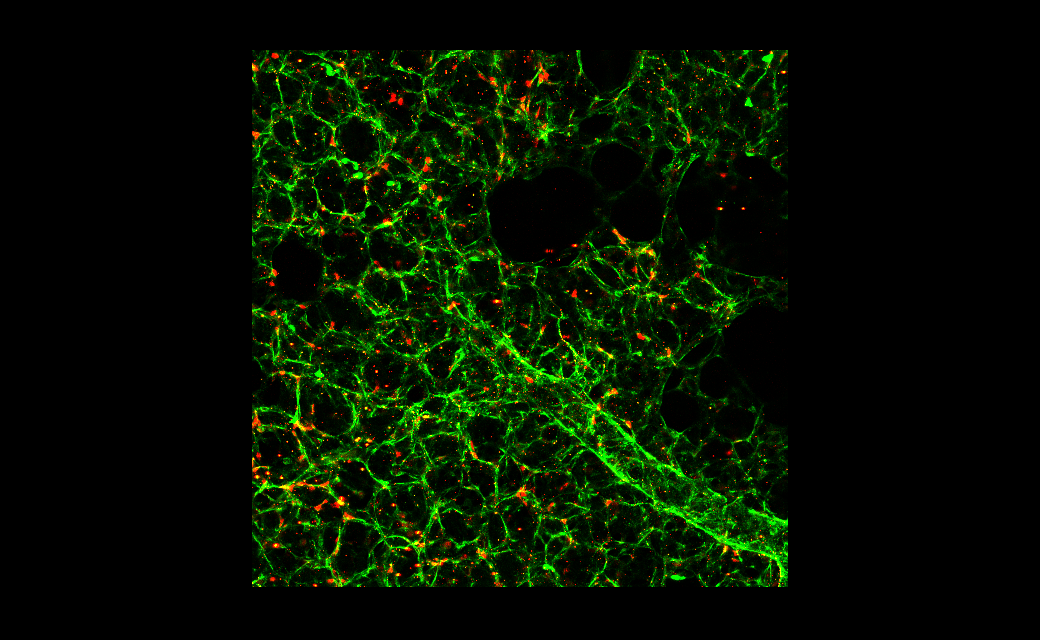

Supplement: Supplementary file 12 — Source Data Fig. 4 [file 44319_2023_54_MOESM12_ESM.zip › 4D/2_NG2_HIF2a OE_Normoxia_HIF2a-Red_CD31-Green.bmp]

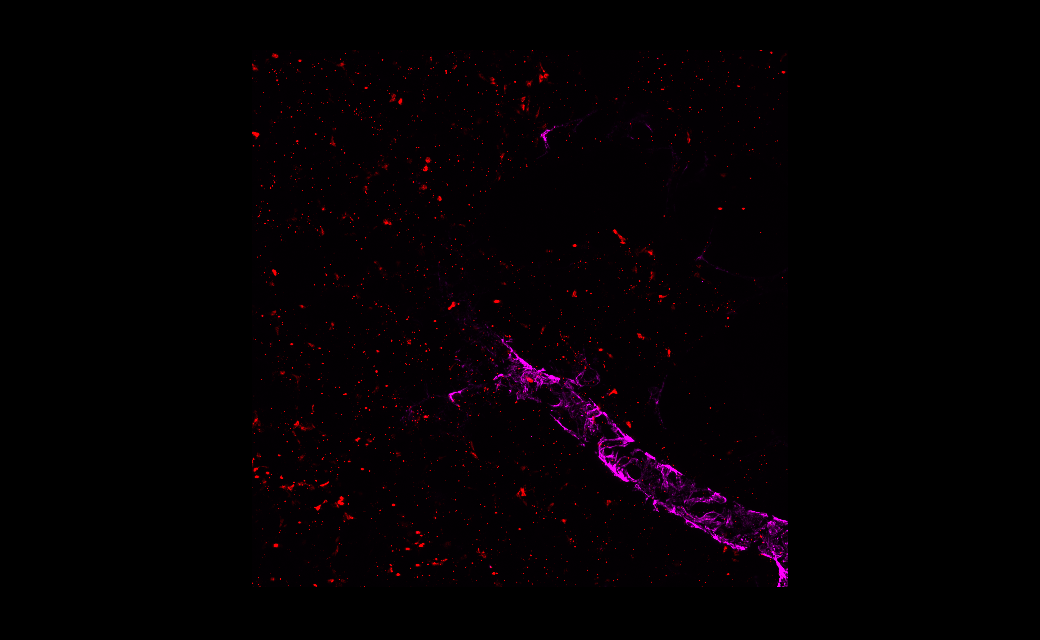

Supplement: Supplementary file 12 — Source Data Fig. 4 [file 44319_2023_54_MOESM12_ESM.zip › 4D/2_NG2_HIF2a OE_Normoxia_HIF2a-Red_SMA-Magenta.bmp]

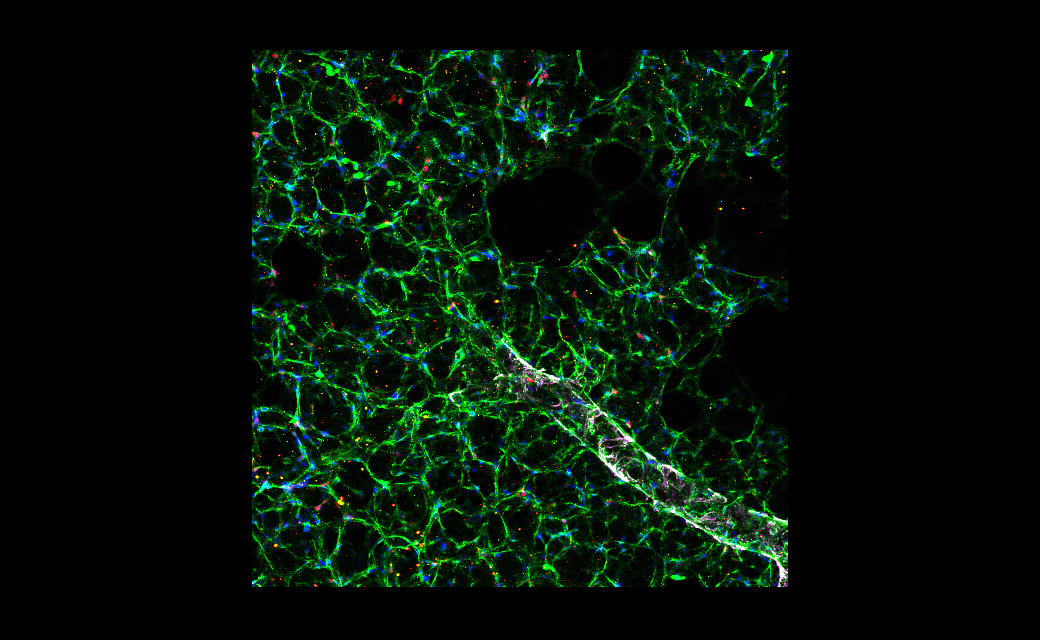

Supplement: Supplementary file 12 — Source Data Fig. 4 [file 44319_2023_54_MOESM12_ESM.zip › 4D/2_NG2_HIF2a OE_Normoxia_Merge.bmp]

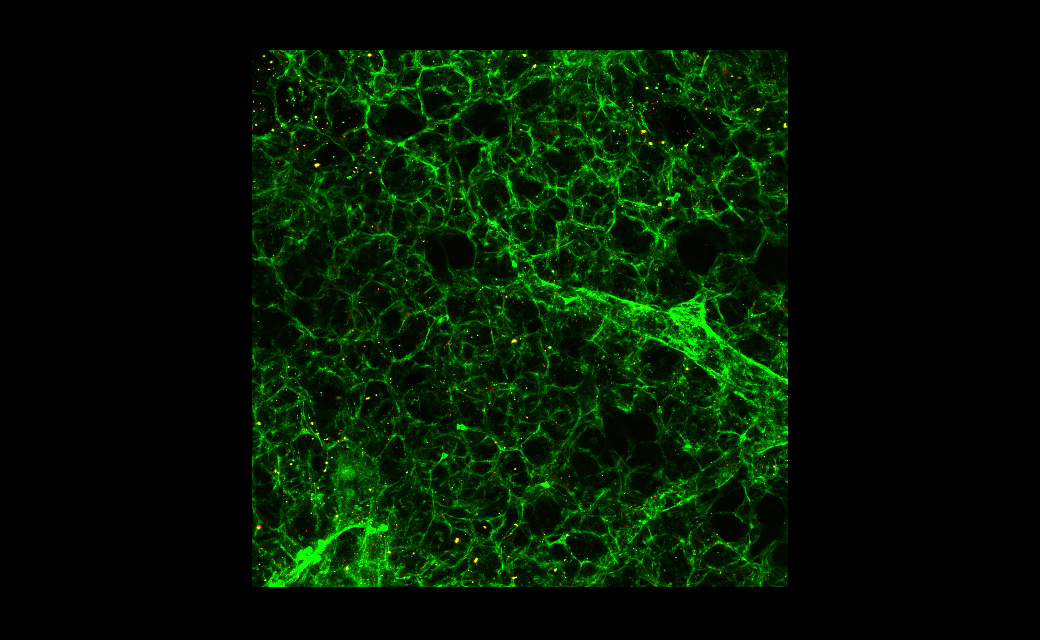

Supplement: Supplementary file 12 — Source Data Fig. 4 [file 44319_2023_54_MOESM12_ESM.zip › 4D/3_NG2_HIF2a KO_Normoxia_HIF2a-Red_CD31-Green.bmp]

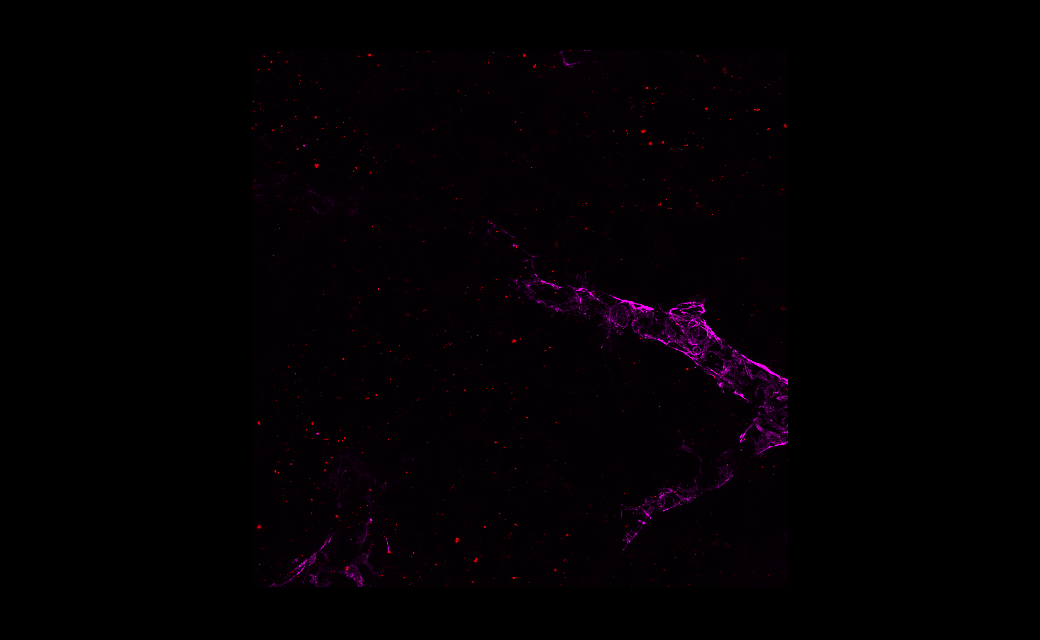

Supplement: Supplementary file 12 — Source Data Fig. 4 [file 44319_2023_54_MOESM12_ESM.zip › 4D/3_NG2_HIF2a KO_Normoxia_HIF2a-Red_SMA-Magenta.bmp]

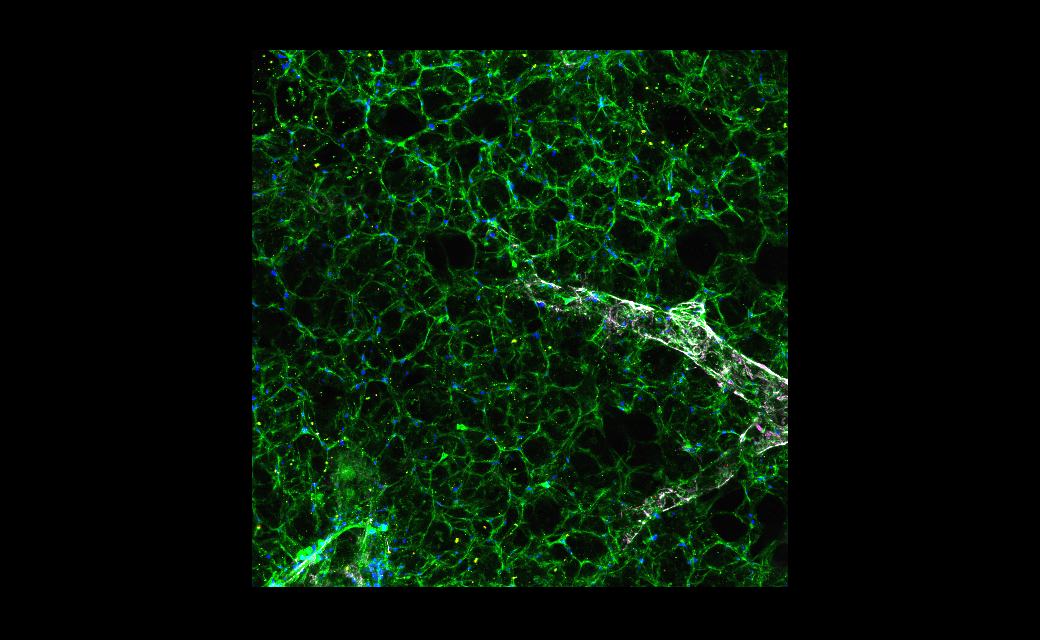

Supplement: Supplementary file 12 — Source Data Fig. 4 [file 44319_2023_54_MOESM12_ESM.zip › 4D/3_NG2_HIF2a KO_Normoxia_Merge.bmp]

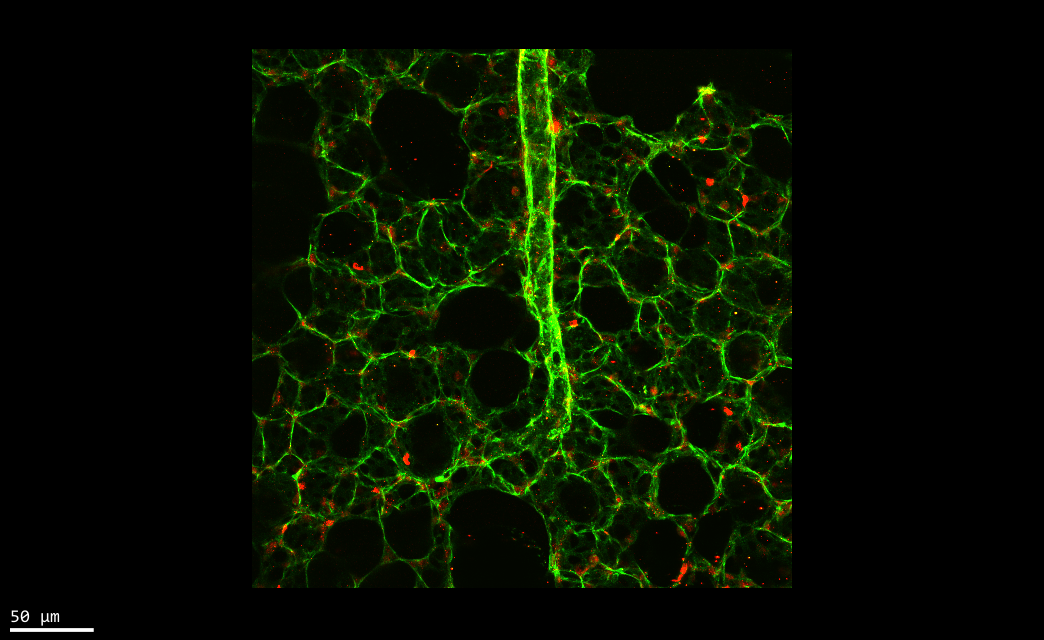

Supplement: Supplementary file 12 — Source Data Fig. 4 [file 44319_2023_54_MOESM12_ESM.zip › 4D/4_NG2_WT_Hx_HIF2a-Red_CD31-Green.bmp]

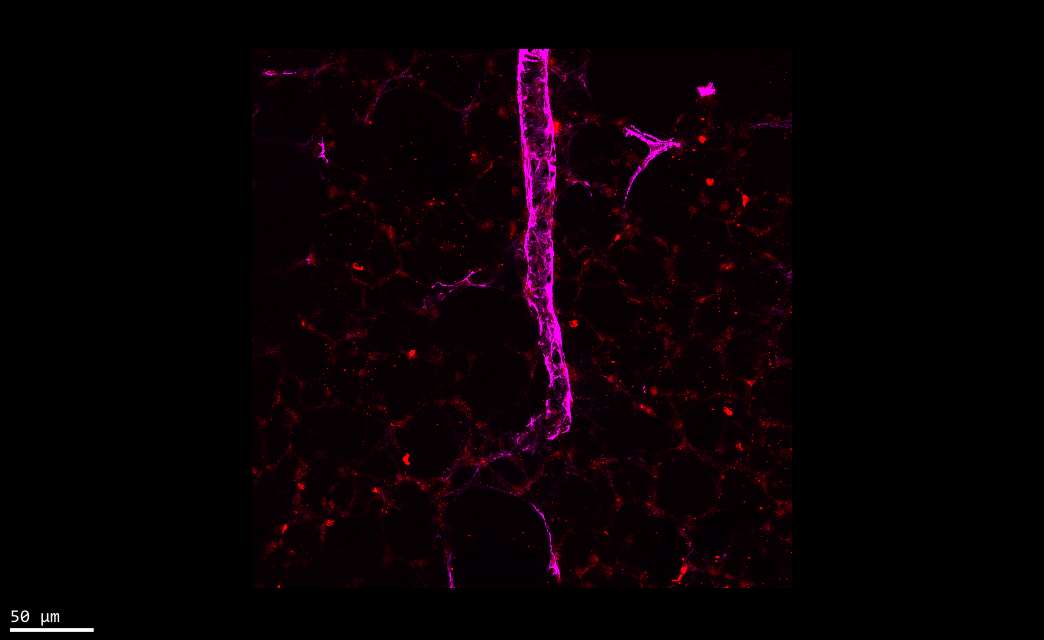

Supplement: Supplementary file 12 — Source Data Fig. 4 [file 44319_2023_54_MOESM12_ESM.zip › 4D/4_NG2_WT_Hx_HIF2a-Red_SMA-Magenta.bmp]

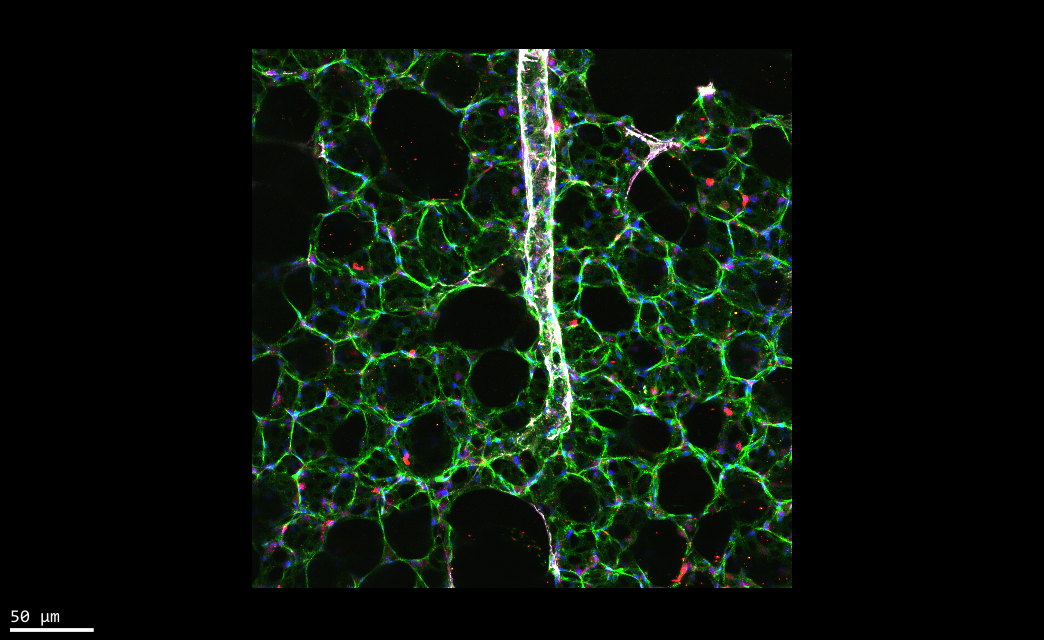

Supplement: Supplementary file 12 — Source Data Fig. 4 [file 44319_2023_54_MOESM12_ESM.zip › 4D/4_NG2_WT_Hx_Merge.bmp]

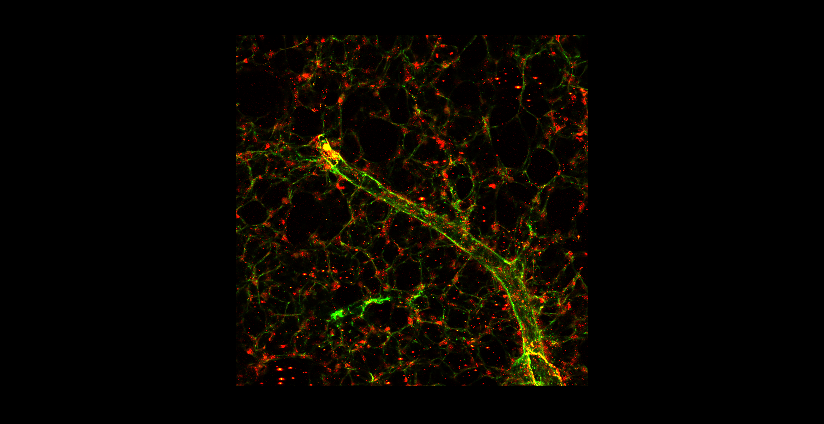

Supplement: Supplementary file 12 — Source Data Fig. 4 [file 44319_2023_54_MOESM12_ESM.zip › 4D/5_NG2_HIF2a OE_Hx_HIF2a-Red_CD31-Green.bmp]

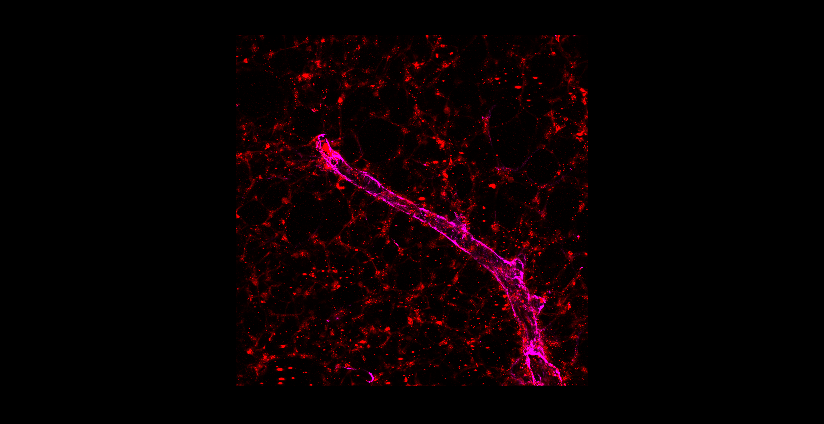

Supplement: Supplementary file 12 — Source Data Fig. 4 [file 44319_2023_54_MOESM12_ESM.zip › 4D/5_NG2_HIF2a OE_Hx_HIF2a-Red_SMA-Magenta.bmp]

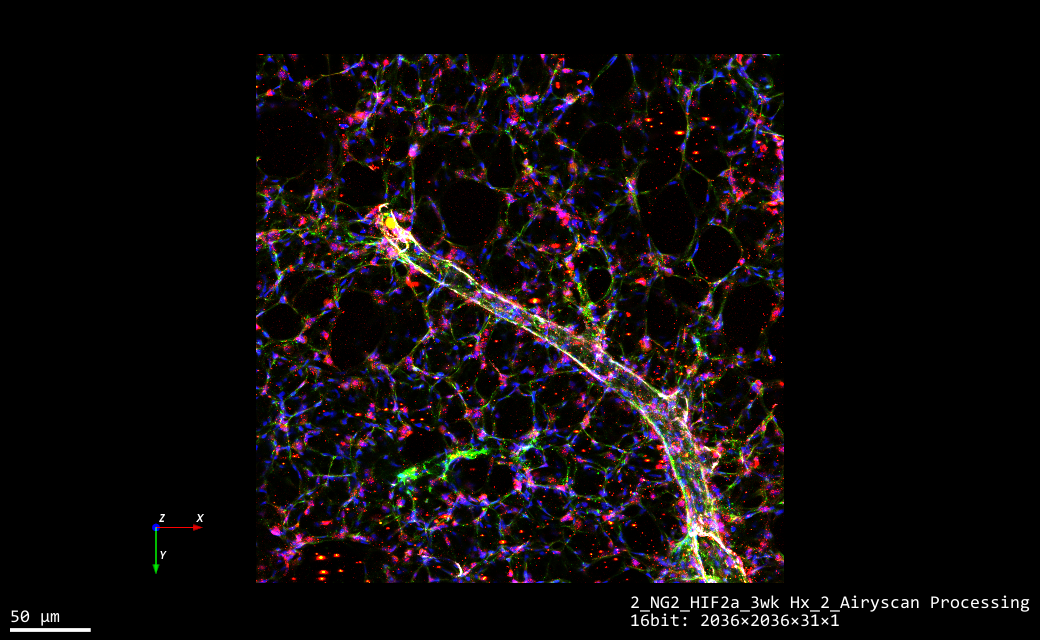

Supplement: Supplementary file 12 — Source Data Fig. 4 [file 44319_2023_54_MOESM12_ESM.zip › 4D/5_NG2_HIF2a OE_Hx_Merge.bmp]

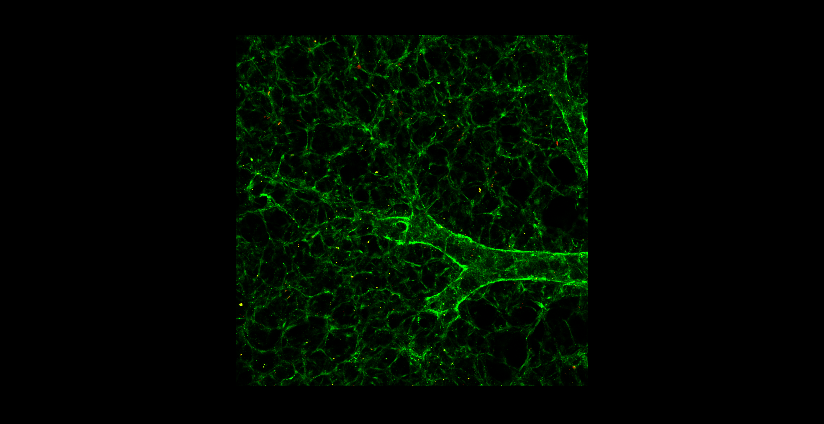

Supplement: Supplementary file 12 — Source Data Fig. 4 [file 44319_2023_54_MOESM12_ESM.zip › 4D/6_NG2_HIF2a KO_Hx_HIF2a-Red_CD31-Green.bmp]

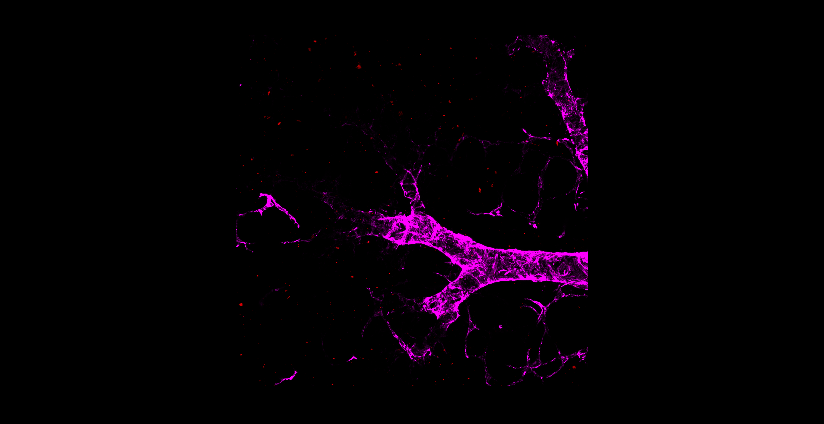

Supplement: Supplementary file 12 — Source Data Fig. 4 [file 44319_2023_54_MOESM12_ESM.zip › 4D/6_NG2_HIF2a KO_Hx_HIF2a-Red_SMA-Magenta.bmp]

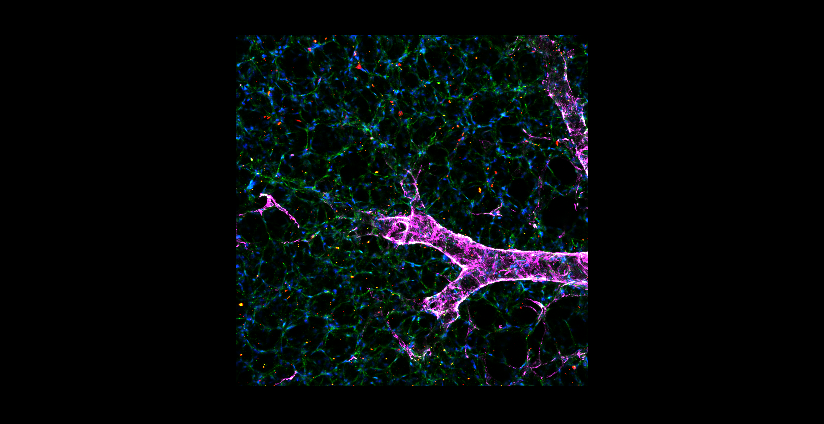

Supplement: Supplementary file 12 — Source Data Fig. 4 [file 44319_2023_54_MOESM12_ESM.zip › 4D/6_NG2_HIF2a KO_Hx_Merge.bmp]

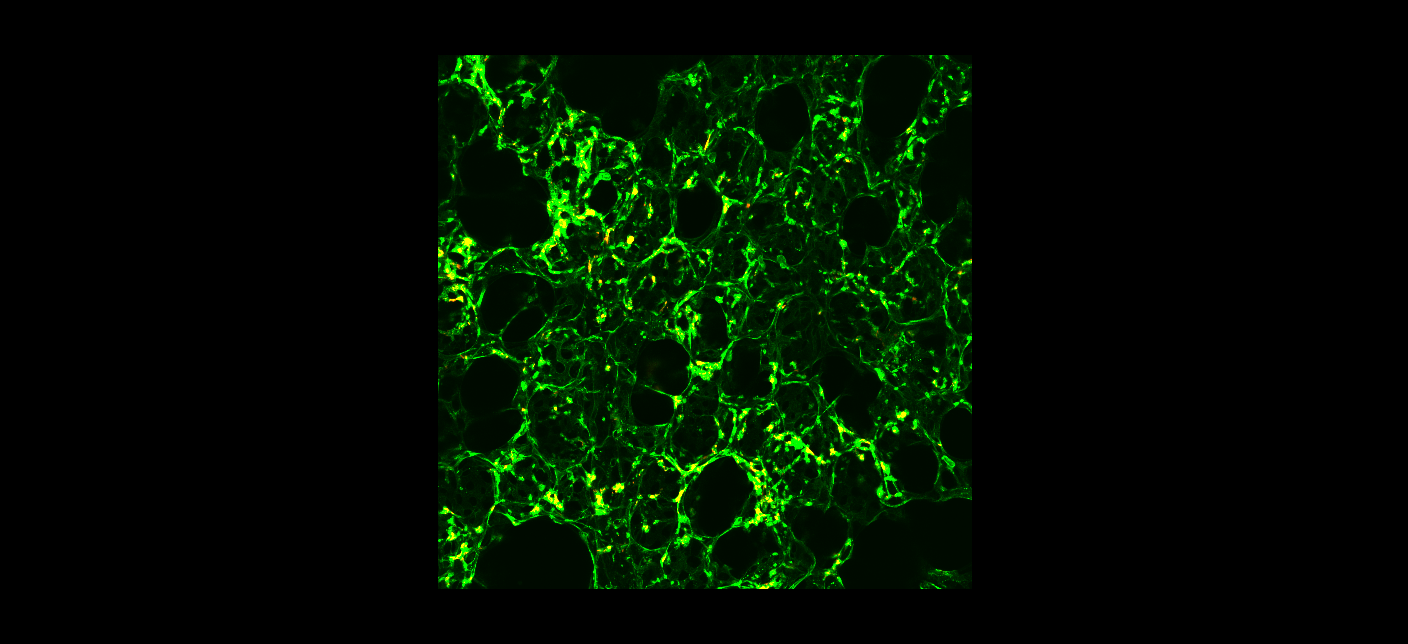

Supplement: Supplementary file 13 — Source Data Fig. 5 [file 44319_2023_54_MOESM13_ESM.zip › 5C/1_WT_Normoxia_Lectin-Green_Merge.bmp]

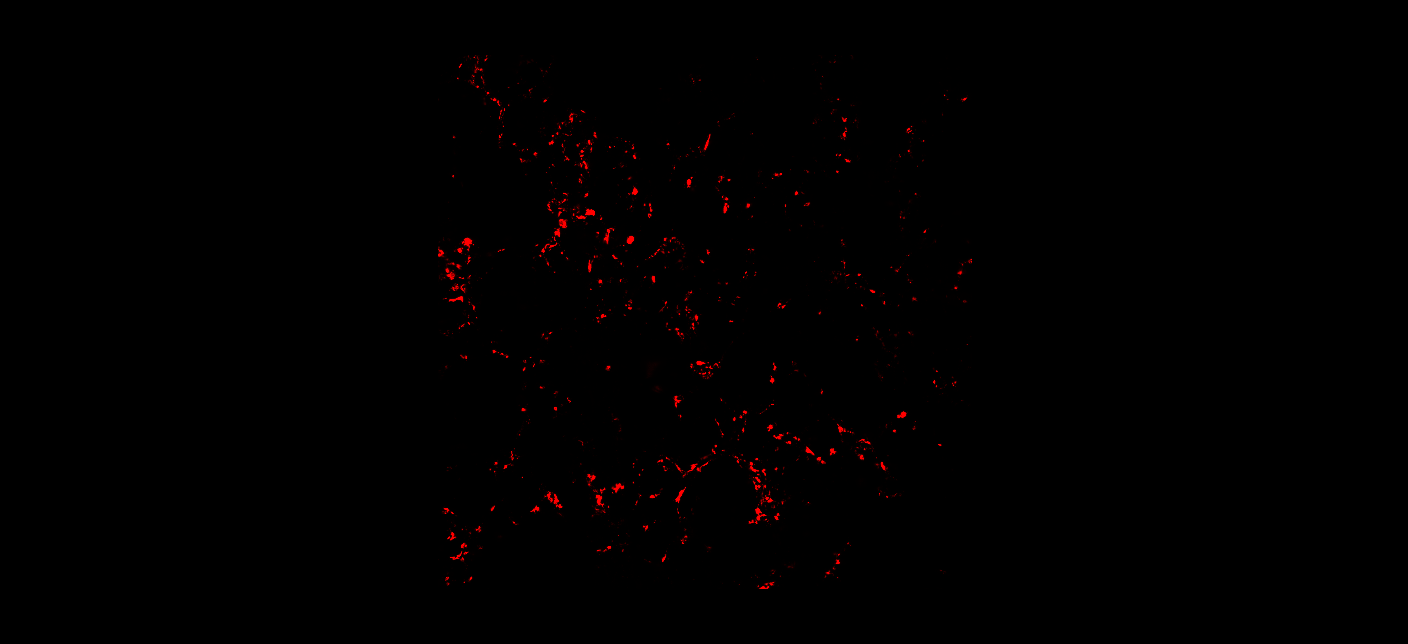

Supplement: Supplementary file 13 — Source Data Fig. 5 [file 44319_2023_54_MOESM13_ESM.zip › 5C/1_WT_Normoxia_Microbeads-Red.bmp]

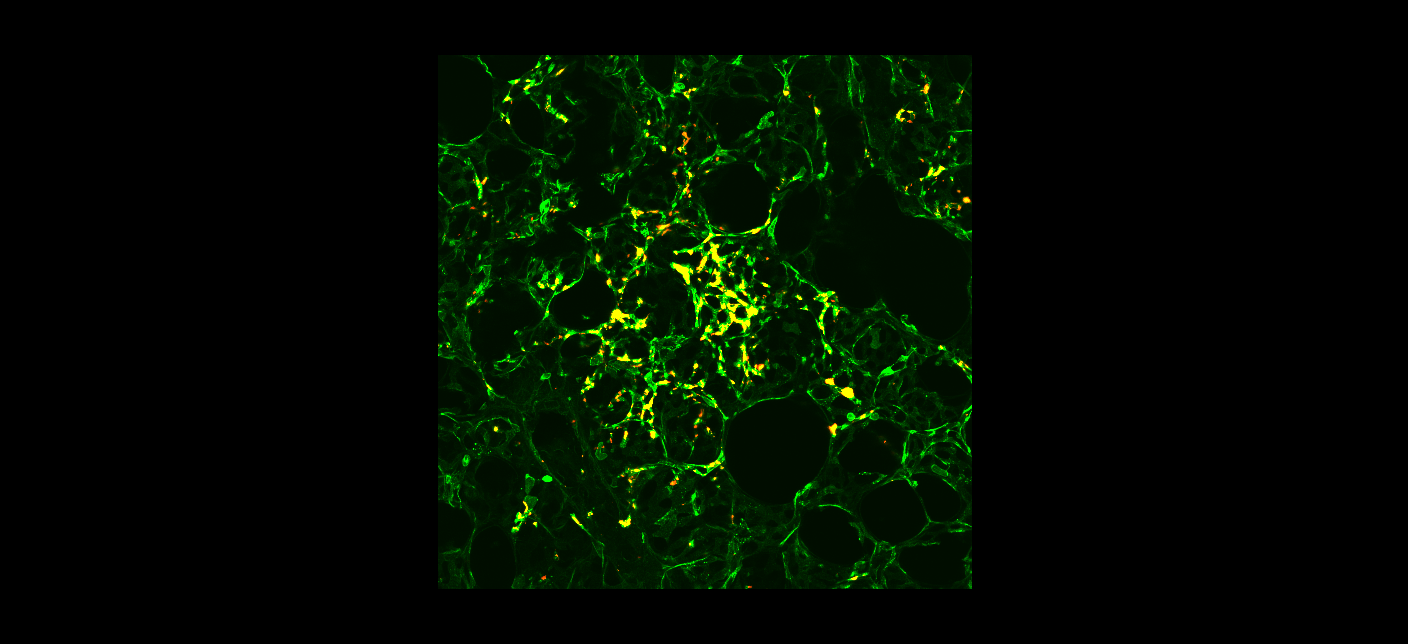

Supplement: Supplementary file 13 — Source Data Fig. 5 [file 44319_2023_54_MOESM13_ESM.zip › 5C/2_WT_Hx_Lectin-Green_Merge.bmp]

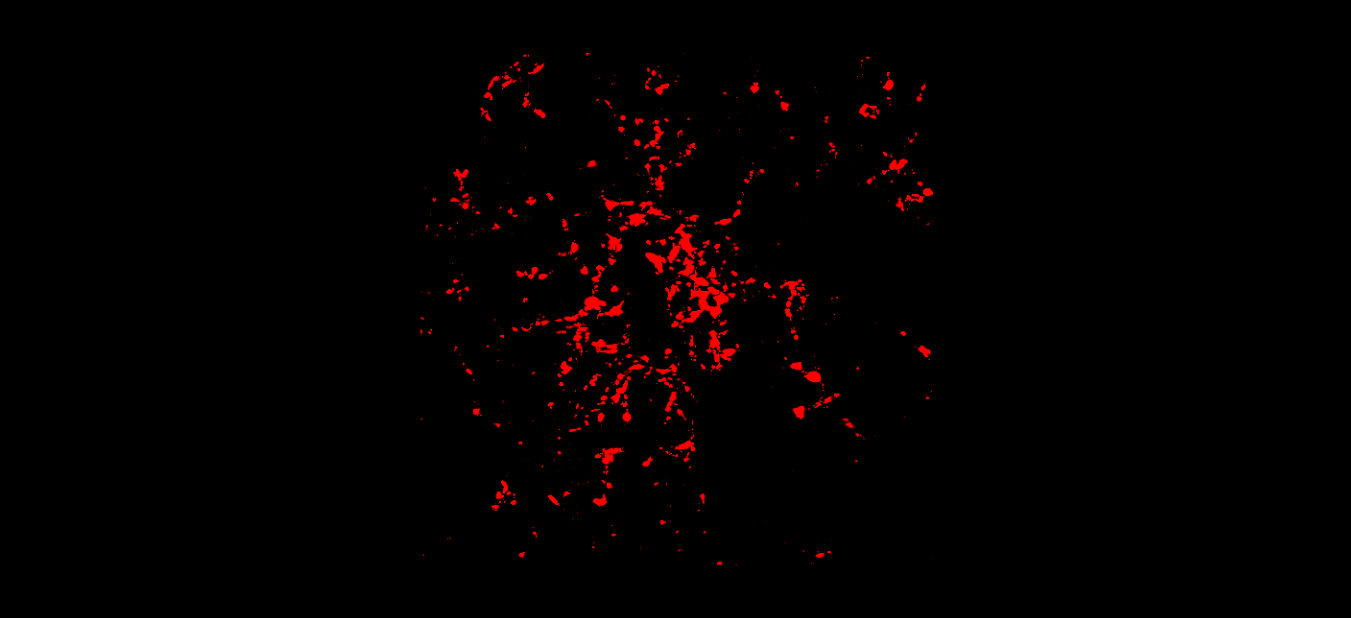

Supplement: Supplementary file 13 — Source Data Fig. 5 [file 44319_2023_54_MOESM13_ESM.zip › 5C/2_WT_Hx_Microbeads-Red.bmp]

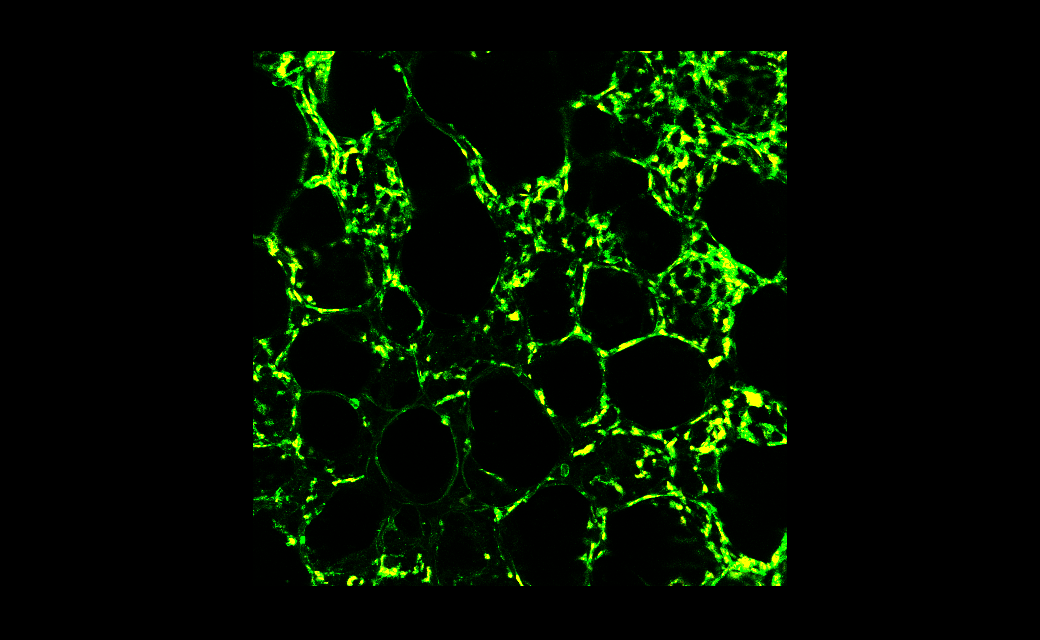

Supplement: Supplementary file 13 — Source Data Fig. 5 [file 44319_2023_54_MOESM13_ESM.zip › 5C/3_NG2-HIF2a_Normoxia_Lectin-Green_Merge.bmp]

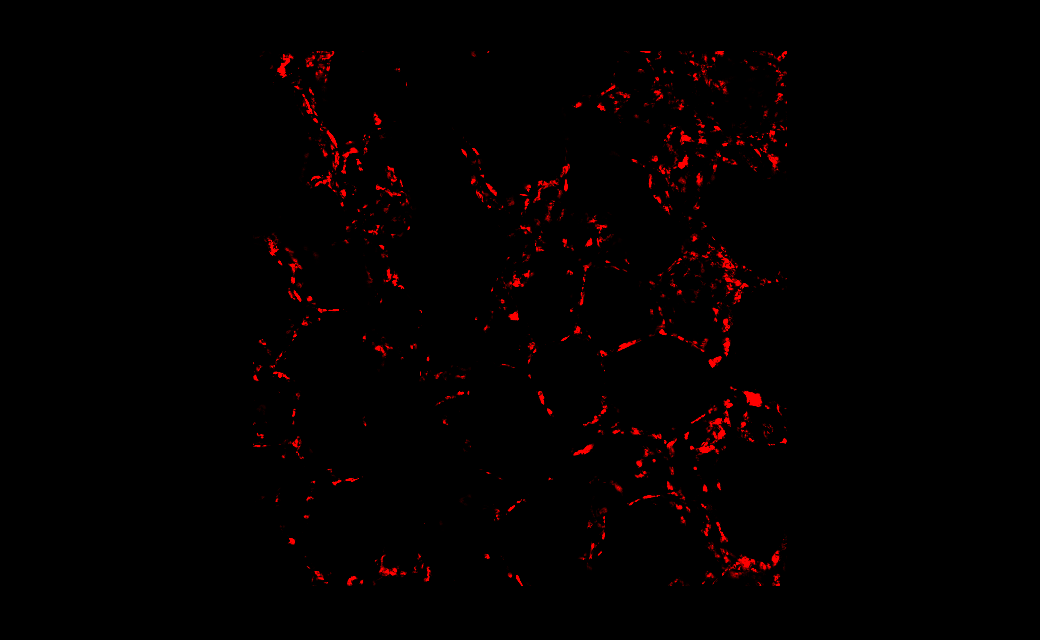

Supplement: Supplementary file 13 — Source Data Fig. 5 [file 44319_2023_54_MOESM13_ESM.zip › 5C/3_NG2-HIF2a_Normoxia_Microbeads-Red.bmp]

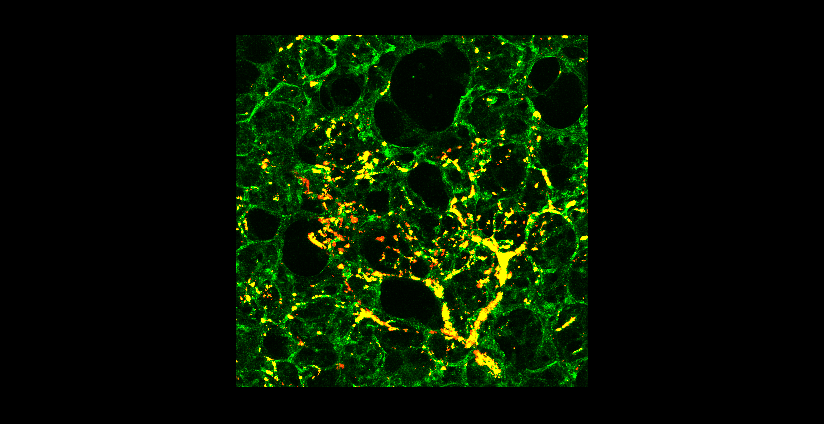

Supplement: Supplementary file 13 — Source Data Fig. 5 [file 44319_2023_54_MOESM13_ESM.zip › 5C/4_NG2-HIF2a_Hx_Lectin-Green_Merge.bmp]

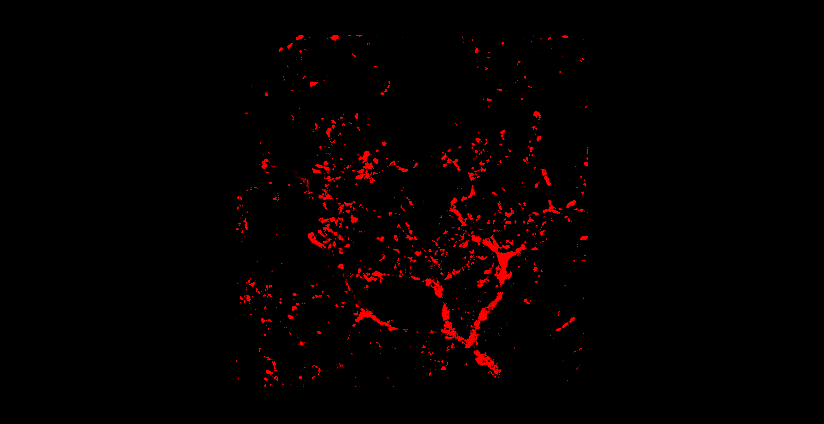

Supplement: Supplementary file 13 — Source Data Fig. 5 [file 44319_2023_54_MOESM13_ESM.zip › 5C/4_NG2-HIF2a_Hx_Microbeads-Red.bmp]

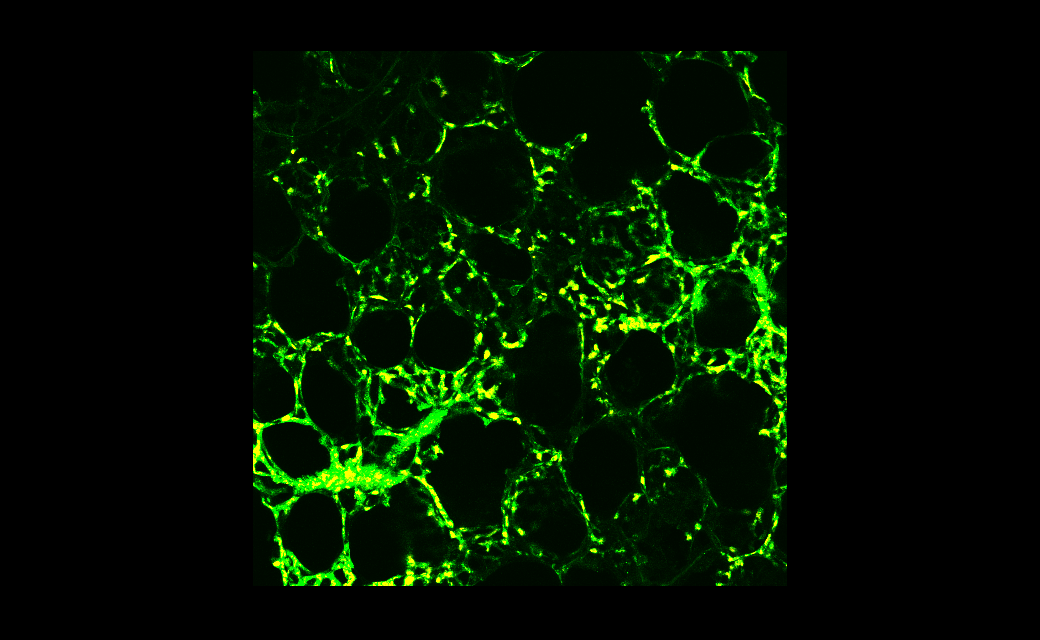

Supplement: Supplementary file 13 — Source Data Fig. 5 [file 44319_2023_54_MOESM13_ESM.zip › 5C/5_NG2-HIF2a KO_Normoxia_Lectin-Green_Merge.bmp]

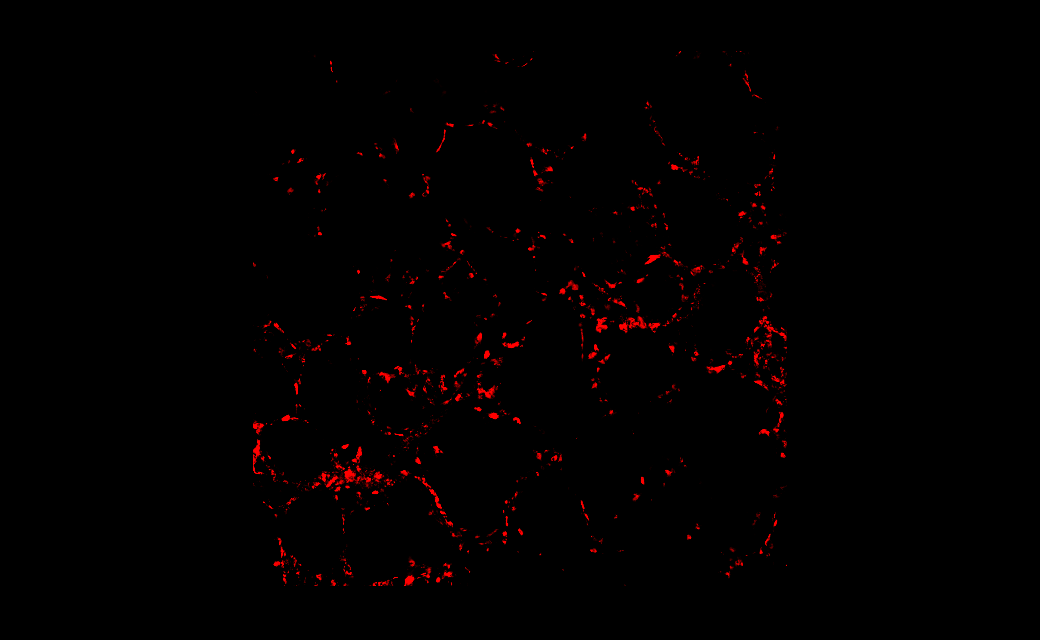

Supplement: Supplementary file 13 — Source Data Fig. 5 [file 44319_2023_54_MOESM13_ESM.zip › 5C/5_NG2-HIF2a KO_Normoxia_Microbeads-Red.bmp]

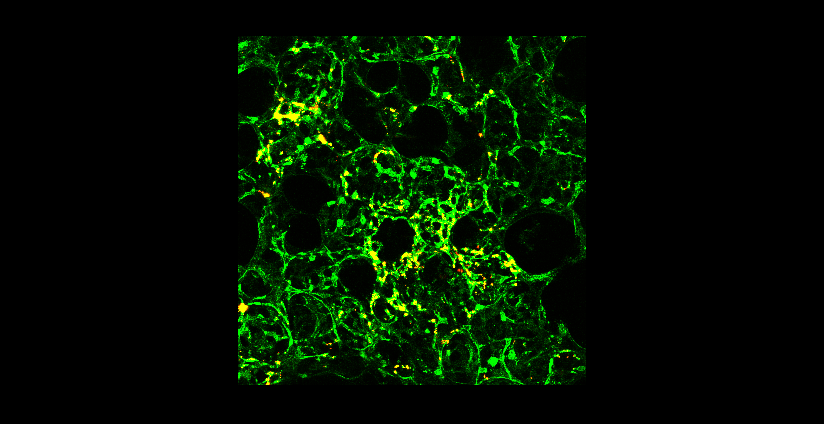

Supplement: Supplementary file 13 — Source Data Fig. 5 [file 44319_2023_54_MOESM13_ESM.zip › 5C/6_NG2-HIF2a KO_Hx_Lectin-Green_Merge.bmp]

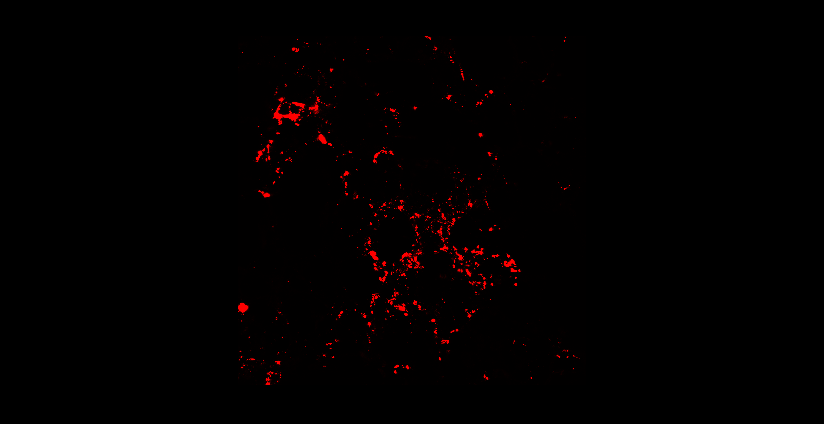

Supplement: Supplementary file 13 — Source Data Fig. 5 [file 44319_2023_54_MOESM13_ESM.zip › 5C/6_NG2-HIF2a KO_Hx_Microbeads-Red.bmp]

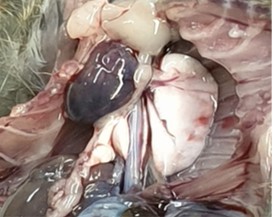

Supplement: Supplementary file 13 — Source Data Fig. 5 [file 44319_2023_54_MOESM13_ESM.zip › 5E/1_WT_Norm.jpg]

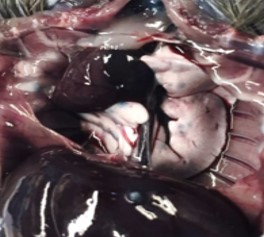

Supplement: Supplementary file 13 — Source Data Fig. 5 [file 44319_2023_54_MOESM13_ESM.zip › 5E/2_WT_Hx.jpg]

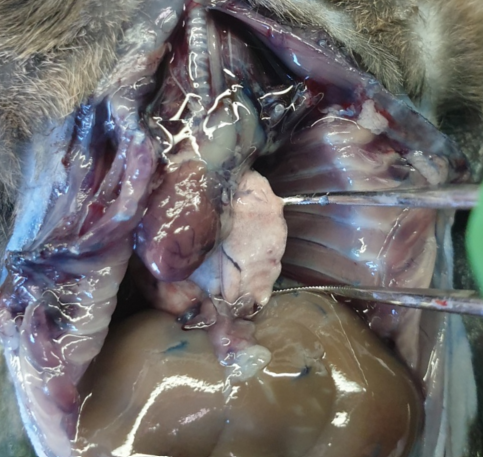

Supplement: Supplementary file 13 — Source Data Fig. 5 [file 44319_2023_54_MOESM13_ESM.zip › 5E/3_NG2 HIF2a OE_Norm.png]

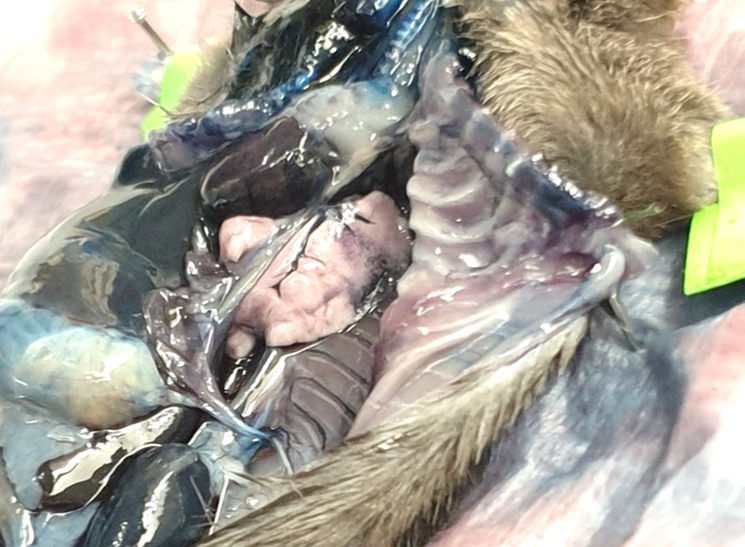

Supplement: Supplementary file 13 — Source Data Fig. 5 [file 44319_2023_54_MOESM13_ESM.zip › 5E/4_NG2 HIF2a OE_Hx.tif]

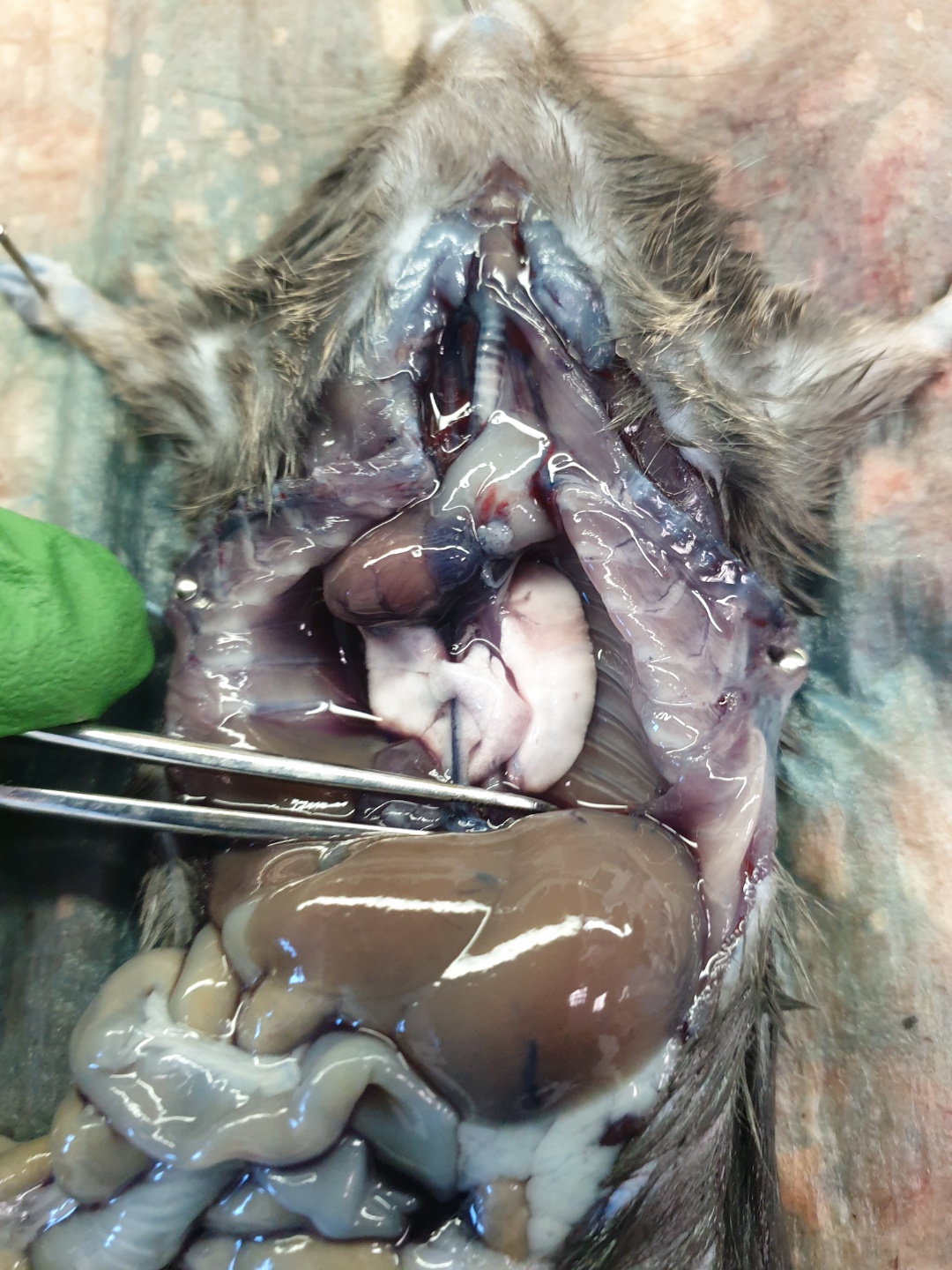

Supplement: Supplementary file 13 — Source Data Fig. 5 [file 44319_2023_54_MOESM13_ESM.zip › 5E/5_NG2 HIF2a KO_Norm.jpg]

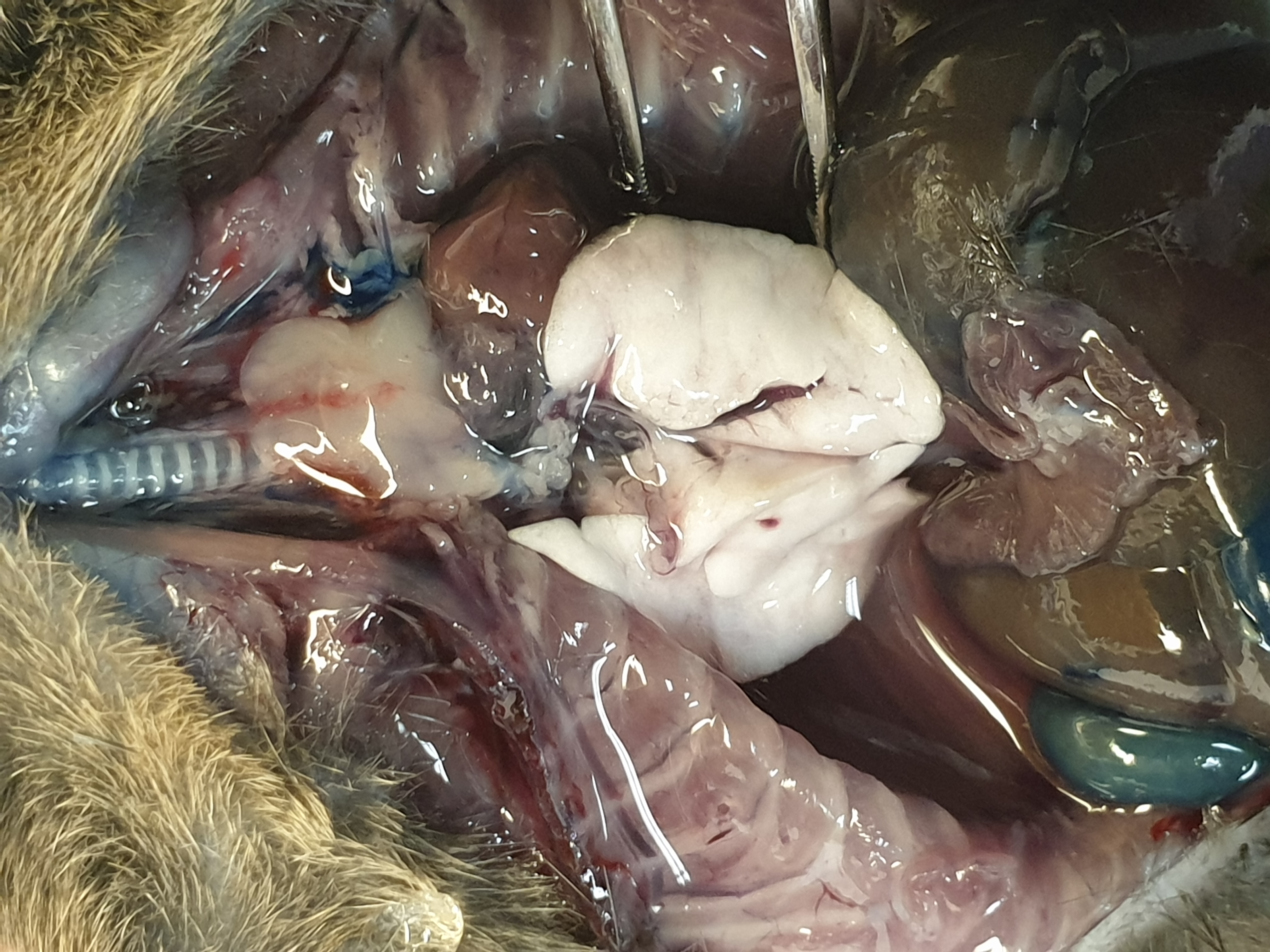

Supplement: Supplementary file 13 — Source Data Fig. 5 [file 44319_2023_54_MOESM13_ESM.zip › 5E/6_NG2 HIF2a KO_Hx.jpg]

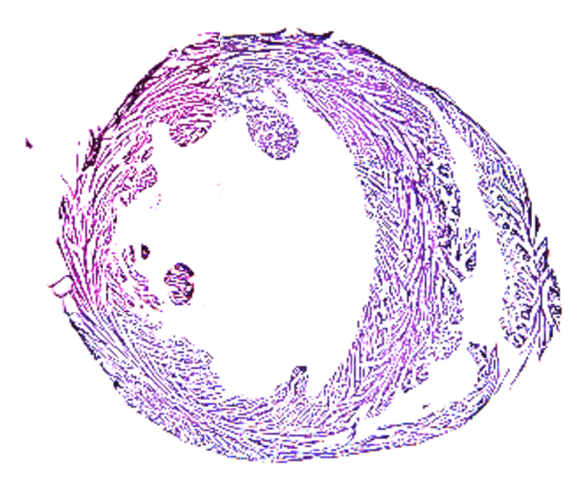

Supplement: Supplementary file 14 — Source Data Fig. 6 [file 44319_2023_54_MOESM14_ESM.zip › 6F/1_WT norm/NG2-Wt_Normoxia.png]

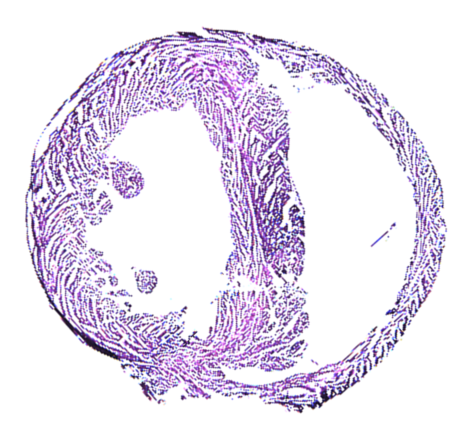

Supplement: Supplementary file 14 — Source Data Fig. 6 [file 44319_2023_54_MOESM14_ESM.zip › 6F/2_WT Hx/NG2-Wt_3wk Hx.png]

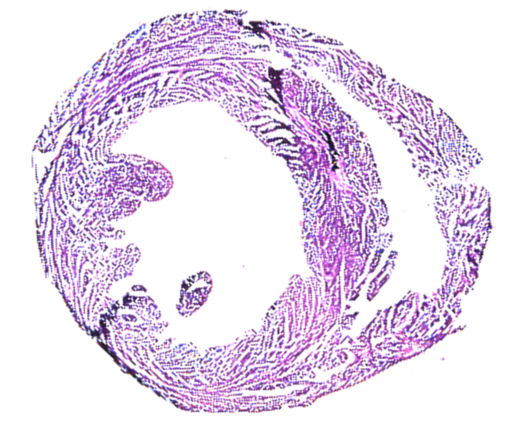

Supplement: Supplementary file 14 — Source Data Fig. 6 [file 44319_2023_54_MOESM14_ESM.zip › 6F/3_NG2 HIF2a OE Norm/NG2-HIF2a OE_Normoxia.png]

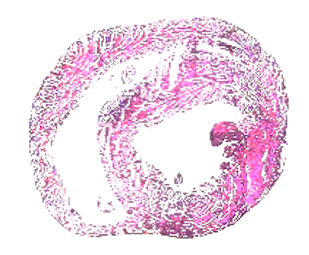

Supplement: Supplementary file 14 — Source Data Fig. 6 [file 44319_2023_54_MOESM14_ESM.zip › 6F/4_NG2 HIF2a OE Hx/NG2-HIF2a OE_3wk Hx.png]

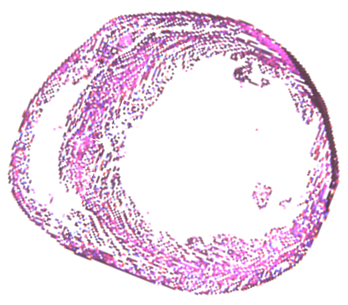

Supplement: Supplementary file 14 — Source Data Fig. 6 [file 44319_2023_54_MOESM14_ESM.zip › 6F/5_NG2 HIF2a KO Norm/NG2-HIF2a KO_Normoxia.png]

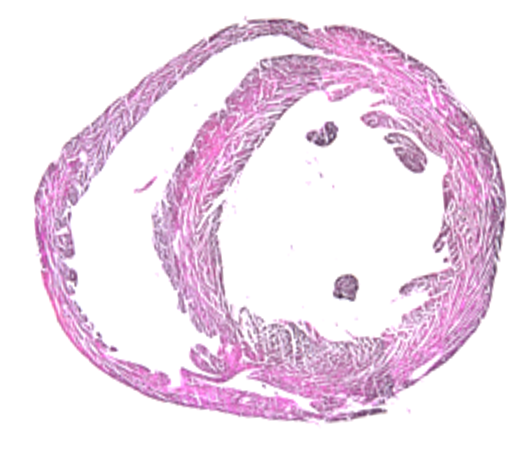

Supplement: Supplementary file 14 — Source Data Fig. 6 [file 44319_2023_54_MOESM14_ESM.zip › 6F/6_NG2 HIF2a KO Hx/NG2-HIF2a KO_3wk Hx.png]

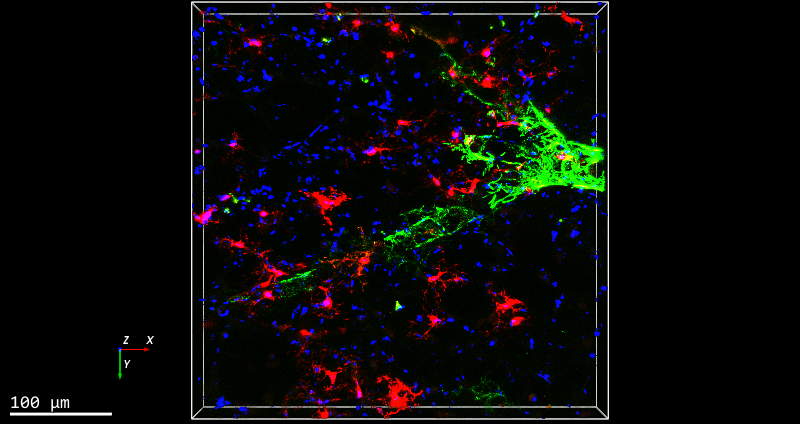

Supplement: Supplementary file 15 — Source Data Fig. 7 [file 44319_2023_54_MOESM15_ESM.zip › 7C/Day 0.bmp]

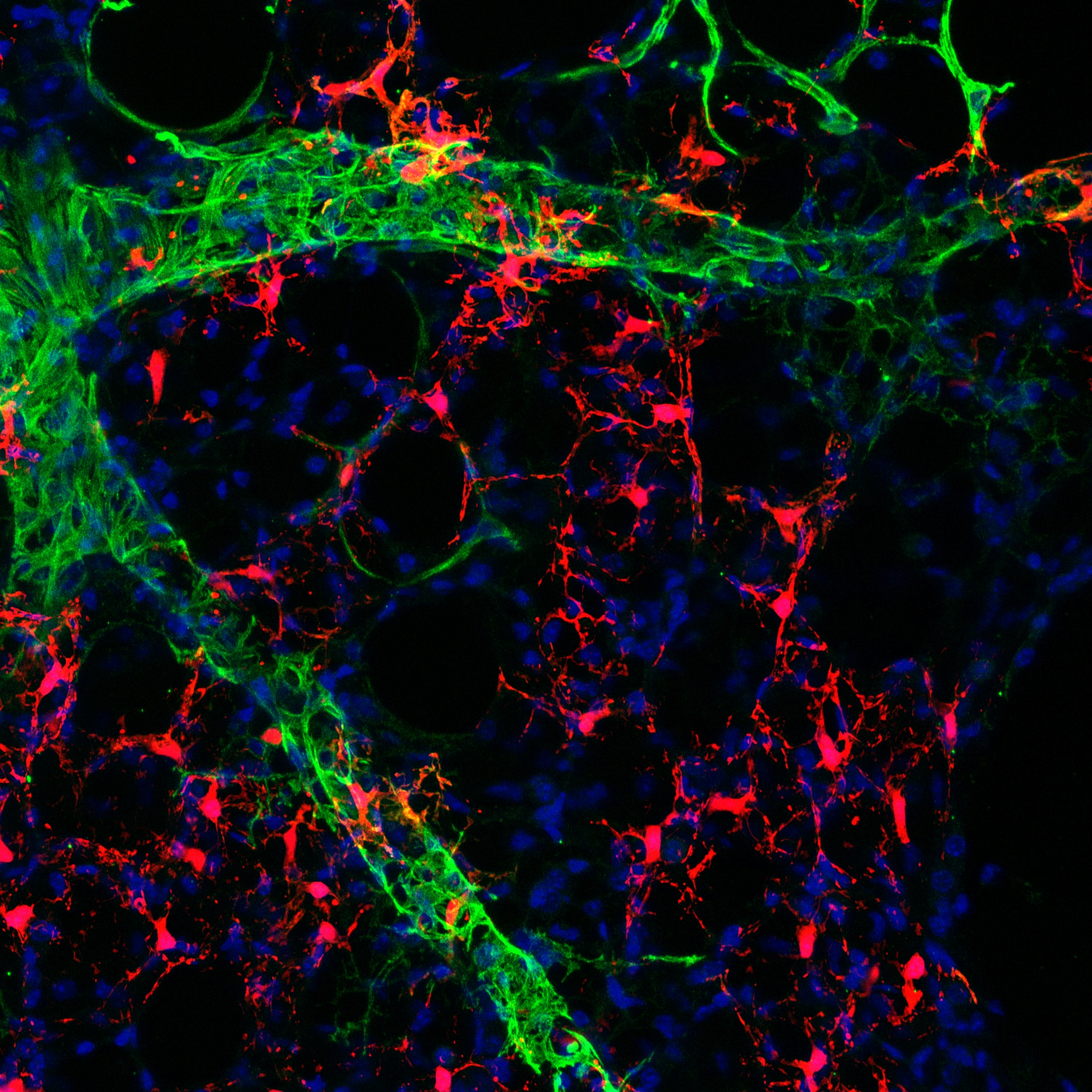

Supplement: Supplementary file 15 — Source Data Fig. 7 [file 44319_2023_54_MOESM15_ESM.zip › 7C/Day 14.jpg]

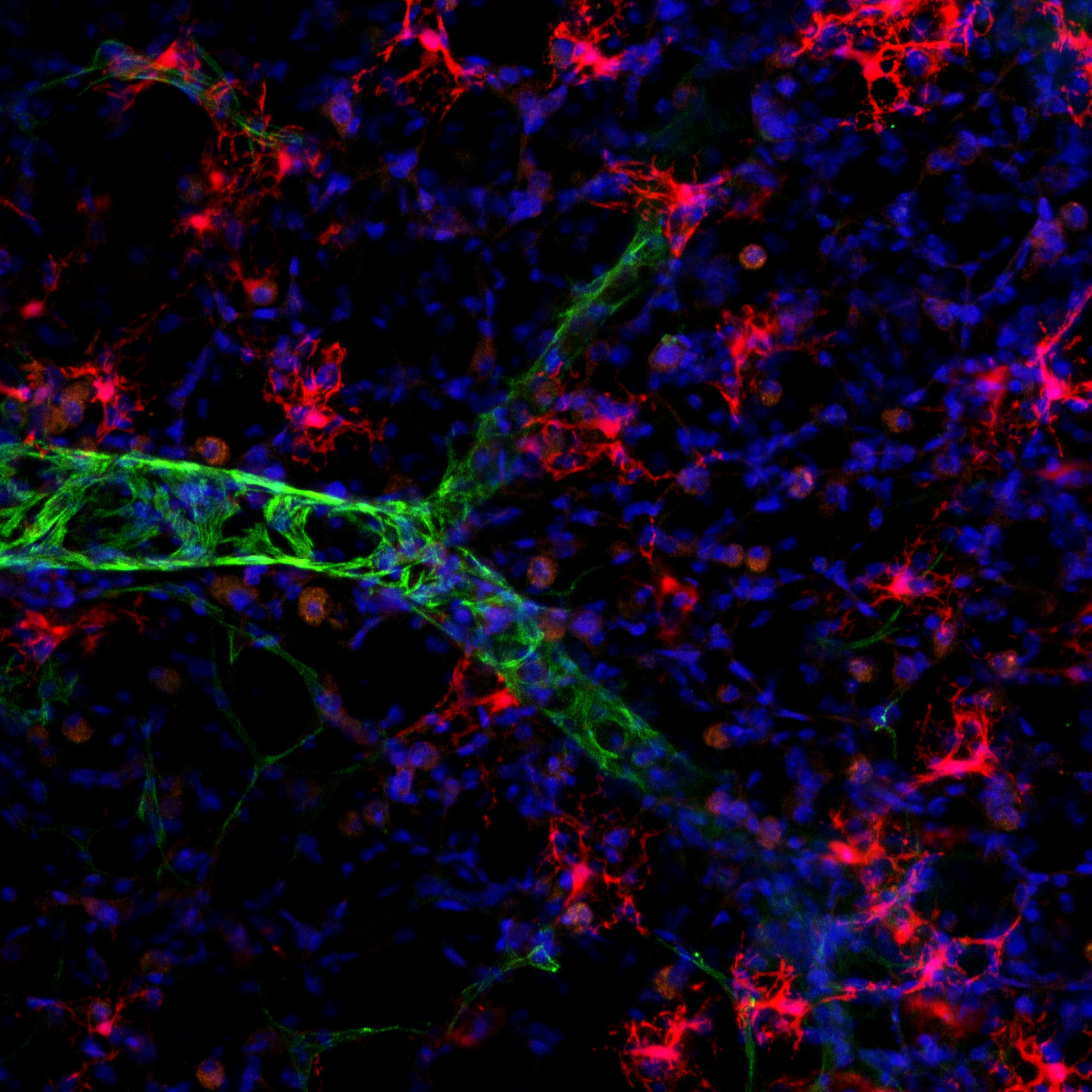

Supplement: Supplementary file 15 — Source Data Fig. 7 [file 44319_2023_54_MOESM15_ESM.zip › 7C/Day 2.jpg]

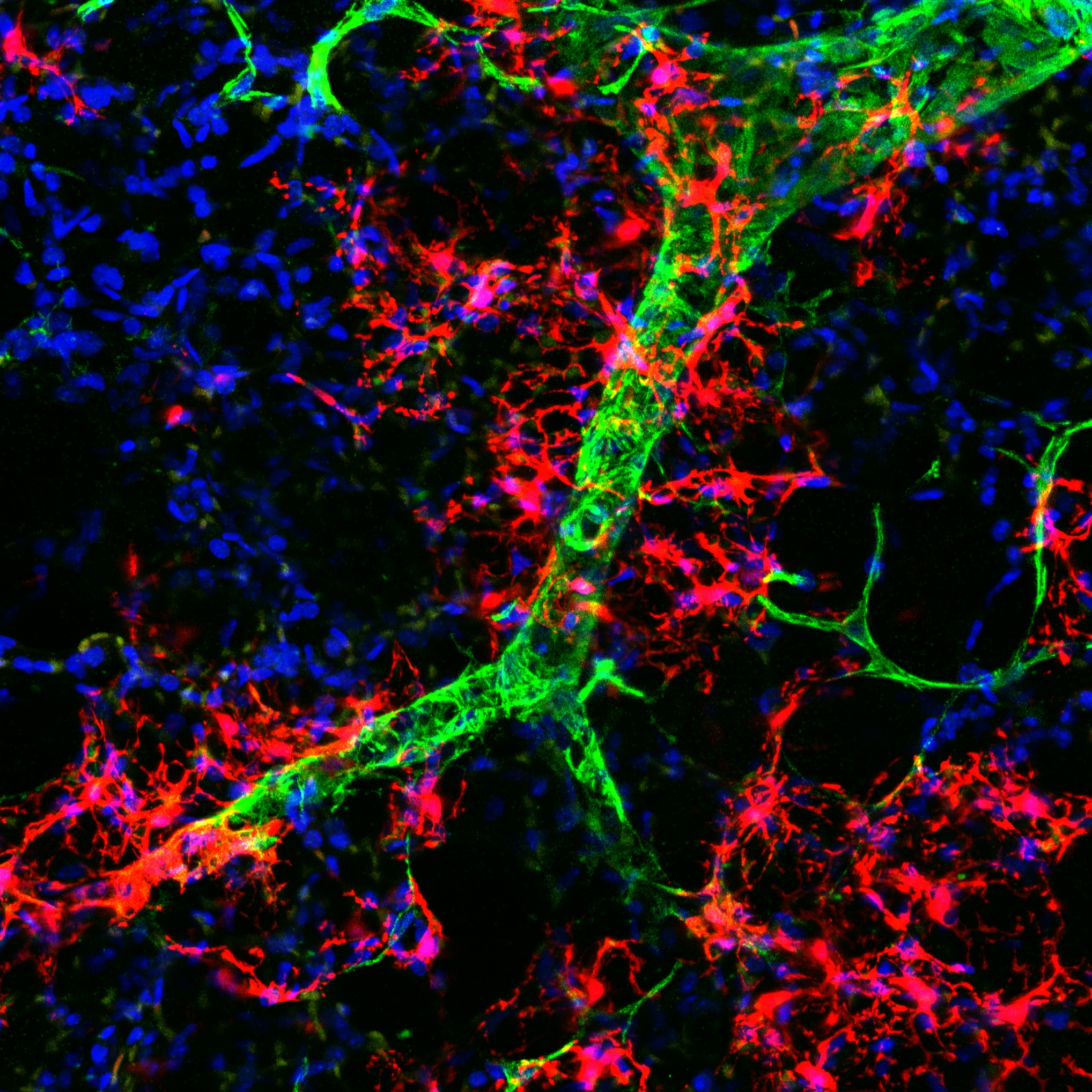

Supplement: Supplementary file 15 — Source Data Fig. 7 [file 44319_2023_54_MOESM15_ESM.zip › 7C/Day 21.jpg]

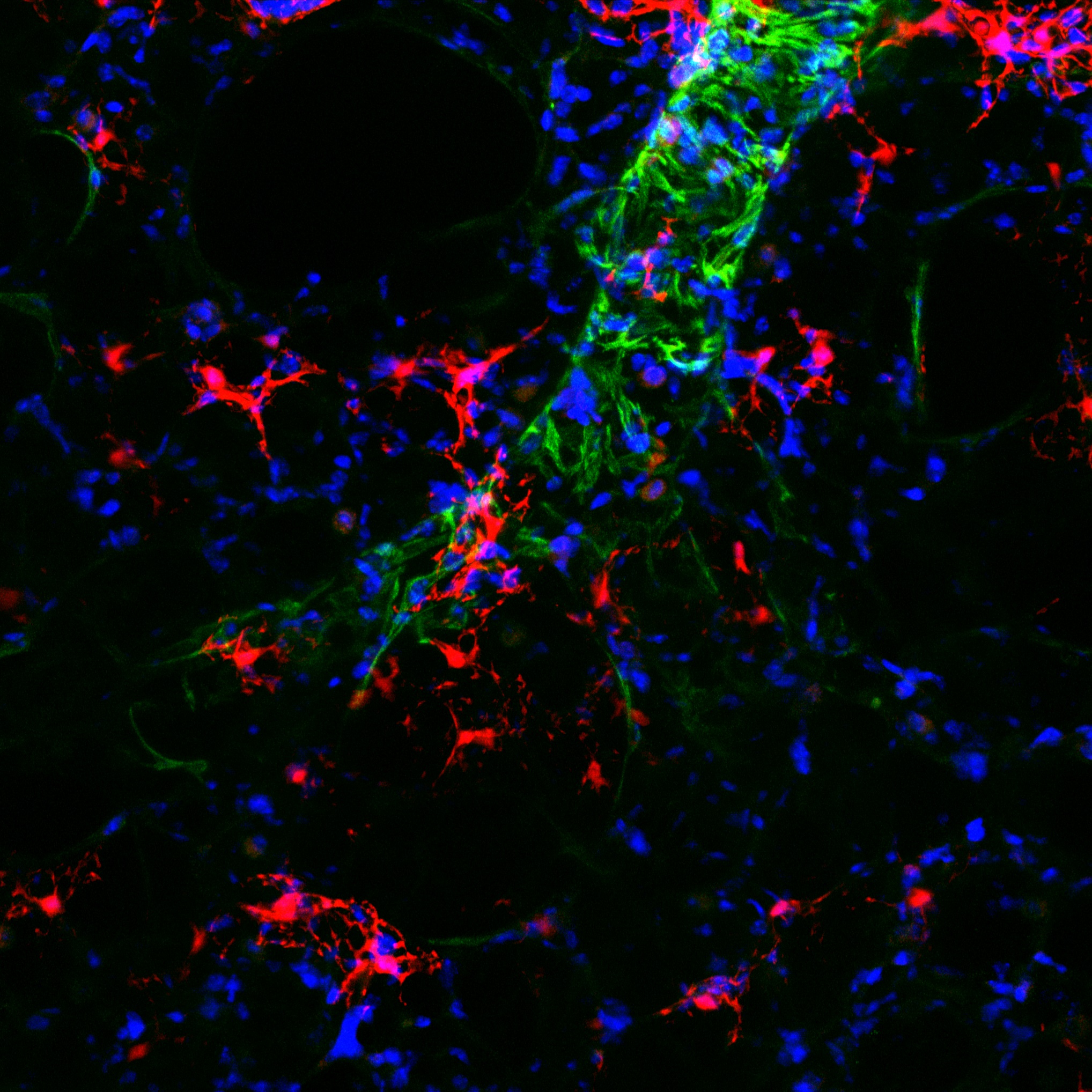

Supplement: Supplementary file 15 — Source Data Fig. 7 [file 44319_2023_54_MOESM15_ESM.zip › 7C/Day 4.jpg]

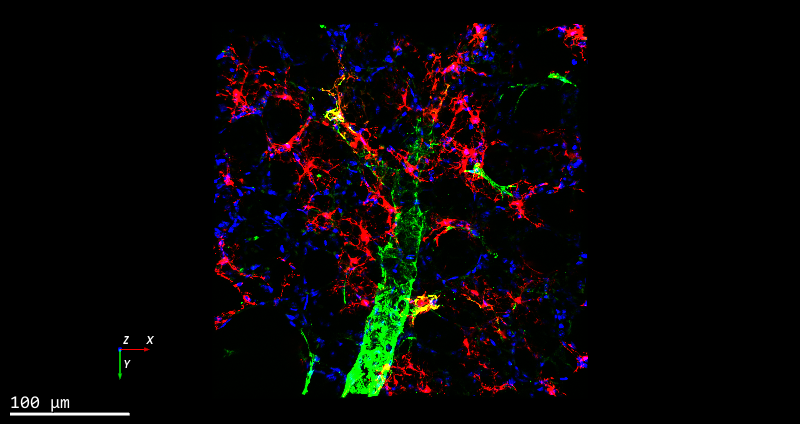

Supplement: Supplementary file 15 — Source Data Fig. 7 [file 44319_2023_54_MOESM15_ESM.zip › 7C/Day 7.bmp]

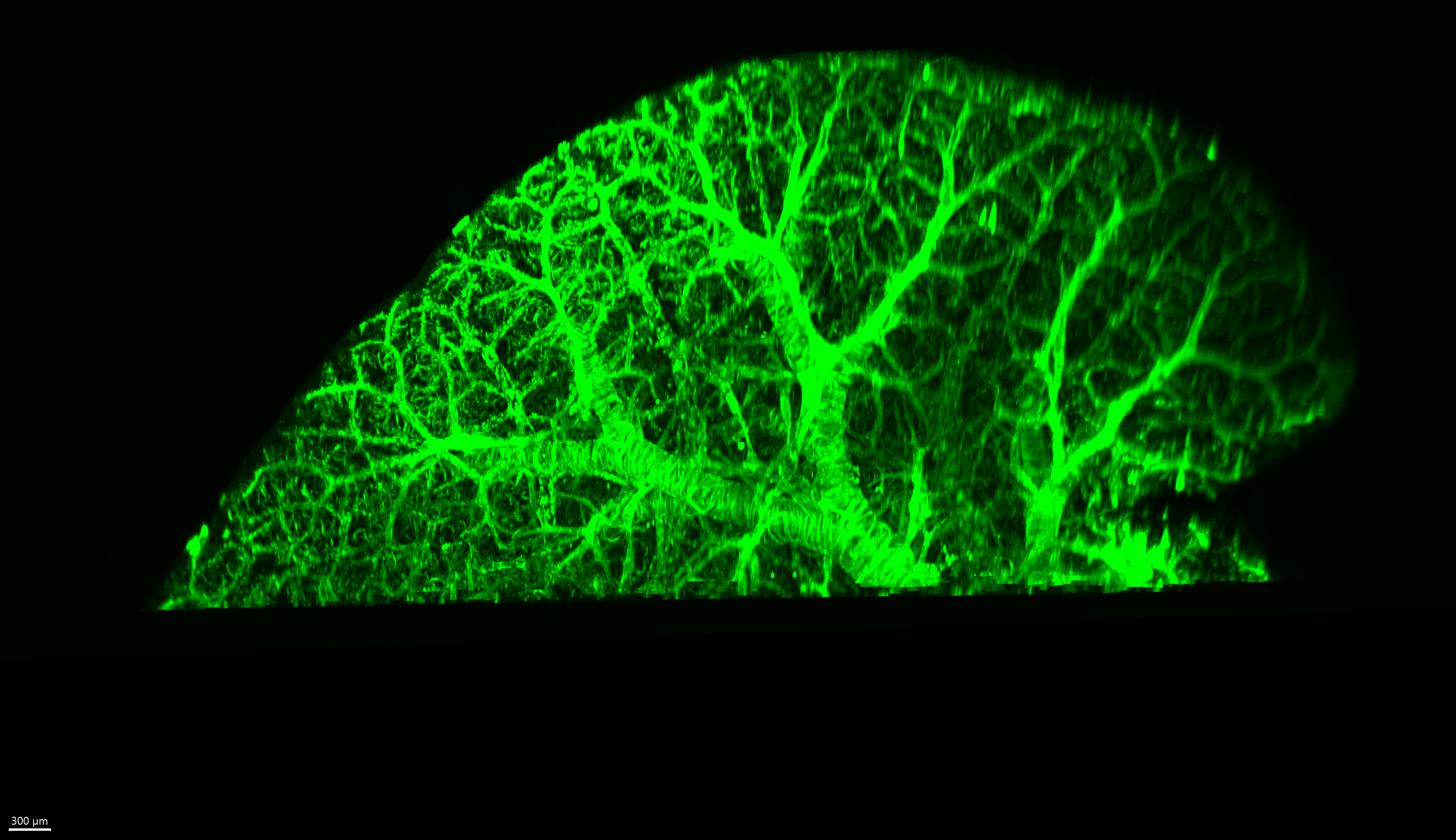

Supplement: Supplementary file 15 — Source Data Fig. 7 [file 44319_2023_54_MOESM15_ESM.zip › 7D/Fig 7D-NG2hif2a3wkHx_1.tif]

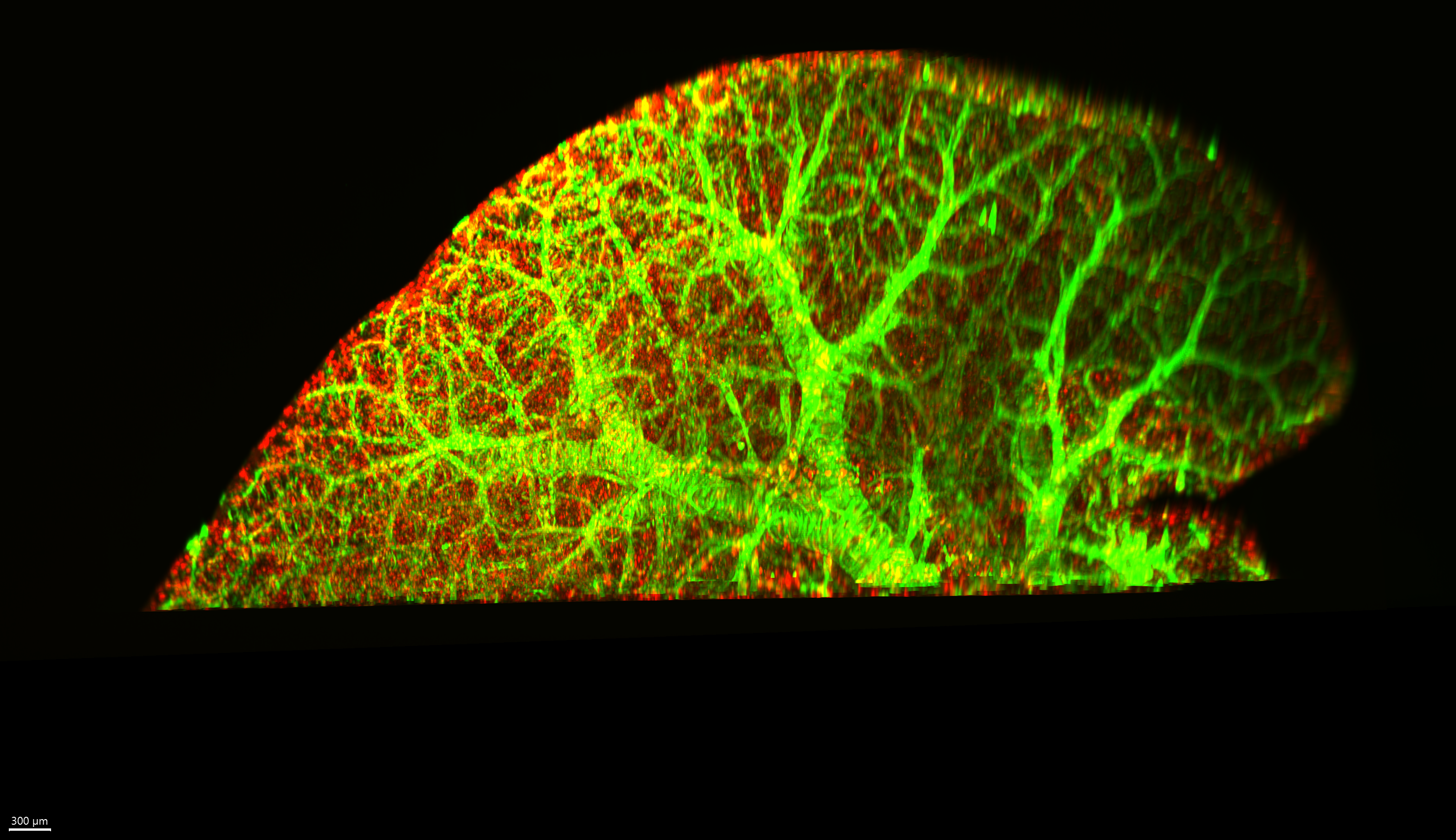

Supplement: Supplementary file 15 — Source Data Fig. 7 [file 44319_2023_54_MOESM15_ESM.zip › 7D/Fig7D-Hx-NG2hif2a3wkHx_.tif]

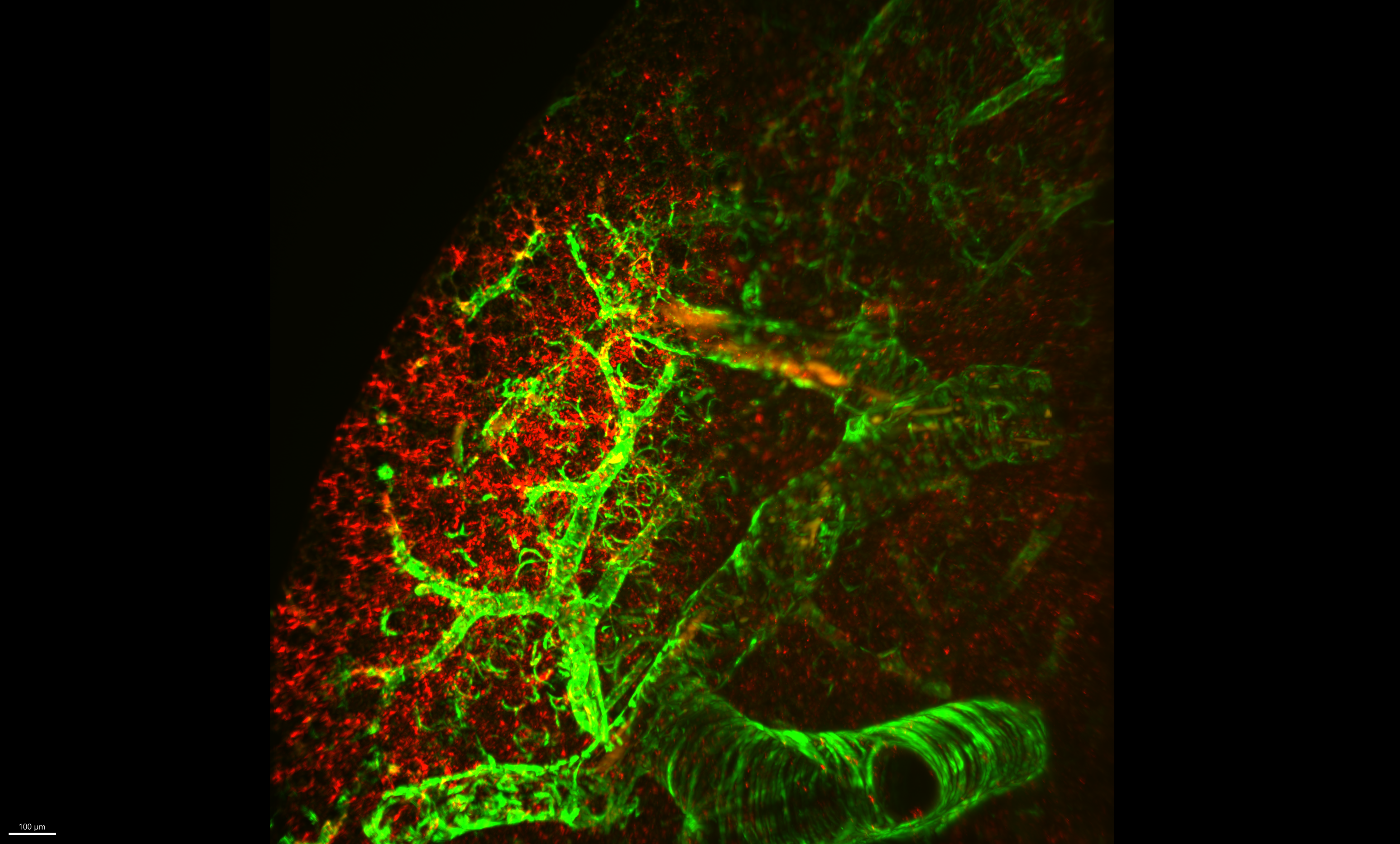

Supplement: Supplementary file 15 — Source Data Fig. 7 [file 44319_2023_54_MOESM15_ESM.zip › 7D/Fig7D-ng2hif2atdT-3wkhx_3.tif]

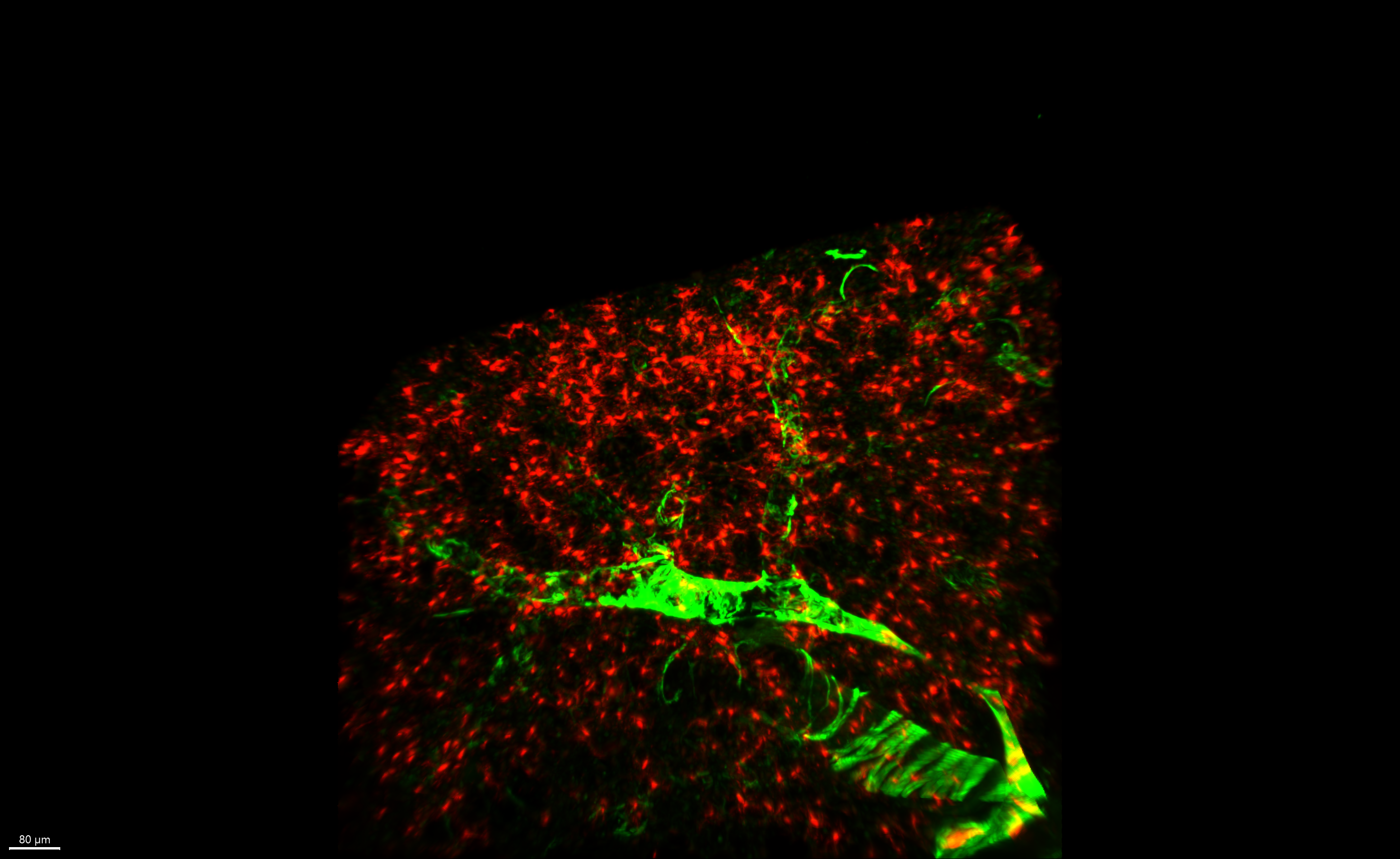

Supplement: Supplementary file 15 — Source Data Fig. 7 [file 44319_2023_54_MOESM15_ESM.zip › 7D/Fig7D-NG2HIF2atdTnorm-3.tif]

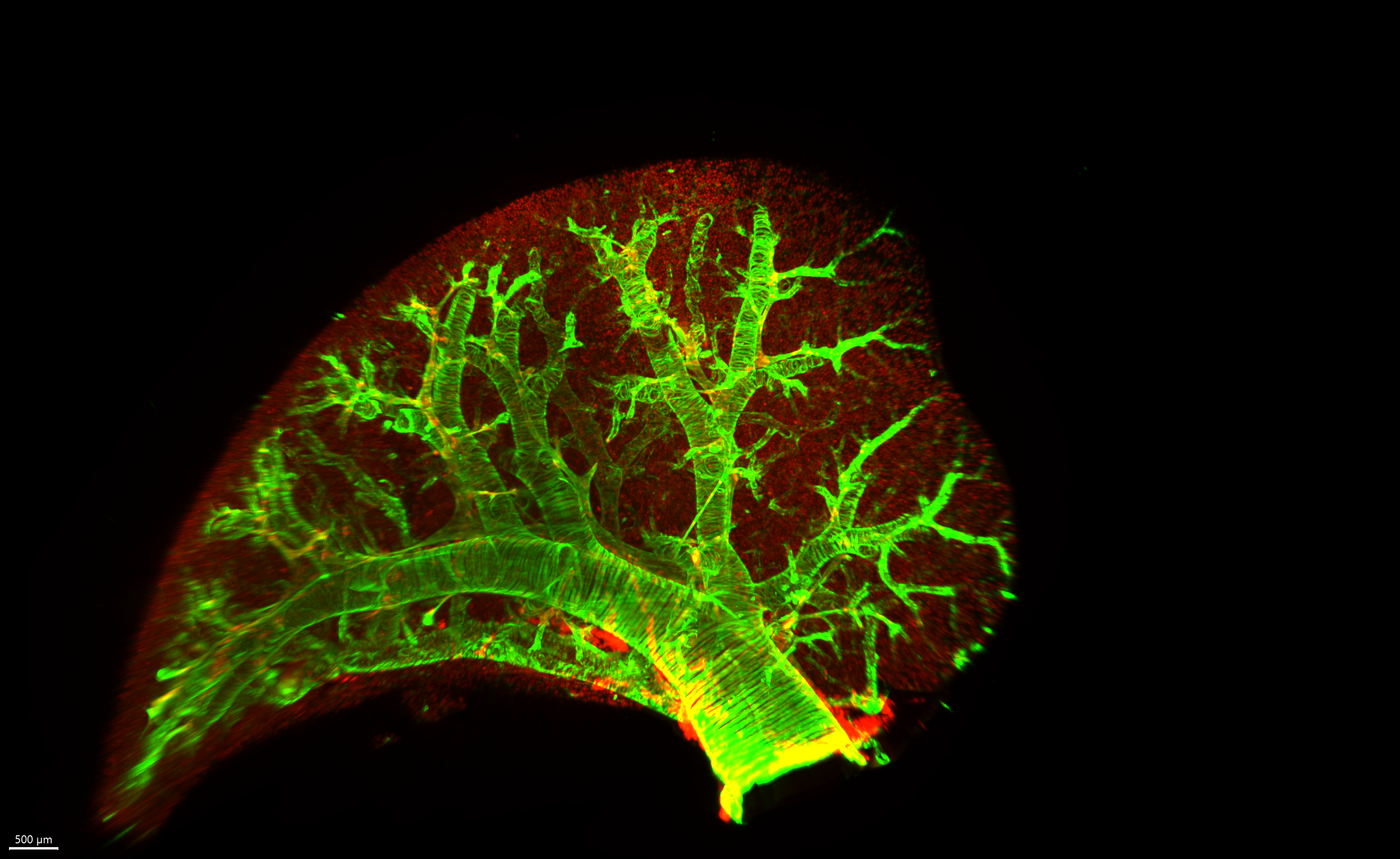

Supplement: Supplementary file 15 — Source Data Fig. 7 [file 44319_2023_54_MOESM15_ESM.zip › 7D/Fig7D-NG2HIF2atdTnorm_.tif]

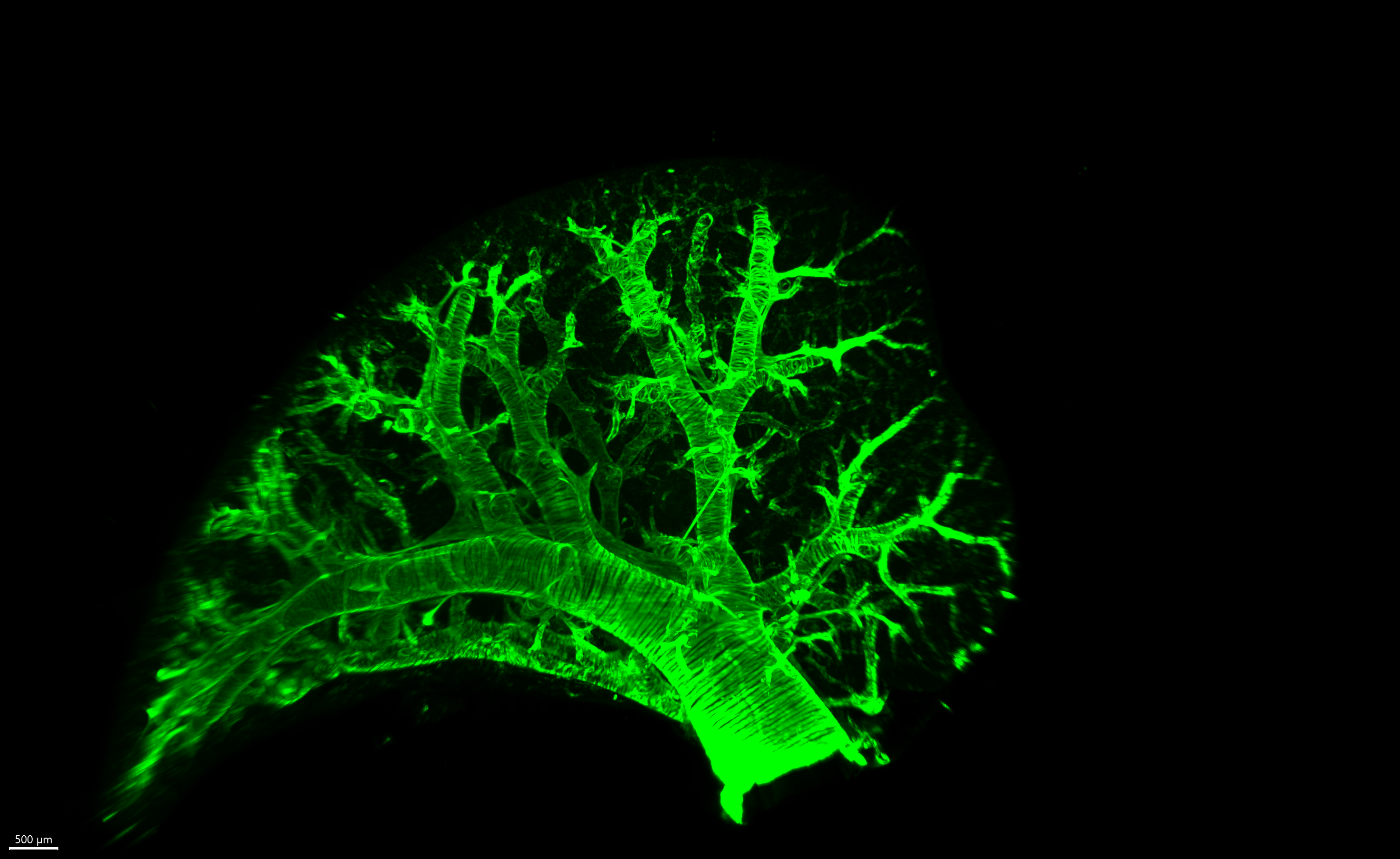

Supplement: Supplementary file 15 — Source Data Fig. 7 [file 44319_2023_54_MOESM15_ESM.zip › 7D/Fig7D-NG2HIF2atdTnorm_1.tif]

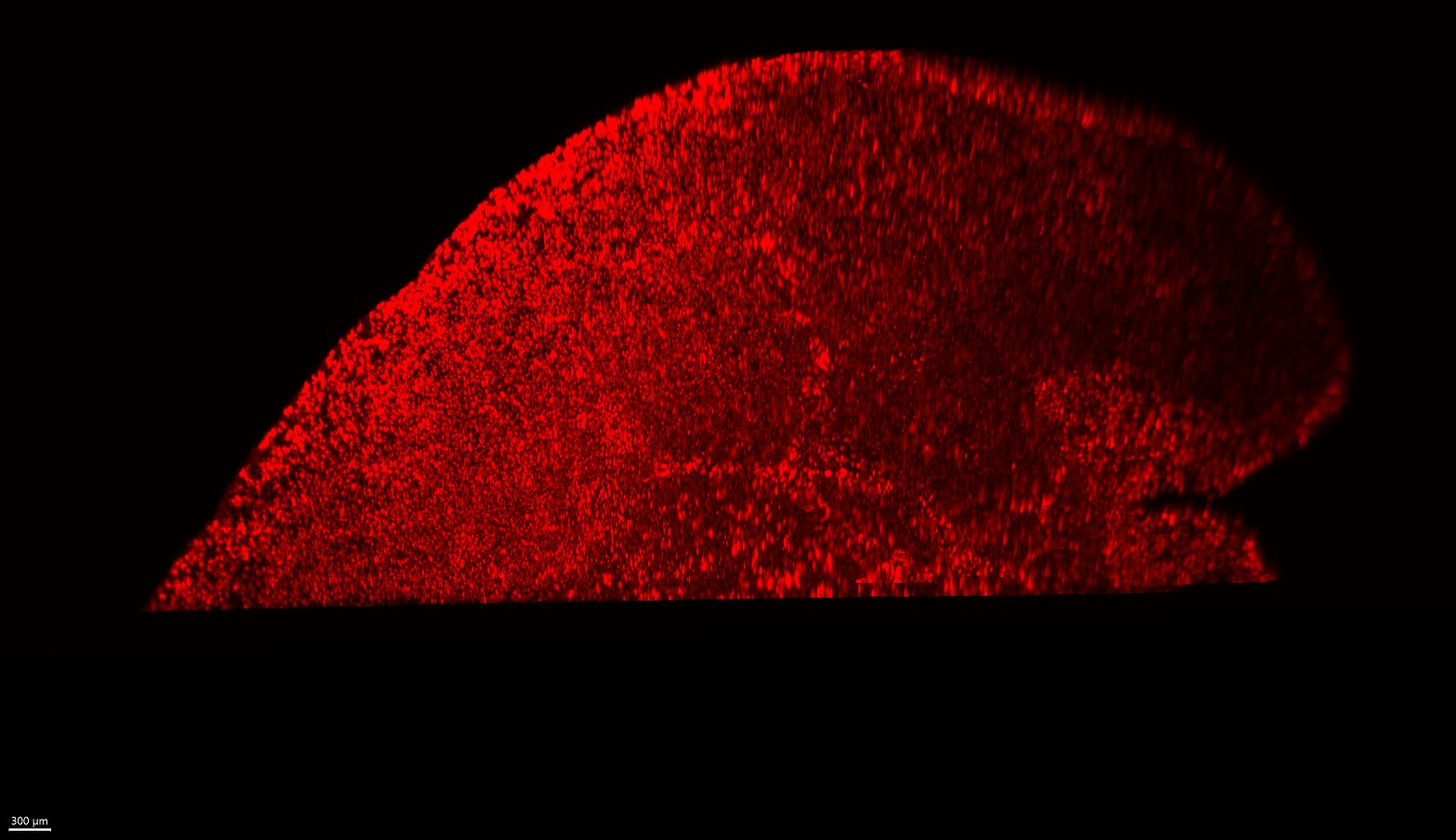

Supplement: Supplementary file 15 — Source Data Fig. 7 [file 44319_2023_54_MOESM15_ESM.zip › 7D/NG2hif2a3wkHx_C00_z0000.ome_2021-06-30T10-26-34.893.tif]

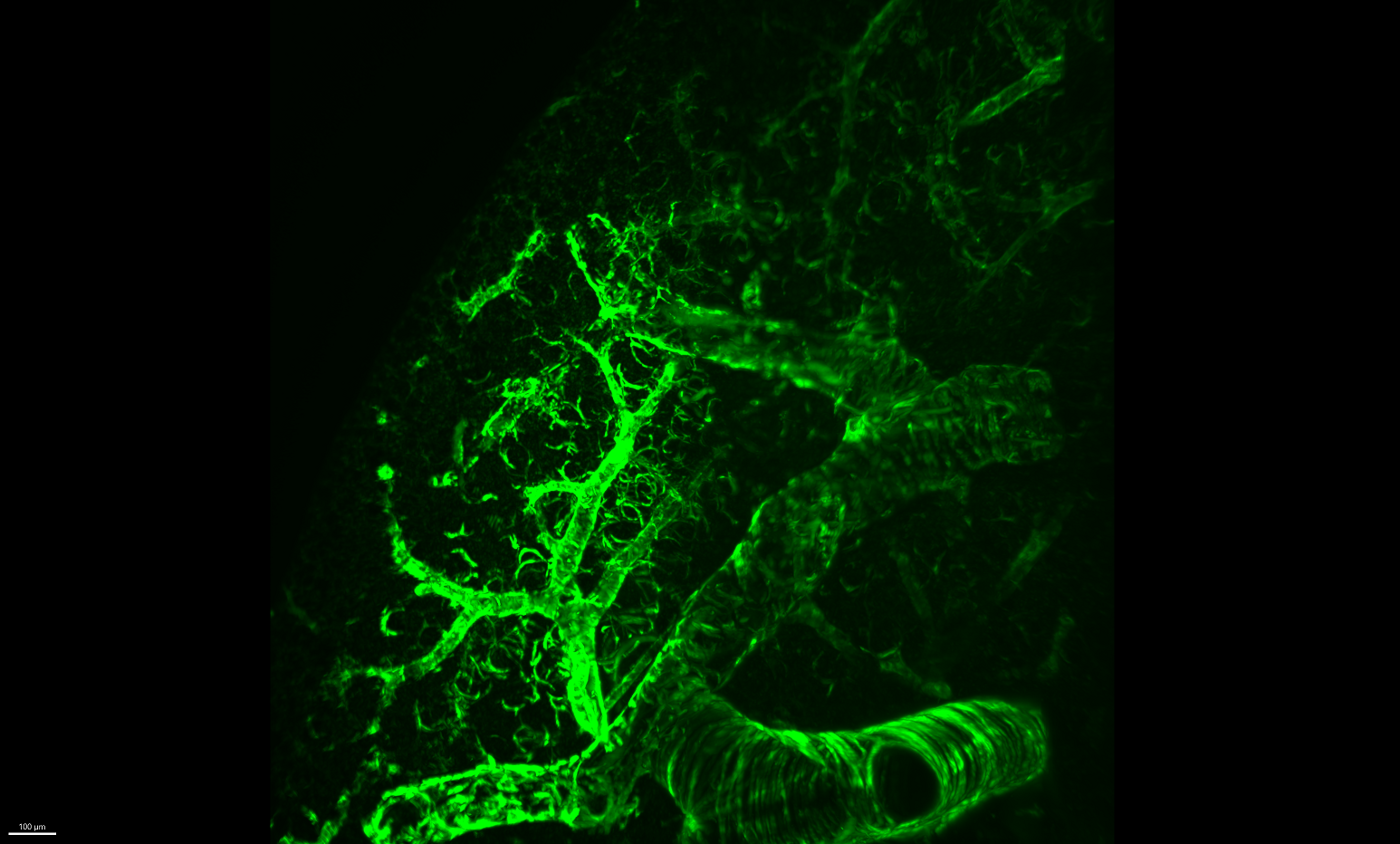

Supplement: Supplementary file 15 — Source Data Fig. 7 [file 44319_2023_54_MOESM15_ESM.zip › 7D/ng2hif2a6wkhx_c00_z0000.ome_1_2021-06-30T13-43-27.591.tif]
