# Supplementary figures and images for: Pericytes contribute to pulmonary vascular remodeling via HIF2α signaling (part 2 of 2)
Source: EMBO Rep. 2024 Jan 19;25(2):13. doi: 10.1038/s44319-023-00054-w (PMC10897382; doi:10.1038/s44319-023-00054-w)

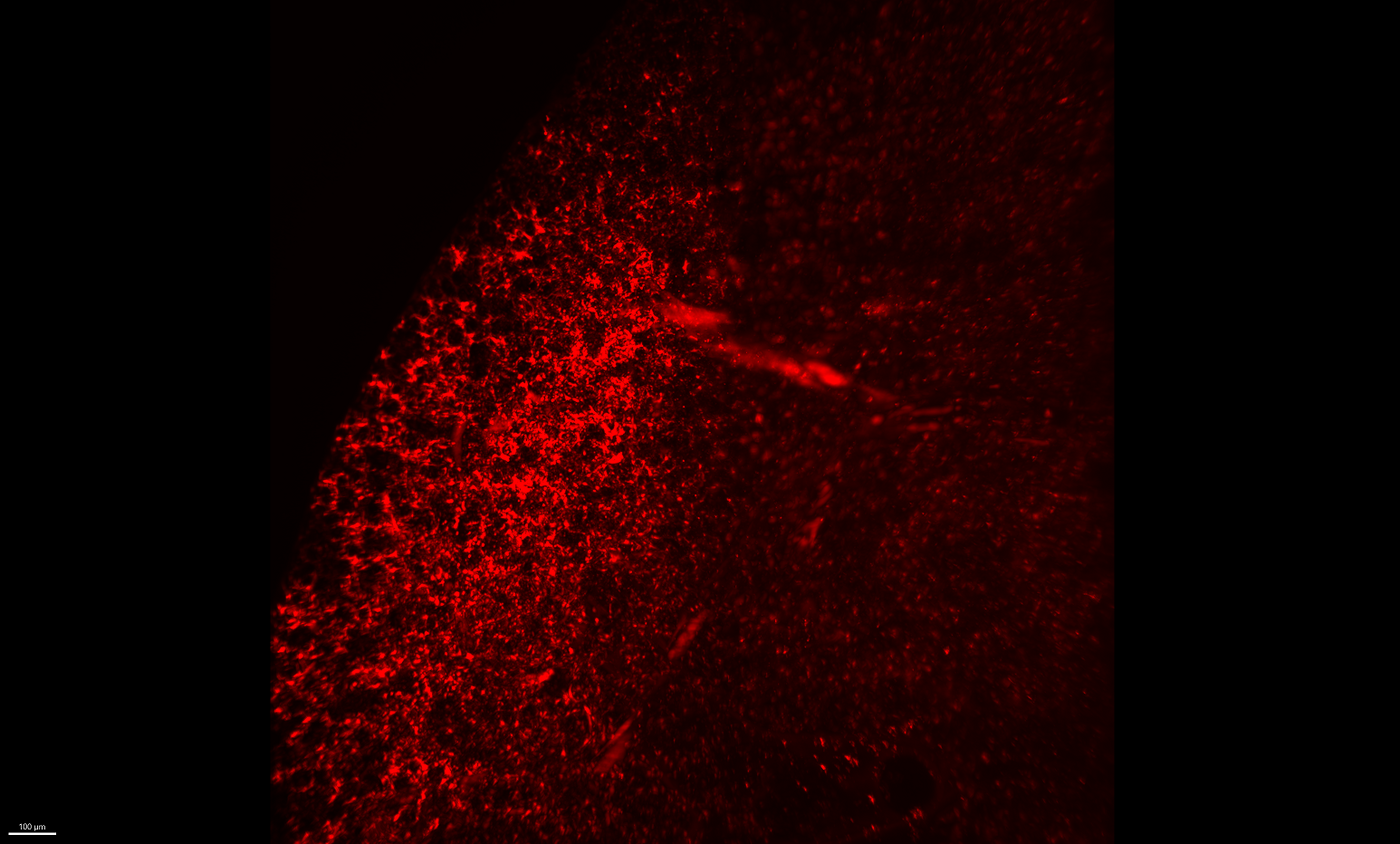

Supplement: Supplementary file 15 — Source Data Fig. 7 [file 44319_2023_54_MOESM15_ESM.zip › 7D/ng2hif2a6wkhx_c00_z0000.ome_1_2021-06-30T13-43-39.356.tif]

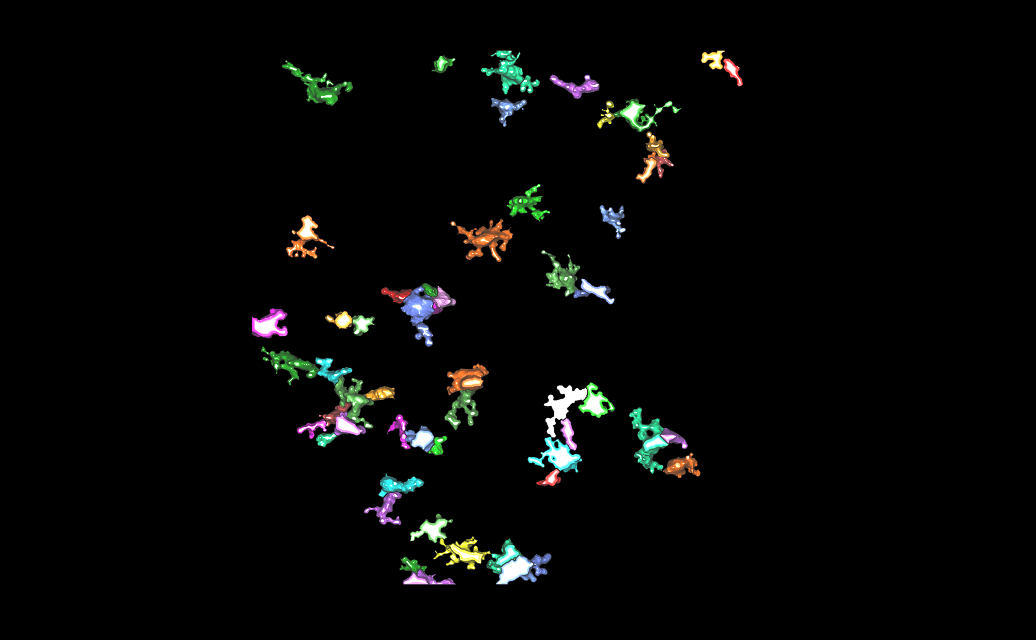

Supplement: Supplementary file 15 — Source Data Fig. 7 [file 44319_2023_54_MOESM15_ESM.zip › 7E/1. Day 0/3_Normoxia_4mg TAM_20x_NG2_HIF2a_SMA_4_Airyscan Processing_z15_t000.bmp]

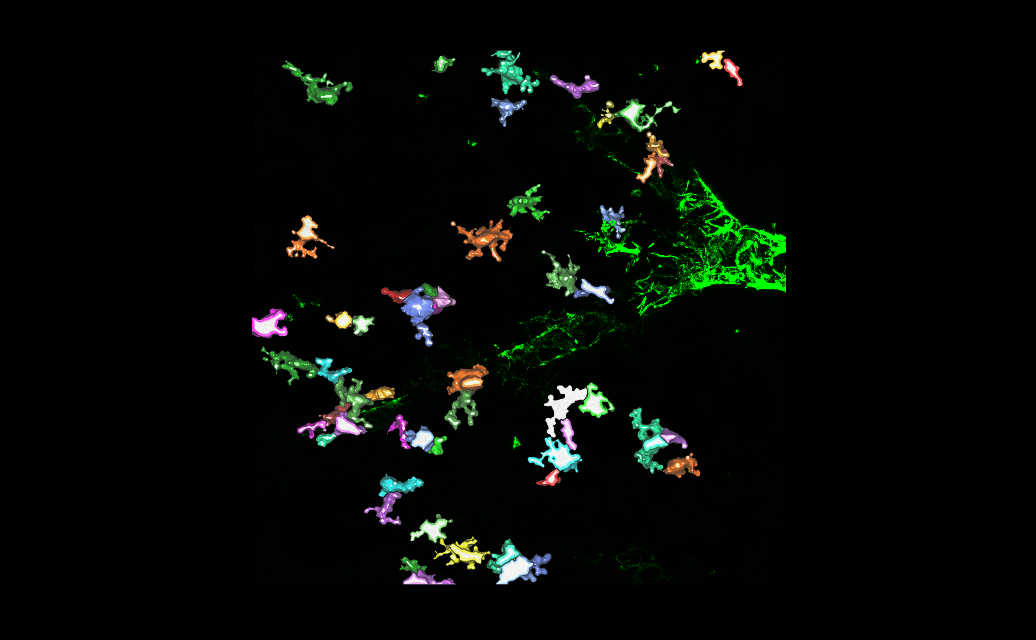

Supplement: Supplementary file 15 — Source Data Fig. 7 [file 44319_2023_54_MOESM15_ESM.zip › 7E/1. Day 0/3_Normoxia_4mg TAM_20x_NG2_HIF2a_SMA_4_Airyscan Processing_z15_t000_SMA.bmp]

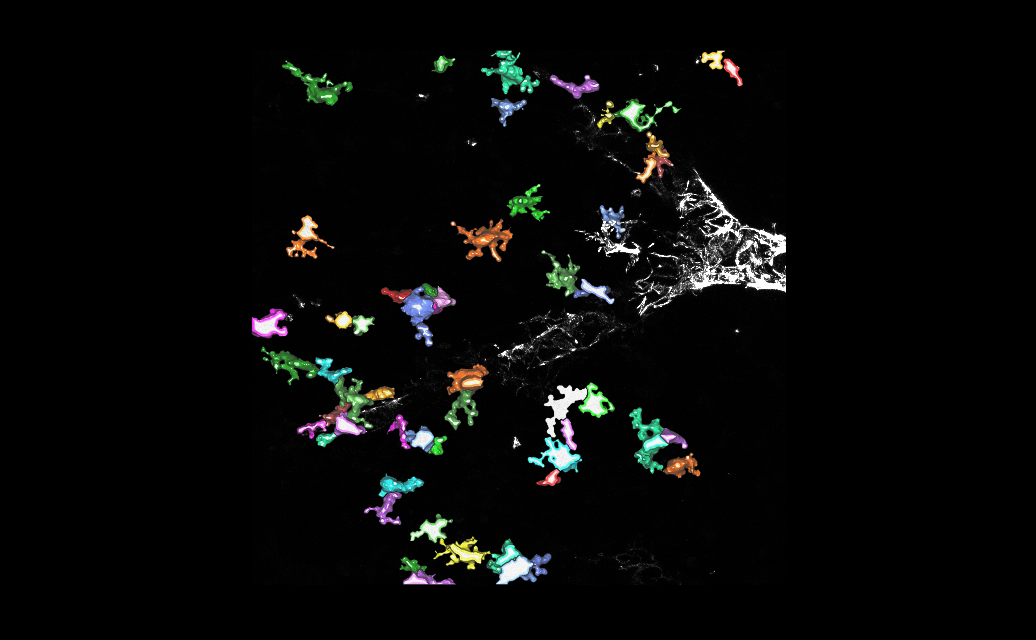

Supplement: Supplementary file 15 — Source Data Fig. 7 [file 44319_2023_54_MOESM15_ESM.zip › 7E/1. Day 0/3_Normoxia_4mg TAM_20x_NG2_HIF2a_SMA_4_Airyscan Processing_z15_t000_SMA2.bmp]

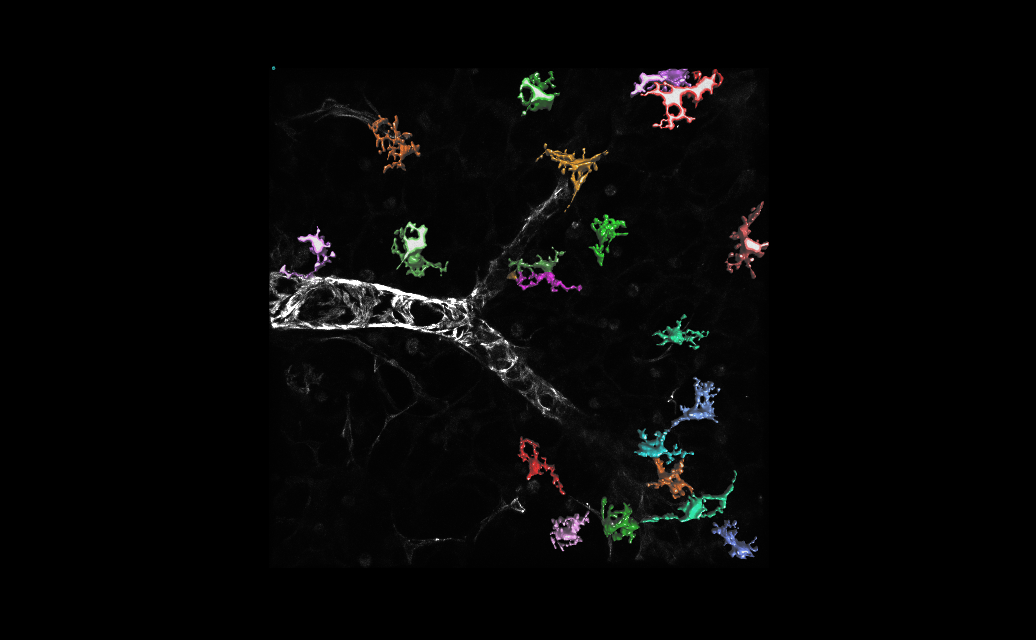

Supplement: Supplementary file 15 — Source Data Fig. 7 [file 44319_2023_54_MOESM15_ESM.zip › 7E/2. Day 2/Day 2_1_tdT_SMA_DAPI_1_Airyscan Processing_z00_t000_SMA2.bmp]

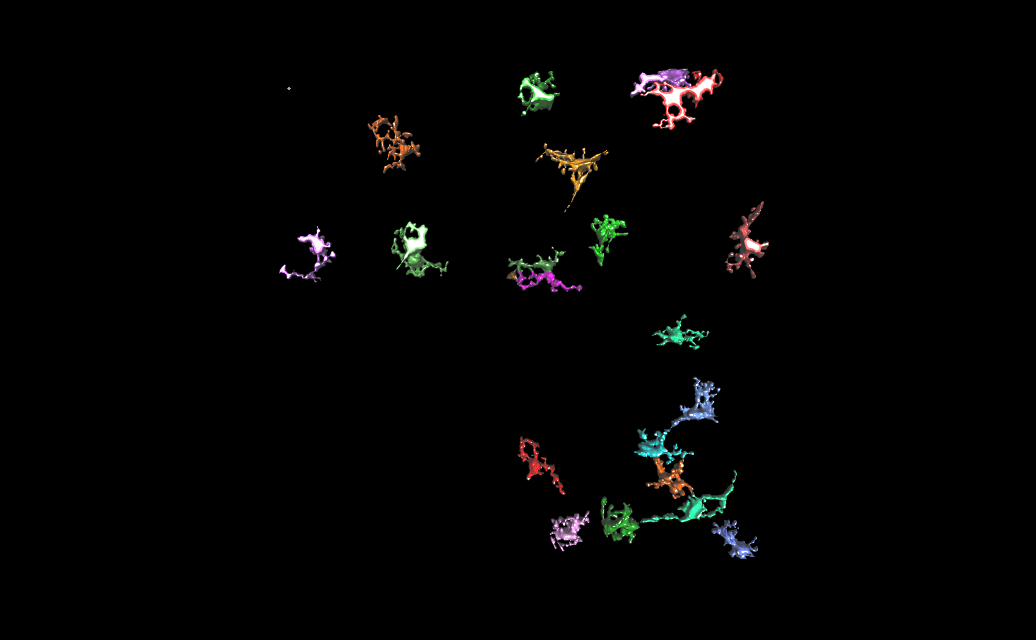

Supplement: Supplementary file 15 — Source Data Fig. 7 [file 44319_2023_54_MOESM15_ESM.zip › 7E/2. Day 2/Day 2_1_tdT_SMA_DAPI_1_Airyscan Processing_z02_t000.bmp]

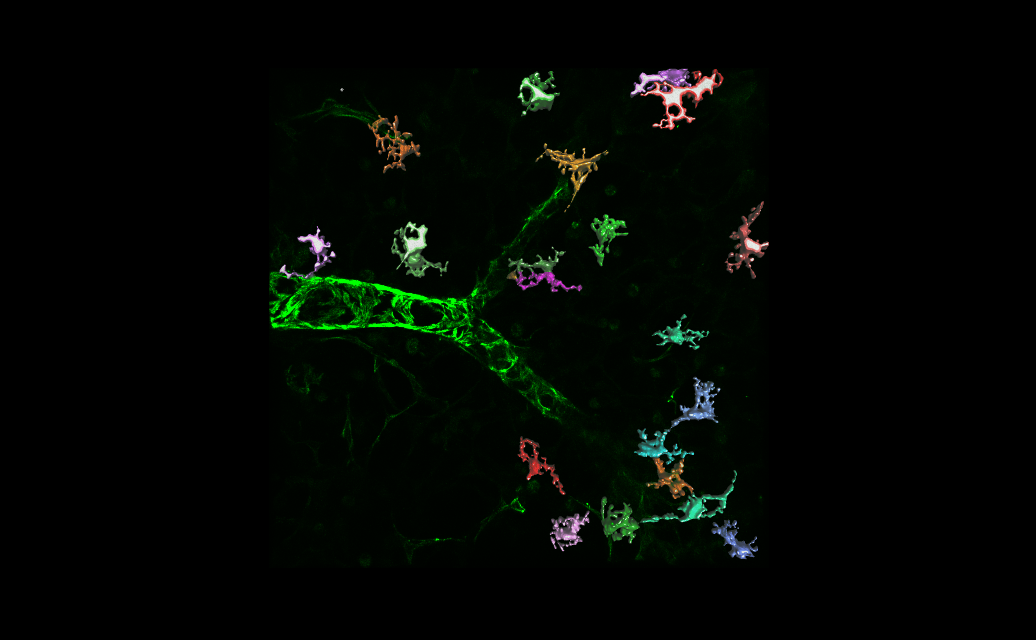

Supplement: Supplementary file 15 — Source Data Fig. 7 [file 44319_2023_54_MOESM15_ESM.zip › 7E/2. Day 2/Day 2_1_tdT_SMA_DAPI_1_Airyscan Processing_z02_t000_SMA.bmp]

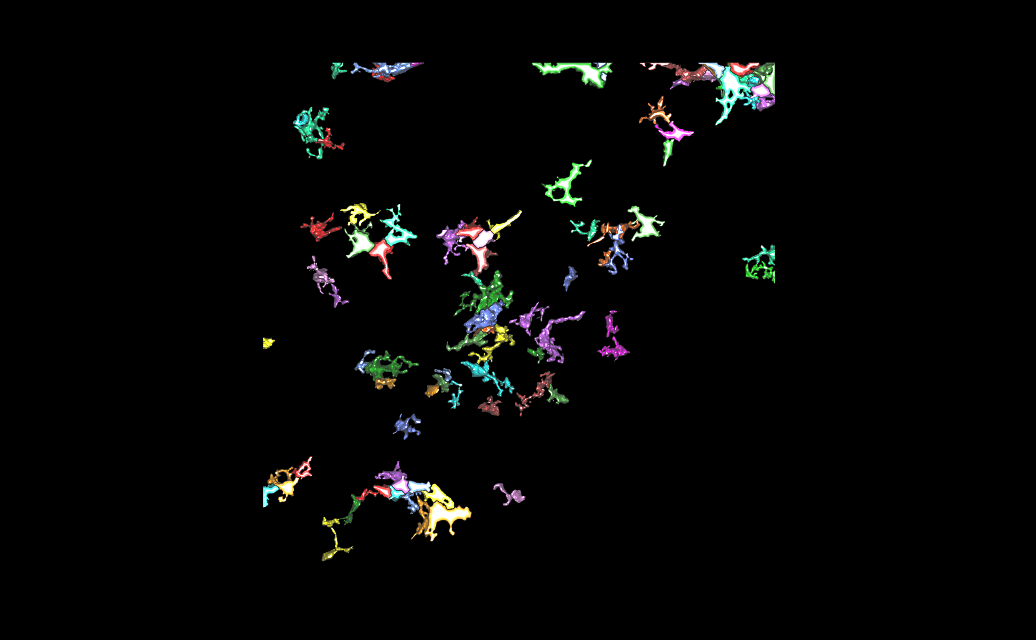

Supplement: Supplementary file 15 — Source Data Fig. 7 [file 44319_2023_54_MOESM15_ESM.zip › 7E/3. Day 4/Day 4_1_tdT_SMA_DAPI_2_Airyscan Processing_z16_t000.bmp]

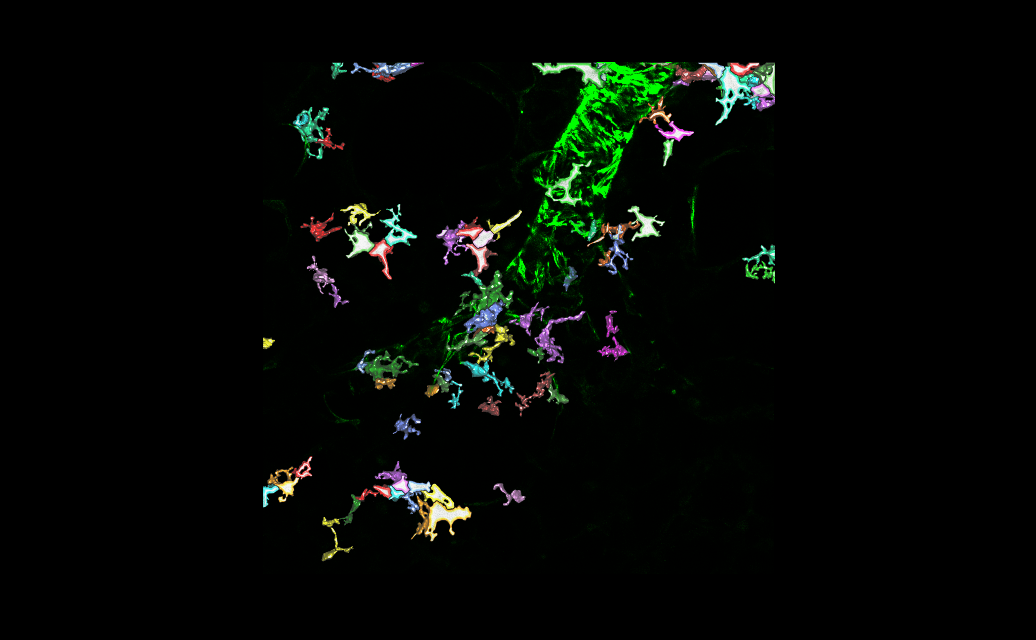

Supplement: Supplementary file 15 — Source Data Fig. 7 [file 44319_2023_54_MOESM15_ESM.zip › 7E/3. Day 4/Day 4_1_tdT_SMA_DAPI_2_Airyscan Processing_z16_t000_SMA.bmp]

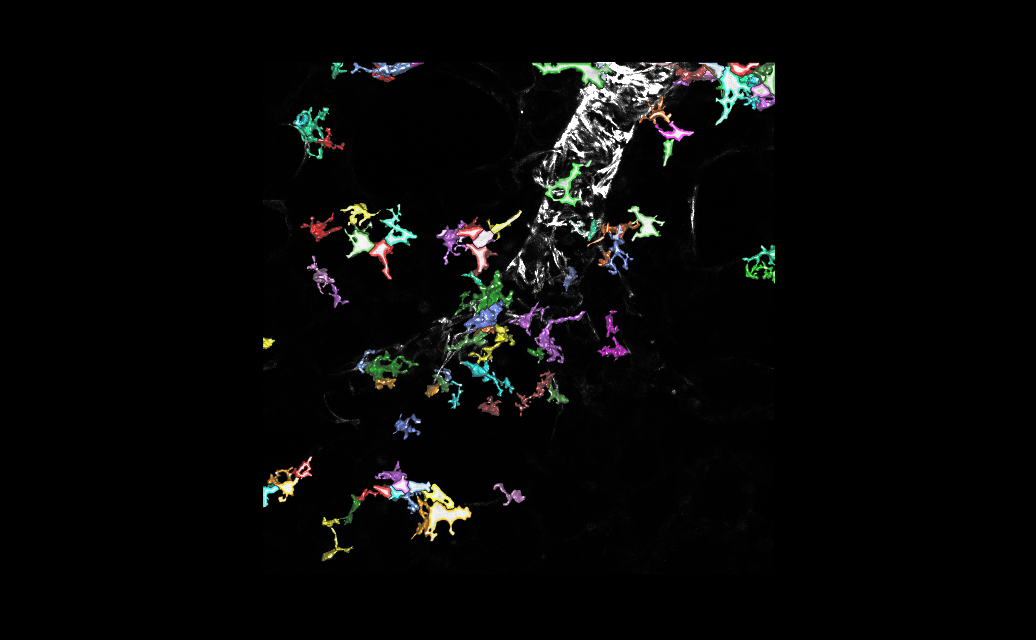

Supplement: Supplementary file 15 — Source Data Fig. 7 [file 44319_2023_54_MOESM15_ESM.zip › 7E/3. Day 4/Day 4_1_tdT_SMA_DAPI_2_Airyscan Processing_z16_t000_SMA2.bmp]

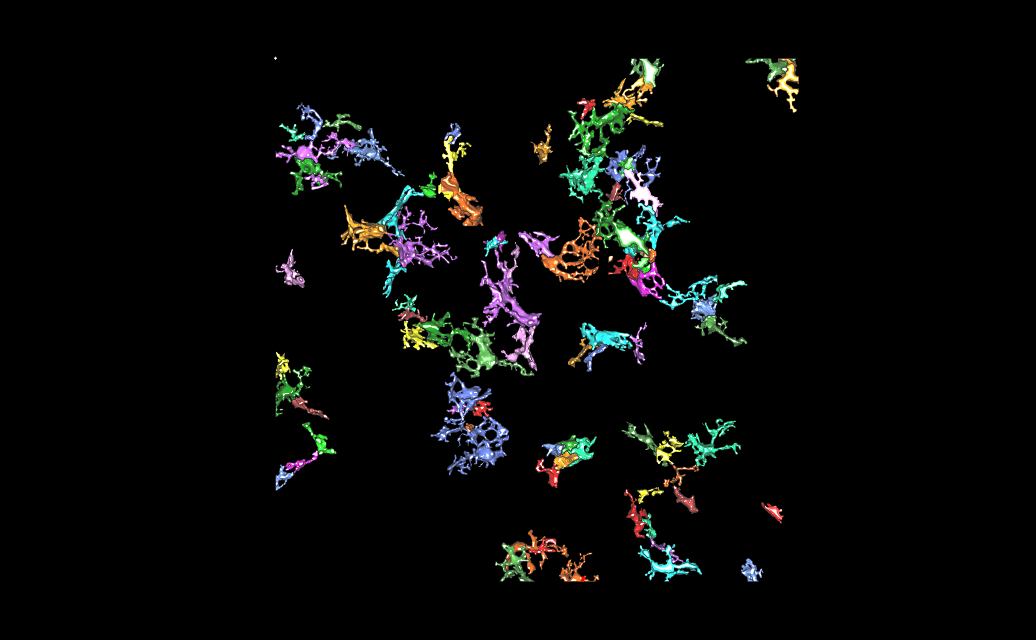

Supplement: Supplementary file 15 — Source Data Fig. 7 [file 44319_2023_54_MOESM15_ESM.zip › 7E/4. Day 7/1_1wk hx_4mg TAM_20x_NG2_HIF2a_SMA_3_Airyscan Processing_z04_t000.bmp]

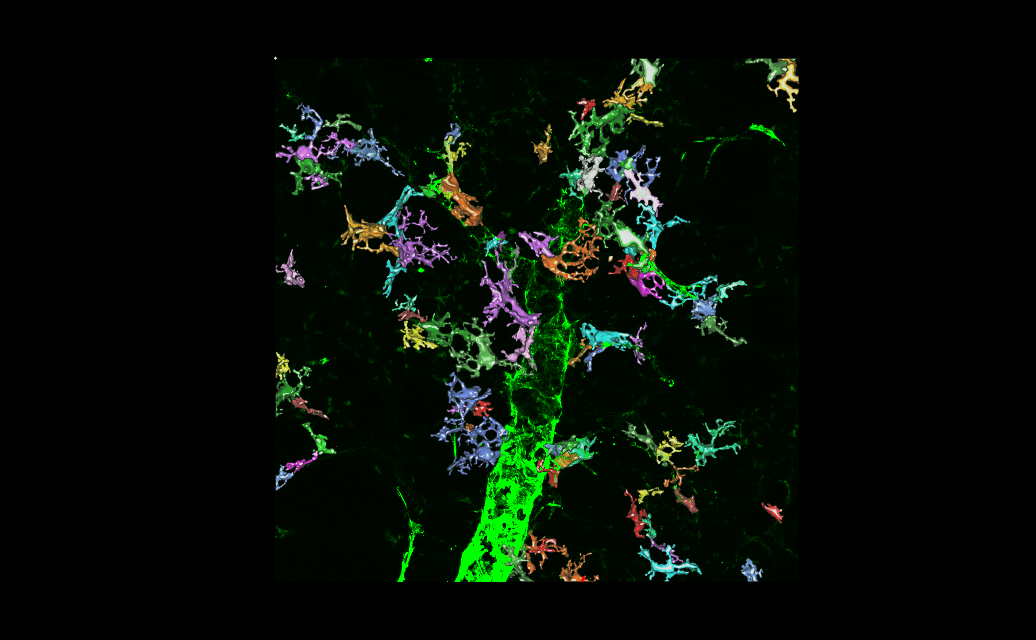

Supplement: Supplementary file 15 — Source Data Fig. 7 [file 44319_2023_54_MOESM15_ESM.zip › 7E/4. Day 7/1_1wk hx_4mg TAM_20x_NG2_HIF2a_SMA_3_Airyscan Processing_z27_t000_SMA.bmp]

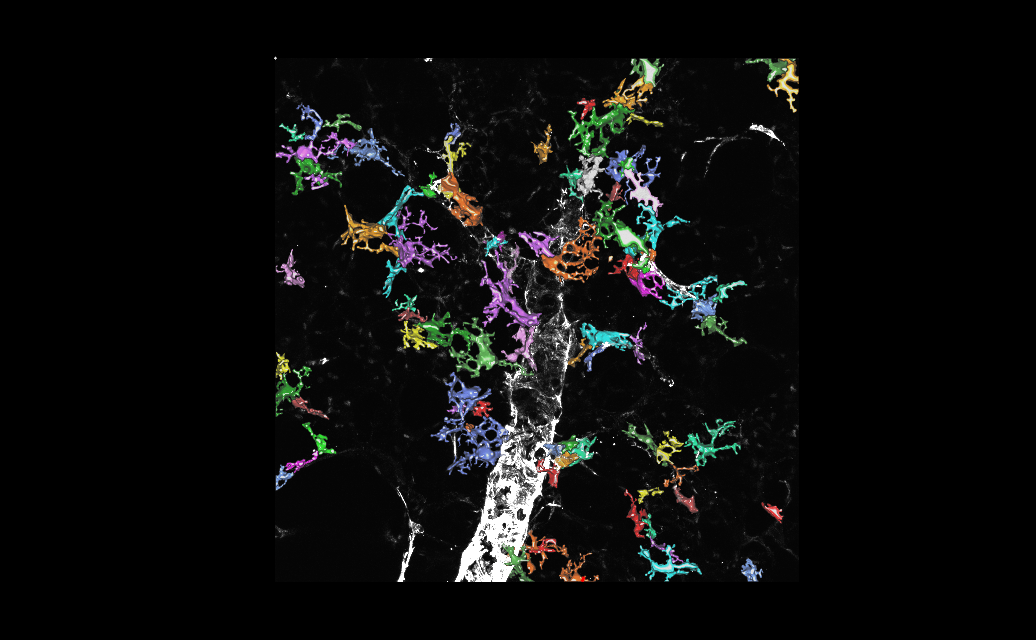

Supplement: Supplementary file 15 — Source Data Fig. 7 [file 44319_2023_54_MOESM15_ESM.zip › 7E/4. Day 7/1_1wk hx_4mg TAM_20x_NG2_HIF2a_SMA_3_Airyscan Processing_z27_t000_SMA2.bmp]

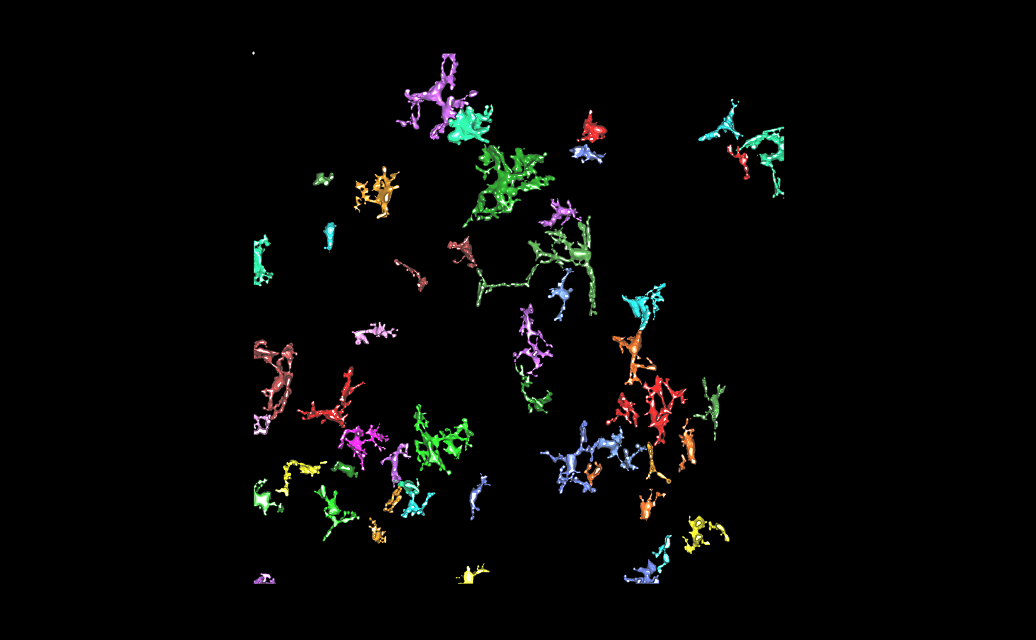

Supplement: Supplementary file 15 — Source Data Fig. 7 [file 44319_2023_54_MOESM15_ESM.zip › 7E/5. Day 14/5_Day 14_1_tdT_SMA_DAPI_1_Airyscan Processing_z18_t000.bmp]

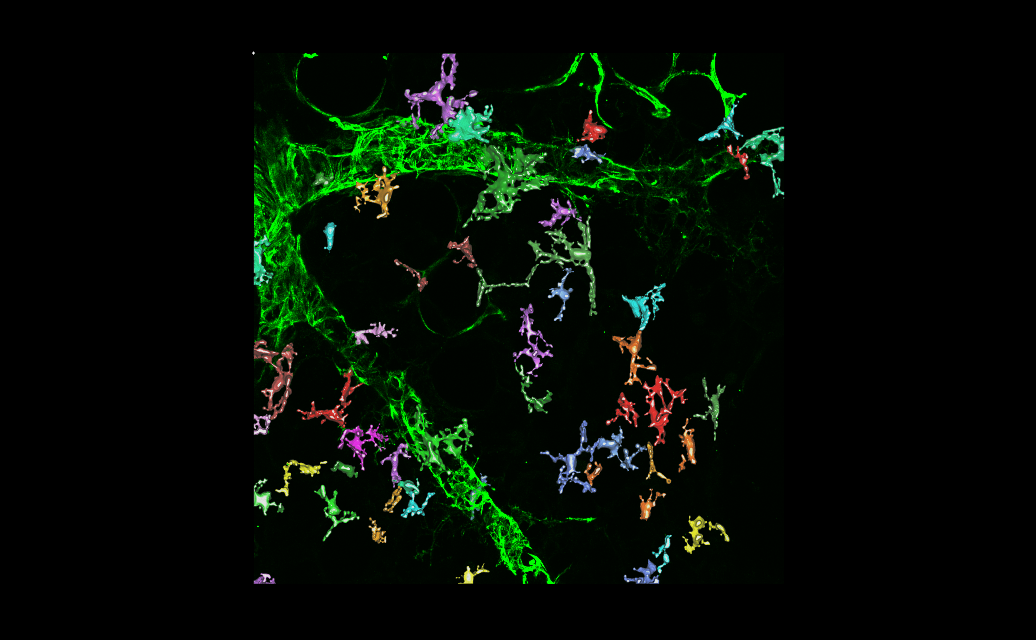

Supplement: Supplementary file 15 — Source Data Fig. 7 [file 44319_2023_54_MOESM15_ESM.zip › 7E/5. Day 14/5_Day 14_1_tdT_SMA_DAPI_1_Airyscan Processing_z18_t000_SMA.bmp]

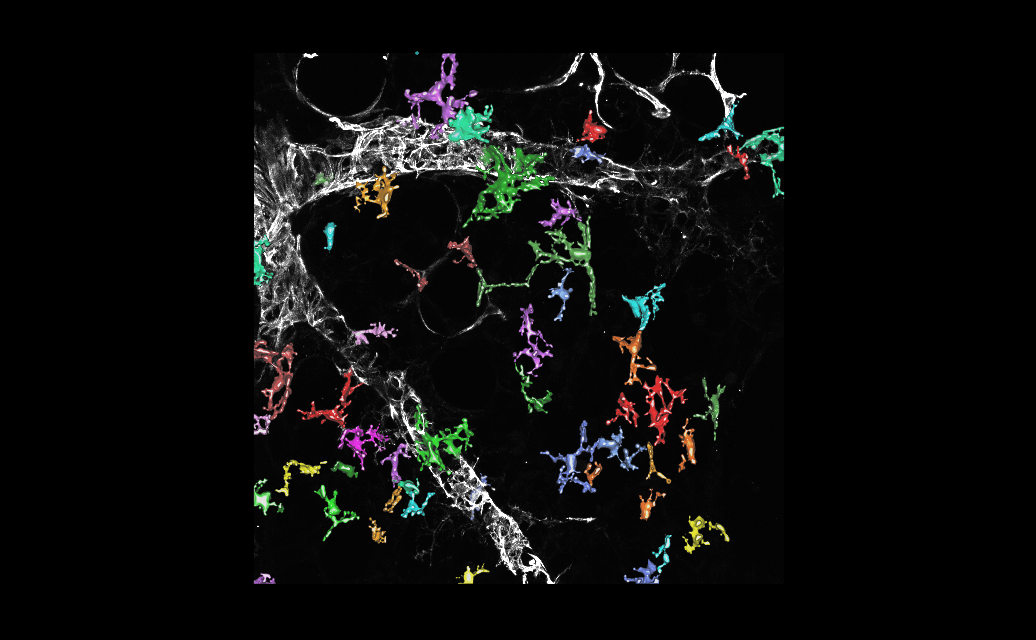

Supplement: Supplementary file 15 — Source Data Fig. 7 [file 44319_2023_54_MOESM15_ESM.zip › 7E/5. Day 14/5_Day 14_1_tdT_SMA_DAPI_1_Airyscan Processing_z18_t000_SMA2.bmp]

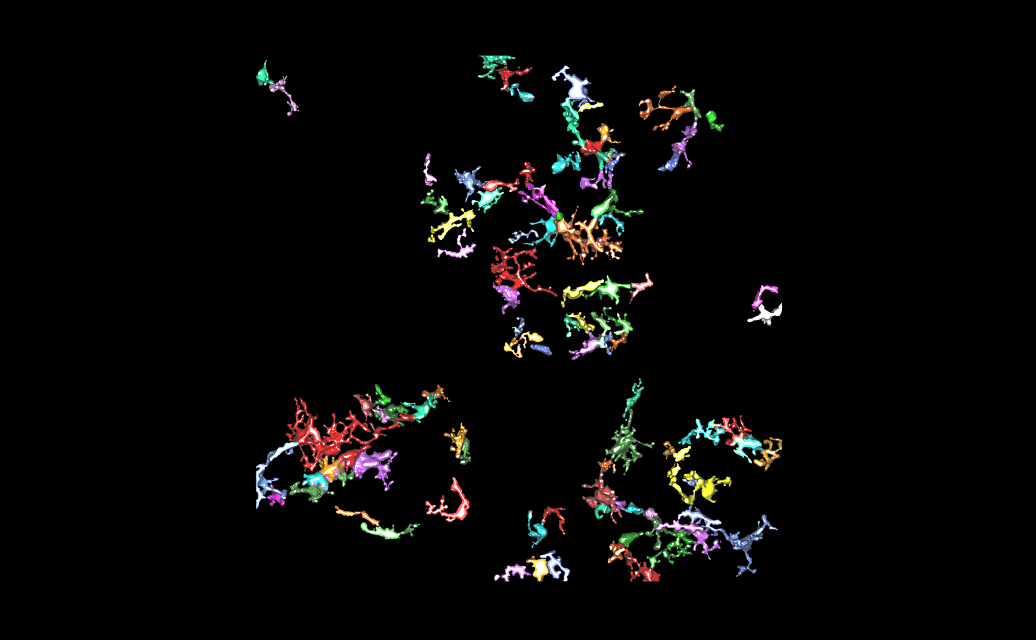

Supplement: Supplementary file 15 — Source Data Fig. 7 [file 44319_2023_54_MOESM15_ESM.zip › 7E/6. Day 21/2_3wk hx_4mg TAM_20x_NG2_HIF2a_SMA_1_Airyscan Processing_z13_t000.bmp]

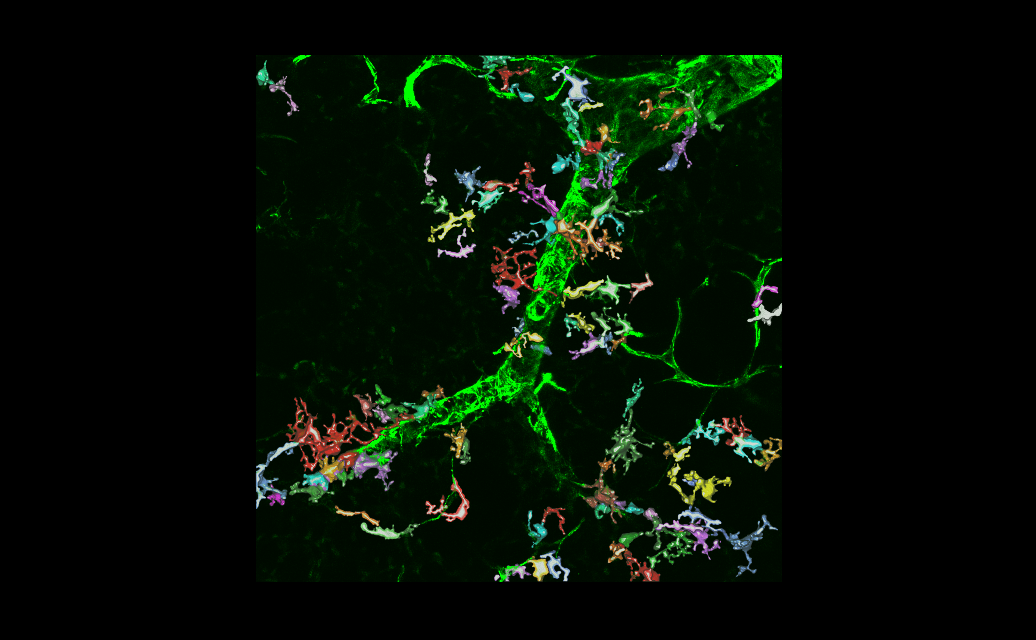

Supplement: Supplementary file 15 — Source Data Fig. 7 [file 44319_2023_54_MOESM15_ESM.zip › 7E/6. Day 21/2_3wk hx_4mg TAM_20x_NG2_HIF2a_SMA_1_Airyscan Processing_z13_t000_SMA.bmp]

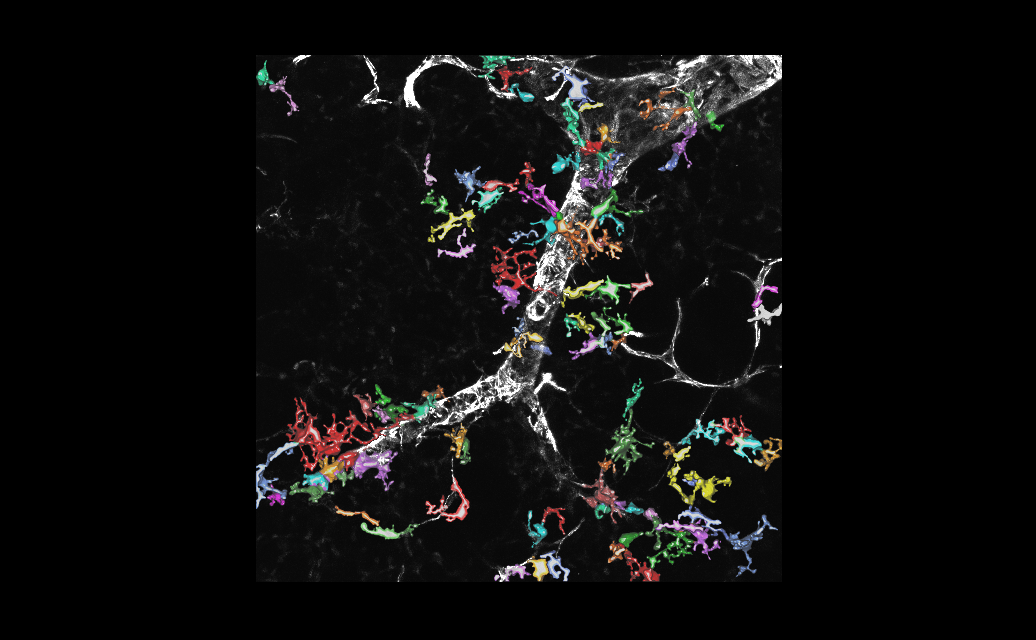

Supplement: Supplementary file 15 — Source Data Fig. 7 [file 44319_2023_54_MOESM15_ESM.zip › 7E/6. Day 21/2_3wk hx_4mg TAM_20x_NG2_HIF2a_SMA_1_Airyscan Processing_z13_t000_SMA2.bmp]

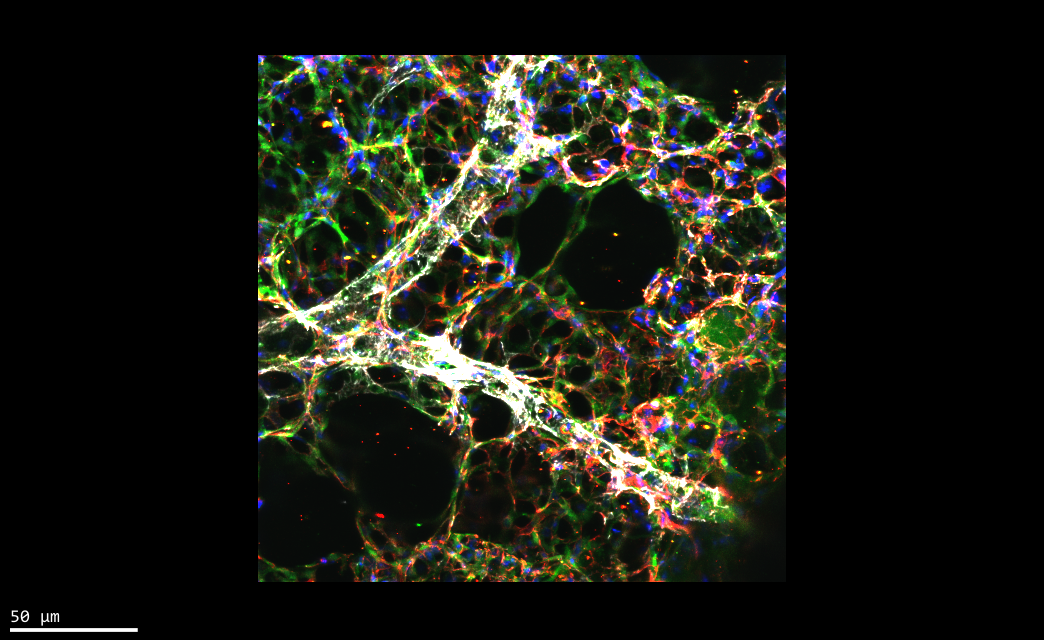

Supplement: Supplementary file 16 — Source Data Fig. 8 [file 44319_2023_54_MOESM16_ESM.zip › 8B/1_NG2-HIF2a_3wk Hx_Merge.bmp]

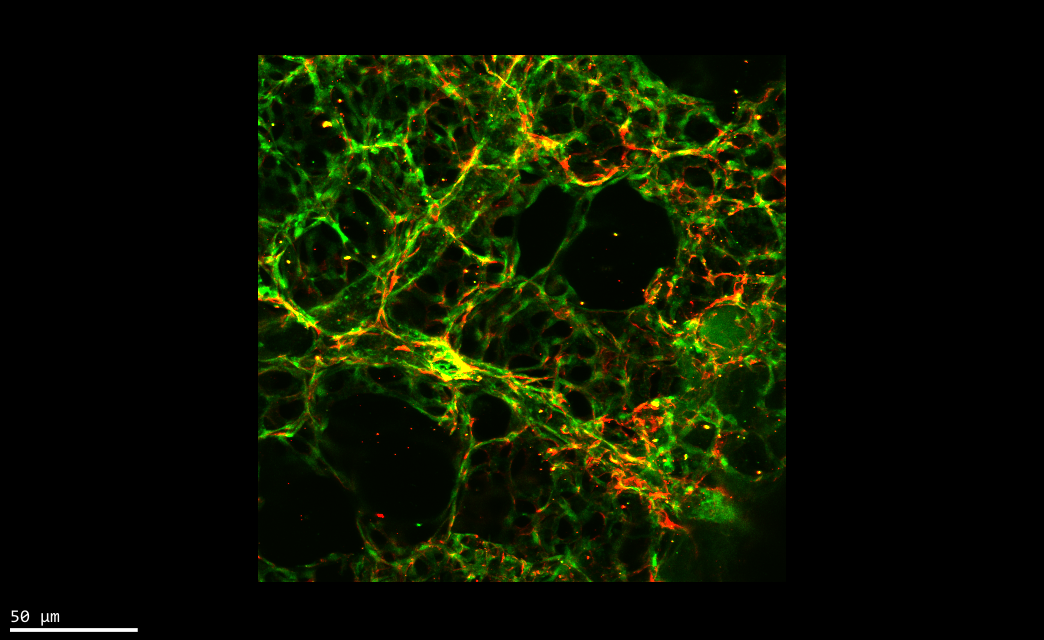

Supplement: Supplementary file 16 — Source Data Fig. 8 [file 44319_2023_54_MOESM16_ESM.zip › 8B/1_NG2-HIF2a_3wk Hx_Red-NG2_Green-CD31.bmp]

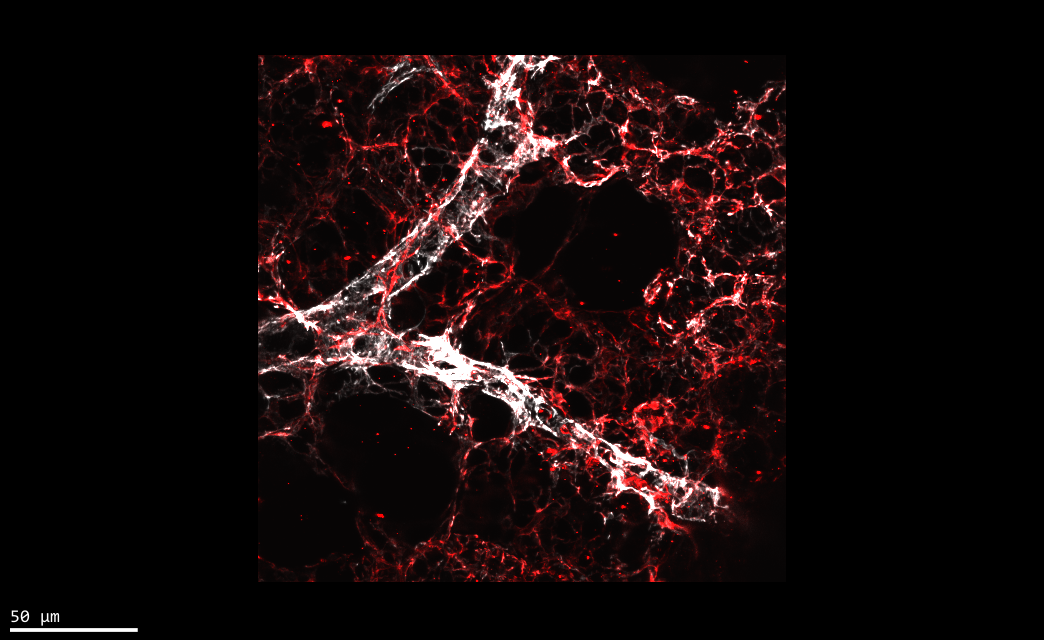

Supplement: Supplementary file 16 — Source Data Fig. 8 [file 44319_2023_54_MOESM16_ESM.zip › 8B/1_NG2-HIF2a_3wk Hx_Red-NG2_White-SMA.bmp]

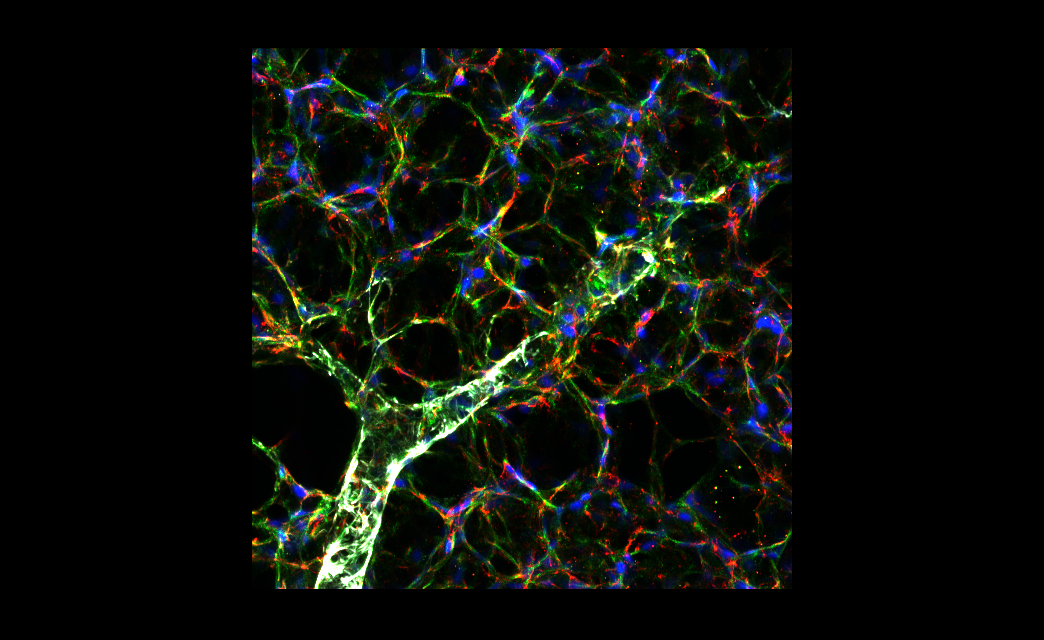

Supplement: Supplementary file 16 — Source Data Fig. 8 [file 44319_2023_54_MOESM16_ESM.zip › 8B/2_NG2-HIF2a_3wk Hx_AMD3100_Merge.bmp]

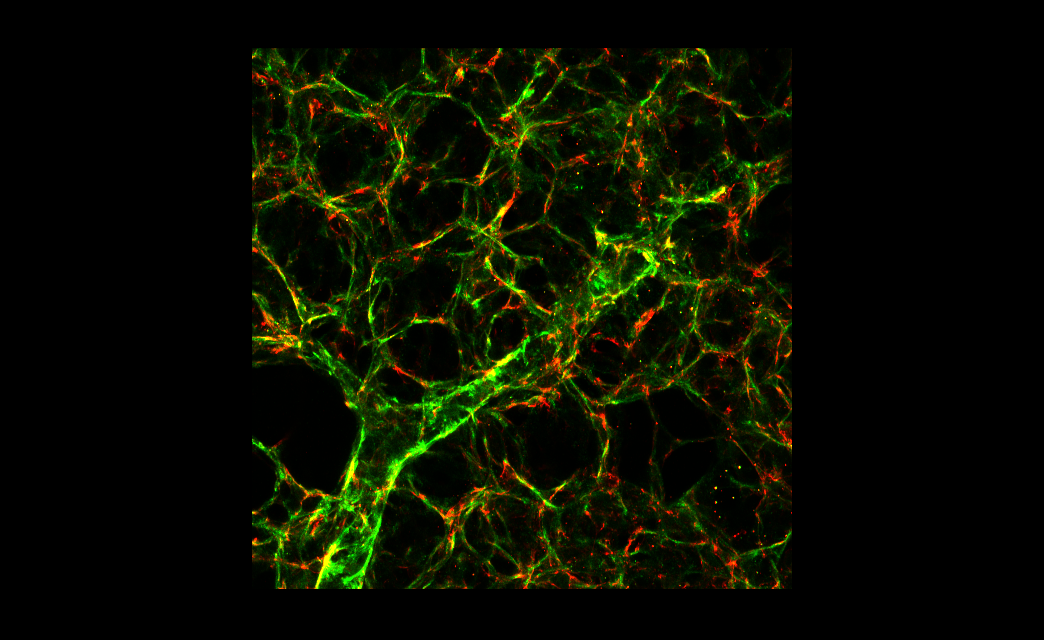

Supplement: Supplementary file 16 — Source Data Fig. 8 [file 44319_2023_54_MOESM16_ESM.zip › 8B/2_NG2-HIF2a_3wk Hx_AMD3100_Red-NG2_Green-CD31.bmp]

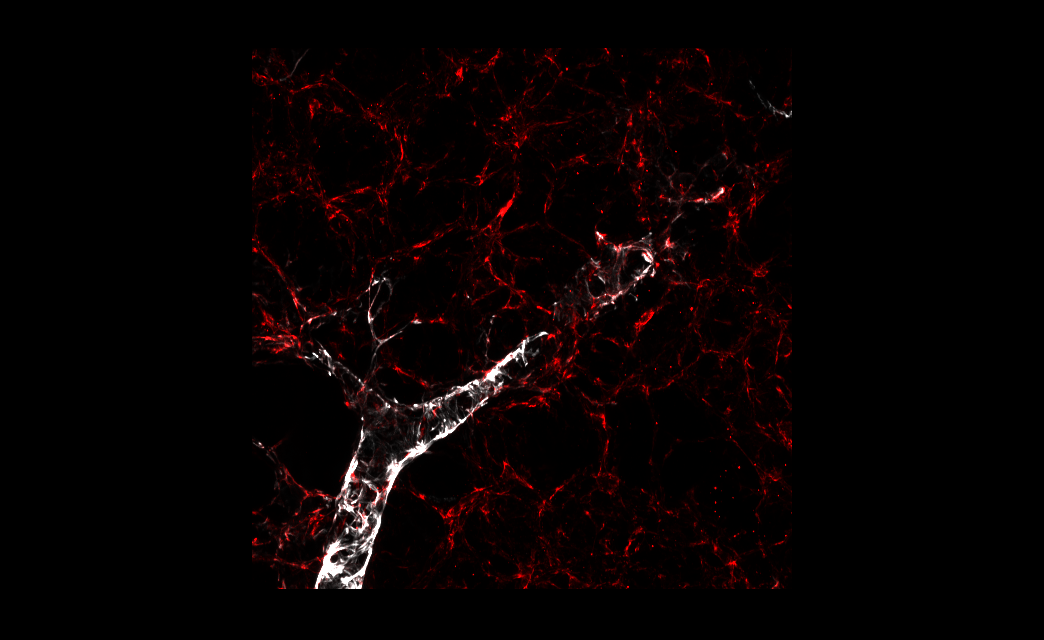

Supplement: Supplementary file 16 — Source Data Fig. 8 [file 44319_2023_54_MOESM16_ESM.zip › 8B/2_NG2-HIF2a_3wk Hx_AMD3100_Red-NG2_White-SMA.bmp]

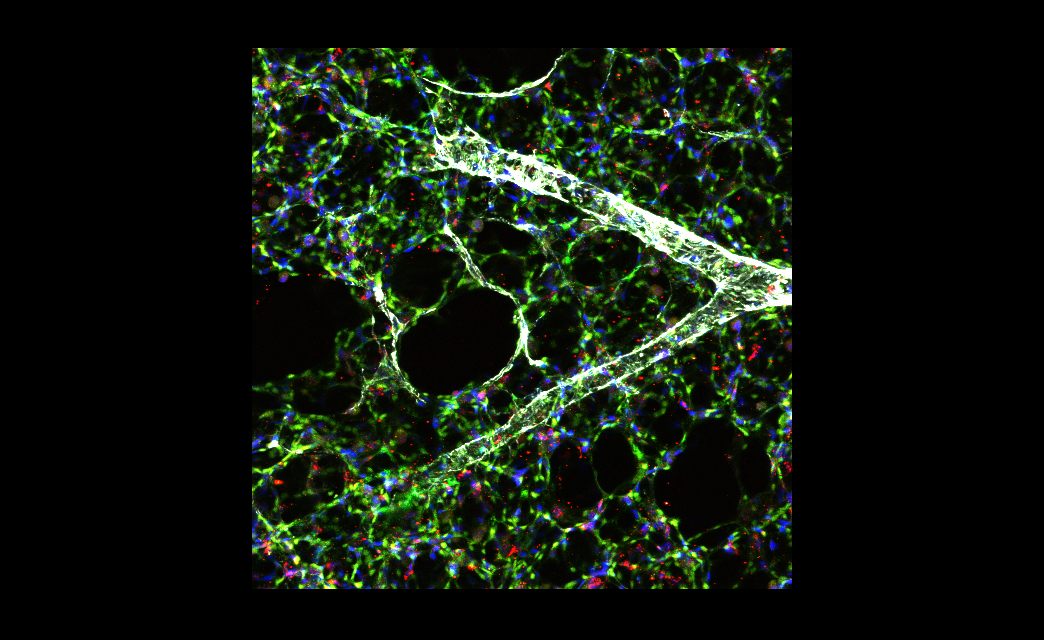

Supplement: Supplementary file 16 — Source Data Fig. 8 [file 44319_2023_54_MOESM16_ESM.zip › 8B/3_NG2-HIF2a_3wk Hx_Merge.bmp]

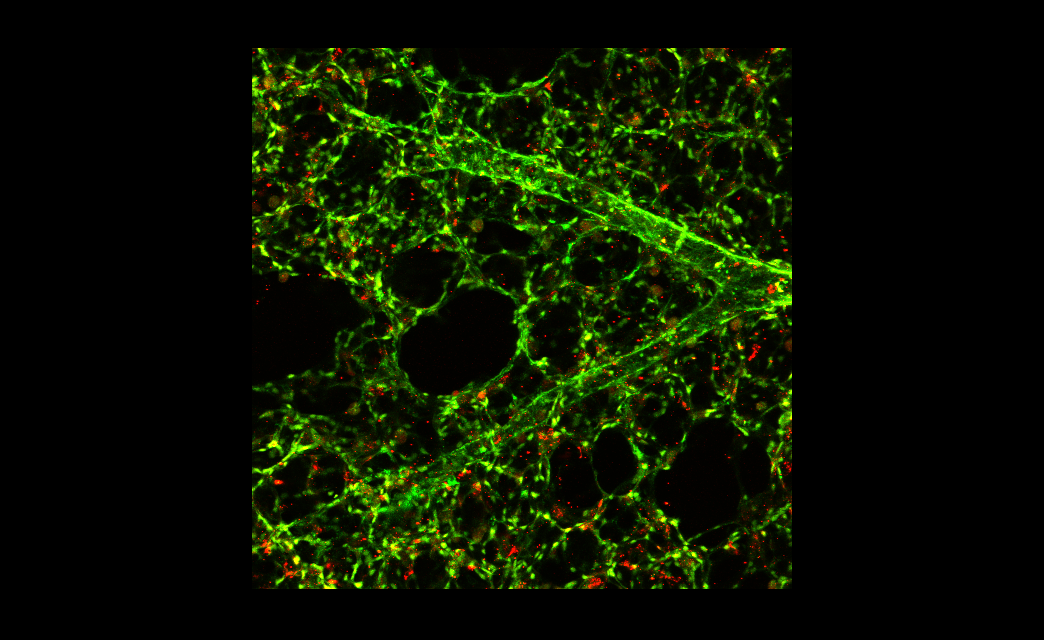

Supplement: Supplementary file 16 — Source Data Fig. 8 [file 44319_2023_54_MOESM16_ESM.zip › 8B/3_NG2-HIF2a_3wk Hx_Red-HIF2a_Green-PDGFRb.bmp]

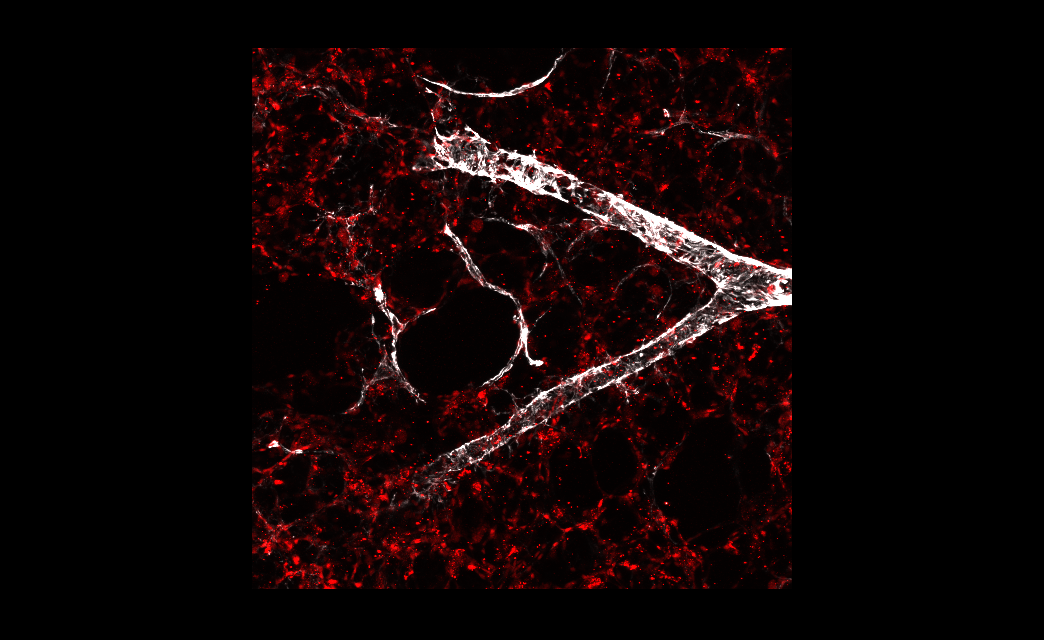

Supplement: Supplementary file 16 — Source Data Fig. 8 [file 44319_2023_54_MOESM16_ESM.zip › 8B/3_NG2-HIF2a_3wk Hx_Red-HIF2a_White-SMA.bmp]

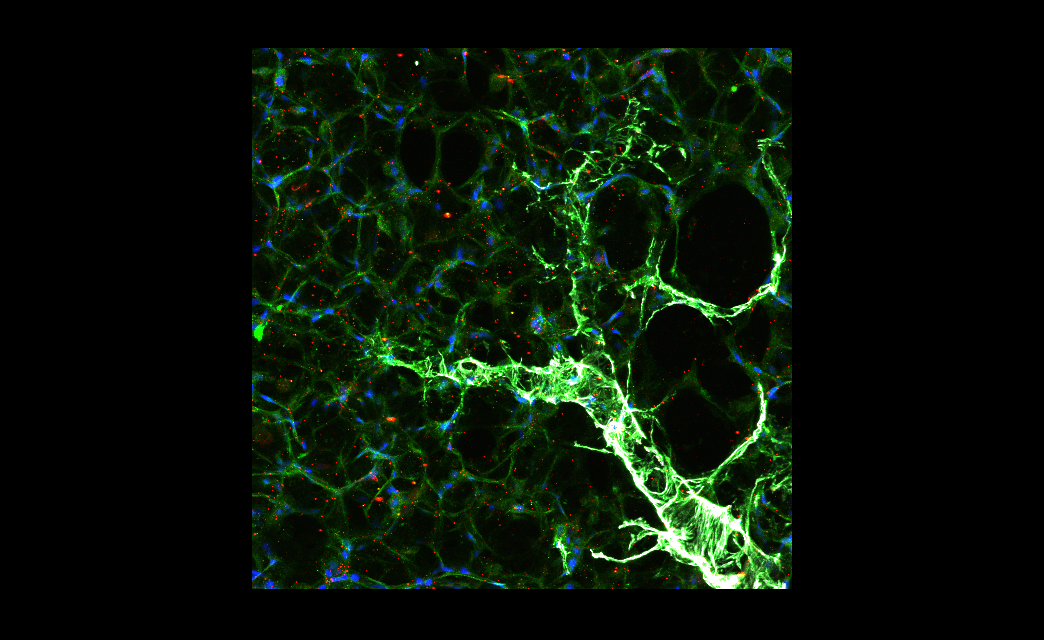

Supplement: Supplementary file 16 — Source Data Fig. 8 [file 44319_2023_54_MOESM16_ESM.zip › 8B/4_NG2-HIF2a_3wk Hx_AMD3100_Merge.bmp]

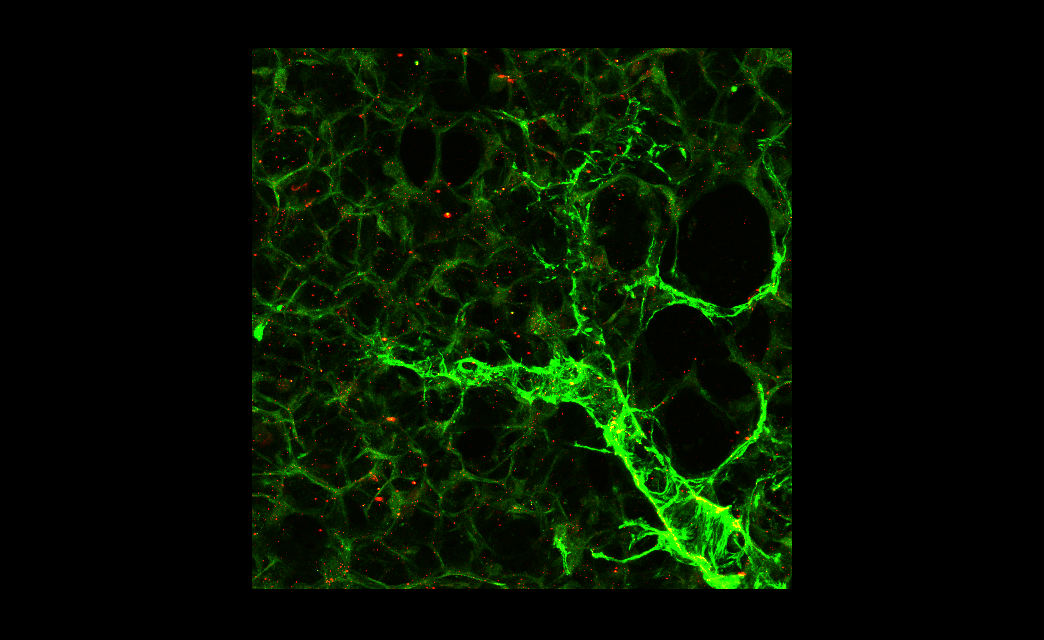

Supplement: Supplementary file 16 — Source Data Fig. 8 [file 44319_2023_54_MOESM16_ESM.zip › 8B/4_NG2-HIF2a_3wk Hx_AMD3100_Red-HIF2a_Green-PDGFRb.bmp]

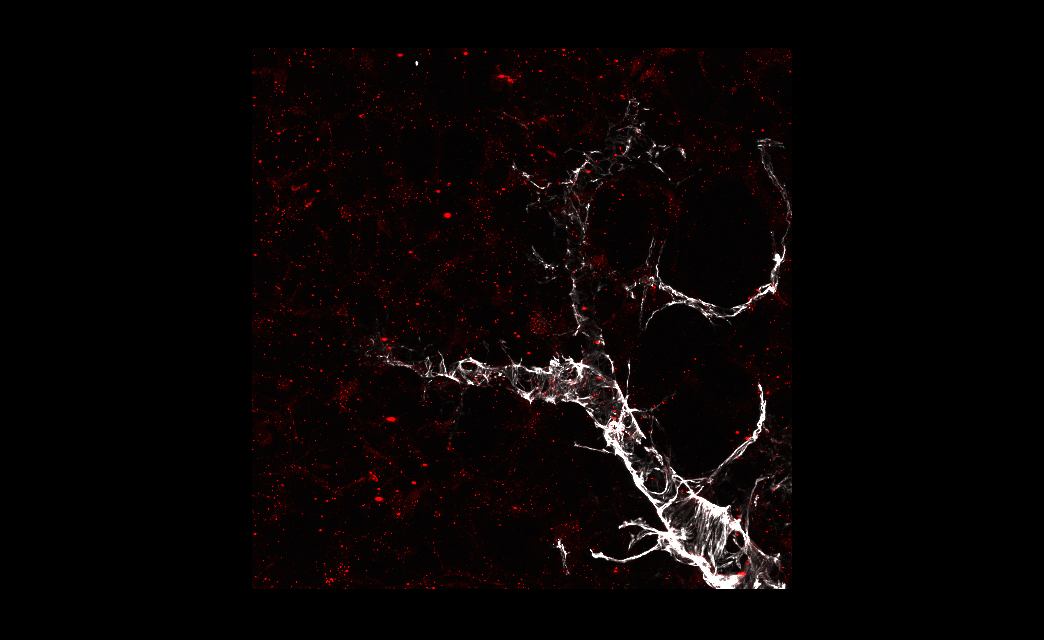

Supplement: Supplementary file 16 — Source Data Fig. 8 [file 44319_2023_54_MOESM16_ESM.zip › 8B/4_NG2-HIF2a_3wk Hx_AMD3100_Red-HIF2a_White-SMA.bmp]

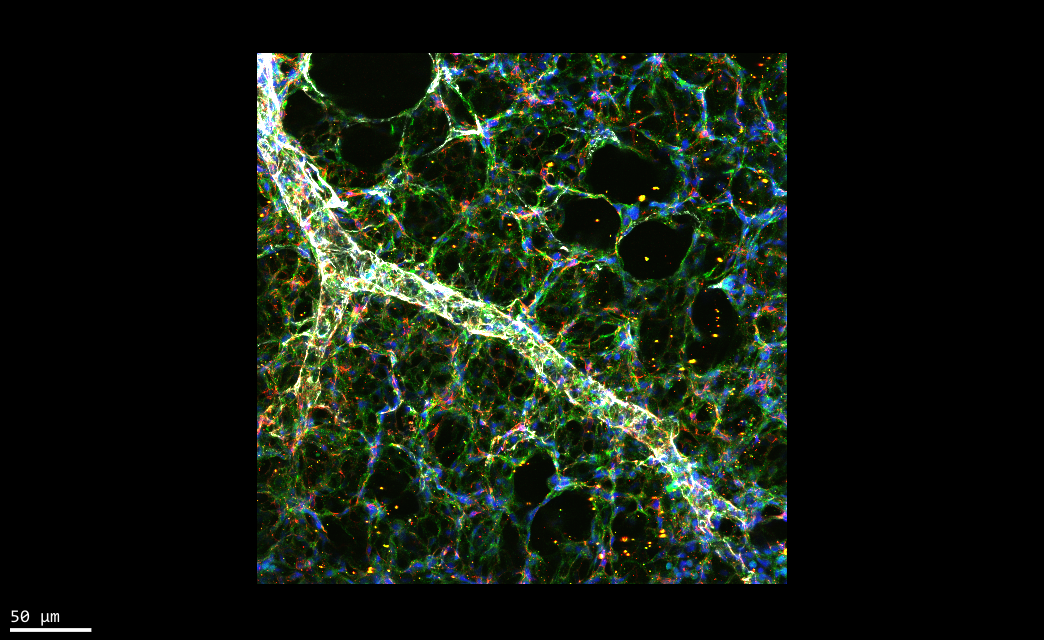

Supplement: Supplementary file 16 — Source Data Fig. 8 [file 44319_2023_54_MOESM16_ESM.zip › 8E/1_WT_3wk Hx_PBS_Merge.bmp]

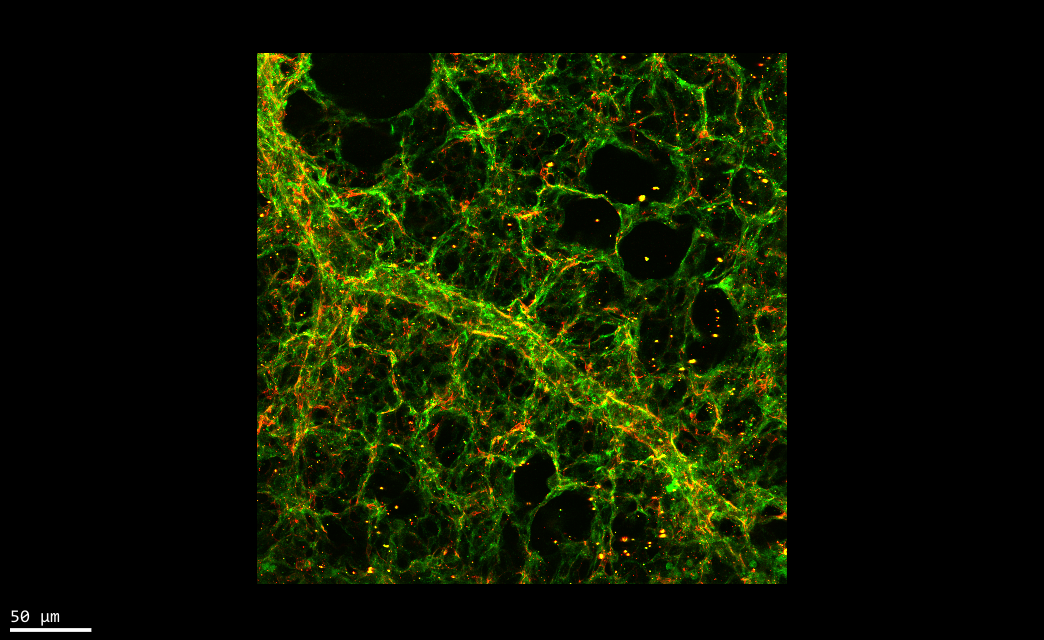

Supplement: Supplementary file 16 — Source Data Fig. 8 [file 44319_2023_54_MOESM16_ESM.zip › 8E/1_WT_3wk Hx_PBS_Red-NG2_Green-CD31.bmp]

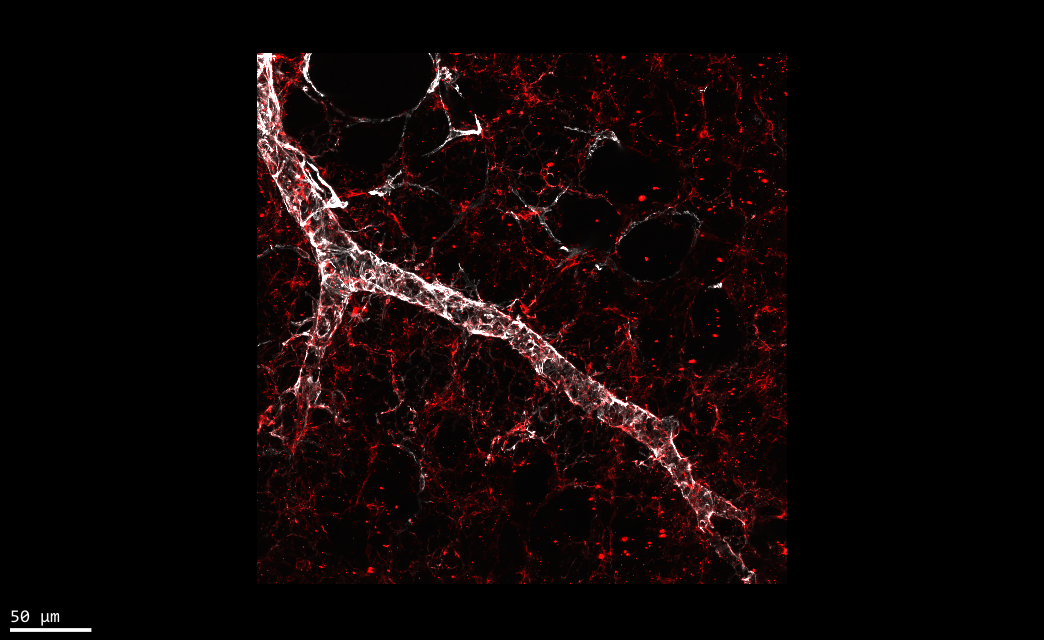

Supplement: Supplementary file 16 — Source Data Fig. 8 [file 44319_2023_54_MOESM16_ESM.zip › 8E/1_WT_3wk Hx_PBS_Red-NG2_White-SMA.bmp]

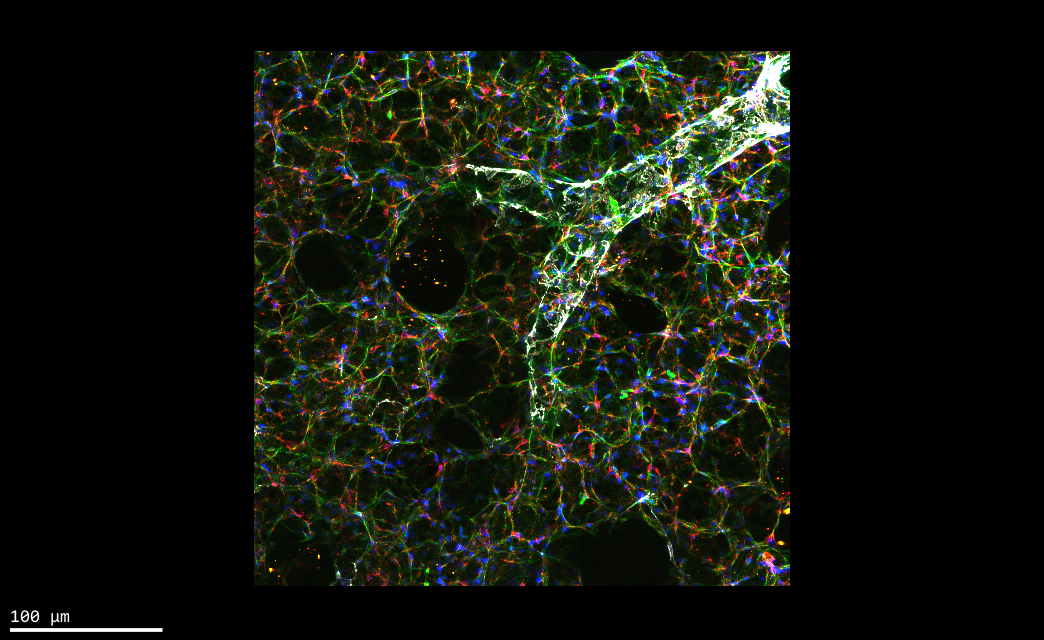

Supplement: Supplementary file 16 — Source Data Fig. 8 [file 44319_2023_54_MOESM16_ESM.zip › 8E/2_WT_3wk Hx_AMD3100_Merge.bmp]

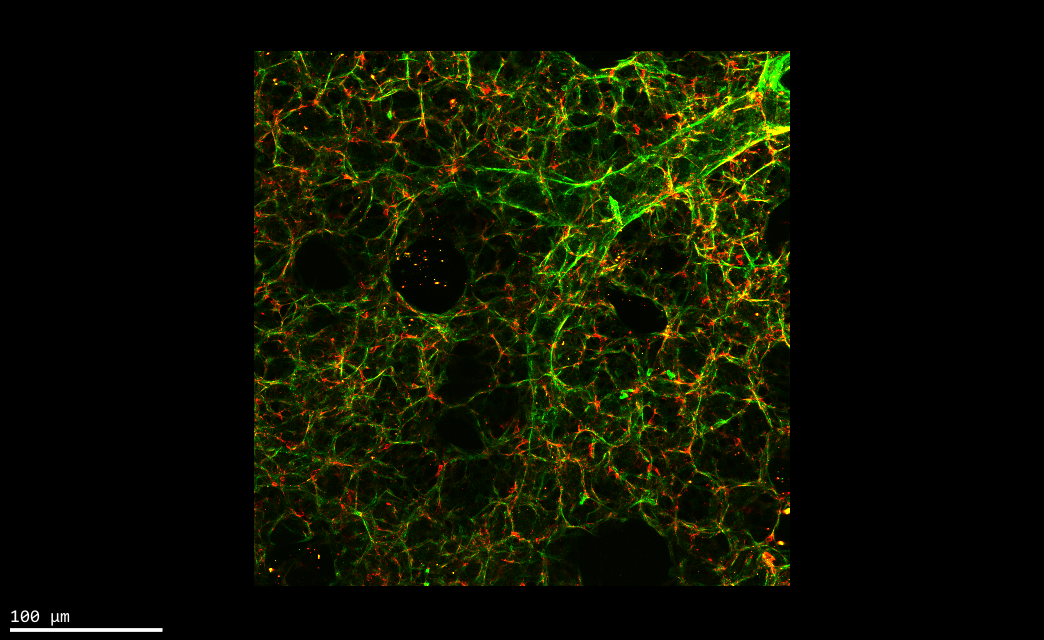

Supplement: Supplementary file 16 — Source Data Fig. 8 [file 44319_2023_54_MOESM16_ESM.zip › 8E/2_WT_3wk Hx_AMD3100_Red-NG2_Green-CD31.bmp]

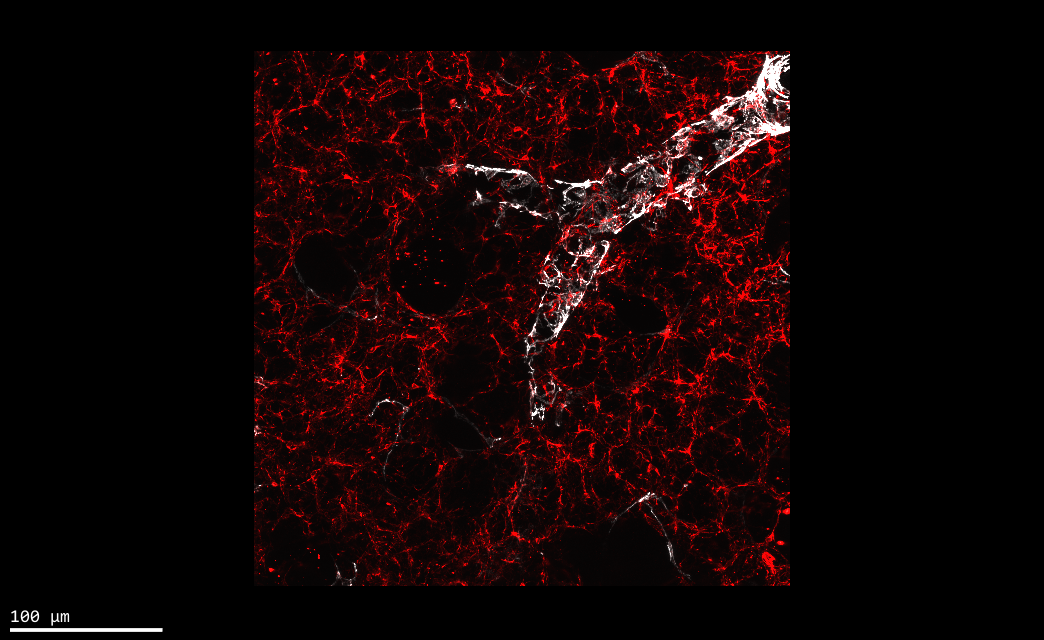

Supplement: Supplementary file 16 — Source Data Fig. 8 [file 44319_2023_54_MOESM16_ESM.zip › 8E/2_WT_3wk Hx_AMD3100_Red-NG2_White-SMA.bmp]

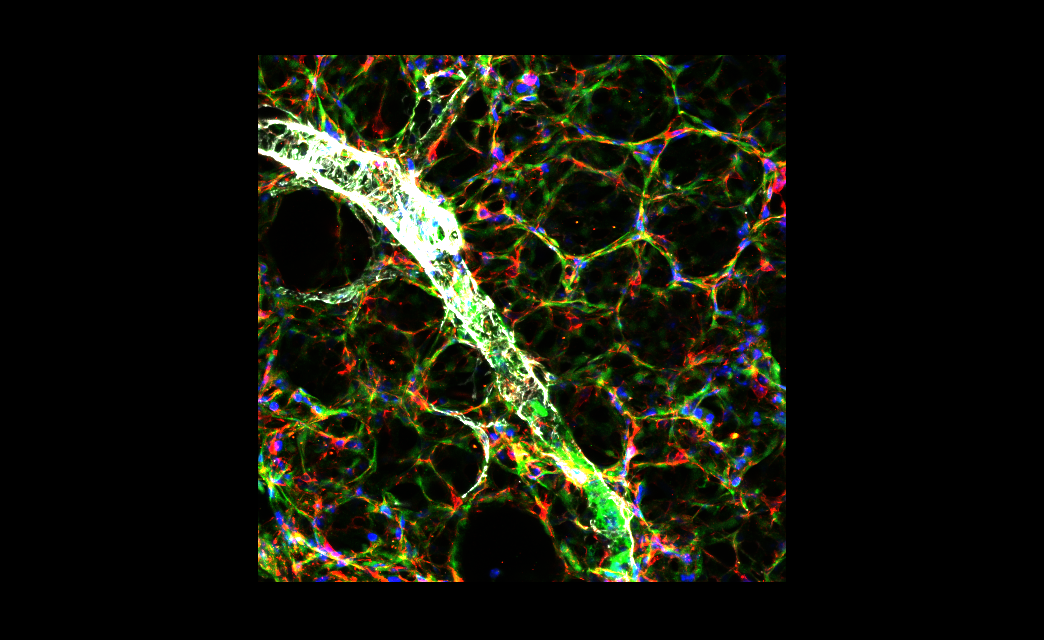

Supplement: Supplementary file 16 — Source Data Fig. 8 [file 44319_2023_54_MOESM16_ESM.zip › 8E/3_WT_6wk Hx_PBS_Merge.bmp]

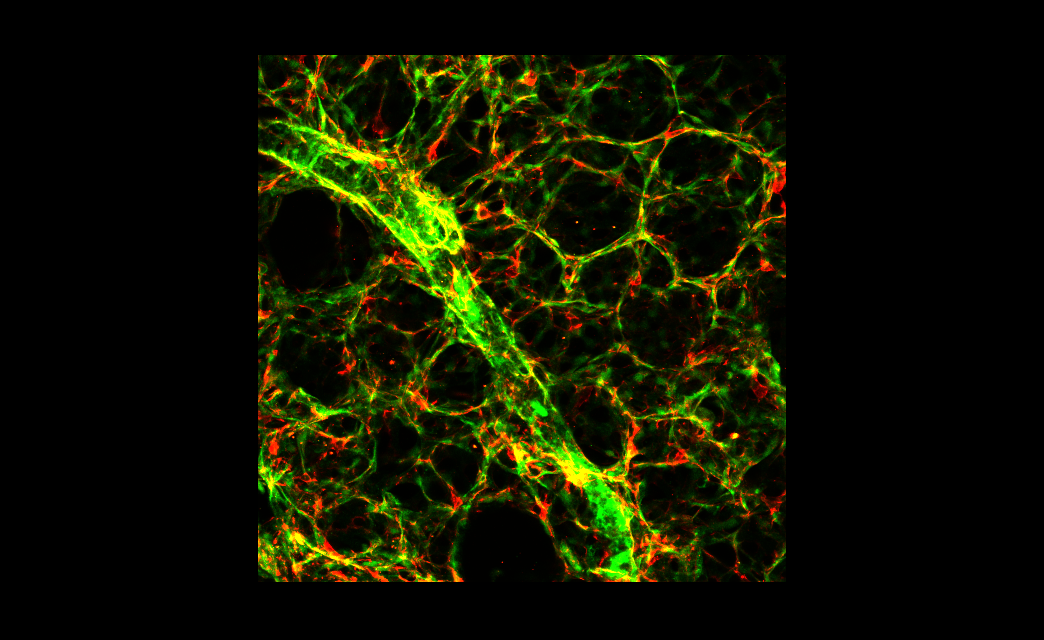

Supplement: Supplementary file 16 — Source Data Fig. 8 [file 44319_2023_54_MOESM16_ESM.zip › 8E/3_WT_6wk Hx_PBS_Red-NG2_Green-CD31.bmp]

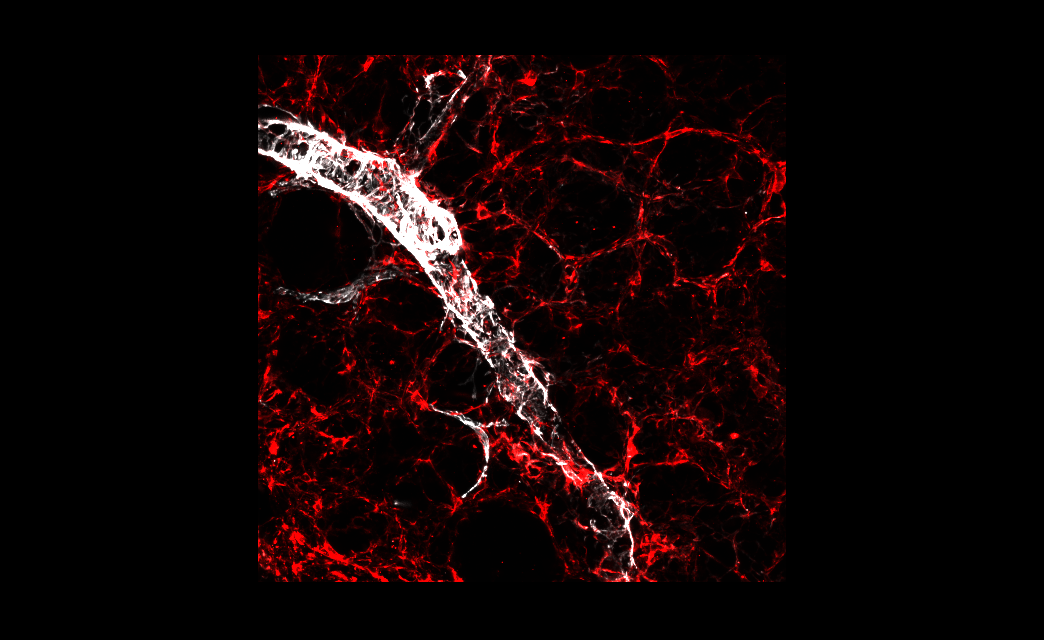

Supplement: Supplementary file 16 — Source Data Fig. 8 [file 44319_2023_54_MOESM16_ESM.zip › 8E/3_WT_6wk Hx_PBS_Red-NG2_White-SMA.bmp]

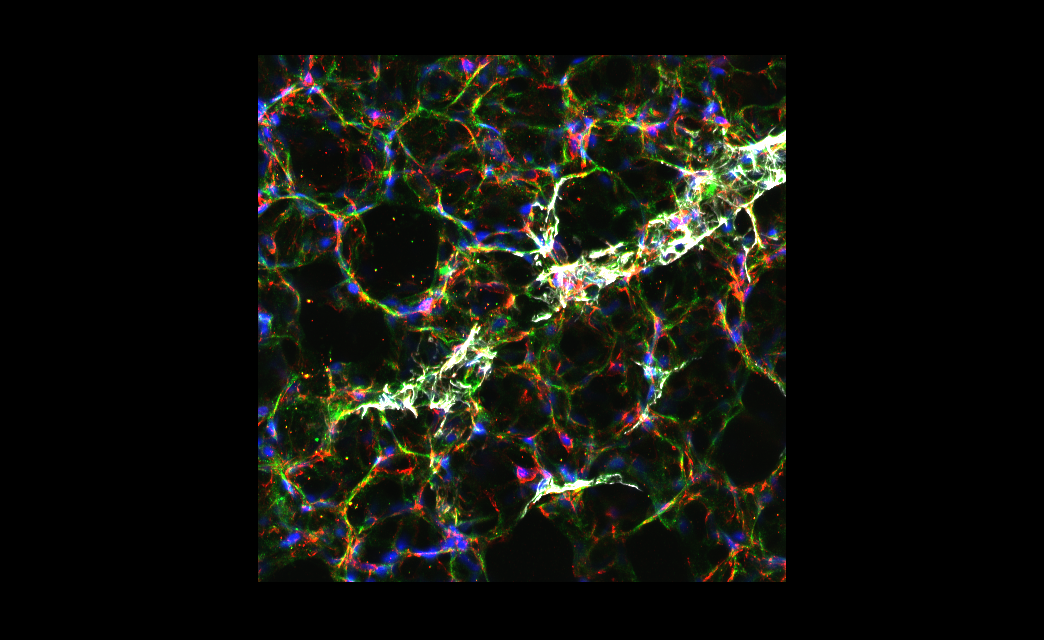

Supplement: Supplementary file 16 — Source Data Fig. 8 [file 44319_2023_54_MOESM16_ESM.zip › 8E/4_WT_6wk Hx_AMD3100_Merge.bmp]

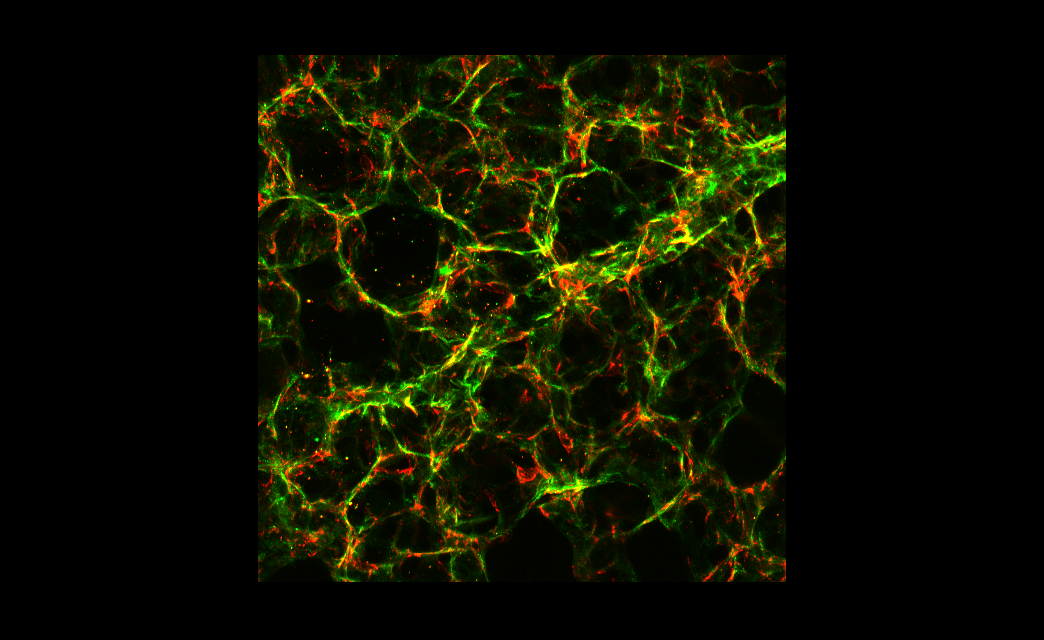

Supplement: Supplementary file 16 — Source Data Fig. 8 [file 44319_2023_54_MOESM16_ESM.zip › 8E/4_WT_6wk Hx_AMD3100_Red-NG2_Green-CD31.bmp]

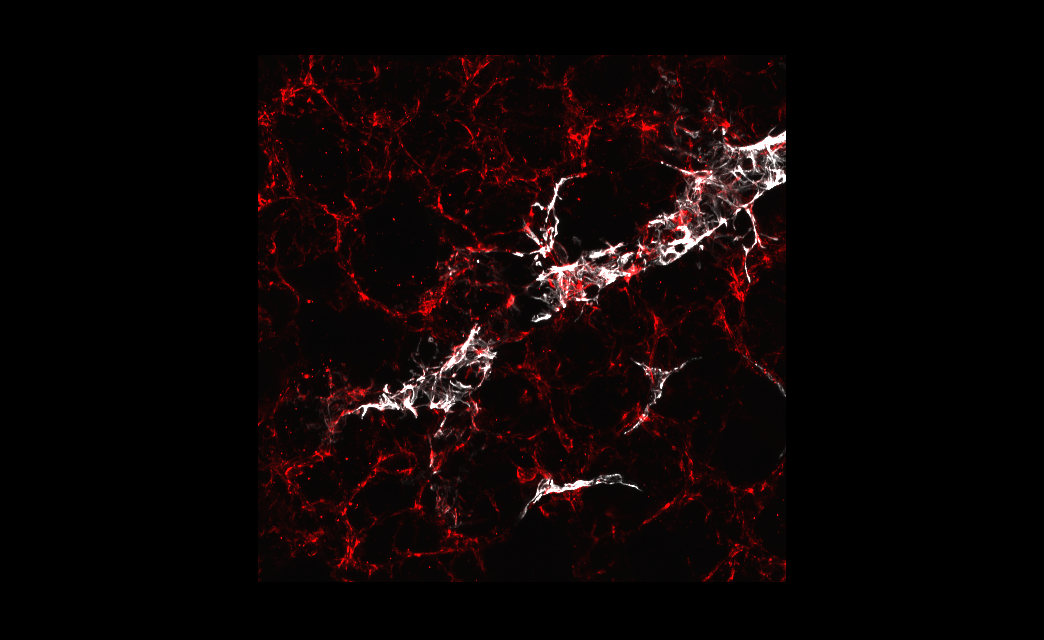

Supplement: Supplementary file 16 — Source Data Fig. 8 [file 44319_2023_54_MOESM16_ESM.zip › 8E/4_WT_6wk Hx_AMD3100_Red-NG2_White-SMA.bmp]
