# Supplementary material for: Pericytes contribute to pulmonary vascular remodeling via HIF2α signaling
Source: EMBO Rep. 2024 Jan 19;25(2):13. doi: 10.1038/s44319-023-00054-w (PMC10897382; doi:10.1038/s44319-023-00054-w)
Supplement: Supplementary file 18 — Expanded View Figures [file 44319_2023_54_MOESM18_ESM.pdf]

Expanded View Figures

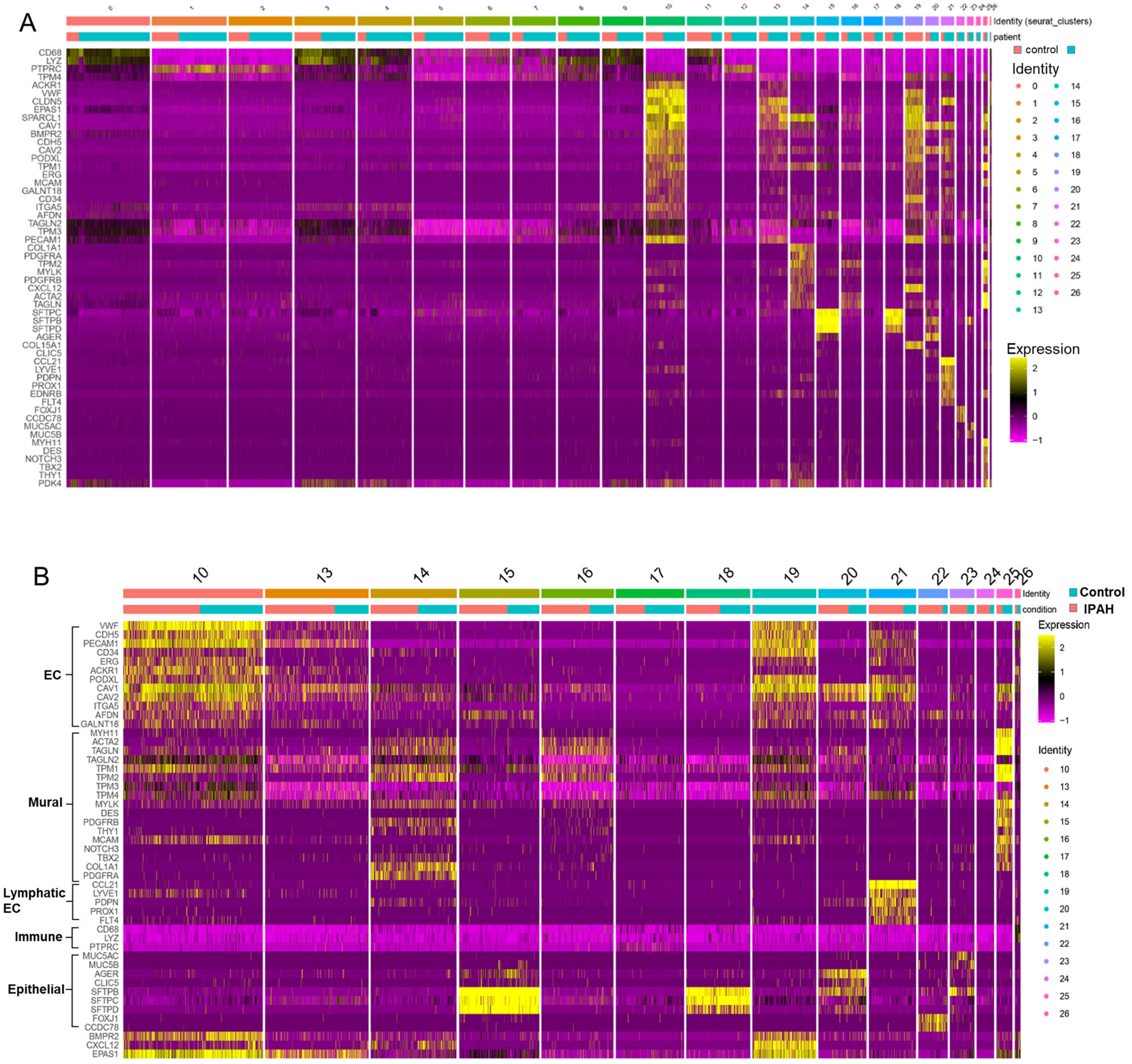

Figure EV1. 27 clusters are identified using human IPAH lung single-cell RNAseq.

(**A**) 27 distinct cell clusters were generated by Uniform Manifold Approximation and Projection (UMAP) plot from both human control and IPAH lungs by single-cell RNA-sequencing analysis. (**B**) The heatmap of expressed genes in clusters 10 and 13–26 was determined by the top gene expressions.

© The Author(s)

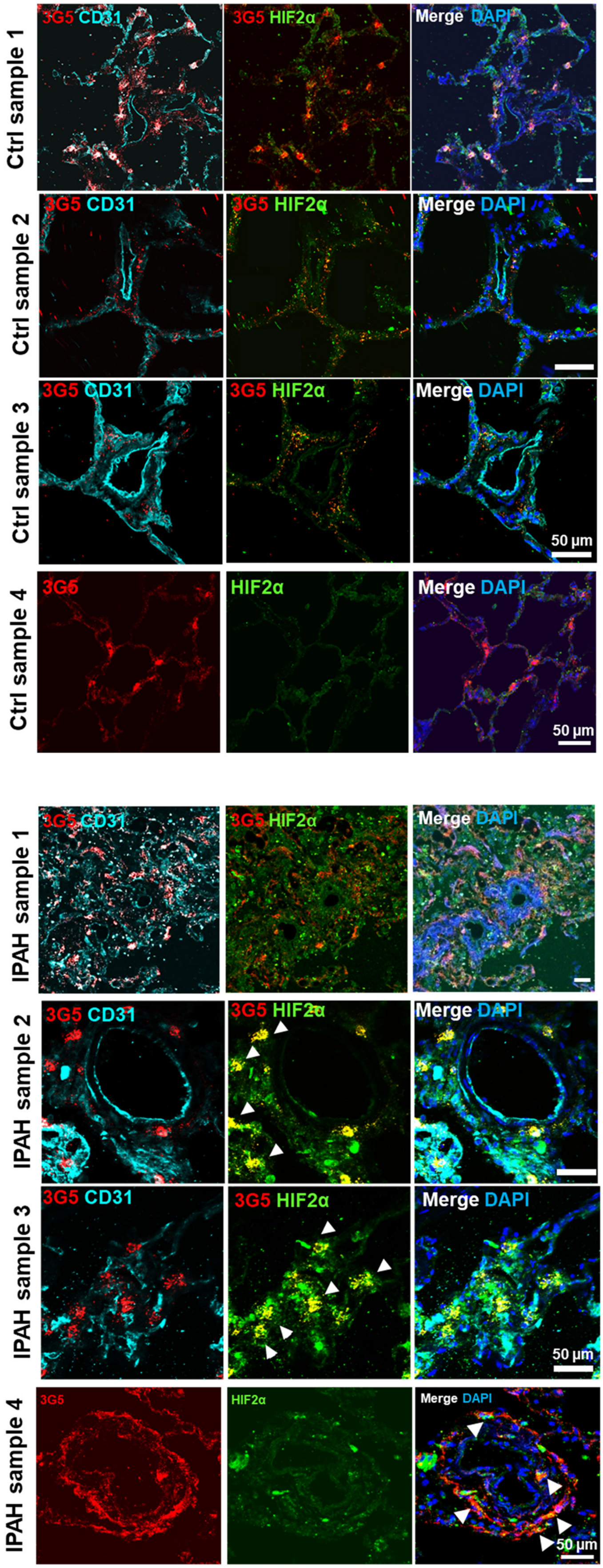

Ctrl sample 1
3G5 CD31
3G5 HIF2α
Merge DAPI
Ctrl sample 2
Ctrl sample 3
50 µm
Ctrl sample 4
3G5
HIF2α
IPAH sample 1
IPAH sample 2
IPAH sample 3
IPAH sample 4

Figure EV2. Four more control and Four more IPAH patient lung tissues were immunofluorescence stained using HIF2α (Green), 3G5 (Red), CD31 (Cyan), and nuclei were stained by DAPI (blue).

3G5 colocalized by HIF2α was indicated by arrows. The higher magnification images are on the bottom two rows. Scale bar = 50 µm.

 © The Author(s)

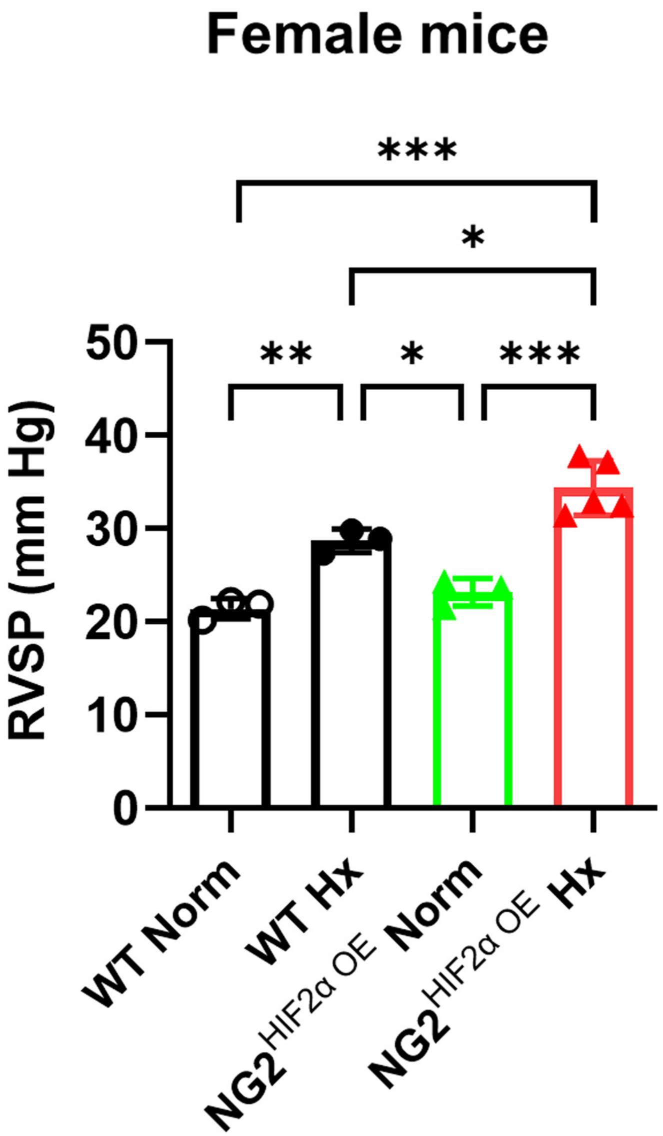

Figure EV3. RVSP measurements were performed on WT and NG2$^{HIF2\alpha\ OE}$ female mice with or without 3-wk Hx.

*Depicts a statistically significant difference: *$P < 0.05$, **$P < 0.01$, and ***$P < 0.001$ (one-way ANOVA with Turkey's multiple comparisons test).

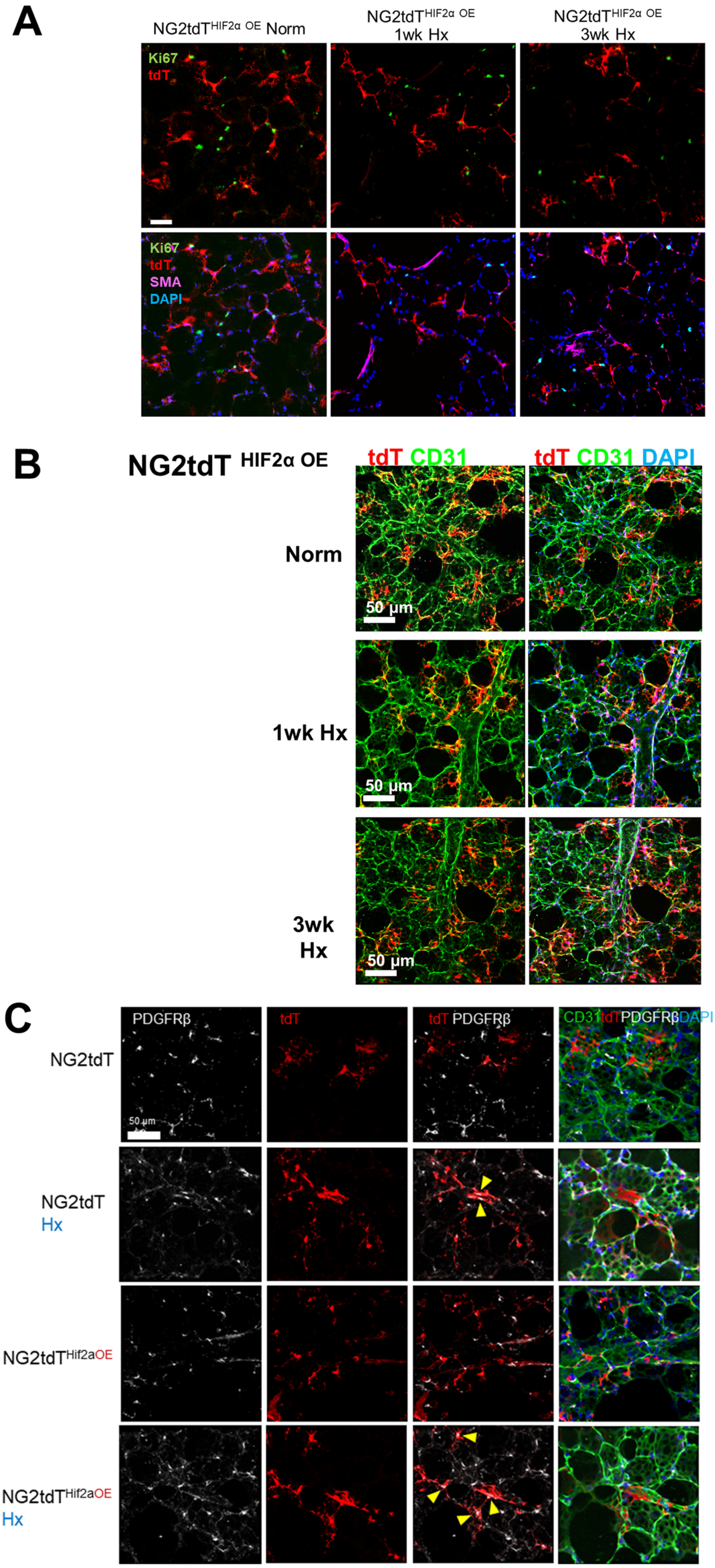
A
NG2tdT HIF2α OE Norm
NG2tdT HIF2α OE 1wk Hx
NG2tdT HIF2α OE 3wk Hx
Ki67
tdT
Ki67
tdT
SMA
DAPI
B
NG2tdT HIF2α OE
tdT CD31
tdT CD31 DAPI
Norm
1wk Hx
3wk Hx
50 μm
50 μm
50 μm
C
PDGFRβ
tdT
tdT PDGFRβ
CD31 tdT PDGFRβ DAPI
NG2tdT
NG2tdT Hx
NG2tdT Hif2aOE
NG2tdT Hif2aOE Hx
50 μm

 © The Author(s)

Figure EV4. Characterization of tdT cells in NG2tdT$^{HIF2\alpha\ OE}$ lungs.

(**A**) tdT cells did not colocalize with Ki67 (cell proliferation marker) under normoxia and hypoxia over time (1 wk and 3-wk Hx), by comparing the precision-cut lung slices of NG2tdT$^{HIF2\alpha\ OE}$ mice. Slices were stained for Ki67 (green), tdTamato (red), SMA (magenta) and DAPI (blue). (**B**) tdT cells closely contacted with CD31(Green) endothelial cells under normoxia and hypoxia over time (1 wk and 3-wk Hx). (**C**) tdT cells from both NG2tdT and NG2tdT$^{HIF2\alpha\ OE}$ co-expressed PDGFRβ (White) under normoxia and 3-wk Hx. Yellow arrowheads indicate tdT wrapped around arterioles and co-expressed PDGFRβ. Scale bar = 50 μm.

© The Author(s)

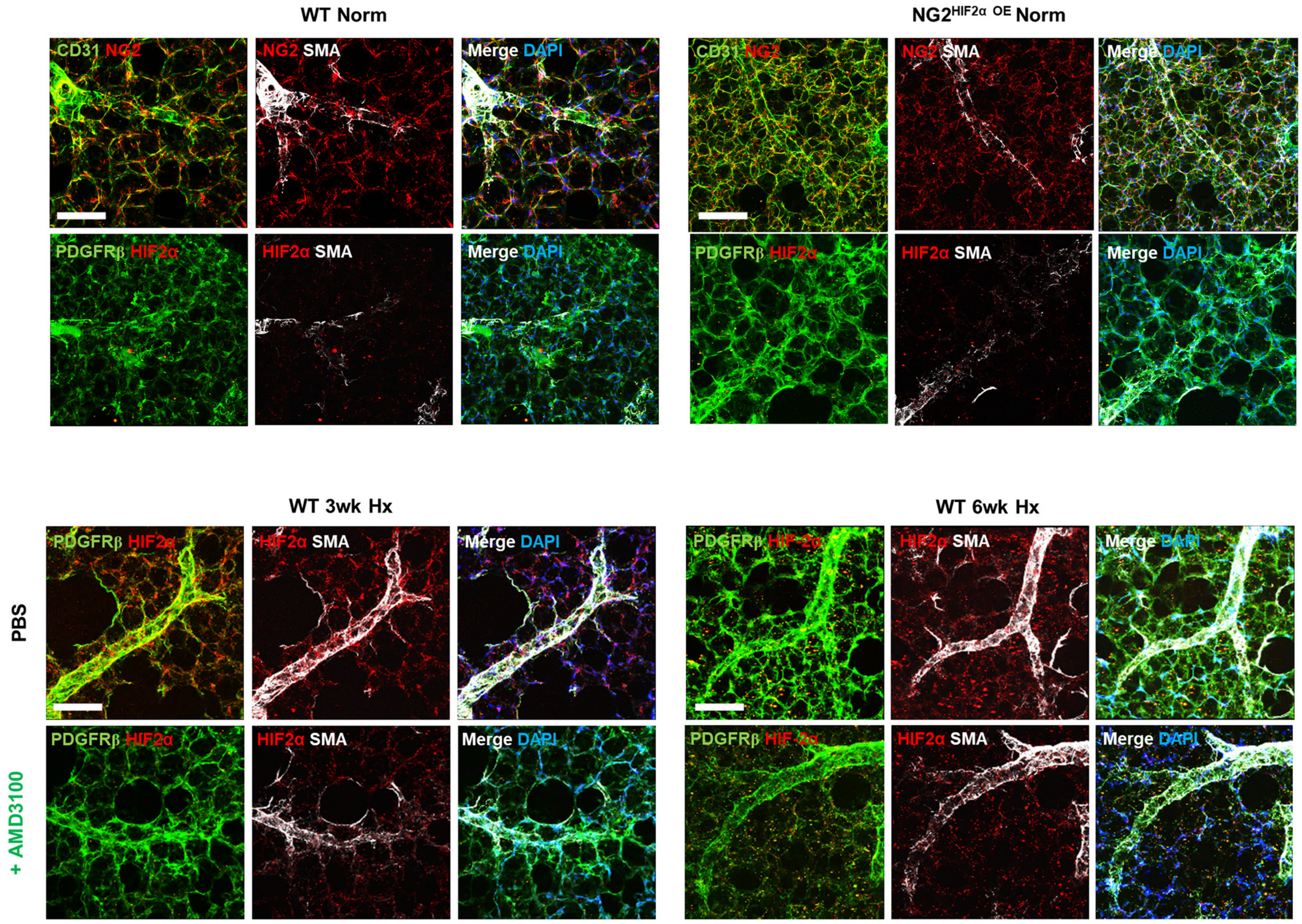

Figure EV5. Treatment of AMD3100 successfully prevents and alleviates vessel muscularization.

The precision-cut lung slices of WT mice in 3-wk (left) and 6-wk (right) hypoxia were stained for PDGFRβ (green), HIF2α (red), and SMA (white) with DAPI (blue) stained for nuclei. Scale bar = 50 μm.

 © The Author(s)
